# Efficient Preparation of Homogenous Antibody Conjugates via Glycosite‐Specific Transglycosylation Enabled by Readily Available Glycosyl Donors

**DOI:** 10.1002/anie.202518579

**Published:** 2026-01-04

**Authors:** Deqin Cai, Yuan Zhao, Gaoyuan Lu, Chunrong Li, Yichong Lao, Ramesh Mudududdla, Jiahao Zhang, Peijing Jia, Penghsuan Huang, Wenxin Wu, Thao‐Vy T. Nguyen, Xuhui Huang, Lingjun Li, Weiping Tang

**Affiliations:** ^1^ Lachman Institute of Pharmaceutical Development School of Pharmacy University of Wisconsin‐Madison Madison WI 53705 USA; ^2^ Biophysics Graduate Program University of Wisconsin‐Madison Madison WI 53706 USA; ^3^ Department of Chemistry University of Wisconsin‐Madison Madison WI 53706 USA; ^4^ Theoretical Chemistry Institute University of Wisconsin‐Madison Madison WI 53706 USA

**Keywords:** Disaccharide oxazoline, IgG glycoengineering, Site‐specific ADCs, Site‐specific DACs

## Abstract

Site‐specific antibody conjugation through glycoengineering offers a promising route to generate homogeneous glycosite‐specific antibody‒drug conjugates (gsADCs) with improved therapeutic indices. Dozens of gsADCs are advancing from preclinical studies to clinical trials. However, current methods involve either multiple enzymes or lengthy preparation of substrates. Herein, we report a novel and synthetically streamlined platform utilizing LacNAc‐derived 4,6‐acetal glycosyl donors for glycosite‐specific transglycosylation mediated by a single enzyme. These glycosyl donors can be synthesized in as few as two steps, representing a major advancement in synthetic accessibility compared to previously reported glycosyl donors, which often require more than 15 steps. Computational analysis showed that the acetal ring restricts conformation, directing donor **7** to a π–π‐stabilized groove of the enzyme. Donor **7**, along with a positive control, was evaluated in the context of gsADCs, consistently demonstrating potent and selective cytotoxicity toward HER2‐positive cancer cells, while sparing HER2‐negative cells. Furthermore, donor **7** was successfully adapted to generate glycosite‐specific degrader‐antibody conjugates (gsDACs), highlighting its broad utility. Additional studies revealed that donor **7** produces antibodies with markedly enhanced resistance to Endo S2 mediated hydrolysis. Together, these findings establish a practical and broadly applicable platform for glycosite‐specific antibody conjugation, paving the way for next‐generation antibody‐based therapeutics.

## Graphical abstract

A streamlined platform for glycosite‐specific antibody conjugation is enabled by LacNAc‐derived cyclic acetal donors, which can be prepared in only two steps and used directly in single‐enzyme Endo S2‐mediated transglycosylation. The platform supports the broad construction of homogeneous gsADCs and gsDACs, with donor **7** producing glycoengineered antibodies that exhibit markedly enhanced stability toward Endo S2‐mediated hydrolysis.

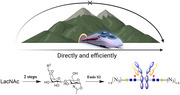

## Introduction

Antibody conjugates bearing diverse functional moieties hold great promise for targeted therapy. Examples include antibody‐drug conjugates (ADCs), degrader‐antibody conjugates (DACs), and antibody‐based lysosome targeting chimeras (LYTACs). ADCs have demonstrated the most rapid clinical progress. The global revenue from currently approved ADCs, along with those in late‐stage clinical development, is projected to reach $26 billion per year by 2028.^[^ ^1^ ^]^

Compared to conventional heterogeneous ADCs, which pose challenges for characterization and quality control, homogeneous ADCs not only overcome these limitations but also frequently demonstrate improved pharmacokinetics, enhanced stability, greater tolerability, and superior in vivo efficacy.^[^ ^2^, ^3^ ^]^ In the past decades, many site‐specific conjugation methods have been reported,^[^ ^2^ ^]^ such as disulfide rebridging,^[^ ^4^, ^5^ ^]^ cysteines engineering,^[^ ^6^, ^7^ ^]^ incorporation of non‐canonical amino acids,^[^ ^8^, ^9^, ^10^, ^11^, ^12^ ^]^ selective C‐/N‐terminal modifications,^[^ ^13^, ^14^ ^]^ enzymatic modification of the antibody's protein backbone,^[^ ^15^, ^16^, ^17^, ^18^, ^19^ ^]^ and glycoengineering.^[^ ^20^, ^21^, ^22^, ^23^, ^24^, ^25^, ^26^ ^]^ Among various site‐specific approaches, glycoengineering at the conserved Fc N297 glycosylation site has emerged as a powerful and broadly applicable platform, owing to its compatibility with native antibodies, its ability to generate truly homogenous antibody conjugates with a single glycoform, and its minimal impact on antigen binding.^[^ ^27^ ^]^ Notably, glycoengineering at the Fc N297 site can potentially adjust antibody‐dependent cellular cytotoxicity (ADCC effect) and immune effector–mediated toxicity, as the Fc glycan modulates interactions with Fcγ receptors on the immune cells and C1q of the complement system.^[^ ^28^, ^29^, ^30^ ^]^ In contrast, non‐glycoengineering conjugation methods yield ADCs with inherently heterogeneous glycan structures, posing risk to product consistency and clinical performance. Clinically, two glycosite‐specific antibody‒drug conjugates (gsADCs), IBI343 and JSKN003, have advanced to phase 3 trials, alongside several others in early clinical development (phase 1/2), highlighting the translational potential of gsADCs.^[^ ^31^, ^32^, ^33^ ^]^

Previously reported Fc glycan engineering strategies typically require at least two different enzymes and rely on structurally complex glycan substrates as the glycosyl donors.^[^ ^33^ ^]^ Recently, Wang's group reported promising IgG glycoengineering methods using disaccharide substrates.^[^ ^21^, ^22^ ^]^ Additionally, Huang and co‐workers have described an enzymatic route to prepare LacNAc‐derived donors through galactose C6 oxidation.^[^ ^20^, ^25^ ^]^ These disaccharide substrates are well‐suited for one‐pot transglycosylation reactions, as they are amenable to transglycosylation yet relatively resistant to deglycosylation, thereby enabling efficient and streamlined conversion.^[^ ^21^ ^]^ Importantly, the shortened glycan length allows the payload to be partially shielded within the IgG Fc cavity, which can significantly enhance the therapeutic index.^[^ ^3^ ^]^ More recently, GeneQuantum Healthcare disclosed a related patent involving trisaccharide substrates.^[^ ^34^ ^]^ The emergence of such strategies in industrial patent filings highlights the growing interest and potential of these methods in antibody conjugate development.

**Figure 1:**
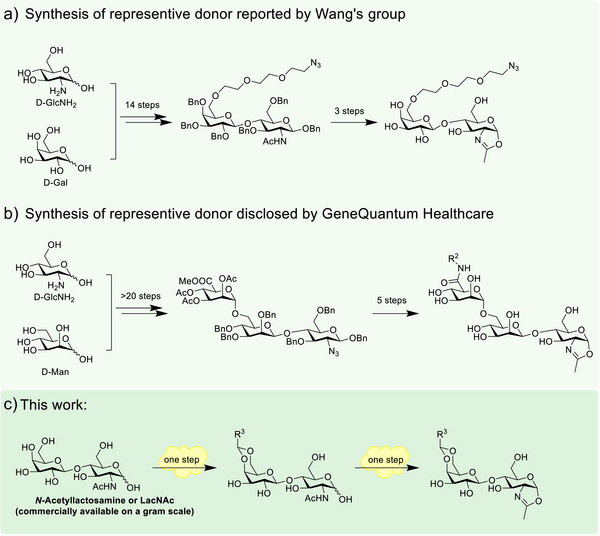
Chemical synthesis of representative oxazoline glycosyl donors from previous reports and this work.

Despite these advantages, a common limitation of these methods is that the reported glycosyl donors or enzyme substrates typically require multiple synthetic steps and involve challenging glycosylation reactions, which limits their accessibility and scalability (Figure 1). For example, the chemical synthesis of even the simplest disaccharide glycosyl donors requires more than 15 steps starting from commercially available monosaccharides. While galactose C6 oxidation strategy is mechanistically elegant, its preparation relies on a multi‐enzyme/O_2_ system of relatively high operational complexity, making the process less suitable for routine synthesis or scale‐up. Clearly, there is an urgent need for a readily accessible glycosyl oxazoline donor, which would make glycoengineering approaches more practical and widely applicable across the scientific and biotherapeutic communities.

In this work, we report the design and synthesis of a series of glycosyl donors with significantly reduced synthetic complexity. Among these, *N*‐acetyllactosamine (LacNAc)‐derived acetal glycosyl donors exhibited the highest efficiency. Notably, they can be synthesized in as few as two steps from commercially available materials (Figure 1), offering an unprecedented level of accessibility that overcomes a major barrier to widespread adoption of previously reported glycoengineering methods. These readily available glycosyl donors enable highly efficient and site‐specific conjugation at the conserved Fc N297 glycosylation site, facilitating the generation of homogeneous antibody conjugates. In contrast, several non‐LacNAc analogs were evaluated but failed to support productive conjugation, providing valuable structure–activity relationship (SAR) insights. Collectively, this study establishes a practical, scalable, and broadly applicable platform for rapid and reliable generation of homogeneous antibody conjugates. The platform holds strong potential for widespread use by the broad research and biotherapeutic development community. It offers a compelling foundation for advancing ADCs, DACs, and other antibody‐based therapeutics.

## Results and Discussion

### Screening of Oxazoline Glycosyl Substrates for One‐Step IgG Glycoengineering

We have previously reported various antibody conjugates as degraders of extracellular and membrane proteins.^[^ ^35^, ^36^, ^37^ ^]^ However, these conjugates are prepared as complex mixtures via non‐selective acylation of lysine residues, posing challenges for characterization, optimization, and translational development. After surveying a range of antibody conjugation methods,^[^ ^2^, ^38^ ^]^ glycoengineering emerged as the most accessible strategy for generating homogenous antibody conjugates from native antibodies. Most notably, the one‐pot transglycosylation reaction mediated by Endo S2 using disaccharide glycosyl substrates appears to be one of the most efficient and direct method, as it employs a single enzyme for both deglycosylation and transglycosylation.^[^ ^21^, ^22^ ^]^ In addition, antibody conjugates bearing a disaccharide motif are relatively resistant to enzymatic deglycosylation, an improvement over those using complex oligosaccharides that mimic natural glycans.^[^ ^21^ ^]^

Furthermore, the synthesis of disaccharide is significantly simpler than that of previously reported glycosyl substrates.^[^ ^26^ ^]^ However, we found that the synthesis of the simplest disaccharide glycosyl substrates from commercially available monosaccharides requires nearly 20 synthetic steps (Figure 1a), and the synthesis of the Man‐GlcNAc glycosyl substrate, known to provide the highest transglycosylation efficiency, requires even more steps.

**Figure 2:**
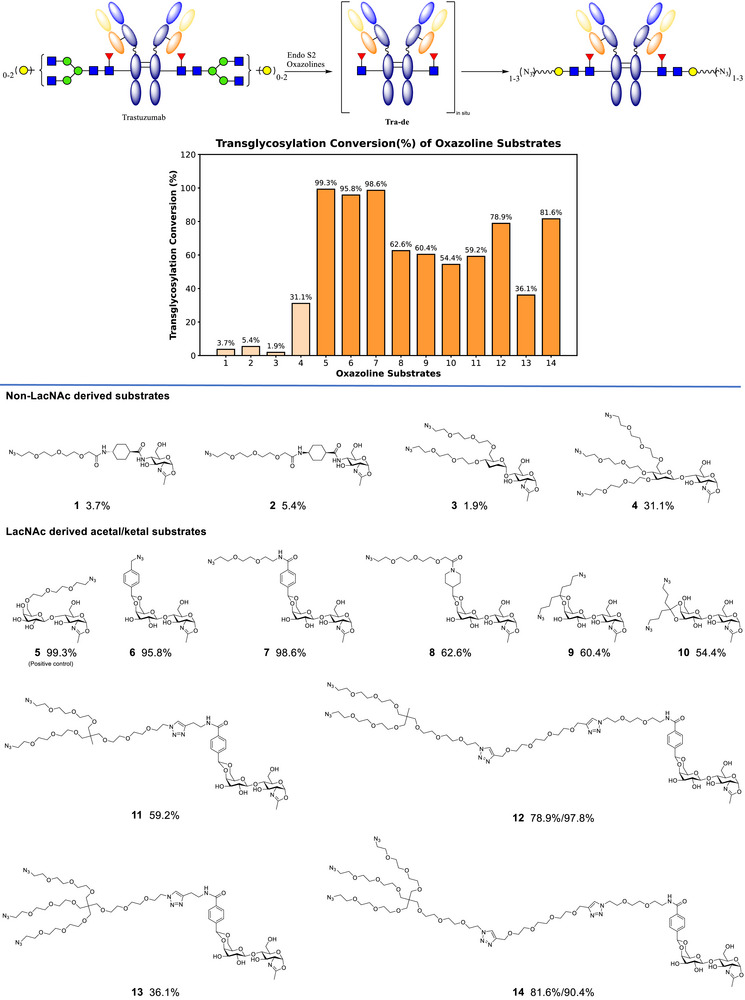
Transglycosylation conversion (%) of trastuzumab with substrates **1**–**14** under standard condition as described in the Supporting Information. Higher conversions of substrates **12** and **14** were achieved after optimized conditions as discussed in the text.

Based on this analysis, we aimed to develop simplified glycosyl substrates by replacing one of the sugar residue distal to the transglycosylation site. This led to the design of functionalized glycosyl substrates **1**–**4** (Figure 2). These simplified substrates can be synthesized in approximately 10 steps, fewer than those required for previously reported substrates. Transglycosylation reactions using these oxazoline glycosyl substrates were carried out under standardized conditions (8% Endo S2 and 20 equiv. of substrate for 1 h) using trastuzumab, a Her2 antibody.

Unfortunately, glycosyl substrates **1**–**4** all exhibited minimal to low activity. Notably, compound **4**—which is structurally similar to a substrate reported by the Wang group but lacks the C2‐hydroxyl group on the mannose unit—showed only 31% conversion, highlighting the structural sensitivity of disaccharide substrates.^[^ ^21^ ^]^ In contrast, the reported disaccharide substrate **5** achieved 99% conversion under the same conditions (Figure 2).

These results suggest that intact disaccharides with most of their hydroxyl groups may be necessary for efficient transglycosylation reaction. Therefore, the most practical route to a suitable glycosyl substrate may involve using a naturally occurring disaccharide that is commercially available in bulk, such as LacNAc. However, site‐selective modification of LacNAc to introduce functional handles, such as azide for drug conjugation, remains a challenge, because LacNAc contains seven hydroxyl groups, including an anomeric hydroxyl. Upon careful analysis of LacNAc's structure, we envisioned that the 4,6‐diol moiety on the galactose unit could be selectively modified in a single step by forming a cyclic acetal with an aldehyde or an acetal (Figure 1c). Previous studies have shown that cyclic acetals on payloads can serve as effective linkers for attaching payloads to antibodies and they are stable under physiological extracellular conditions.^[^ ^39^ ^]^ Additionally, glycosidic bonds, being acetals themselves, are generally regarded stable under physiological conditions.

Although the formation of cyclic acetal from the 4,6‐diol in LacNAc may seem conceptually straightforward, identifying the optimal reaction conditions required extensive effort and systematic investigation, as detailed in Table S1. Our optimization process involved exploring a range of parameters, including starting materials, solvent, reaction temperature, and reaction time. We also found that high vacuum significantly improved the conversion, likely due to efficient removal of methanol and suppression of hydrolysis by maintaining a low water content. Among the solvents tested, DMSO outperformed acetonitrile and DMF, due to the superior solubility of both reactants and its high boiling point, which supports elevated reaction temperatures and high vacuum. Carefully analysis of the byproducts formed during the synthesis of acetal **S29** (an intermediate for substrate **7**) revealed the presence of a side product, **S30** (Figure S1). Based on structural elucidation through total acetylation, NMR, and LC‐MS, **S30** was identified as a 5‐membered ring acetal formed via the 3,4‐OH of galactose. In contrast to the 6‐membered ring acetal **S29**, the acetal ring in **S30** gives rise to two stereoisomers, which were observed in an approximately 1:1 ratio.

In addition, dimethyl acetal exhibited significantly higher reactivity compared to related aldehyde and were essential for achieving good conversion. For substrate **6,** 4‐(azidomethyl)benzaldehyde is relatively small and could still react efficiently without the need for acetal activation. Except this, all other substrates were synthesized from the corresponding acetals/ketals.

A series of acetal‐containing glycosyl substrates were synthesized similarly in two steps from commercially available LacNAc (Figure 2). Gratifyingly, LacNAc‐derived substrates **6**–**14** generally exhibited markedly improved performance. The transglycosylation conversion (%) was assessed by electrospray ionization mass spectrometry (ESI/MS) (Figure S2). The successful transglycosylation of substrate **5**, a known positive control previously reported by Wang's group,^[^ ^22^ ^]^ validated our assay conditions. Among the newly synthesized oxazolines, substrates **6** and **7**, bearing acetals derived from benzaldehydes, achieved the highest conversion (%), comparable to the positive control **5**. Substrate **6**, which lacks the short PEG linker present in **7**, showed slightly reduced activity. Surprisingly, substrate **8**, a structural analog of **7** in which the aromatic ring is replaced with a saturated aliphatic ring, exhibited markedly lower conversion efficiency. We were also pleased to find that compounds **9** and **10**, featuring ketal motifs, were capable of participating in the transglycosylation reaction. Nonetheless, their reduced efficiency compared to benzyl acetals suggests a structural preference for benzyl acetal substrates, further emphasizing the impact of subtle structural features of the substrates on reactivity.

We treated trastuzumab (**Tra**), **Tra‐de** (Fuc‐α‐1,6‐GlcNAc‐trastuzumab), and **Tra‐7** (transglycosylated trastuzumab product using substrate **7**) with PNGase F, which cleaves all N‐glycans on the antibodies. The ESI/MS results of trastuzumab and **Tra‐7** after treatment were consistent, while **Tra‐de** remain unaffected, confirming that substrate **7** was site‐specifically installed at the N‐glycosylation site of trastuzumab (Figure S3).

Multiple azide handles could also be introduced to substrates **11**–**14**. While substrates **11** and **13** demonstrated only moderate conversions, extension of the central PEG spacer (as in **12** and **14**) substantially enhanced the transglycosylation efficiency. Specifically, prolonging the reaction time for **12** (2 h) and adjusting the equivalents and reaction time of **14** (15 equiv. and 3.5 h) can improve the conversion to >90%.

Collectively, these results underscore the critical influence of subtle structural features of the acetal—particularly the presence of an aromatic ring and PEG linkers—highlighting the dependence of catalytic efficiency on substrate recognition and properties. The observed trends offer valuable SAR insights that can guide the rational design of next‐generation glycan‐based conjugation platforms.

Importantly, the optimized substrate **7** proved readily transferrable to multiple antibody scaffolds, including a human IgG1 isotype control (IgG) and Cetuximab (Ctx), yielding highly homogeneous antibody conjugates (Figure S4). This demonstrates that the platform is broadly applicable and not limited to trastuzumab, further supporting its potential as a generalizable approach for site‐specific antibody conjugation.

### Modeling of Interactions of Substrates 5, 7, and 8 with Endo S2

**Figure 3:**
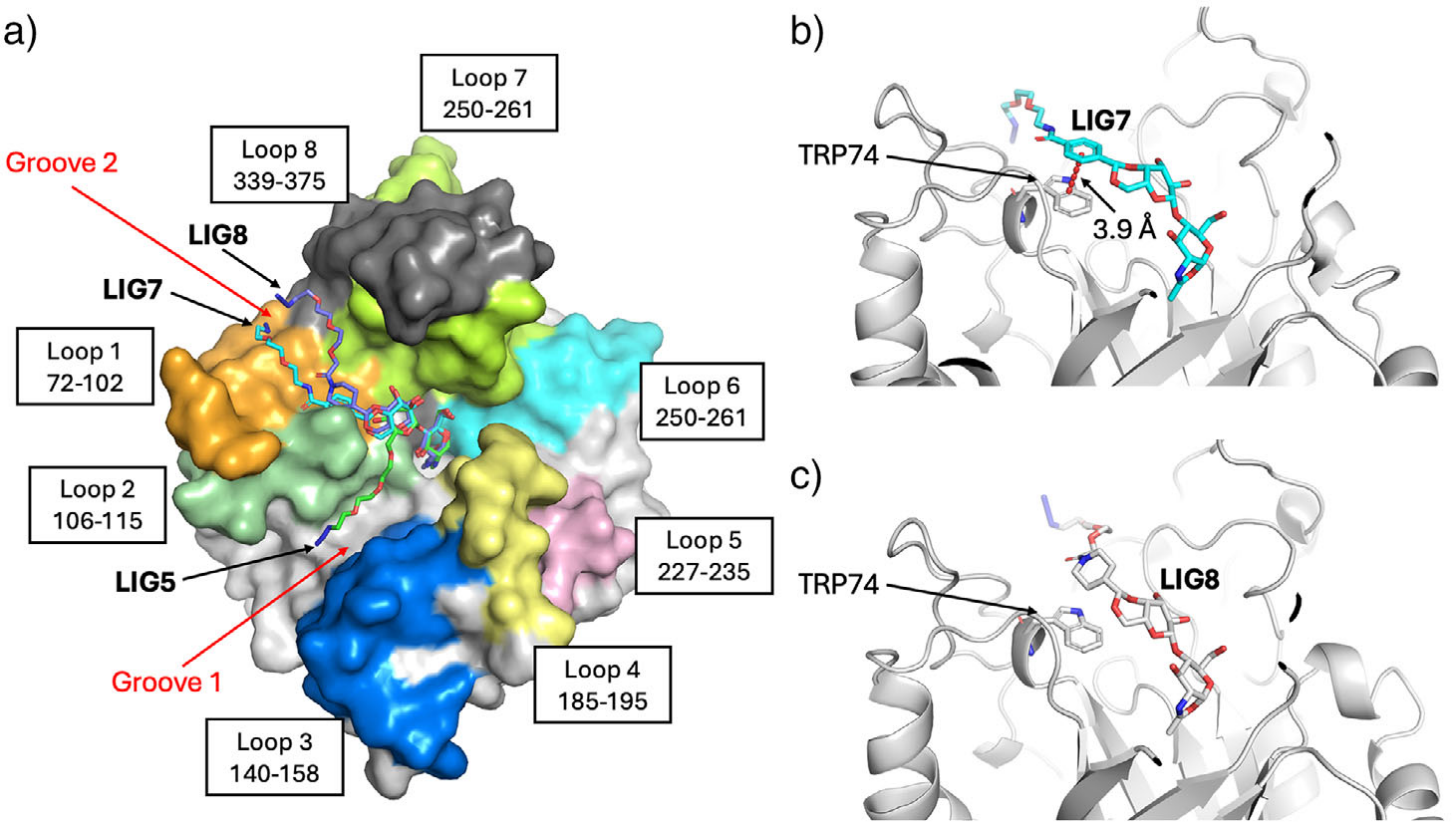
Binding modes of substrates **5**, **7**, and **8** in Endo S2. a) Predicted binding poses of substrates **5**, **7**, and **8** in Endo S2 obtained by Boltz‐2 docking. The enzyme surface is shown in a segmented representation highlighting loop regions that define two distinct glycan‐binding grooves. Representative structure of the Endo S2–substrate **7** complex b) and Endo S2–substrate **8** complex c) from the MD simulations. A π–π stacking interaction is labeled as red dash line between substrate **7** and Trp74.

To gain deeper insights into the molecular basis of substrate recognition by Endo S2, we modeled complex structures on substrates **5**, **7**, and **8** using the Boltz2, followed by 3 independent 100 ns Molecular Dynamics (MD) simulations for each complex (see SI section: Molecular Modeling and MD simulations). Structural analysis of Endo S2 reveals two distinct glycan‐binding grooves located between loop regions (Groove 1 and Groove 2) (Figure 3a). Boltz2 results indicated that substrates **7** and **8** preferentially bind to Groove 2, which is formed between Loop 1 (residues 72–102) and Loop 8 (residues 339–375), whereas substrate **5** predominantly occupies Groove 1. This binding mode difference is attributed to the acetal ring at Gal residue in substrates **7** and **8**, which restricts conformational flexibility and limits their accessible binding orientations, thereby favoring Groove 2. In contrast, substrate **5** lacks such steric constraints and adopts a more flexible conformation that allows productive binding in Groove 1. Interestingly, a previously reported crystal structure (PDB: 6MDS) shows that biantennary N‐glycan can also engage both grooves, supporting the notion that both sites are competent glycan‐recognition regions.^[^ ^40^ ^]^

Further inspection of the complex structures revealed a notable π–π stacking interaction between the benzyl acetal moiety of substrate **7** and Trp74, whereas such interaction is absent in substrate **8**, which lacks the aryl ring (Figure 3b,c). We performed MM/PBSA calculations using the last 50 ns of MD trajectories yielded binding free energies of −92.71 ± 5.60 kcal mol^−1^ for substrate **5**, −90.40 ± 8.15 kcal mol^−1^ for substrate **7**, and −84.38 ± 12.47 kcal mol^−1^ for substrate **8**, which is consistent with the experimental activity trend (**5** ≈ **7** > **8**). These results indicate that although aromatic interactions with Trp74 can contribute to stabilization in the case of substrate **7**, they are not the dominant determinant of substrate efficiency. Instead, overall binding appears to be governed primarily by the compatibility between donor conformation and the spatial constraints of the binding grooves.

### Evaluation of gsADCs Derived from Glycosyl Donors 5 and 7 in Anti‐Proliferation Assays

**Figure 4:**
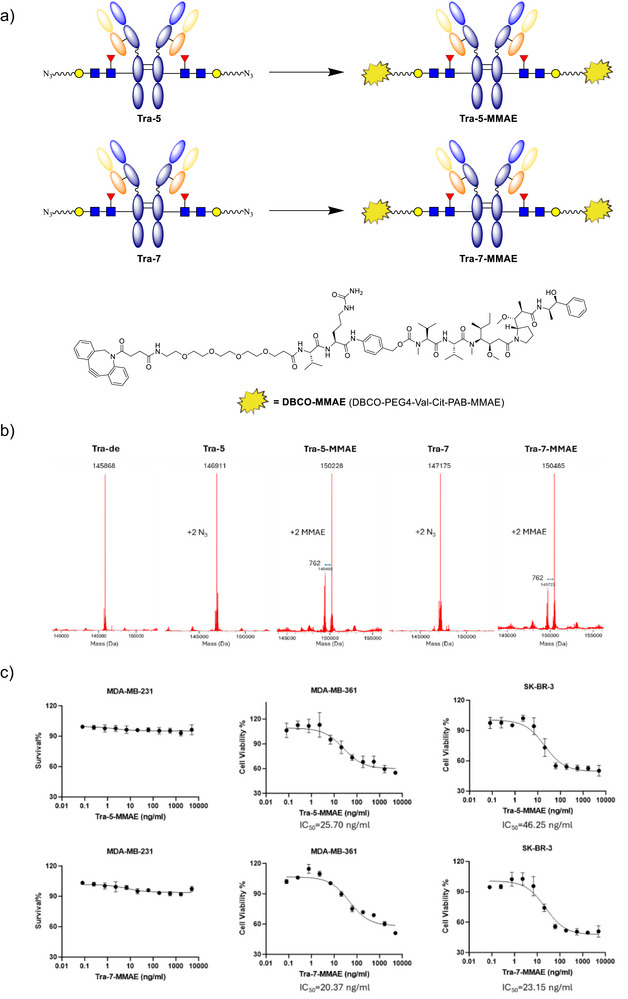
Synthesis, characterization and in vitro evaluation of gsADCs **Tra‐5‐MMAE** and **Tra‐7‐MMAE**. a) Synthesis of structurally defined gsADCs: **Tra‐5‐MMAE** and **Tra‐7‐MMAE**. b) The deconvoluted ESI‐MS spectra. c) In vitro antiproliferation of the two gsADCs against two HER2 positive tumor cell lines, SK‐BR‐3 and MDA‐MB‐361 and HER2 negative tumor cell line MDA‐MB‐231. (*n* = 3).

To assess the translational utility of our glycosyl donors in site‐specific antibody–drug conjugation, trastuzumab was used as the antibody scaffold to generate two gsADCs: **Tra‐5‐MMAE** as the positive control and **Tra‐7‐MMAE**. Both conjugates were constructed via Endo S2‐mediated transglycosylation at the Fc N297 site to generate **Tra‐5** (the transglycosylated trastuzumab product using donor **5**) and **Tra‐7**, followed by strain‐promoted azide–alkyne cycloaddition (SPAAC) with **DBCO–MMAE** (Figure 4a). The conjugation reactions proceeded efficiently for both azide‐modified antibody. The expected molecular masses were confirmed by ESI/MS (Figures 4b and S5). The appearance of dual peaks in intact MS traces results from voltage fragment of the linker‐MMAE moiety. Additionally, analytical size‐exclusion chromatography (SEC) was performed to assess monodispersity of Tra, **Tra‐5‐MMAE**, and **Tra‐7‐MMAE**, all of which showed single, symmetric peaks with no detectable aggregation (Figure S6). Hydrophobic interaction chromatography (HIC) was conducted to provide additional evidence for the DAR of **Tra‐5‐MMAE** and **Tra‐7‐MMAE**, with clean peak shapes and retention shifts consistent with MS. Tra, **Tra‐5**, and **Tra‐7** served as monodisperse controls (Figure S7).

The in vitro cytotoxicity of the two gsADCs was assessed in HER2‐positive (MDA‐MB‐361 and SK‐BR‐3) and HER2‐negative (MDA‐MB‐231) cancer cell lines (Figure 4c). Both conjugates exhibited potent activity against HER2‐positive cells, while showing minimal effects on HER2‐negative cells, highlighting their selectivity and target‐dependent cytotoxicity.

### In Vivo Evaluation of Tra‐5‐MMAE and Tra‐7‐MMAE

**Figure 5:**
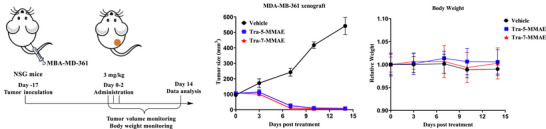
In vivo efficacy assay of **Tra‐5‐MMAE** and **Tra‐7‐MMAE** on the MDA‐MB‐361 xenograft mice model.

To further evaluate the therapeutic potential of **Tra‐7‐MMAE**, it's in vivo antitumor activity was examined in an established MDA‐MB‐361 xenograft model, with **Tra‐5‐MMAE** included as a positive control. Once the tumor volume reached an average 100 mm^3^, mice were randomized and treated with either **Tra‐5‐MMAE**, **Tra‐7‐MMAE**, or vehicle via intraperitoneal (*i.p*.) injection at a dose of 3 mg kg^−1^, once daily for three consecutive days (Figure 5). Both **Tra‐5‐MMAE** and **Tra‐7‐MMAE** elicited marked tumor growth suppression compared to vehicle at the dose of 3 mg kg^−1^. Importantly, treatment with either conjugate did not induce significant body weight loss, indicating good tolerability.

Additional toxicity profiling was conducted to evaluate the safety of the site‐specific conjugates (Figure S8). Hematological evaluation revealed that WBC, RBC, HGB, and PLT values stayed within physiological ranges throughout the study, suggesting that neither **Tra‐7‐MMAE** nor **Tra‐5‐MMAE** induces detectable bone‐marrow suppression. Serum biochemistry demonstrated normal hepatic and renal function, with ALT, AST, ALP, BUN, CRE, and electrolyte levels falling within reference intervals. Histopathological analysis of major organs (liver, spleen, lung, kidney, intestine, and heart) further showed no detectable tissue injury or inflammation in mice treated with **Tra‐7‐MMAE** or **Tra‐5‐MMAE**, and all morphological features remained comparable to PBS controls. Together, these results demonstrate that **Tra‐7‐MMAE** and **Tra‐5‐MMAE** exhibit excellent tolerability under the tested dosing regimen.

Collectively, these results demonstrate that **Tra‐7‐MMAE**, synthesized using an efficiently prepared glycosyl donor, is a gsADC capable of achieving potent antitumor activity and showing good tolerability at the tested dose. These findings support the suitability of donor **7** for constructing gsADCs for further pharmacological exploration.

### Assess the Activity of gsDAC Derived from Glycosyl Donor 7

**Figure 6:**
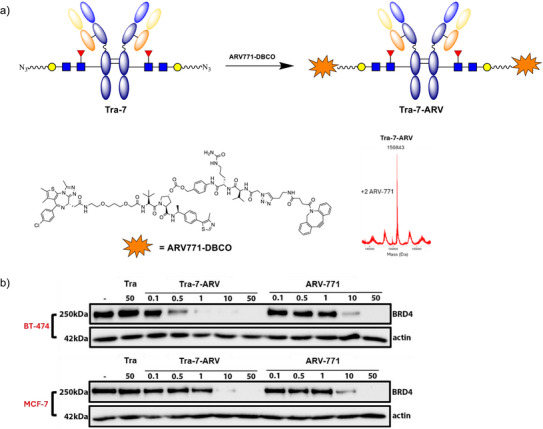
Synthesis and cellular degradation activity of gsDAC **Tra‐7‐ARV**. a) Synthesis of structurally defined gsDAC: **Tra‐7‐ARV** and the deconvoluted ESI‐MS spectrum. b) Western blot analysis of BRD4 degradation in BT‐474 cells and MCF‐7 cells treated with Tra, **Tra‐7‐ARV** and **ARV‐771** for 24 h.

To further demonstrate the utility of this site‐specific antibody conjugation platform, donor **7** was employed in the synthesis of gsDAC. The readily prepared **Tra‐7** was conjugated with **ARV771‐DBCO** to afford gsDAC **Tra‐7‐ARV**, and an untargeted control gsDAC, **IgG‐7‐ARV**, was generated from IgG (Figure S9A). Both constructs were characterized by ESI‐MS (Figure S5). Analytical SEC and HIC further confirmed that **Tra‐7‐ARV**, **IgG‐7‐ARV**, and their precursor antibodies are monodisperse and free of aggregation, each displaying single, symmetric SEC peaks and uniform HIC profiles (Figures S6 and S7). Notably, BRD4 degradation exhibited significant selectivity between HER2‐positive BT‐474 cells and HER2‐negative MCF‐7 cells (Figure 6). In BT‐474 cells, BRD4 degradation was enhanced by

Her2‐mediated uptake, with near‐complete degradation of BRD4 observed at 1 nM of **Tra‐7‐ARV**. In contrast, a ten‐fold higher concentration (10 nM) was required to achieve a comparable effect in MCF‐7 cells. **IgG‐7‐ARV** displayed markedly reduced and similar activity in both cell lines, consistent with its lack of antigen targeting (Figure S9B). The observed trend aligns with previously reported carbonate‐linked HER2‐directed DACs, which also retain measurable activity in MCF‐7 cells despite low HER2 expression (Figure S9C).^[^ ^41^ ^]^ Importantly, **ARV‐771** itself displayed similar activity across both cell lines, indicating that the observed selectivity of the gsDAC is conferred by the antibody conjugate rather than inherited from the parent compound.

### Comparative Evaluation of Substrates 5 and 7 in Glycoengineering Applications

Beyond synthetic streamlining, substrate **7** performs comparably to substrate **5** across key glycoengineering parameters, supporting high transglycosylation efficiency, broad substrate compatibility,^[^ ^21^ ^]^ and similar conjugation yields. The resulting **Tra‐7** and **Tra‐5** conjugates also showed good thermostability, remaining aggregation‐free upon 36 h incubation at 60 °C (Figure S10).

Beyond these comparable properties, substrate **7** exhibits several practical advantages arising from its distinct structural features. First, the glycoengineered antibodies derived from substrate **7** showed markedly enhanced resistance to Endo S2 hydrolysis, with approximately 90% of **Tra‐7** remaining intact after overnight incubation, compared with only ∼7% for **Tra‐5** (Figure S11). Such stability may help minimize undesired enzymatic cleavage during handling and offers a more robust scaffold for further manipulations. Second, the aromatic moiety in substrate **7** confers strong UV absorption, greatly facilitating HPLC monitoring and simplifying routine analytical method development (Figure S12).

Together, these results establish substrate **7** as a synthetically streamlined platform that not only matches the enzymatic performance of substrate **5** but also offers additional functional and analytical conveniences valuable for practical glycoengineering applications.

## Conclusion

In conclusion, we present an efficient platform for site‐specific antibody conjugation based on a newly developed class of LacNAc‐derived acetal donors. Compared to previously reported glycan‐based scaffolds, our LacNAc‐acetal donors offer a significantly streamlined synthetic route, requiring as few as two steps from commercially available LacNAc—thereby improving accessibility and enabling broader application. Through systematic evaluation of non‐LacNAc‐ and LacNAc‐derived glycosyl donors, we elucidated key SARs for the rational design of next‐generation glycan handles for antibody conjugation. Furthermore, computational analysis revealed that the acetal ring in substrate **7** imposes a conformational constraint that directs the molecule into an alternative groove of Endo S2, where an additional π–π interaction provides enhanced stabilization, provides a structural basis for its observed reactivity.

Importantly, glycosyl donor **7** was successfully utilized in the construction of glycoengineered site‐specific antibody–drug conjugates (gsADCs), which demonstrated potent and selective cytotoxicity against HER2‐positive cancer cells while sparing HER2‐negative cells. In vivo, the gsADC derived from donor **7** also displayed robust antitumor activity at the tested dose, underscoring the therapeutic relevance and practical utility of this conjugation platform. In addition, comprehensive hematological and biochemical evaluations confirmed that treatment with **Tra‐7‐MMAE** did not induce detectable systemic toxicity. Further extending the versatility of our approach, donor **7** was adapted for the synthesis of a glycoengineered site‐specific degrader–antibody conjugate (gsDAC), illustrating the platform's applicability across diverse therapeutic modalities.

Notably, glycoengineered antibodies produced from donor **7** exhibited markedly enhanced resistance to Endo S2–mediated hydrolysis. The optimized donor **7** was also readily transferrable to additional antibody scaffolds, including IgG and Ctx, yielding highly homogeneous glycoengineered antibodies and demonstrating the platform's broad applicability across different antibody frameworks.

Taken together, these findings validate the functional utility of our simplified transglycosylation substrates and underscore the transformative potential of glycoengineering strategies for generating homogeneous, targeted, and highly selective antibody conjugates. By addressing major limitations of existing methodologies—including synthetic complexity, limited substrate accessibility, and lack of modularity—our platform offers a practical, scalable, and broadly adaptable solution for site‐specific antibody modification.

This technology facilitates efficient conjugate preparation and provides a robust foundation for the advancement of next‐generation therapeutics, including ADCs, DACs, LYTACs, and other emerging antibody‐based modalities. As such, it holds strong promise for widespread adoption in both academic and industrial settings, accelerating the development of innovative treatments for a range of diseases.

## Supporting Information

The Supporting Information including additional figures, table, experimental details, materials, methods, and NMR spectra. The authors have cited additional references within the Supporting Information.^[^ ^42^, ^43^, ^44^, ^45^, ^46^, ^47^, ^48^, ^49^, ^50^, ^51^, ^52^, ^53^, ^54^, ^55^, ^56^, ^57^, ^58^, ^59^, ^60^, ^61^ ^]^

## Conflict of Interests

A patent application related to this manuscript has been filed. D.C., Y.Z., and W.T. are listed as inventors.

## Supporting information



Supporting Information

## Data Availability

The data that support the findings of this study are available in the supplementary material of this article.
