# Supplementary material for: Efficient Preparation of Homogenous Antibody Conjugates via Glycosite‐Specific Transglycosylation Enabled by Readily Available Glycosyl Donors
Source: Angew Chem Int Ed Engl. 2026 Jan 4;65(7):e18579. doi: 10.1002/anie.202518579 (PMC12887610; doi:10.1002/anie.202518579)
Supplement: Supplementary file 1 — Supporting Information [file ANIE-65-e18579-s001.pdf]

Efficient Preparation of Homogenous Antibody Conjugates via Glycosite-Specific Transglycosylation Enabled by Readily Available Glycosyl Donors

Deqin Cai,[1,†] Yuan Zhao,[1,†] Gaoyuan Lu,[1,†] Chunrong Li,[1,†] Yichong Lao,[2,3] Ramesh Mudududdla,[1] Jiahao Zhang,[1,2] Peijing Jia,[1] Penghsuan Huang,[3] Wenxin Wu,[3] Thao-Vy T. Nguyen,[1] Xuhui Huang,[3,4] Lingjun Li,[1,3] and Weiping Tang[1,3,*]

[1] Dr. D. Cai, Y. Zhao, G. Lu, Dr. C. Li, Dr. R. Mudududdla, J. Zhang, P. Jia, T. Nguyen, Prof. Dr. L. Li, Prof. Dr. W. Tang
Lachman Institute of Pharmaceutical Development, School of Pharmacy, University of Wisconsin-Madison, Madison, WI, 53705, USA
E-mail: weiping.tang@wisc.edu

[2] Y. Lao, J. Zhang
Biophysics Graduate Program, University of Wisconsin-Madison, Madison, WI, 53706, USA.

[3] Y. Lao, P. Huang, Dr. W. Wu, Prof. Dr. L. Li, Prof. Dr. W. Tang
Department of Chemistry, University of Wisconsin-Madison, Madison, WI, 53706, USA

[4] Prof. Dr. X. Huang
Theoretical Chemistry Institute, University of Wisconsin-Madison, Madison WI, 53706, USA

†These authors contributed equally.

Table of Contents

Supplementary table and figures S2
Experimental section S19
- **Methods of electrospray ionization mass spectrometry (ESI-MS)** S19
- **General procedures for transglycosylation activity evaluation of sugar oxazolines** S19
- **PNGase F treatment to confirm reaction site** S19
- **Molecular Modeling and MD Simulations** S19
- **Synthesis of gsADCs Tra-5-MMAE and Tra-7-MMAE** S20
- **Synthesis of IgG-7 and Ctx-7** S20
- **Synthesis of gsDACs Tra-7-ARV and IgG-7-ARV** S20
- **Size-exclusion Chromatography (SEC) Analysis of Antibody Conjugates** S20
- **Hydrophobic Interaction Chromatography (HIC) Analysis of Antibody Conjugates** S20
- **Stability analysis of gsADCs** S21
- **Cell culture** S21
- **Cell Viability Assay** S21
- **BRD4 degradation** S21
- **In Vivo Efficacy Evaluation** S22
- **In Vivo Toxicity Evaluation** S22
- **Synthesis of oxazoline substrates 1-14** S23
- **Synthesis of ARV771-DBCO** S44

NMR S46
HRMS S91
Reference S102

Supplementary table and figures

Table S1. Optimization of the reaction conditions for one-step acetal formation

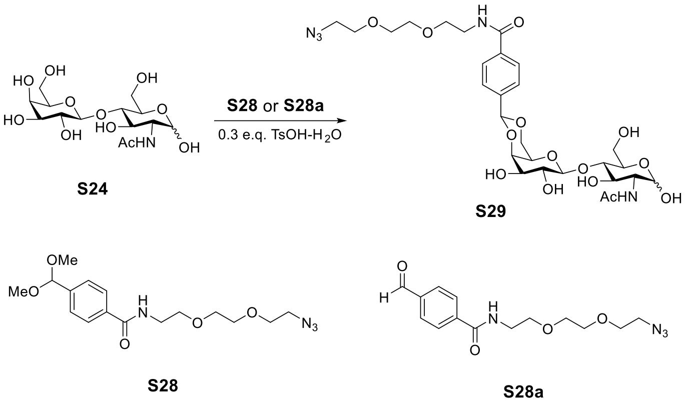

Entry	Reagent	T (℃)	t (h)	solvent	yield	Methods
1	**S28a**	50	5	ACN	<5%*	A
2	**S28a**	reflux	5	ACN	<5%*	A
3	**S28**	70	5	ACN	<5%*	A
4	**S28a**	70	16	DMF	<5%*	A
5	**S28a**	70	16	DMSO	<5%*	A
6	**S28a**	70	5	DMSO	~20%*	B
7	**S28**	70	5	DMSO	44.5%**	A
8	**S28**	70	3	DMSO	61.0%**	B

*The yield was estimated by TLC, **Isolated yield.

Method A: To a solution of LacNAc (1.0 e.q.) and **S28** or **S28a** (1.2 e.q., co-distilled with toluene before use) in dry solvent (0.2 M) was added TsOH-H_2O (0.3 e.q.). The reaction mixture was heated at indicated temperature and monitored by TLC with MeOH/DCM = 1/3. Additional **S28** or **S28a** (up to 3 e.q. in total) can be added to promote conversion.

Method B: A solution of LacNAc (1.0 e.q.) and **S28** or **S28a** (1.2 e.q.) in DMSO (0.2 M) was co-distilled with toluene twice at 40 ℃ to remove residual water. TsOH-H_2O (0.3 e.q.) was then added, followed by additional co-distillation with toluene under the same conditions. The mixture was subsequently continue rotated at 70 ℃ under around 10 mbar for 2-3 h. The reaction was monitored by TLC with MeOH/DCM = 1/3 every 30 min and followed by toluene co-distilled once. Additional **S28** or **S28a** (up to 3 e.q. in total) could be added if necessary to drive the reaction to the end.

A

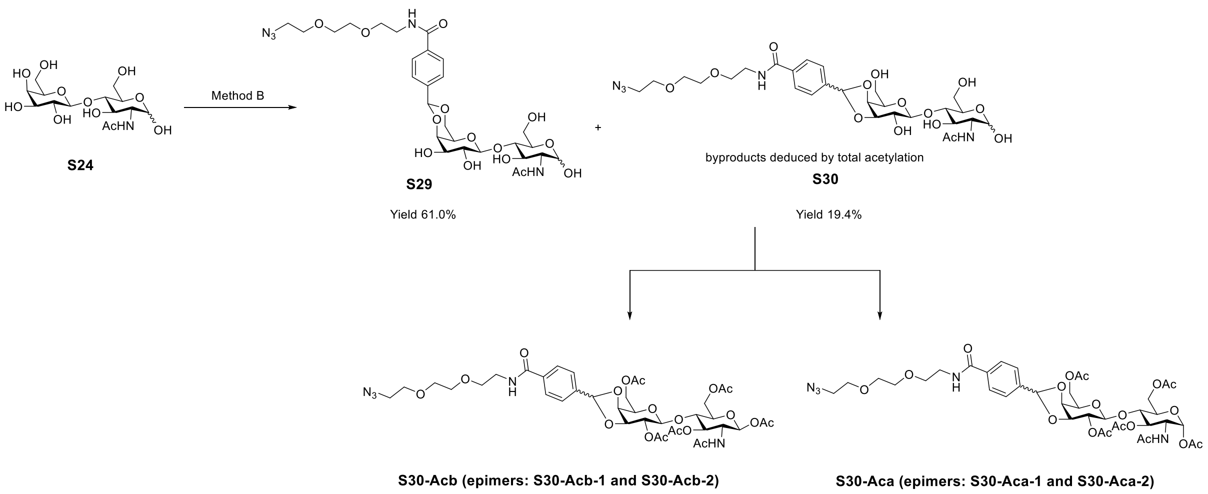

B

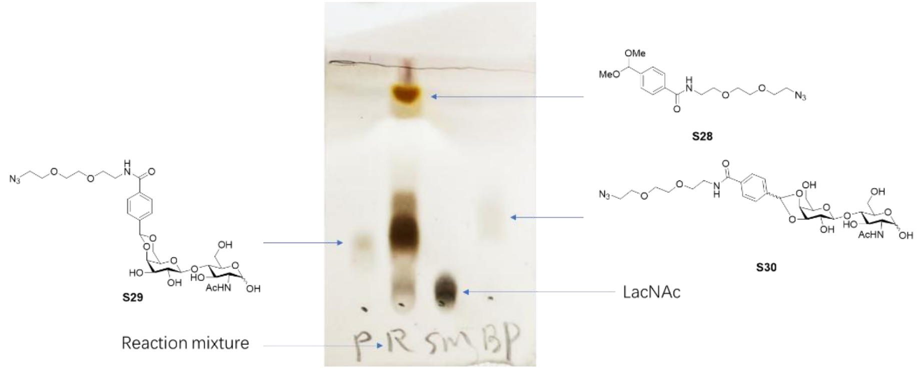

C

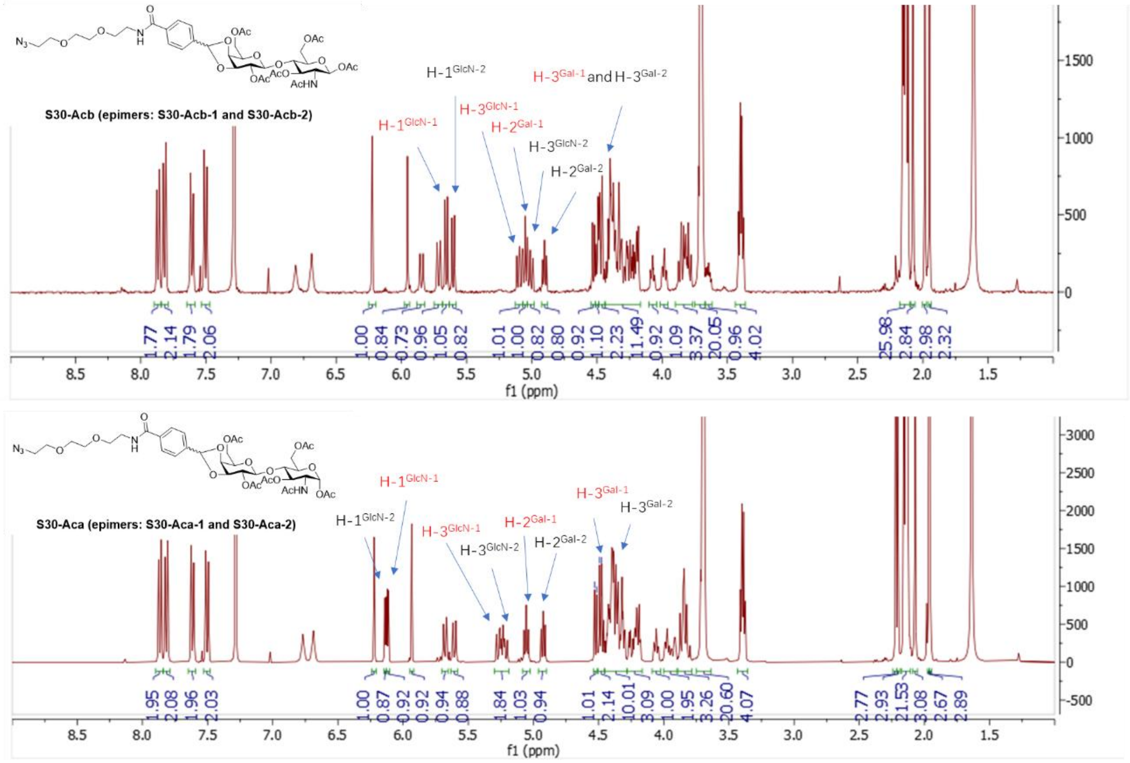

Figure S1 Characterization of the byproduct formed during the synthesis of **S29** under optimized

conditions. The byproduct is a mixture of 5-member ring acetals in a 1:1 ratio. The structures were deduced by total acetylation, LCMS and COSY. The assignment of sugar ring protons supports the proposed structure. As indicated in the ^{1}H NMR spectrum, protons adjacent to acetylated positions resonate downfield (δ > 4.8 ppm), whereas protons at unacetylated positions appear upfield (δ < 4.6 ppm). (A) Synthetic route for the identification byproduct, (B) TLC analysis of the reaction mixture with MeOH/DCM = 1/3. (C) Key ^{1}H NMR assignment of fully acetylated byproducts.

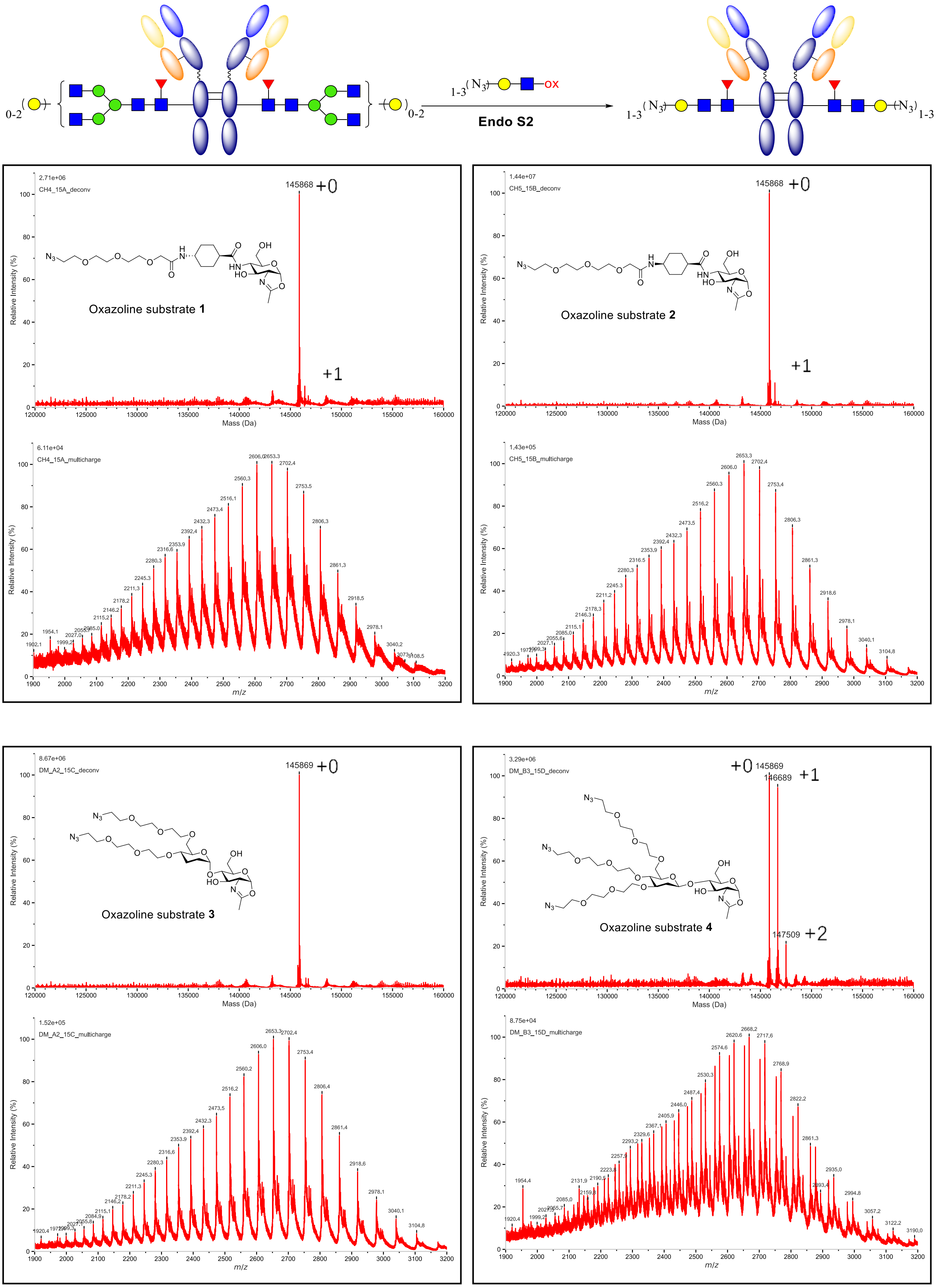

Endo S2
2.71e+06
CH4_15A_deconv
145868 +0
+1
Oxazoline substrate 1
Mass (Da)
Relative Intensity (%)
6.11e+04
CH4_15A_multicharge
m/z
1.44e+07
CH5_15B_deconv
145868 +0
+1
Oxazoline substrate 2
1.43e+05
CH5_15B_multicharge
8.67e+06
DM_A2_15C_deconv
145869 +0
Oxazoline substrate 3
1.52e+05
DM_A2_15C_multicharge
3.29e+06
DM_B3_15D_deconv
+0 145869
146689 +1
147509 +2
Oxazoline substrate 4
8.75e+04
DM_B3_15D_multicharge

Continue

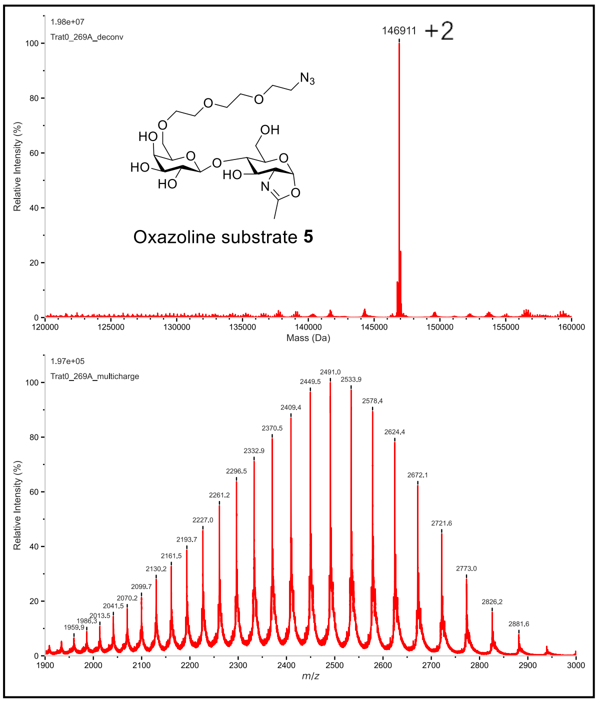
1.98e+07
Trat0_269A_deconv
146911 +2
Oxazoline substrate **5**
Relative Intensity (%)
Mass (Da)
1.97e+05
Trat0_269A_multicharge
m/z

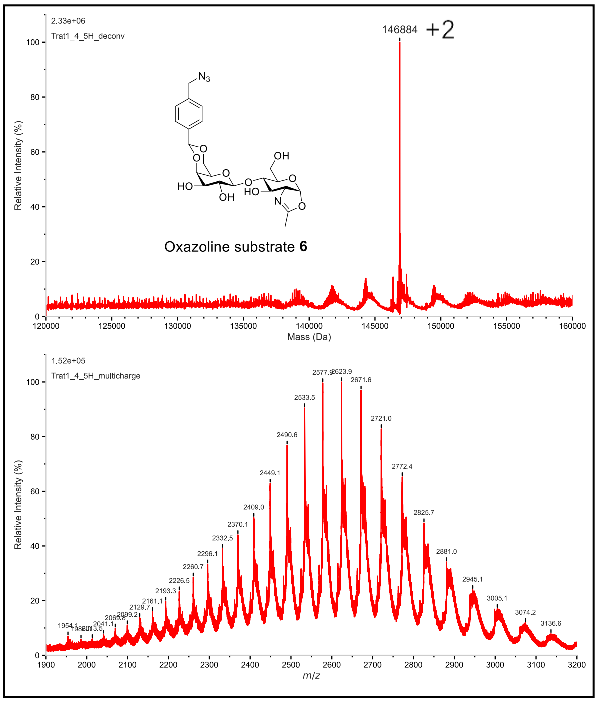
2.33e+06
Trat1_4_5H_deconv
146884 +2
Oxazoline substrate **6**
Relative Intensity (%)
Mass (Da)
1.52e+05
Trat1_4_5H_multicharge
m/z

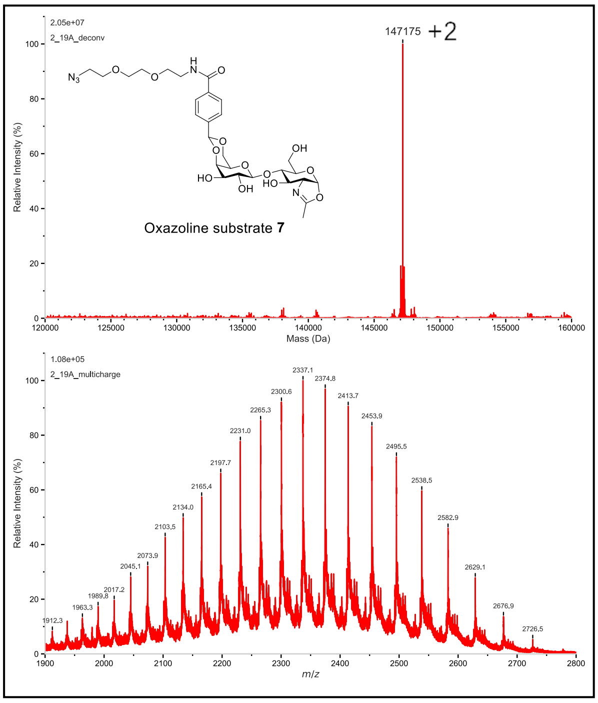
2.05e+07
2_19A_deconv
147175 +2
Oxazoline substrate **7**
Relative Intensity (%)
Mass (Da)
1.08e+05
2_19A_multicharge
m/z

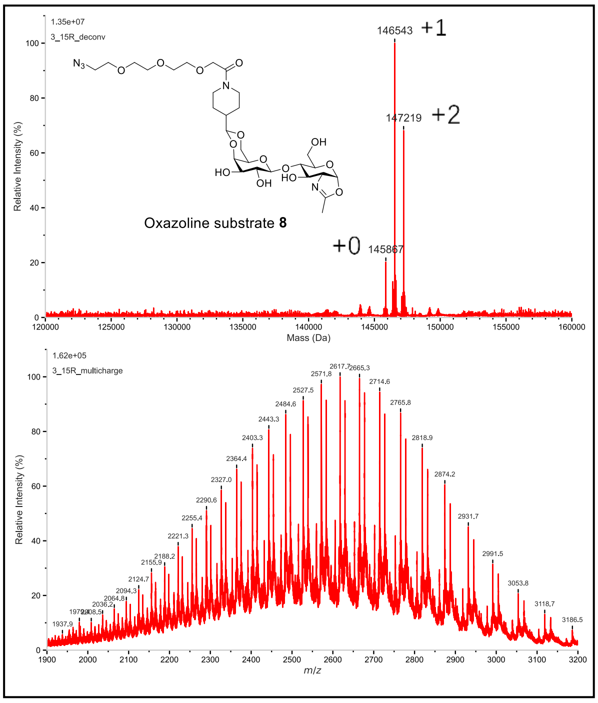
1.35e+07
3_15R_deconv
146543 +1
147219 +2
+0 145867
Oxazoline substrate **8**
Relative Intensity (%)
Mass (Da)
1.62e+05
3_15R_multicharge
m/z

Continue

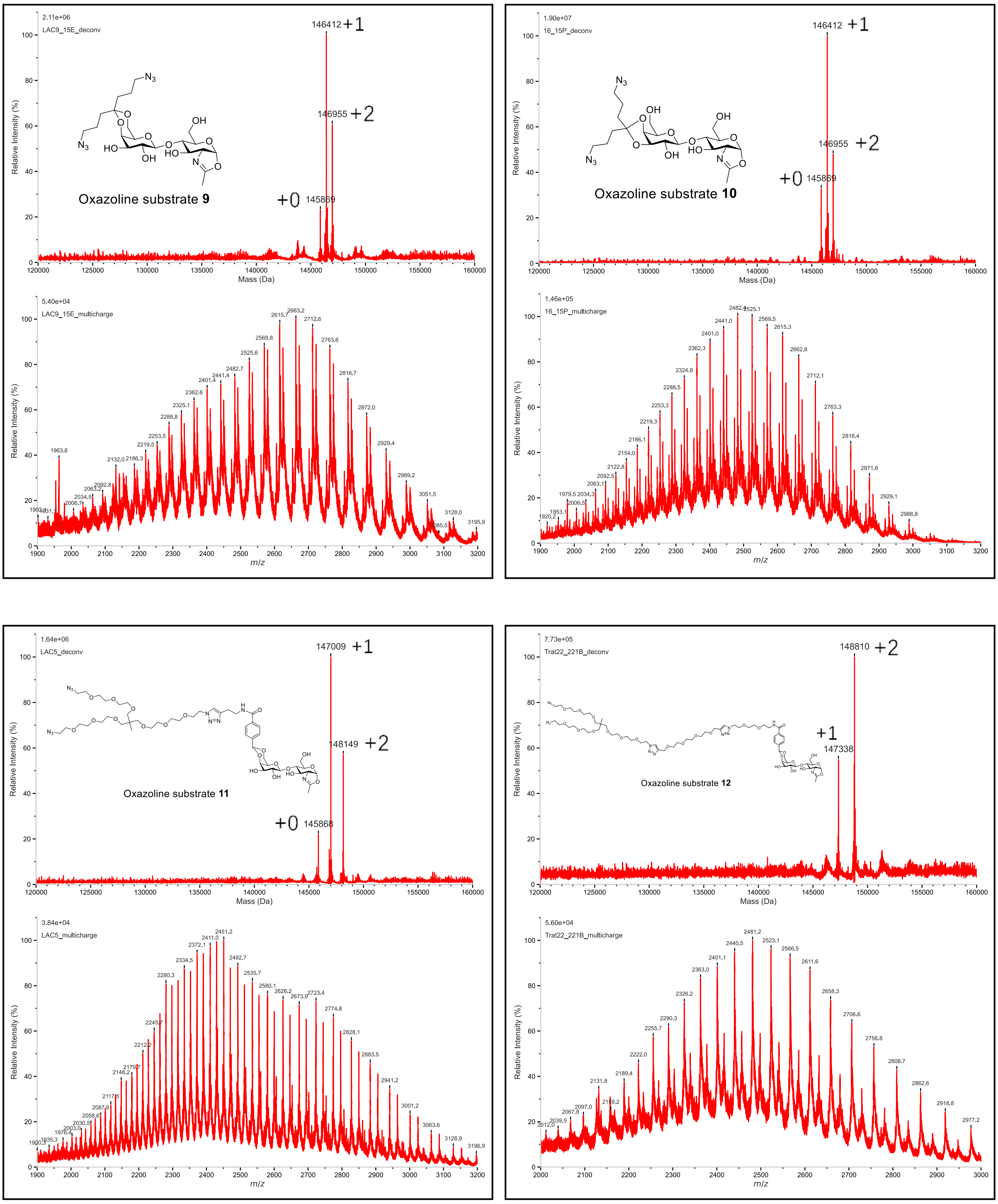
2.11e+06
LAC9_15E_deconv
146412 +1
146955 +2
+0 145869
Oxazoline substrate 9
Relative Intensity (%)
Mass (Da)
5.40e+04
LAC9_15E_multicharge
m/z
1.90e+07
16_15P_deconv
146412 +1
146955 +2
+0 145869
Oxazoline substrate 10
1.46e+05
16_15P_multicharge
1.64e+06
LAC5_deconv
147009 +1
148149 +2
+0 145868
Oxazoline substrate 11
3.84e+04
LAC5_multicharge
7.73e+05
Trat22_221B_deconv
148810 +2
+1 147338
Oxazoline substrate 12
5.60e+04
Trat22_221B_multicharge

Continue

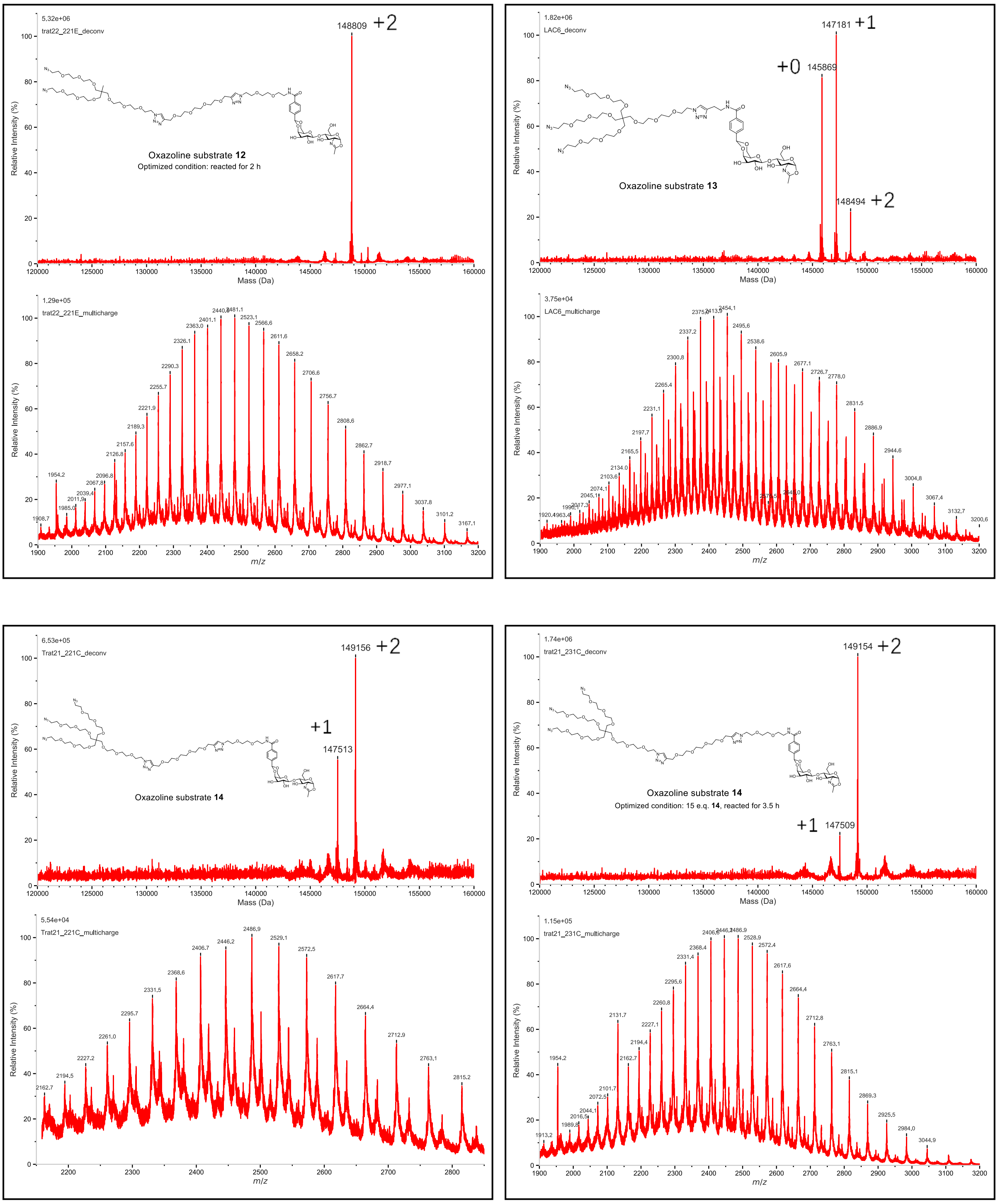

Figure S2 ESI-MS spectra of Endo-S2 catalyzed transglycosylation of trastuzumab with indicated oxazoline substrates.

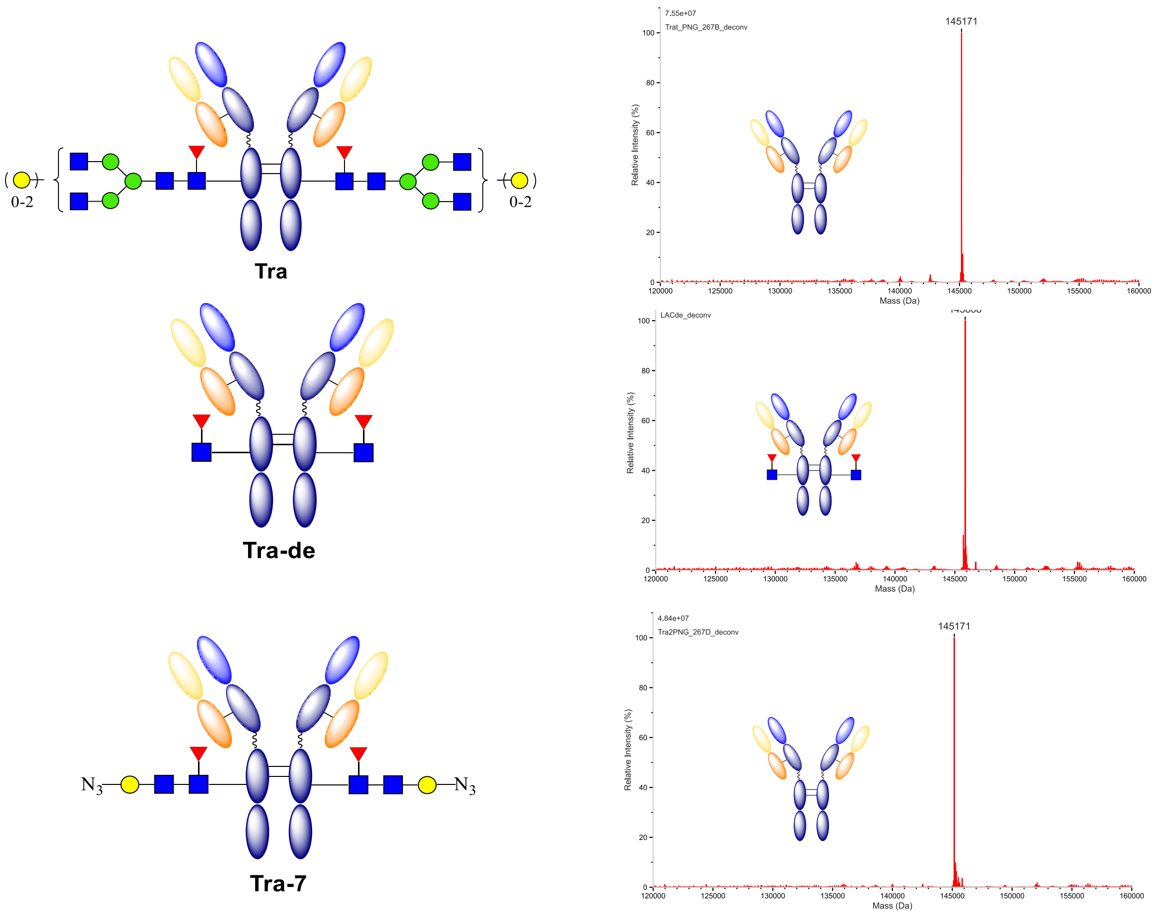

Figure S3 Digestion of Trastuzumab, **Tra-de** and **Tra-7** with PNGase F to identify the conjugation site.

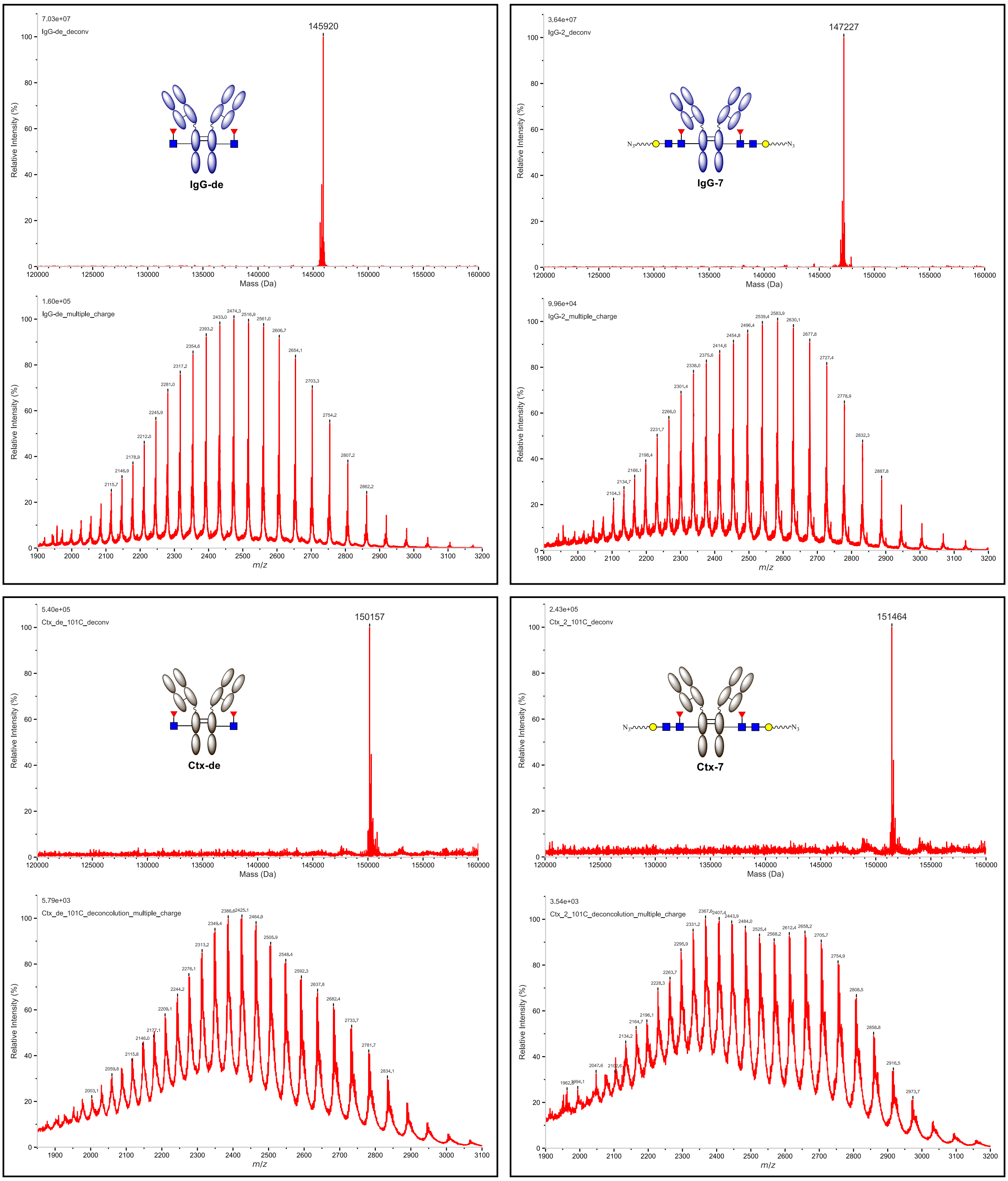

Figure S4 ESI-MS spectra of Endo-S2–catalyzed transglycosylation of Human IgG1 isotype control (IgG) and Cetuximab (Ctx) with oxazoline substrate **7**. **IgG-de** and **Ctx-de** refer to the corresponding antibodies whose native Fc N-glycans were trimmed by Endo-S2 in the absence of substrate **7**.

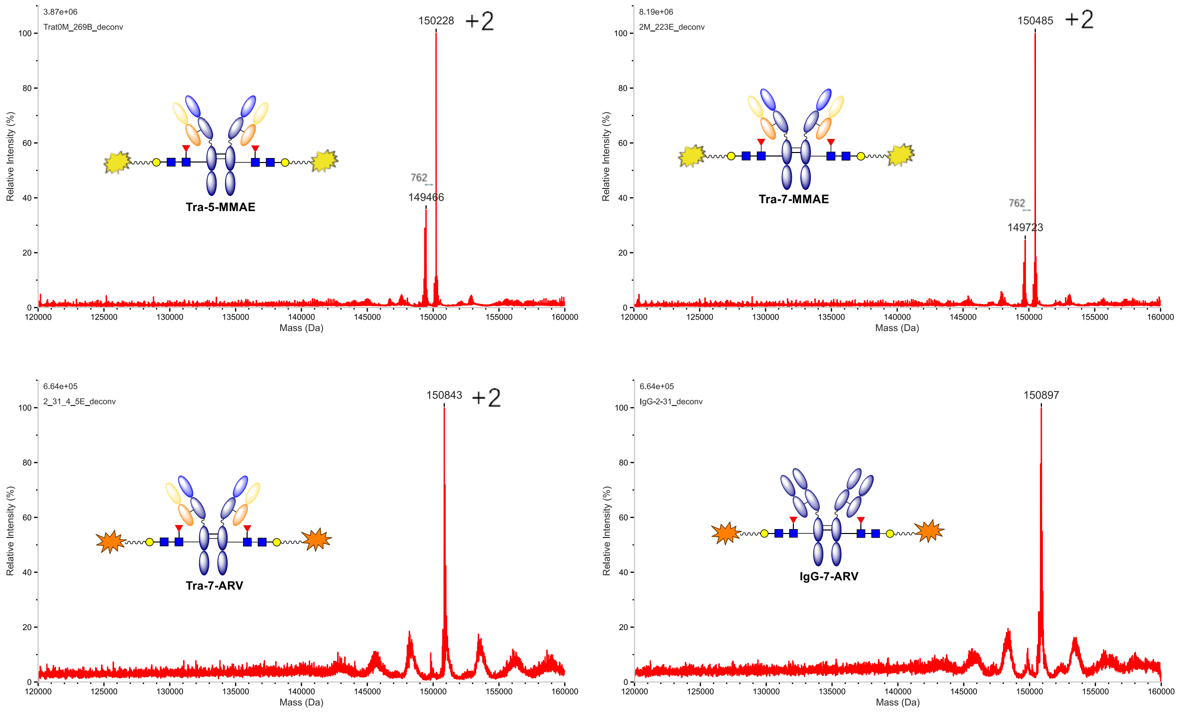

Figure S5 Deconvoluted ESI-MS spectra of **Tra-5-MMAE**, **Tra-7-MMAE**, **Tra-7-ARV** and **IgG-7-ARV**.

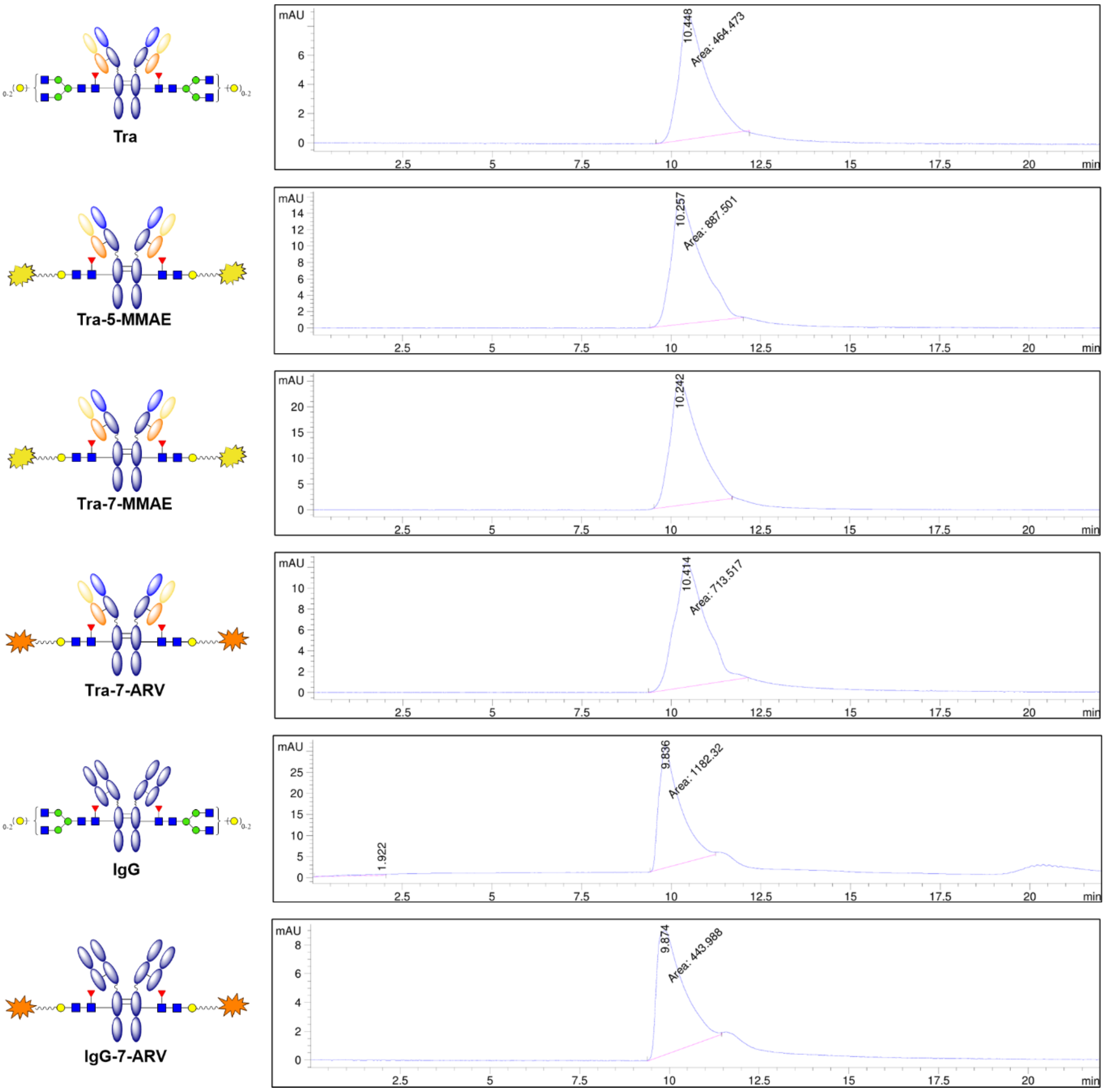

Figure S6 SEC analysis of ADCs (**Tra-5-MMAE**, **Tra-7-MMAE**), DACs (**Tra-7-ARV**, **IgG-7-ARV**) and their corresponding parent antibodies.

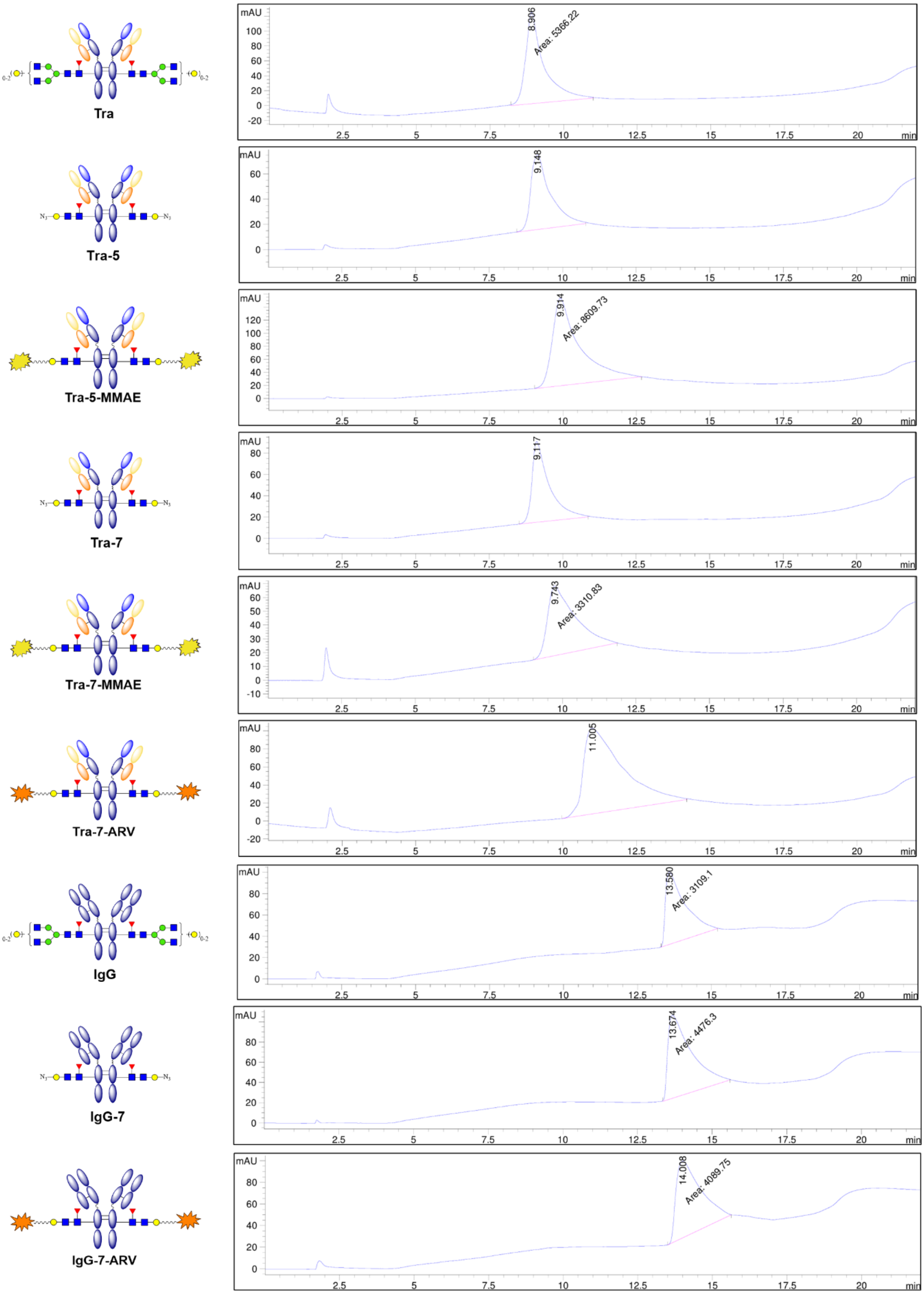

Figure S7 HIC analysis of ADCs (**Tra-5-MMAE**, **Tra-7-MMAE**), DACs (**Tra-7-ARV**, **IgG-7-ARV**), and the corresponding parent antibodies and glycoengineered intermediates.

A

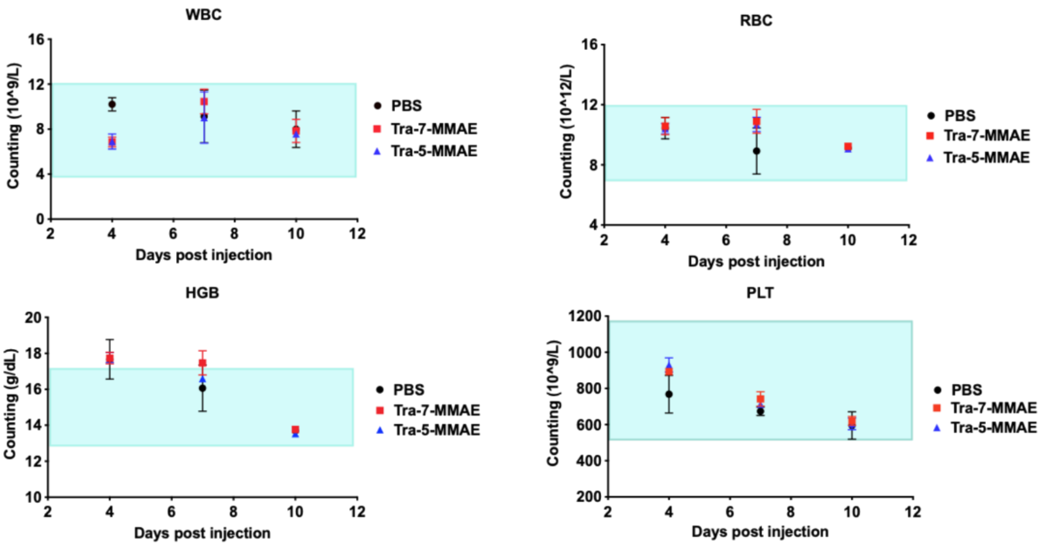

B

Blood Chemistry

Index		PBS	Tra-7-MMAE	Tra-5-MMAE	Reference
Hepatic function	ALT	29	22	25.5	39-70 U/L
	ALP	123	137.5	145.5	76-143 U/L
	AST	98.5	88	104	50-100 U/L
	TBIL	0.3	0.3	0.3	0.1-0.3 mg/dL
	ALB	4.1	4.05	4.35	2.4-3.9 g/dL
	TP	5.3	5.5	5.6	4.7-6.0 g/dL
Kidney function	CRE	0.2	0.2	0.2	0.1-0.3 mg/dL
	GLOB	1.2	1.45	1.25	1.7-2.7 g/dL
	GLU	211.5	180	169.5	150-250 mg/dL
	BUN	21	19	21	14-28 mg/dL
	NA+	153	153	157	145-155 mmol/L
	K+	7.45	7.6	7.45	4.5-9.0 mmol/L
	CA	11.5	11.05	11.5	8.8-11 mg/dL

C

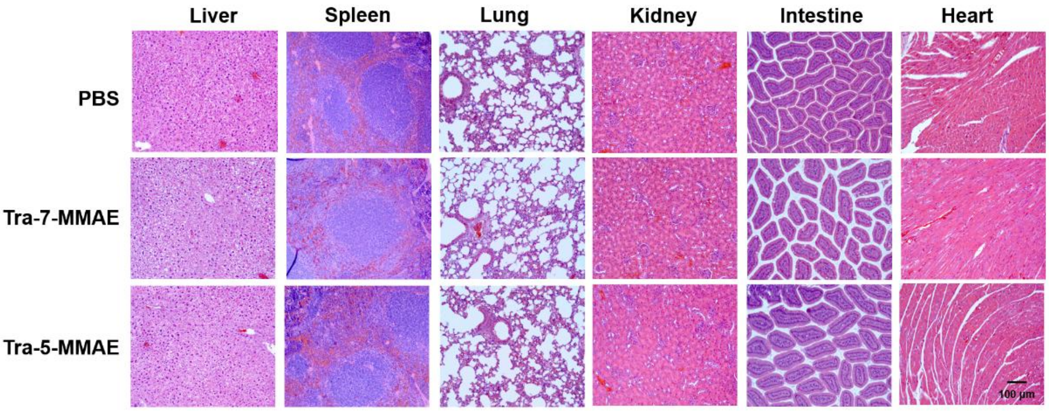

Figure S8 The toxicity evaluation of **Tra-7-MMAE** and **Tra-5-MMAE**. (A) Hematology (CBC): complete blood counting, including WBC, RBC, HGB, and PLT, measured on days 4, 7, and 10 post-injections. (B) Serum chemistry analysis: blood chemistry parameters were evaluated on day 10, including ALT, AST, ALP, TBIL, ALB, TP, CRE, GLOB, GLU, BUN, Na^+, K^+, and Ca^{2+}. (C) Histopathological analysis

(H&E staining): representative H&E-stained sections of liver, spleen, lung, kidney, intestine, and heart collected on day 10 after three daily injections of PBS, **Tra-7-MMAE** (3 mg/kg), or **Tra-5-MMAE** (3 mg/kg). Scale bar: 100 μm.

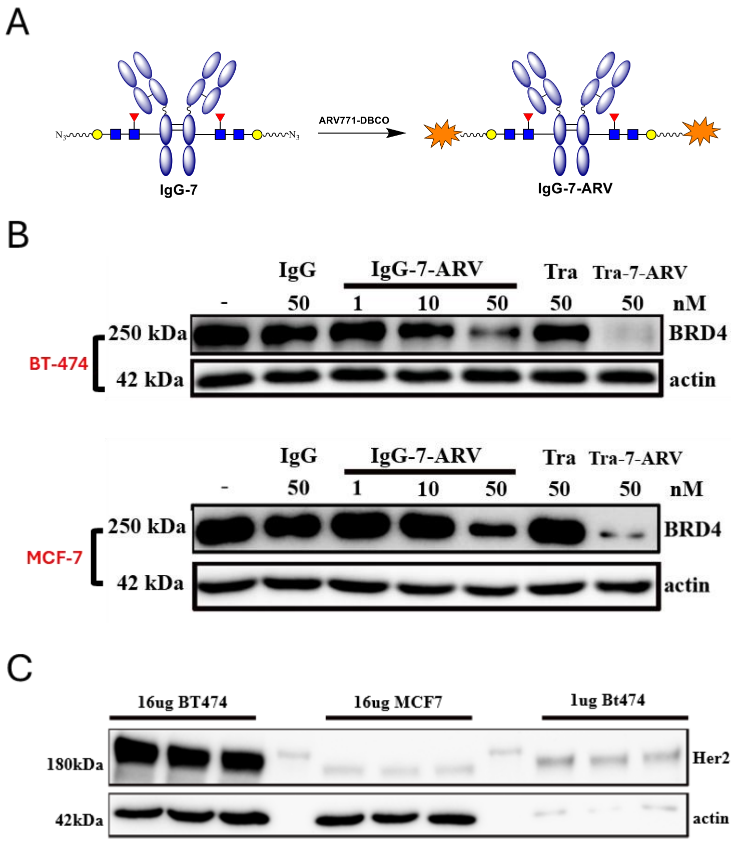

Figure S9 Synthesis and cellular activity of the non-targeting gsDAC control **IgG-7-ARV**. (A) Synthesis of structurally defined gsDAC: **IgG-7-ARV**. (B) Western blot analysis of BRD4 degradation in BT-474 cells and MCF-7 cells treated with IgG, **IgG-7-ARV** and **Tra-7-ARV** for 24 h. (C) Western blot analysis of HER2 expression in BT-474 and MCF-7 cells. HER2 is highly expressed in BT-474, while low but detectable levels are present in MCF-7. For comparison, 1 μg of BT-474 lysate and 16 μg of MCF-7 lysate were loaded.

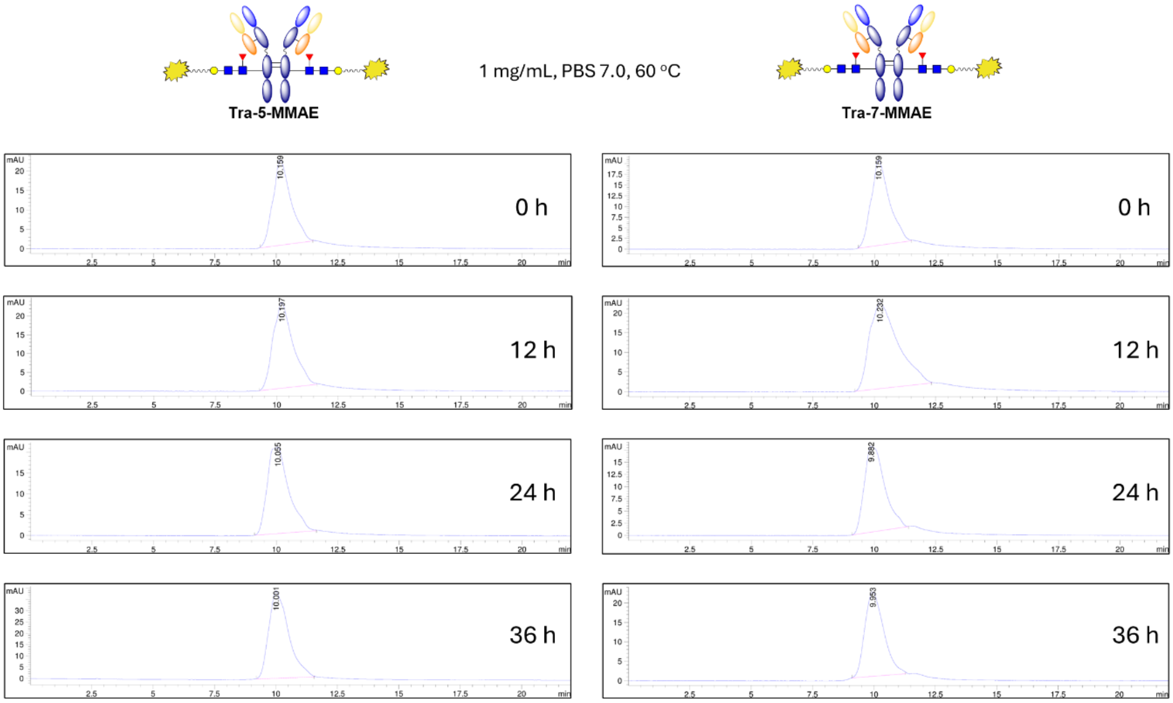

Figure S10 SEC analysis of ADCs **Tra-5-MMAE** and **Tra-7-MMAE** after incubation at 60 °C for 0, 12, 24, and 36 h.

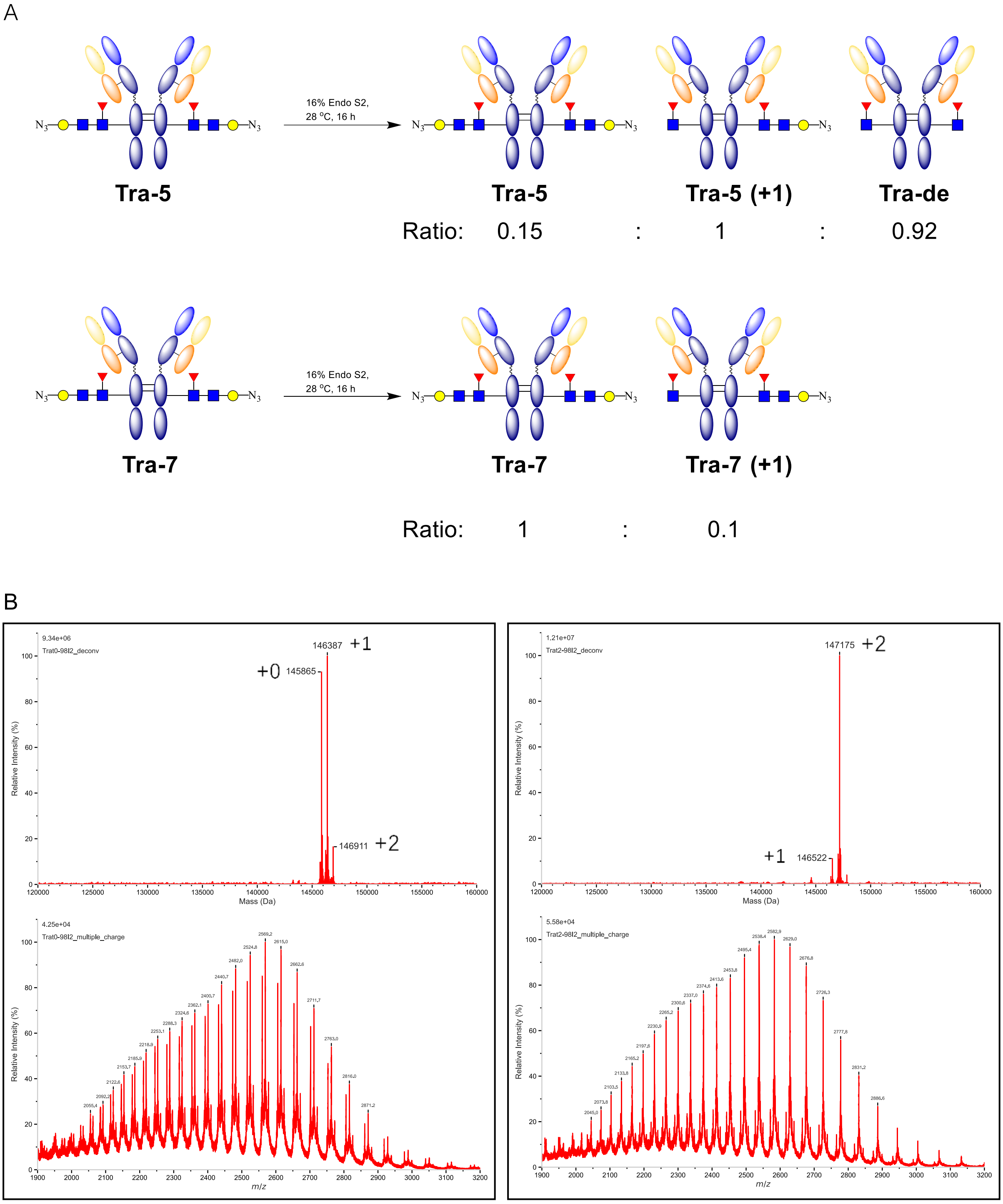

Figure S11 Stability of glycoengineered antibodies toward Endo S2–mediated hydrolysis. Only 7.2% of **Tra-5** remained intact after Endo S2 treatment (calculated as 0.15 / (0.15 + 1 + 0.92)), whereas 90.9% of **Tra-7** preserved its glycan structure under the same conditions. (A) N-glycan hydrolysis profiles of **Tra-5** and **Tra-7** after incubation with 16% Endo S2 at 28 °C for 16 h. The relative abundances of the +0, +1, and deglycosylated species reveal that **Tra-7** exhibits markedly higher resistance to Endo S2 digestion than **Tra-5**. (B) ESI-MS spectra of the corresponding reaction mixtures after desalting by a 50 kDa molecular weight cutoff filter.

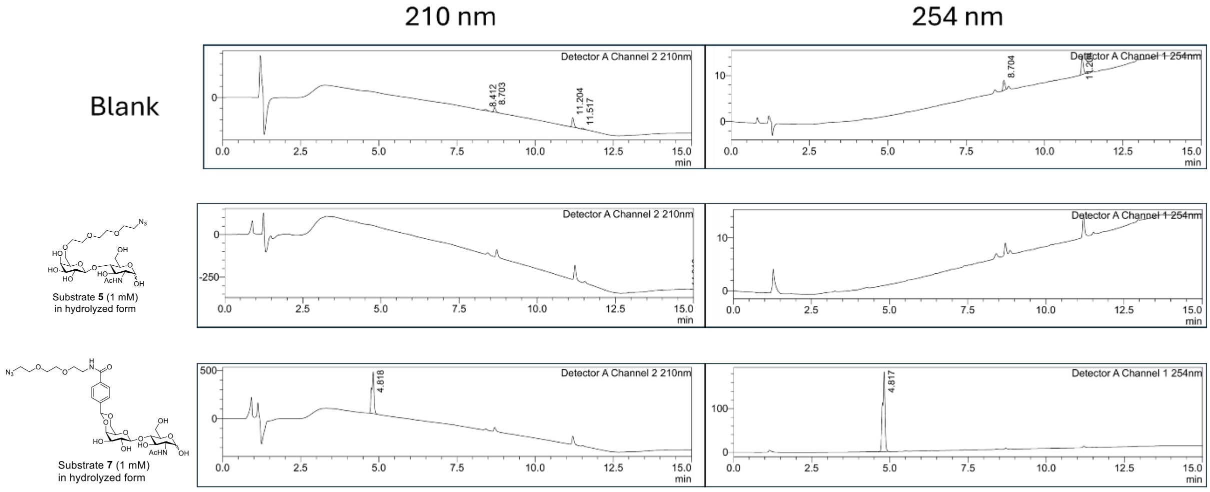

Figure S12 HPLC-UV analysis of substrate **5** and substrate **7** in their hydrolyzed forms, monitored at 210 nm and 254 nm on a Shimadzu Nexera LC-40 HPLC. Substrate **7** shows clear UV absorbance at both wavelengths, whereas substrate **5** displays negligible UV signal under identical conditions.

Experimental section

Methods of electrospray ionization mass spectrometry (ESI-MS)

The antibody samples, previously quantified by NanoDrop, were diluted to 0.1 mg/mL in 50% aqueous acetonitrile containing 0.1% formic acid. The prepared sample was directly infused at a flow rate of 100 µL/min into a Waters Select Series Cyclic IMS mass spectrometer. Data were acquired in positive ion mode across a mass range of *m/z* 100–4000. The electrospray ionization (ESI) source was operated with the following parameters: capillary voltage, 3.0 kV; cone voltage, 15 V; source offset, 30 V; source temperature, 150 °C; desolvation gas flow, 800 L/h; and nebulizer gas pressure, 7 Bar. The acquired mass spectra of the multiply charged antibody conjugate species were deconvoluted to produce a zero-charge spectrum, focusing on the mass range of 120,000 to 160,000 Da.

General procedures for transglycosylation activity evaluation of sugar oxazolines

A solution of Wild-type trastuzumab (Trastuzumab-ANNS (Kanjinti) from UW Health Pharmacy) (150 µg), sugar oxazolines (20 e.q.), and PBS buffer (500 mM, pH 6.5, 7.6 µL) was incubated with Endo-S2 (from Cell Science, Cat. No.: YXX06601B, 12 µg in 2.4 µL of 10 mM PBS, pH 7.4) at 28 °C for 1 h. The reaction mixture was desalted using distilled water through a 50 kDa molecular weight cutoff filter, repeated seven times. The resulting modified antibodies were characterized by electrospray ionization mass spectrometry (ESI-MS) to confirm transglycosylation.

PNGase F treatment to confirm reaction site

A solution of Wild-type trastuzumab or **Tra-de** or **Tra-7** (70 µg) was incubated with PNGase F (1 µL, 500 U/µL) at 30 °C for 12 h. The reaction mixture was desalted using distilled water through a 50 kDa molecular weight cutoff filter, repeated seven times. The resulting modified antibodies were characterized by electrospray ionization mass spectrometry (ESI-MS).

Molecular Modeling and MD Simulations

The complex structures of Endo S2 with substrates **5**, **7**, and **8** were generated using Boltz-2[1], employing 10 recycling iterations and 25 output samples per run. The highest-ranking predicted model for each complex was selected for subsequent molecular dynamics (MD) simulations. Each complex was placed in a cubic simulation box with a minimum solute-to-boundary distance of 1.5 nm and solvated using TIP3P[2] water molecules. Sodium and chloride ions were added to neutralize the system and to achieve a physiological ionic strength of 0.15 M.

All systems were energy-minimized using the steepest descent method prior to equilibration. A two-stage equilibration procedure was performed, consisting of 1 ns of NVT simulation at 300 K followed by 1 ns of NPT simulation at 1 bar. During equilibration, harmonic positional restraints (force constant: 1000 $kJ \cdot mol^{-1} \cdot nm^{-2}$) were applied to protein heavy atoms. Temperature was controlled using the velocity-rescale thermostat[3] with a coupling constant of 0.1 ps, while pressure was maintained using the Parrinello–Rahman barostat[4] with a coupling constant of 2 ps.

Production MD simulations were then carried out for 100 ns without restraints. Long-range electrostatics were treated using the Particle Mesh Ewald (PME)[5-6] method with a real-space cutoff of 1.2 nm, and van der Waals interactions were truncated at the same cutoff. All covalent bonds involving hydrogen atoms were constrained using the LINCS[7] algorithm, allowing an integration timestep of 2 fs via the leap-frog integrator. All MD simulations were performed with GROMACS 2022.5.[8] The CHARMM36m[9]

force field was used to model protein interactions, and ligand parameters were assigned using the CHARMM General Force Field (CGENFF)[10].

Binding free energy calculations were conducted using the gmx_MMPBSA[11] package following the molecular mechanics/Poisson–Boltzmann surface area (MM/PBSA) framework. The solute–solvent dielectric boundary was defined using a level-set approach[12], and nonpolar solvation contributions[13] were approximated as a SASA-dependent term. Dielectric constants were set to 1.0 for the solute interior and 80.0 for the solvent. A halogen-optimized atomic radius set (mbondi_pb2[14]) was employed, and the salt concentration was set to 0.15 M for all calculations.

Synthesis of gsADCs Tra-5-MMAE and Tra-7-MMAE

A solution of wild-type trastuzumab (600 µg), oxazolines **5** or **7** (20 e.q.), and PBS buffer (500 mM, pH 6.5, 30.4 µL) was incubated with Endo-S2 (48 µg in 9.6 µL of 10 mM PBS, pH 7.4) at 28 °C for 1 h. The reaction mixture was immediately purified using protein A resin to afford corresponding azide modified trastuzumab: **Tra-5** (340 µg) and **Tra-7** (346 µg) as quantified by NanoDrop, respectively.

A solution of azide-modified trastuzumab (**Tra-5** or **Tra-7**, 300 µg) in PBS buffer (50 mM, pH 7.4, 120 µL) was incubated with **DBCO-MMAE** (20 e.q. in 30 uL DMSO, from BROADPHARM, purity: 98%) at 28 °C for 16 h. The reaction mixture was purified using protein A resin to afford corresponding gsADCs: **Tra-5-MMAE** (208 µg) and **Tra-7-MMAE** (210 µg) as quantified by NanoDrop, respectively. ESI-MS analysis confirmed the drug-to-antibody ratio (DAR ≈ 2.0).

Synthesis of IgG-7 and Ctx-7

Human IgG1 isotype control (A2051) was purchased from Selleckchem. Cetuximab (Erbitux®, NDC 66733-948-23) was obtained from Eli Lilly and Company Branchburg, NJ, 08876,USA.

The commercially obtained antibody solution was concentrated (35 mg/mL for IgG, 29.7 mg/mL for Ctx) using a 50 kDa MWCO centrifugal filter. The concentrated antibody was subjected to the conjugation procedure described for **Tra-7**, yielding corresponding azide modified trastuzumab: **IgG** (338 µg) and **Ctx-7** (365 µg) as quantified by NanoDrop, respectively.

Synthesis of gsDACs Tra-7-ARV and IgG-7-ARV

A solution of azide-modified trastuzumab (**Tra-7** or **IgG-7**, 300 µg) in PBS buffer (50 mM, pH 7.4, 50 µL) and 1,2-propanediol (44 µL) was incubated with **ARV771-DBCO** (20 e.q. in 6 uL DMSO) at 28 °C for 5 h. The reaction mixture was diluted with 900 µL PBS and filtered through a 0.22 µm filter to remove most of the hydrophobic payload and the filtrate was purified using protein A resin to afford corresponding gsDACs: **Tra-7-ARV** (232 µg) and **IgG-7-ARV** (231 µg) as quantified by NanoDrop, respectively. ESI-MS analysis confirmed the drug-to-antibody ratio (DAR ≈ 2.0).

Size-exclusion Chromatography (SEC) Analysis of Antibody Conjugates

Antibody samples in PBS were analyzed on an Agilent 1200 series HPLC equipped with a PolyHYDROXYETHYL A™ Colum, 200x4.0-mm; 2 µm, 1000 Å with a gradient of 100% mobile phase (50 mmol/L Sodium phosphate, pH 7.0, with 10% ACN) in 22 min.

Hydrophobic Interaction Chromatography (HIC) Analysis of Antibody Conjugates

Antibody samples in PBS were analyzed on an Agilent 1200 series HPLC equipped with a PolyPROPYL A™ Column, 100x4.6-mm; 3 µm, 1500 Å with a linear with a linear gradient of 100% mobile phase A

(1.4 mol/L $(NH_4)_2SO_4$ in 100 mmol/L phosphate buffer, pH 7.0) to 100% mobile phase B (100 mmol/L phosphate buffer, pH 7.0, with 5% *i*-PrOH) in 22 min.

Stability analysis of gsADCs

ADC samples 1 mg/mL in 1 PBS was heated at 60 ℃ for 36 h. Aliquots were taken at time point 0, 12, 24, and 36 h, and were analyzed by SEC. ADC samples were first formulated at a concentration of 1 mg/mL in 1× PBS. The solutions were then incubated at 60 °C for 36 h. At predetermined time points (0, 12, 24, and 36 h), aliquots of each sample were withdrawn for analysis. All collected samples were cooled to room temperature and subjected to SEC analysis to monitor over time.

Cell culture

MDA-MB-231 (RRID: CVCL_0062), MDA-MB-361 (RRID: CVCL_0620), SK-BR-3 (RRID: CVCL_0033), and MCF-7 (RRID: CVCL_0031) cells were cultured in high-glucose DMEM medium supplemented with 10% fetal bovine serum and 1% Penicillin/Streptomycin. BT474 (RRID: CVCL_VL01) cells were cultured in RPMI supplemented with 10% fetal bovine serum, 1% Penicillin/Streptomycin, 1% Sodium Pyruvate and 1% HEPES. All cell lines were grown at 37°C in a humidified 5% CO_2 atmosphere.

Cell Viability Assay

Human breast cancer cell lines MDA-MB-231, MDA-MB-361, and SK-BR-3 were seeded in 96-well plates at a density of 1 × 10^4 cells per well and incubated overnight at 37 °C. Cells were then treated with a three-fold serial dilution of trastuzumab or antibody–drug conjugate (ADC) samples (from 5000 to 0.085 ng/mL). After 72-hour treatment, Alamar Blue reagent (10% v/v) was added to each well and incubated for 2–4 hours at 37 °C. Finally, fluorescence intensity of each well was measured (excitation: 530–560 nm; emission: 590 nm) using a microplate reader, and Cell viability curves were generated using GraphPad Prism.

BRD4 degradation

BT474 and MCF7 cells were seeded in 24-well plate at a density of 1.2*10^5 cells pre well and incubated overnight at 37 °C. Cells were then treated with indicated concentration of Trastuzumab, **Tra-7-ARV**, or **ARV-771** for 24h. After washing with PBS for three times, cells were incubated with lysis buffer (25 mM Tris, pH 7−8, 150 mM NaCl, 0.1% (w/v) sodium dodecyl sulfate (SDS), 0.5% sodium deoxycholate, 1% (v/v) Triton X-100) and protease inhibitor on ice for 1h, followed by centrifugation at 16000g for 15min. Protein concentrations in the supernatant was determined by BCA assay. The total volume and protein amount of samples were normalized based on the results from BCA assay. After adding 4x loading Laemmli loading buffer, samples were boiled at 99 °C for 5min. Boiled samples were then separated by 7.5% SDS-polyacrylamide gel electrophoresis and transferred onto PVDF membranes. After blocking with 5% (w/v) milk in TBST, the membranes were incubated with the anti-BRD4 primary antibody (CST, 13440S) overnight at 4 °C. After three washes with TBST, membranes were incubated with HRP conjugated anti-rabbit secondary antibody at RT for 45 min. After three washes with TBST, the membranes were incubated with clarity ECL substrate and imaged by ChemiDoc MP imaging system. Band intensities were quantified using ImageJ, and data were analyzed and graphed with GraphPad Prism.

In Vivo Efficacy Evaluation

All animal procedures were approved by the Institutional Animal Care and Use Committee (IACUC) at the University of Wisconsin–Madison (Protocol #: M006790-A02). Immunodeficient NSG mice (J:NU 005557) were obtained from The Jackson Laboratory and bred in the UW–Madison Mouse Breeding and Research Services Core. Her2-positive MDA-MB-361 breast cancer cells were subcutaneously inoculated into the flanks of female mice aged 12 to 14 weeks.

When tumors reached an approximate volume of 100 mm^3, mice were randomized into three treatment cohorts: vehicle, **Tra-5-MMAE**, and **Tra-7-MMAE**. Test compounds were administered via intraperitoneal injection at a dose of 3 mg/kg daily for three consecutive days. Tumor volumes were measured twice weekly using digital calipers and calculated using the formula: Tumor volume = (width × width × length) / 2. Tumor growth inhibition was assessed by comparing tumor volume progression across treatment groups. Mice were monitored throughout the study for changes in body weight and overall health to evaluate treatment tolerability.

In Vivo Toxicity Evaluation

Wild-type Female C57BL/6 mice (6–8 weeks old, n = 3 per group) were injected with PBS, **Tra-7-MMAE** (3 mg/kg), or **Tra-5-MMAE** (3 mg/kg) via intraperitoneal injection once daily for three consecutive days. Peripheral blood was collected via the retro-orbital plexus for hematology (at day 4, 7 and 10 post-treatment) and serum chemistry analyses (at day 10 post-treatment) using a VetScan HM5 hematology analyzer (Abaxis, Union City, CA, USA) and a VetScan VS2 chemistry analyzer (Abaxis, Union City, CA, USA). Major organs (liver, spleen, lung, kidney, intestine, and heart) were harvested on day 10 post-treatment, fixed, paraffin-embedded, sectioned, and stained with H&E for histopathological evaluation. All procedures followed institutional IACUC guidelines.

Synthesis of oxazoline substrates 1-14

Unless otherwise stated, all commercial reagents were used as received. Unless stated otherwise, reactions were performed at room temperature (rt). Thin-layer chromatography (TLC) was conducted on plates (EMD Chemical Inc. 60, F254). Flash column chromatography was performed with silica gel (Silicycle, 40-63 μm). ^{1}H and ^{13}C nuclear magnetic resonance spectra (NMR) were obtained on Brooker 400 MHz and 500MHz. Chemical shifts were reported in parts per million (ppm), Coupling constants (J) were reported in Hertz (Hz). All high resolution mass spectra were performed by Analytical Instrument Center at the School of Pharmacy (UW-Madison) on an Electrospray Injection (ESI) mass spectrometer.

General method for amide bond synthesis: To a solution of the amine (1.0 e.q.), the carboxylic acid (1.3 e.q.) and DIEA (2.0 e.q.) in DMF (0.1 M) was added HATU (1.5 e.q.). The reaction was stirred at RT until LCMS indicated the end. The result mixture was purified by flash column to afford amide product. (DIEA: N,N-diisopropylethylamine, HATU: 1-[Bis(dimethylamino)methylene]-1*H*-1,2,3-triazolo[4,5-*b*]pyridinium 3-oxid hexafluorophosphate).

General method for oxazoline synthesis: To a solution of 1-OH GlcNAc containing starting material (1.0 e.q.) and K_3PO_4 (30 e.q.) in D_2O (100 V to sugar) was added DMC (20 e.q.) at 0 ℃. The reaction mixture was allowed to warm to room temperature and stirred overnight. The reaction was monitored by proton NMR. The resulted mixture was purified by preparative HPLC C18 reverse phase column eluting with acetonitrile/water (0.03% NH_3) to afford oxazoline product. DMC: 2-chloro-1,3-dimethylimidazolinium chloride.

General method for acetal/ketal LacNAc synthesis: A solution of LacNAc (1.0 e.q.) and corresponding aldehyde or dimethyl acetal or dimethyl ketal (1.2 e.q.) in DMSO (0.2 M) was co-distilled with toluene twice at 40 ℃ to remove residual water. TsOH-H_2O (0.3 e.q.) was then added, followed by an additional co-distillation with toluene under the same conditions. The mixture was subsequently continue rotated at 70 ℃ under around 10 mbar for 2-3 h. The reaction was monitored by TLC with MeOH/DCM = 1/3. Additional aldehyde or dimethyl acetal or dimethyl ketal could be added if necessary to drive the reaction to the end. The resulting mixture was purified by preparative HPLC C18 reverse phase column eluting with acetonitrile/water (0.1% formic acid) to afford the desired acetal- or ketal-modified LacNAc product.

General method for click chemistry: To a solution of azido compound (2.5 e.q.), ligand tris[(1-benzyl-1H-1,2,3-triazol-4-yl)methyl]amine (0.5 e.q.) and copper(II) sulfate (0.5 e.q.) in DMF/water (10/1, 100 V) was added sodium ascorbate (1.0 e.q.) under protection of nitrogen. Then, alkyne compound (1.0 e.q.) in DMF (50 V) was added in portions (0.2 e.q./10 min). The reaction was stirred at room temperature. The reaction mixture was purified by preparative HPLC C18 reverse phase column eluting with acetonitrile/water (0.1% formic acid).

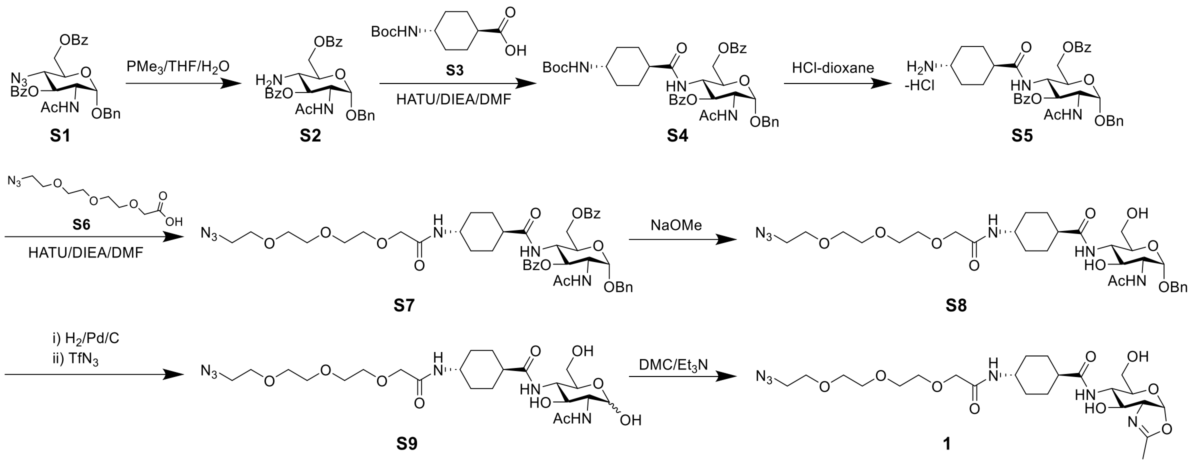

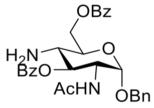

S2

To a solution of **S1**[15] (39 mg, 0.072 mmol) in THF/H_2O (10/1, 2.2 mL) was added PMe_3 (143 μL, 1 M in THF, 0.143 mmol). The mixture was stirred for 3 h and then concentrated to dryness. The residue was dried under vacuum and used directly in the next step.

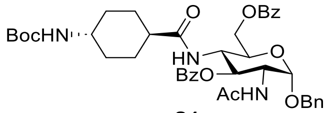

S4

S4 (22 mg, yield 40.0%) was synthesized from **S2** (38 mg, 0.074 mmol) and **S3** (26.6 mg, 0.11 mmol) according to the "*General method for amide bond synthesis*". ^{1}H NMR (400 MHz, $CDCl_3$) δ 8.12 – 8.06 (m, 2H), 8.01 – 7.94 (m, 2H), 7.61 – 7.53 (m, 2H), 7.49 – 7.39 (m, 4H), 7.37 – 7.31 (m, 3H), 7.29 – 7.24 (m, 5H), 5.66 (d, *J* = 9.7 Hz, 1H), 5.61 (d, *J* = 9.1 Hz, 1H), 5.35 (t, *J* = 10.5 Hz, 1H), 5.02 (d, *J* = 3.7 Hz, 1H), 4.71 (d, *J* = 11.6 Hz, 1H), 4.59 (ddd, *J* = 17.6, 11.1, 2.9 Hz, 2H), 4.49 (d, *J* = 11.6 Hz, 1H), 4.44 – 4.32 (m, 2H), 4.17 – 4.05 (m, 1H), 3.29 (s, 1H), 2.04 – 1.84 (m, 3H), 1.76 (dd, *J* = 14.5, 2.1 Hz, 1H), 1.64 (d, *J* = 19.6 Hz, 2H), 1.41 (s, 10H), 1.30 – 1.21 (m, 1H), 1.05 – 0.89 (m, 2H).

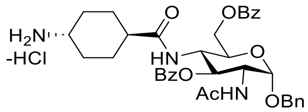

S5

To a solution of **S4** (24 mg, 0.032 mmol) in dioxane (1 mL) was added HCl-dioxane (4 M, 1 mL). The mixture was stirred for 3 h and then concentrated to dryness. The residue was dried under vacuum and used directly in the next step.

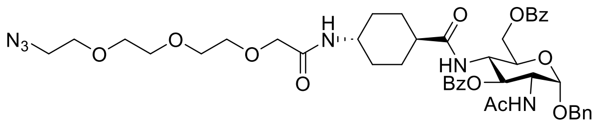

S7

S7 (12.2 mg, yield 40.0%) was synthesized from **S5** (crude, ~0.032 mmol) and **S6** (16 mg, 0.048 mmol) according to the "*General method for amide bond synthesis*". ^{1}H NMR (400 MHz, $CDCl_3$) δ 8.17 – 8.08 (m, 2H), 8.05 – 7.94 (m, 2H), 7.66 – 7.54 (m, 2H), 7.54 – 7.41 (m, 4H), 7.40 – 7.33 (m, 3H), 7.31 – 7.26 (m, 3H), 6.69 (d, *J* = 8.3 Hz, 1H), 5.72 (dd, *J* = 9.4, 4.2 Hz, 2H), 5.38 (t, *J* = 10.5 Hz, 1H, H-3), 5.04 (d, *J* = 3.6 Hz, 1H, H-1), 4.73 (d, *J* = 11.6 Hz, 1H), 4.66 – 4.56 (m, 2H, H-2 and H-6a), 4.52 (d, *J* = 11.6 Hz, 1H), 4.47 – 4.34 (m, 2H, H-4 and H-6b), 4.14 (ddd, *J* = 10.7, 6.2, 2.1 Hz, 1H, H-5), 3.95 (s, 2H), 3.78 – 3.60 (m, 10H), 3.44 – 3.35 (m, 2H), 2.01 – 1.88 (m, 3H), 1.88 – 1.74 (m, 4H), 1.65 (dt, *J* = 12.9, 3.2 Hz, 1H), 1.39 (dqd, *J* = 42.5, 13.1, 3.4 Hz, 2H), 1.19 – 1.03 (m, 2H). ^{13}C NMR (100 MHz, $CDCl_3$) δ 175.2, 169.8, 169.0, 167.9, 166.4, 136.5, 133.8, 133.2, 130.0, 129.9, 129.7, 128.7, 128.7, 128.7, 128.5, 128.4, 128.3, 96.6, 71.9, 70.9, 70.7, 70.6, 70.4, 70.3, 70.2, 70.1, 69.9, 63.9, 51.6, 50.7, 50.7, 47.0, 44.6, 32.1, 31.9, 28.1, 27.9, 23.2.

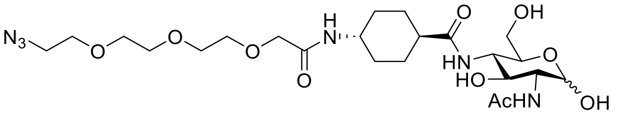

S9

To a solution of **S7** (12 mg, 0.014 mmol) in methanol (1 mL) was added NaOMe (30% in methanol) to adjust pH to around 11. The mixture was stirred for 7 h and then neutralized with HCl (1 M). The residue of **S8** was concentrated to dryness and used directly in the next step. To a solution of crude **S8** in *t*-BuOH/H_2O (10/1, 3.3 mL) was added Pd/C (5%, 5 mg) with the protection of nitrogen. The reaction mixture was degassed with hydrogen balloon and stirred for an additional 3 h. The crude product was afforded after filtration through celite. The conversion of the amine to azide was according to the reported procedure[16]. The reaction mixture was purified by preparative HPLC C18 reverse phase column eluting with acetonitrile/water (0.1% formic acid) to afford **S9** (4.3 mg, 54.8% over three steps). ^{1}H NMR (400 MHz, D_2O) δ 5.17 (d, *J* = 3.4 Hz, 0.56H), 4.65 (d, *J* = 7.8 Hz, 0.44H), 3.98 (s, 2H), 3.90 – 3.81 (m, 1H), 3.81 – 3.73 (m, 1H), 3.73 – 3.56 (m, 13H), 3.56 – 3.51 (m, 1H), 3.51 – 3.40 (m, 2H), 2.21 (t, *J* = 12.2 Hz, 1H), 1.97 (d, *J* = 1.0 Hz, 3H), 1.87 (t, *J* = 15.1 Hz, 4H), 1.47 (q, *J* = 13.0 Hz, 2H), 1.29 (q, *J* = 12.3 Hz, 2H).

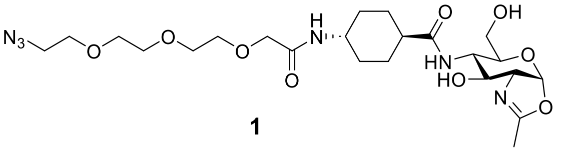

1

To a solution of **S9** (4.3 mg, 0.0077 mmol) and Et_3N (21.2 μL, 0.154 mmol) in D_2O (500 μL) was added DMC (13.0 mg, 0.076 mmol) at 0 ℃. The reaction mixture was allowed to warm to room temperature and stirred overnight. The reaction was monitored by proton NMR. The resulted mixture was purified G10 column to afford **1** (3.5 mg, yield 83.9%). ^{1}H NMR (400 MHz, D_2O) δ 6.05 (d, *J* = 7.3 Hz, 1H), 4.12 – 4.06 (m, 1H), 3.98 (s, 2H), 3.92 (t, *J* = 3.3 Hz, 1H), 3.80 – 3.75 (m, 1H), 3.70 – 3.62 (m, 12H), 3.61-3.55 (m, 2H), 3.45-3.40 (m, 2H), 2.24 – 2.13 (m, 1H), 2.02 (d, *J* = 1.7 Hz, 3H), 1.94 – 1.74 (m, 5H), 1.51 – 1.39 (m, 2H), 1.31 – 1.28 (m, 1H). ^{13}C NMR (125 MHz, D_2O) δ 179.0, 171.4, 168.8, 100.2, 70.6, 70.1, 69.6, 69.5, 69.4, 69.2, 65.3, 61.9, 50.1, 49.5, 47.9, 43.4, 30.7, 30.7, 27.8, 27.6, 13.1. HRMS (ESI) for $C_{23}H_{39}N_6O_9$ ($[M+H^+]$): calcd 543.2778, found 543.2763.

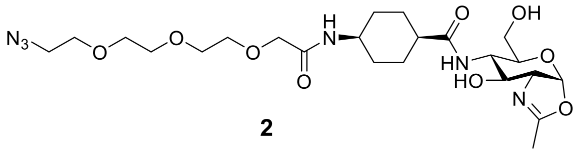

2

Oxazoline **2** was synthesized analogously to **1**, except for the final step, which was according to the "*General method for oxazoline synthesis*". ^{1}H NMR (400 MHz, D_2O) δ 6.05 (d, *J* = 7.3 Hz, 1H), 4.12 – 4.06 (m, 1H), 4.01 (s, 2H), 3.95 – 3.90 (m, 1H), 3.90 – 3.83 (m, 1H), 3.79 (ddd, *J* = 9.5, 2.8, 1.2 Hz, 1H), 3.72 – 3.62 (m, 10H), 3.59 (dd, *J* = 12.4, 2.6 Hz, 1H), 3.52 (dd, *J* = 12.3, 6.4 Hz, 1H), 3.48 – 3.40 (m, 3H), 2.39 – 2.27 (m, 1H), 2.01 (d, *J* = 1.7 Hz, 3H), 1.72 – 1.55 (m, 8H). ^{13}C NMR (125 MHz, D_2O) δ 178.8, 171.8, 169.0, 100.4, 71.0, 70.4, 69.8, 69.7, 69.7, 69.4, 65.5, 62.1, 50.3, 49.7, 45.6, 41.9, 28.2, 24.4, 24.2, 13.3. HRMS (ESI) for $C_{23}H_{39}N_6O_9$ ([M+H$^+$]): calcd 543.2778, found 543.2769.

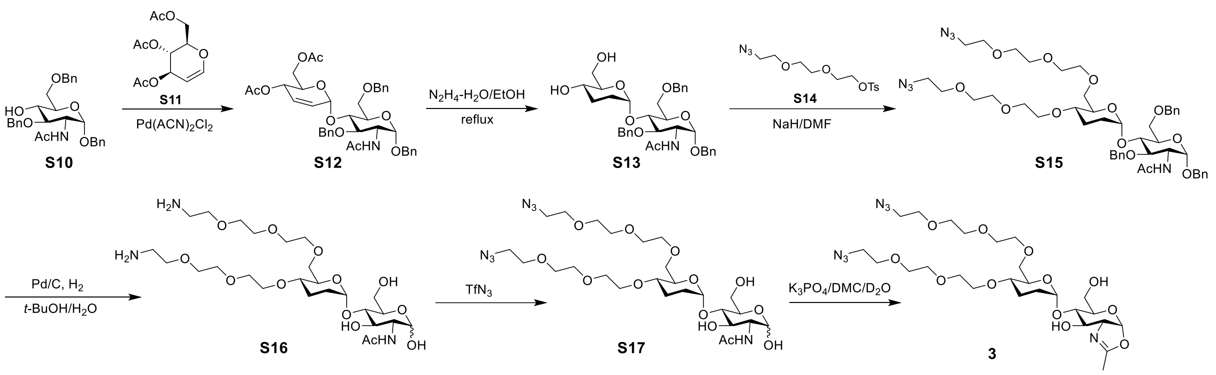

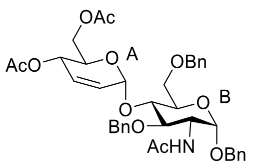

S12

The synthesis was initially inspired by reported work[17]. To a solution of **S10**[18] (300 mg, 0.61 mmol) and **S11** (195.6 mg, 0.73 mmol) in DCM/DCE (1/1, 8 mL) was added Pd(ACN)$_2$Cl$_2$ (47 mg, 0.18 mmol). The reaction mixture was stirred at 50 ℃ for 5 h. LCMS indicated the end reaction. The reaction mixture was purified by preparative HPLC C18 reverse phase column eluting with acetonitrile/water (0.1% formic acid) to afford **S12** (182 mg, 27.5%). ^{1}H NMR (400 MHz, $CDCl_3$) δ 7.45 – 7.29 (m, 15H), 5.84 (dt, *J* = 10.2, 1.5 Hz, 1H), 5.64 (ddd, *J* = 10.2, 2.8, 2.1 Hz, 1H), 5.46 – 5.36 (m, 2H, H-1^A and AcN*H*), 5.24 (dq, *J* = 9.7, 1.8 Hz, 1H), 4.91 (d, *J* = 3.6 Hz, 1H, H-1^B), 4.80 (d, *J* = 11.6 Hz, 1H), 4.72 (d, *J* = 12.3 Hz, 1H), 4.67 (s, 2H), 4.60 (d, *J* = 12.3 Hz, 1H), 4.49 (d, *J* = 11.7 Hz, 1H), 4.34 (td, *J* = 9.7, 4.0 Hz, 1H, H-2^A), 4.09 (dd, *J* = 12.2, 4.7 Hz, 1H), 4.02 – 3.93 (m, 2H), 3.87 – 3.69 (m, 5H, H-3^A), 2.11 (s, 3H), 2.07 (s, 3H), 1.83 (s, 3H). ^{13}C NMR (100 MHz, $CDCl_3$) δ 170.7, 170.1, 169.6, 138.3, 137.9, 137.1, 129.2, 128.6, 128.6, 128.4, 128.2, 128.0, 127.9, 127.6, 127.5, 127.3, 96.9, 95.6, 81.3, 76.6, 74.9, 73.2, 70.8, 69.5, 69.4, 67.2, 64.9, 62.6, 52.4, 23.4, 21.0, 20.8.

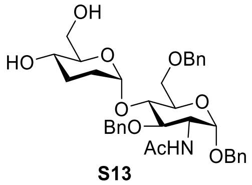

S13

To a solution of **S12** (180 mg, 0.25 mmol) in EtOH (8 mL) was added N_2H_4-H_2O (0.8 mL). The reaction mixture was refluxed at 85 ℃ overnight. The resulting mixture was separated by ethyl acetate and water. The organic layer was further washed with water three times and then concentrated to dryness. The residue was dried under vacuum to afford 158 mg foam solid and used directly in the next step.

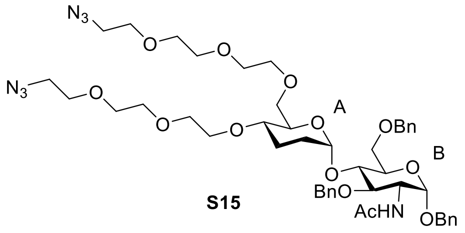

S15

To a solution of **S13** (64 mg, 0.1 mmol) and **S14** (100 mg, 0.3 mmol) in DMF (1 mL) was added NaH (12 mg). The reaction mixture was room temperature overnight. The reaction mixture was purified by preparative HPLC C18 reverse phase column eluting with acetonitrile/water (0.1% formic acid) to afford **S15** (30 mg, 21.2%). ^{1}H NMR (400 MHz, Acetone-d$_6$) δ 7.47 – 7.20 (m, 16H, Ar-*H* and AcN*H*), 5.26 (dd, *J* = 1.4, 3.7 Hz, 1H, H-1^A), 4.91 – 4.84 (m, 2H, H-1^B), 4.80 (d, *J* = 11.9 Hz, 1H), 4.72 – 4.59 (m, 3H), 4.54 (d, *J* = 11.9 Hz, 1H), 4.38 – 4.27 (m, 1H, H-2^B), 3.98 – 3.75 (m, 6H, H-3^B), 3.74 – 3.65 (m, 3H), 3.65 – 3.51 (m, 10H), 3.51 – 3.42 (m, 1H), 3.38 (t, *J* = 5.0 Hz, 2H), 1.91 (s, 3H), 1.84 – 1.65 (m, 3H, H-2a^A), 1.58 (tt, *J* = 13.3, 4.2 Hz, 1H, H-2b^A). ^{13}C NMR (100 MHz, Acetone-d$_6$) δ 169.1, 139.3, 138.9, 137.9, 128.3, 128.2, 128.0, 127.6, 127.6, 127.3, 127.2, 97.3, 97.0, 81.2, 75.1, 73.8, 73.6, 72.8, 71.8, 71.2, 70.8, 70.4, 70.3, 69.9, 69.8, 68.9, 66.6, 52.6, 50.5, 26.9, 22.1.

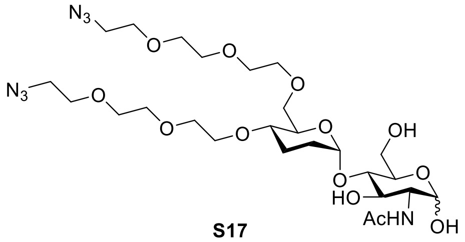

S17

S17 (4.2 mg, yield 23.4% over two steps) was synthesized analogously to **S9**. ^{1}H NMR (400 MHz, D$_2$O) δ 5.28 (s, 1H), 5.12 (d, *J* = 2.8 Hz, 0.62H), 3.89 – 3.57 (m, 34H), 3.47 – 3.40 (m, 5H), 2.06 – 1.94 (m, 4H), 1.88 (d, *J* = 15.2 Hz, 1H), 1.73 (t, *J* = 13.9 Hz, 1H), 1.56 (d, *J* = 13.2 Hz, 1H).

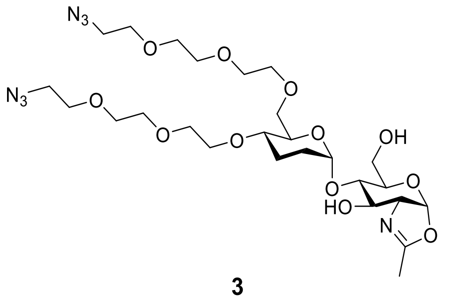

3

Oxazoline **3** (2.4 mg, yield 61.8%) was synthesized according to the "*General method for oxazoline synthesis*". ^{1}H NMR (500 MHz, D$_2$O) δ 6.01 (d, *J* = 7.2 Hz, 1H), 4.98 (d, *J* = 3.3 Hz, 3H), 4.16 – 4.09 (m, 2H), 3.76 – 3.68 (m, 3H), 3.66 – 3.52 (m, 27H), 3.43 – 3.31 (m, 6H), 2.01 – 1.91 (m, 4H), 1.87 – 1.79 (m, 1H), 1.79 – 1.73 (m, 1H), 1.73 – 1.64 (m, 1H), 1.58 – 1.46 (m, 1H). ^{13}C NMR (125 MHz, D$_2$O) δ

168.0, 99.8, 94.7, 73.6, 73.1, 72.0, 71.2, 70.3, 70.1, 69.8, 69.8, 69.7, 69.4, 68.2, 66.9, 65.2, 62.5, 50.3, 47.8, 28.4, 23.3, 13.0. HRMS (ESI) for $C_{26}H_{46}N_7O_{12}$ ($[M+H^+]$): calcd 648.3204, found 648.3191.

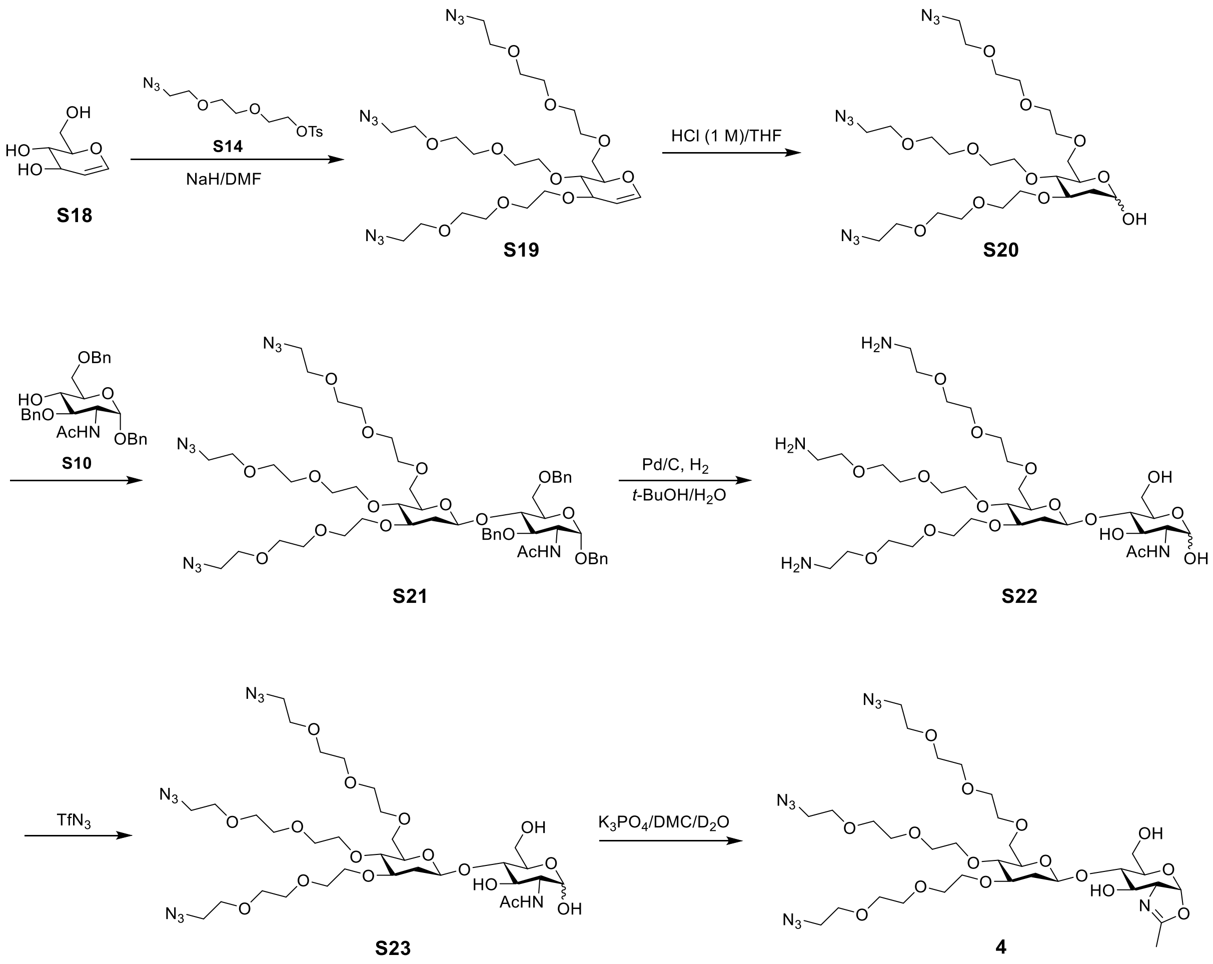

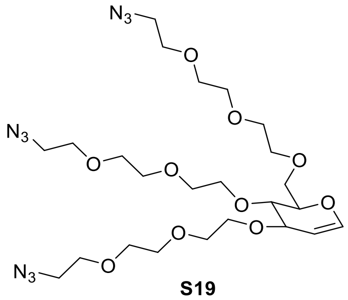

S19

S19 (305 mg, yield 54.4%) was synthesized analogously to **S15**. ^{1}H NMR (400 MHz, $CDCl_3$) δ 6.37 (dd, *J* = 6.1, 1.4 Hz, 1H), 4.82 (dd, *J* = 6.2, 2.6 Hz, 1H), 4.08 (dt, *J* = 6.5, 2.0 Hz, 1H), 4.02 – 3.93 (m, 2H), 3.86 – 3.60 (m, 40H), 3.41 (t, *J* = 5.1 Hz, 6H).

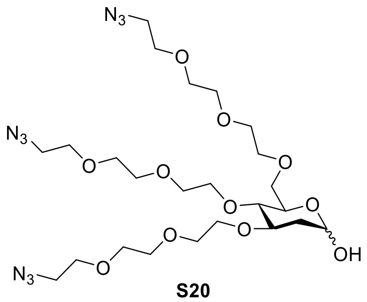

S20

To a solution of **S19** (100 mg, 0.162 mmol) in THF (1 mL) was added HCl (1 M, 1 mL). The reaction mixture was stirred at room temperature for 2 h. The resulting mixture was quenched with an aqueous solution of sodium bicarbonate and separated by ethyl acetate and water. The aqueous layer was extracted with ethyl acetate three times. The combined organic layers were dried, filtered, and concentrated under reduced pressure. The crude product was purified by flash column chromatography using DCM/MeOH as the eluents to afford **S20** (75 mg, 72.9%).

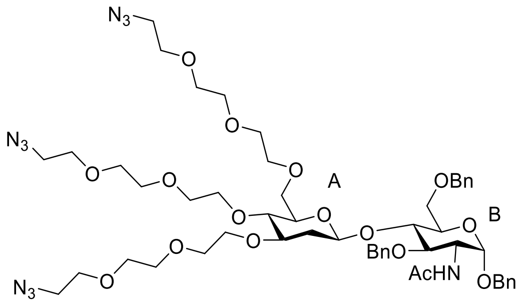

S21

The synthesis of **S21** was according to reported literature[19]. A solution of **S20** (37 mg, 0.058 mmol) and 2,4,6-tri-tert-butylpyrimidine (TTBP, 14 mg, 0.058 mmol) in 1 mL THF was cooled to -78 °C and treated dropwise with potassium hexamethyldisilazane (1 M in THF, 58 µL, 0.058 mmol). After 15 min, a solution of p-toluenesulfonic anhydride (19 mg, 0.058 mmol) in 0.5 mL THF was added rapidly to the reaction. The solution was maintained at −78 °C for 30 min. Meanwhile **S10** (19 mg, 0.038 mmol) was dissolved in 1 mL THF, cooled to −78 °C, and treated with potassium hexamethyldisilazane (1 M in THF, 76 µL, 0.076 mmol). After 15 min, this solution was transferred dropwise by syringe to the primary reaction vessel. The reaction mixture was then allowed to gradually warm to room temperature over the course of 3 h, and stirred for an additional 15 h. The reaction was quenched with several drops of saturated, aqueous ammonium chloride (NH_4Cl), diluted with water, and extracted with DCM (2 × 10 mL). The pooled organic phase was washed with brine (2 × 10 mL) and then dried (Na_2SO_4), filtered, and concentrated under reduced pressure. The crude product was purified by silica gel flash column chromatography using DCM/MeOH/EA/hexane as the eluents to afford the product (9.3 mg, yield 14.4%) as a single β-anomer. ^{1}H NMR (400 MHz, $CDCl_3$) δ 7.32 – 7.15 (m, 15H), 5.16 (d, *J* = 9.1 Hz, 1H, AcN*H*), 4.90 – 4.82 (m, 2H, H-1^B), 4.60 (d, *J* = 11.9 Hz, 2H), 4.52 (d, *J* = 12.2 Hz, 1H), 4.48 (dd, *J* = 9.8, 1.9 Hz, 1H, H-1^A)), 4.44 (d, *J* = 12.1 Hz, 1H), 4.36 (d, *J* = 11.9 Hz, 1H), 4.13 (ddd, *J* = 10.6, 9.1, 3.8 Hz, 1H, H-2^B), 3.95 – 3.82 (m, 2H), 3.74 – 3.43 (m, 40H, H-3^B), 3.39 (t, *J* = 5.1 Hz, 3H), 3.29 (dt, *J* = 11.9, 5.1 Hz, 7H), 3.22 – 3.07 (m, 4H, H-3^A), 2.14 – 2.05 (m, 1H, H-2a^A), 1.69 (s, 3H), 1.36 (q, *J* = 11.4 Hz, 1H, H-2b^A). ^{13}C NMR (100 MHz, $CDCl_3$) δ 169.7, 139.2, 137.9, 137.3, 128.5, 128.5, 128.3, 128.0, 128.0, 127.9, 127.8, 127.6, 127.5, 100.0, 97.1, 79.9, 78.8, 78.4, 76.5, 75.4, 74.1, 73.5, 71.9, 70.9, 70.9, 70.7, 70.6,

70.6, 70.5, 70.1, 69.9, 69.7, 69.1, 68.4, 52.3, 50.7, 36.9, 23.3.

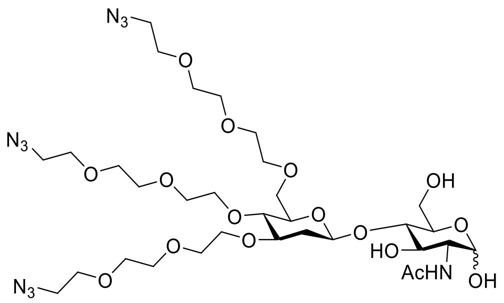

S23

S23 (5.5 mg, crude) was synthesized analogously to **S9**. ^{1}H NMR (400 MHz, D_2O) δ 5.14 (d, *J* = 2.8 Hz, 0.51H), 4.50 (d, *J* = 11.9 Hz, 0.49H), 3.95 (d, *J* = 11.5 Hz, 1H), 3.89 – 3.53 (m, 49H), 3.45 (t, *J* = 4.6 Hz, 8H), 3.30 (q, *J* = 9.1 Hz, 1H), 3.14 (s, 1H), 2.53 – 2.36 (m, 1H), 2.05 – 1.94 (m, 3H), 1.46 (d, *J* = 12.0 Hz, 1H).

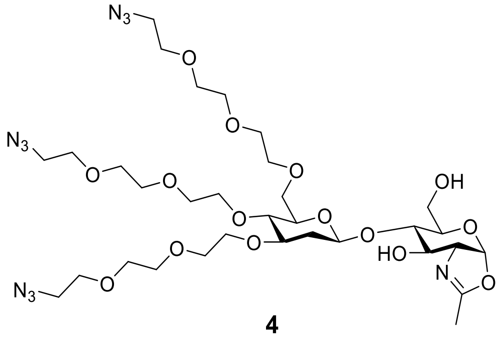

4

Oxazoline **4** (3.1 mg, yield 61.8%) was synthesized according to the "*General method for oxazoline synthesis*". ^{1}H NMR (400 MHz, D_2O) δ 6.01 (d, *J* = 7.3 Hz, 1H), 4.27 (dd, *J* = 3.2, 1.8 Hz, 1H), 4.14 – 4.06 (m, 1H), 3.94 (dt, *J* = 10.9, 4.2 Hz, 1H), 3.83 – 3.52 (m, 41H), 3.48 – 3.39 (m, 7H), 3.30 (ddd, *J* = 8.8, 6.2, 2.5 Hz, 1H), 3.24 (t, *J* = 9.4 Hz, 1H), 2.35 (dd, *J* = 12.2, 4.7 Hz, 1H), 1.98 (d, *J* = 1.9 Hz, 3H), 1.41 (q, *J* = 11.5 Hz, 1H). ^{13}C NMR (125 MHz, D_2O) δ 171.2, 101.5, 100.0, 79.2, 78.2, 78.0, 74.2, 71.7, 71.0, 70.3, 70.2, 70.2, 69.8, 69.8, 69.7, 69.7, 69.4, 68.9, 65.4, 61.8, 50.3, 36.3, 13.1. HRMS (ESI) for $C_{32}H_{57}N_{10}O_{15}$ ([M+H^{+}]): calcd 821.4005, found 821.3993.

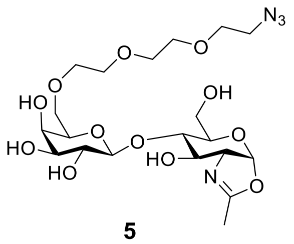

5

Oxazoline **5** was synthesized according to the reported work[16]. ^{1}H NMR (400 MHz, D_2O) δ 6.02 (d, *J* = 7.3 Hz, 1H), 4.37 – 4.31 (m, 2H), 4.12 (ddq, *J* = 7.3, 3.3, 1.5 Hz, 1H), 3.84 (dd, *J* = 3.4, 0.9 Hz, 1H), 3.79 – 3.57 (m, 18H), 3.55 (dd, *J* = 9.9, 3.4 Hz, 1H), 3.47 – 3.40 (m, 3H), 3.38 (ddd, *J* = 8.9, 6.2, 2.4 Hz, 1H), 1.99 (d, *J* = 1.8 Hz, 3H).

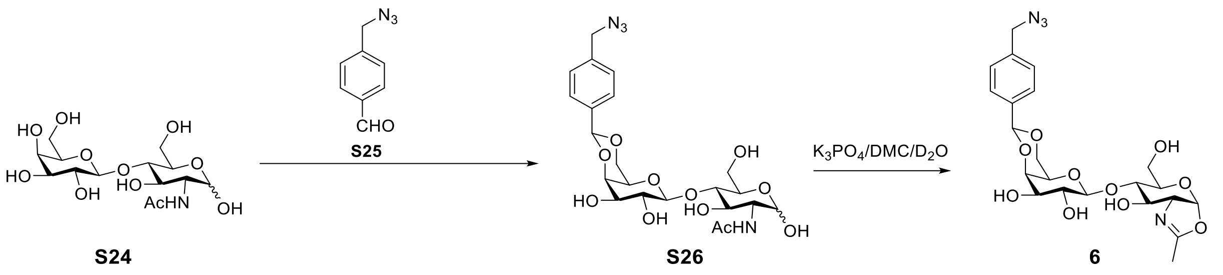

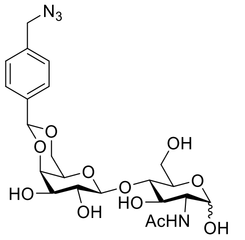

S26

S26 (14.4 mg, yield 55.4%) was synthesized according to the "*General method for acetal/ketal LacNAc synthesis*". ^{1}H NMR (400 MHz, CD_3OD) δ 7.57 (dd, *J* = 8.2, 2.7 Hz, 2H), 7.35 (dd, *J* = 8.3, 1.6 Hz, 2H), 5.64 (d, *J* = 1.5 Hz, 1H), 5.11 (d, *J* = 2.7 Hz, 0.62H), 4.62 – 4.55 (m, 0.5H), 4.54 – 4.46 (m, 1H), 4.36 (s, 2H), 4.26 – 4.12 (m, 3H), 4.00 – 3.84 (m, 3H), 3.84 – 3.75 (m, 1H), 3.72 – 3.59 (m, 5H), 1.98 (d, *J* = 2.1 Hz, 3H). ^{13}C NMR (100 MHz, CD_3OD) δ 172.7, 172.2, 138.2, 136.3, 127.7, 126.6, 103.6, 100.5, 90.9, 79.9, 76.0, 72.1, 70.5, 70.1, 69.3, 68.8, 66.9, 60.4, 54.2, 53.8, 21.2. HRMS (ESI) for $C_{22}H_{30}N_4NaO_{11}$ ([M+Na$^+$]): calcd 549.1809, found 549.1800.

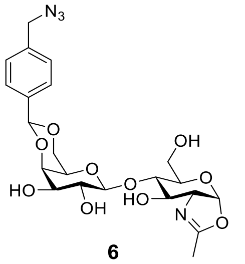

6

Oxazoline **6** (7.2 mg, yield 83.4%) was synthesized according to the "*General method for oxazoline synthesis*". ^{1}H NMR (400 MHz, D_2O) δ 7.53 (d, *J* = 8.2 Hz, 2H), 7.41 (d, *J* = 8.1 Hz, 2H), 6.04 (d, *J* = 7.3 Hz, 1H), 5.69 (s, 1H), 4.52 – 4.44 (m, 2H), 4.40 (s, 2H), 4.30 (d, *J* = 3.7 Hz, 1H), 4.26 – 4.11 (m, 3H), 3.82 – 3.70 (m, 3H), 3.70 – 3.56 (m, 3H), 3.41 (ddd, *J* = 8.8, 6.2, 2.5 Hz, 1H), 2.01 (d, *J* = 1.6 Hz, 3H). ^{13}C NMR (100 MHz, D_2O) δ 171.0, 137.1, 136.8, 128.6, 126.8, 104.6, 100.8, 99.8, 78.8, 75.7, 71.2, 70.9, 70.4, 69.0, 68.6, 66.6, 65.1, 61.7, 53.9, 12.9. HRMS (ESI) for $C_{22}H_{28}KN_4O_{10}$ ([M+K$^+$]): calcd 547.1442, found 547.1433.

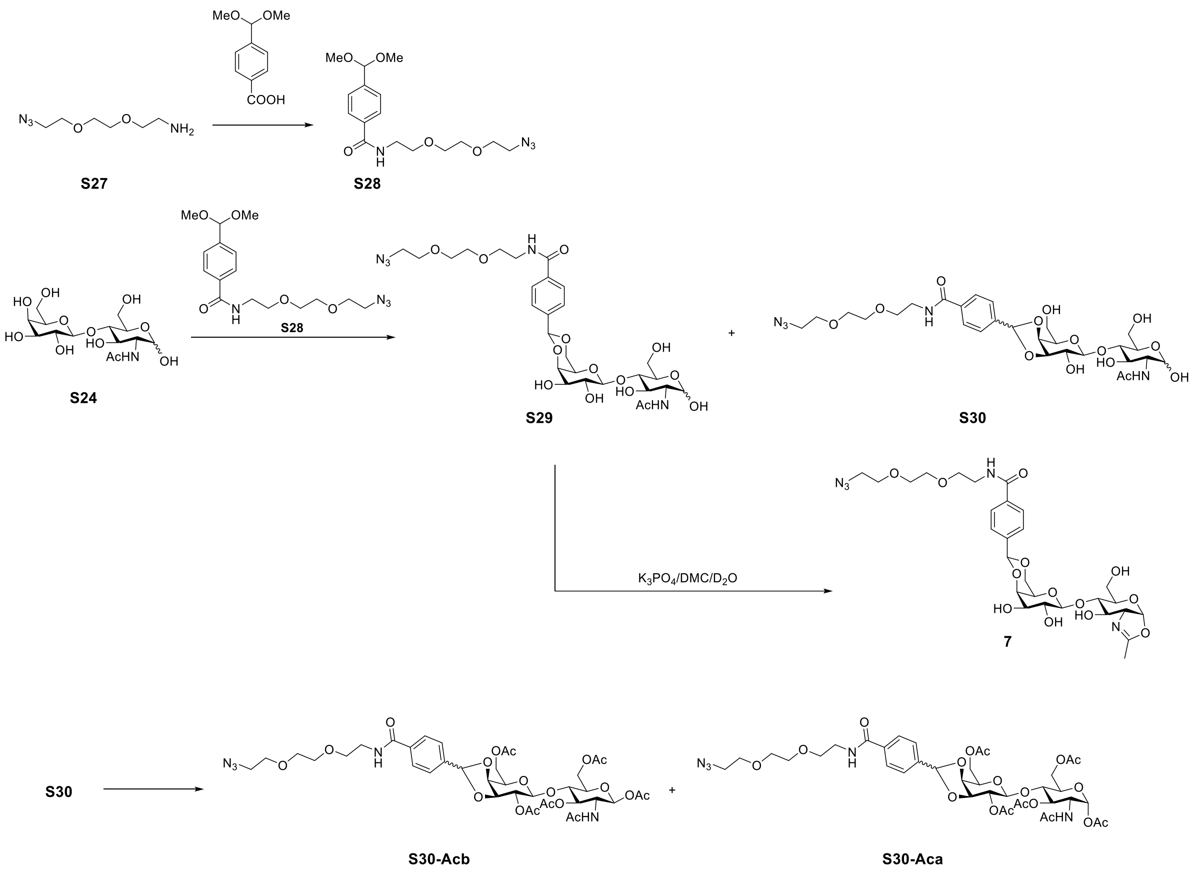

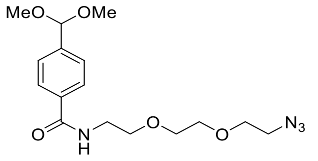

S28

To a solution of **S27** (159 mg, 0.9 mmol), 4-(dimethoxymethyl)benzoic acid (196 mg, 1 mmol), HOBt-H_2O (135 mg, 1 mmol) and triethylamine (207 µL, 1.5 mmol) in DCM (3 mL) was added EDCI (288 mg, 1.5 mmol). The reaction mixture was stirred at room temperature for 2 h. The resulting mixture was separated by ethyl acetate and water. The organic layer was further washed with water and sodium bicarbonate aqueous solution. The organic layers were dried, filtered, and concentrated under reduced pressure to afford **S28** (308 mg, 87.5%). EDCI: 1-Ethyl-3-(3-dimethylaminopropyl)carbodiimide hydrochloride. ^{1}H NMR (400 MHz, $CDCl_3$) δ 7.81 (d, *J* = 8.3 Hz, 2H), 7.55 (d, *J* = 8.3 Hz, 2H), 5.46 (s, 1H), 3.75 – 3.65 (m, 12H), 3.39 (t, *J* = 5.0 Hz, 2H), 3.35 (s, 6H). ^{13}C NMR (100 MHz, $CDCl_3$) δ 167.3, 141.5, 134.7, 127.0, 127.0, 102.5, 77.5, 77.2, 76.8, 70.6, 70.3, 70.1, 69.9, 52.7, 50.7, 39.8, 0.1.

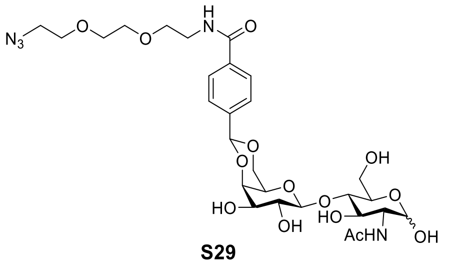

S29

S29 (20.4 mg, yield 61.0%) was synthesized according to the "*General method for acetal/ketal LacNAc*

synthesis". **S30** (6.5 mg, yield 19.4%) was collected at same reaction. ^{1}H NMR (400 MHz, D_2O) δ 7.77 – 7.71 (m, 2H), 7.63 – 7.56 (m, 2H), 5.74 (s, 1H), 5.14 (d, *J* = 3.0 Hz, 0.81H), 4.51 (d, *J* = 7.9 Hz, 1H), 4.32 (d, *J* = 3.6 Hz, 1H), 4.25 – 4.14 (m, 2H), 3.97 – 3.73 (m, 7H), 3.73 – 3.58 (m, 12H), 3.54 (t, *J* = 5.3 Hz, 2H), 3.38 – 3.32 (m, 2H), 1.96 (s, 3H). ^{13}C NMR (100 MHz, D_2O) δ 174.7, 174.4, 170.6, 140.3, 134.7, 127.3, 126.6, 103.0, 100.2, 90.5, 79.2, 75.7, 74.8, 71.2, 70.4, 70.3, 69.5, 69.5, 69.2, 69.1, 69.0, 68.8, 66.6, 53.7, 50.1, 39.6, 22.1, 21.8. HRMS (ESI) for $C_{28}H_{41}N_5NaO_{14}$ ([M+Na$^+$]): calcd 694.2548, found 694.2533.

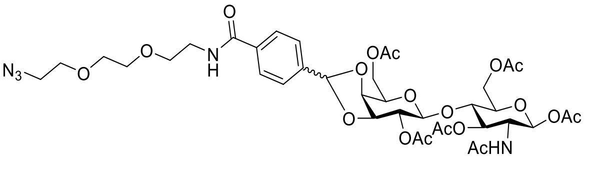

S30-Acb (epimers: S30-Acb-1 and S30-Acb-2)

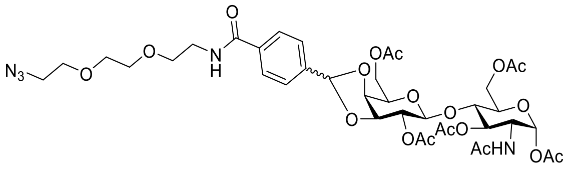

S30-Aca (epimers: S30-Aca-1 and S30-Aca-2)

The total acetylation was done as previously reported[20]. The epimers co-eluted during reverse phase purification.

S30-Acb (**S30-Acb1**/**S30-Acb2** = 0.8/1): ^{1}H NMR (400 MHz, $CDCl_3$) δ 7.90 – 7.84 (m, 1.8H), 7.82 (d, *J* = 8.2 Hz, 2H), 7.61 (d, *J* = 8.2 Hz, 1.8H), 7.50 (d, *J* = 8.2 Hz, 2H), 6.22 (s, 1H), 5.95 (s, 0.8H), 5.85 (d, *J* = 9.7 Hz, 0.8H), 5.71 (d, *J* = 9.7 Hz, 1H), 5.66 (d, *J* = 7.8 Hz, 1H, H-1^{GlcN-2}), 5.60 (d, *J* = 7.8 Hz, 0.8H, H-1^{GlcN-1}), 5.09 (dd, *J* = 9.3, 7.6 Hz, 1H, H-3^{GlcN-2}), 5.07 – 5.03 (m, 1H, H-2^{Gal-2}), 5.03 – 4.98 (m, 0.8H, H-3^{GlcN-1}), 4.90 (t, *J* = 6.2 Hz, 0.8H, H-2^{Gal-1}), 4.53 (d, *J* = 6.6 Hz, 0.8H, H-1^{Gal-1}), 4.51 – 4.49 (m, 1H), 4.49 – 4.44 (m, 2H, H-1^{Gal-2}), 4.44 – 4.17 (m, 11.5H, H-3^{Gal-1}, H-2^{GlcN-1}, and H-2^{GlcN-2}), 4.10 – 4.04 (m, 0.8H), 3.98 (ddd, *J* = 7.3, 5.4, 1.9 Hz, 1H), 3.90 – 3.77 (m, 2.8H, H-4^{GlcN-1} and H-4^{GlcN-2}), 3.75 – 3.67 (m, 20H), 3.64 (ddd, *J* = 8.2, 5.0, 3.0 Hz, 1H), 3.40 (q, *J* = 5.2 Hz, 4H), 2.18 – 2.10 (m, 26H), 2.08 (s, 3H), 1.98 (s, 3H), 1.95 (s, 2.4H). LCMS: 822.3 [M-OAc]$^+$.

S30-Aca (**S30-Aca1**/**S30-Aca2** = 0.9/1): ^{1}H NMR (400 MHz, $CDCl_3$) δ 7.90 – 7.84 (m, 1.8H), 7.84 – 7.79 (m, 2H), 7.65 – 7.59 (m, 1.8H), 7.50 (d, *J* = 8.2 Hz, 2H), 6.22 (s, 1H), 6.14 (d, *J* = 3.7 Hz, 0.9H, H-1^{GlcN-1}), 6.12 (d, *J* = 3.6 Hz, 1H, H-1^{GlcN-2}), 5.93 (s, 1H), 5.68 (d, *J* = 9.2 Hz, 0.9H), 5.61 (d, *J* = 9.1 Hz, 0.88H), 5.30 – 5.19 (m, 1.84H, H-3^{GlcN-1} and H-3^{GlcN-2}), 5.06 (t, *J* = 6.8 Hz, 1H, H-2^{Gal-2}), 4.93 (dd, *J* = 6.8, 5.8 Hz, 0.9H, H-2^{Gal-1}), 4.52 (d, *J* = 6.8 Hz, 0.9H, H-1^{Gal-1}), 4.50 – 4.46 (m, 2H, H-1^{Gal-2}), 4.45 – 4.28 (m, 10H, H-3^{Gal-1}, H-2^{GlcN-1}, and H-2^{GlcN-2}), 4.28 – 4.17 (m, 3H), 4.06 (ddd, *J* = 7.3, 5.4, 2.1 Hz, 1H), 4.00 – 3.90 (m, 1.9H), 3.89 – 3.79 (m, 3H, H-4^{GlcN-1} and H-4^{GlcN-2}), 3.75 – 3.64 (m, 20.6H), 3.39 (q, *J* = 4.8 Hz, 4H), 2.22 (s, 2.7H), 2.20 (s, 3H), 2.17 – 2.11 (m, 21.5H), 2.07 (s, 3H), 1.97 (s, 2.7H), 1.96 (s, 3H). LCMS:882.3 [M+H]$^+$.

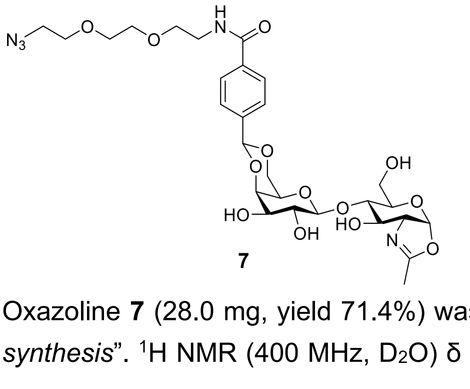

7

Oxazoline **7** (28.0 mg, yield 71.4%) was synthesized according to the "*General method for oxazoline synthesis*". ^{1}H NMR (400 MHz, D_2O) δ 7.77 – 7.71 (m, 2H), 7.64 – 7.56 (m, 2H), 6.02 (d, *J* = 7.3 Hz,

1H), 5.72 (s, 1H), 4.48 (d, *J* = 7.9 Hz, 1H), 4.45 (dd, *J* = 3.2, 1.7 Hz, 1H), 4.34 – 4.28 (m, 1H), 4.26 – 4.10 (m, 3H), 3.82 – 3.58 (m, 15H), 3.58 – 3.50 (m, 2H), 3.40 (ddd, *J* = 8.7, 6.2, 2.5 Hz, 1H), 3.34 (dd, *J* = 5.7, 4.2 Hz, 2H), 1.99 (d, *J* = 1.8 Hz, 3H). ^{13}C NMR (100 MHz, D_2O) δ 170.6, 168.3, 140.4, 134.7, 127.3, 126.6, 104.6, 100.4, 99.8, 78.7, 75.8, 71.2, 70.9, 70.4, 69.5, 69.5, 69.2, 69.1, 68.8, 68.6, 66.6, 65.0, 61.7, 50.1, 39.6, 12.9. HRMS (ESI) for $C_{28}H_{39}N_5NaO_{13}$ ([M+Na$^+$]): calcd 676.2442, found 676.2429.

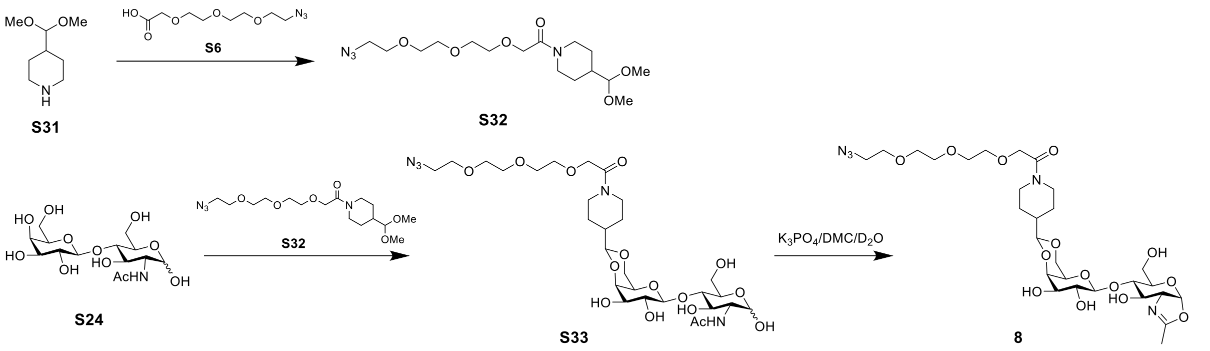

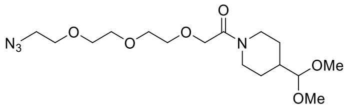

S32

S32 (138.3 mg, yield 92.4%) was synthesized analogously to **S29**. ^{1}H NMR (400 MHz, $CDCl_3$) δ 4.63 – 4.51 (m, 1H), 4.27 – 4.14 (m, 2H), 4.03 (d, *J* = 6.7 Hz, 1H), 3.90 – 3.79 (m, 1H), 3.77 – 3.62 (m, 11H), 3.43 (dd, *J* = 5.6, 4.5 Hz, 2H), 3.36 (s, 6H), 3.02 – 2.90 (m, 1H), 2.55 (td, *J* = 13.0, 2.8 Hz, 1H), 1.91 – 1.73 (m, 3H), 1.33 – 1.13 (m, 2H). ^{13}C NMR (100 MHz, $CDCl_3$) δ 167.6, 107.5, 77.4, 77.1, 76.8, 70.4, 70.3, 70.3, 70.2, 70.1, 69.8, 55.6, 54.1, 54.0, 50.6, 44.6, 43.6, 41.7, 38.8, 27.6, 26.9.

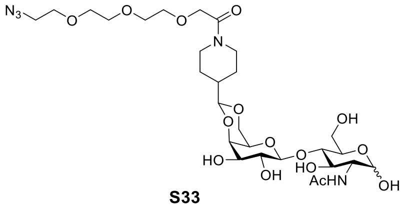

S33

S30 (15.6 mg, yield 45.1%) was synthesized according to the "*General method for acetal/ketal LacNAc synthesis*". ^{1}H NMR (400 MHz, D_2O) δ 5.18 – 5.11 (d, *J* = 2.2 Hz, 0.61H), 4.50-4.40 (m, 2H), 4.35-4.21 (m, 3H), 4.10 – 3.99 (m, 2H), 3.98 – 3.87 (m, 2H), 3.87 – 3.50 (m, 20H), 3.47 – 3.38 (m, 2H), 3.02 (t, *J* = 12.9 Hz, 1H), 2.66 (t, *J* = 12.9 Hz, 1H), 1.97 (s, 3H), 1.86 – 1.70 (m, 3H), 1.39-1.13 (m, 2H). ^{13}C NMR (100 MHz, MeOD) δ 172.7, 172.2, 138.2, 136.3, 127.7, 126.6, 103.6, 100.5, 95.8, 90.9, 79.9, 76.0, 75.2, 72.7, 72.1, 70.5, 70.1, 69.3, 68.8, 66.9, 60.4, 54.2, 53.8, 48.2, 48.0, 47.8, 47.6, 47.4, 47.2, 47.0, 21.5, 21.2. HRMS (ESI) for $C_{28}H_{47}N_5NaO_{15}$ ([M+Na$^+$]): calcd 716.2966, found 716.2963.

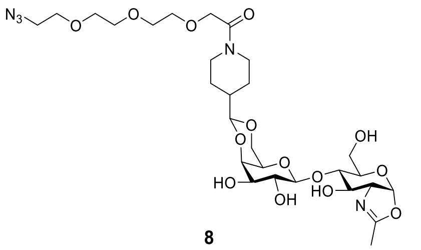

8

Oxazoline **8** (6.8 mg, yield 85.4%) was synthesized according to the "*General method for oxazoline synthesis*". ^1H NMR (400 MHz, D_2O) δ 6.03 (d, J = 7.3 Hz, 1H), 4.49 – 4.39 (m, 3H), 4.34-4.22 (m, 3H), 4.16-4.11 (m, 1H), 4.10 – 4.01 (m, 2H), 3.93 (dd, J = 12.8, 1.7 Hz, 1H), 3.78 – 3.56 (m, 17H), 3.49 (dd, J = 9.9, 7.8 Hz, 1H), 3.45 – 3.36 (m, 3H), 3.00 (t, J = 12.6 Hz, 1H), 2.71-5.59 (m, 1H), 2.01 (d, J = 1.8 Hz, 3H), 1.91 – 1.70 (m, 3H), 1.37 – 1.15 (m, 2H). ^{13}C NMR (150 MHz, D_2O) δ 169.3, 160.3, 104.6, 103.7, 99.9, 78.7, 75.2, 71.4, 70.9, 70.4, 70.0, 69.6, 69.5, 69.2, 68.6, 68.5, 66.9, 65.0, 61.8, 50.2, 44.5, 42.0, 39.4, 26.3, 25.8, 12.9. HRMS (ESI) for $C_{28}H_{46}N_5O_{14}$ ([M+H$^+$]): calcd 676.3041, found 676.3043.

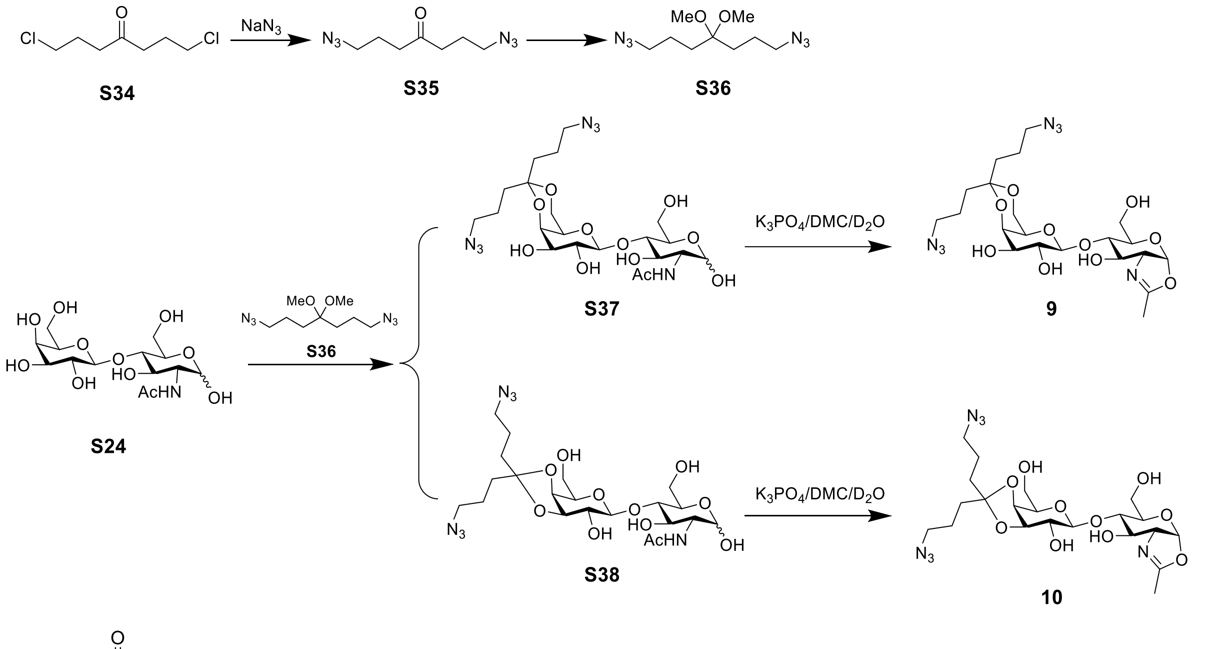

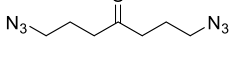

S35

To a solution of **S34** (549 mg, 3 mmol) in DMF (5 mL) was added sodium azide (780 mg, 12 mmol). The reaction mixture was stirred at 80 ℃ for 12 h. The resulting mixture was separated by ethyl acetate and water. The organic layer was further washed with water and sodium bicarbonate aqueous solution. The organic layers were dried, filtered, and purified by silica gel flash column chromatography using ethyl acetate/hexane as the eluents to afford the product (309 mg, yield 52.6%) as an oil. ^1H NMR (400 MHz, $CDCl_3$) δ 3.35 (t, J = 6.6 Hz, 4H), 2.56 (t, J = 7.0 Hz, 4H), 1.90 (p, J = 6.8 Hz, 4H).

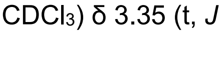

S36

To a solution of **S35** (196 mg, 1 mmol) in MeOH (2 mL) was added $TiCl_4$ (18.9 mg, 0.1 mmol). The

reaction mixture was stirred at 0 °C for 2 h. The resulting mixture was separated by ethyl acetate and water. The organic layer was further washed with sodium bicarbonate aqueous solution and water. The organic layers were dried, filtered, and purified by silica gel flash column chromatography using ethyl acetate/hexane as the eluents to afford a mixture of **S36**/**S35** (1/1.5, 208 mg) as an oil. ^{1}H NMR (400 MHz, $CDCl_3$) δ 3.35 – 3.28 (m, 4H), 3.16 (s, 6H), 1.70 – 1.61 (m, 4H), 1.61 – 1.51 (m, 4H).

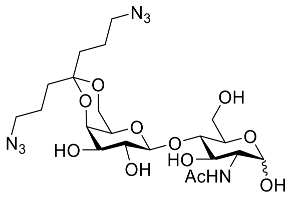

S37

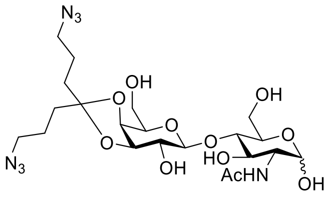

S38

S37 (7.8 mg, yield 28.1%) and **S38** (10.6 mg, yield 38.1%) were synthesized in a single reaction according to the "*General method for acetal/ketal LacNAc synthesis*".

S37: ^{1}H NMR (400 MHz, D_2O) δ 5.13 (d, *J* = 2.8 Hz, 0.67H), 4.45 (d, *J* = 7.7 Hz, 1H), 4.23 (d, *J* = 3.4 Hz, 1H), 4.16 (d, *J* = 13.2 Hz, 1H), 3.95 – 3.72 (m, 5H), 3.71 – 3.52 (m, 5H), 3.37 – 3.24 (m, 4H), 2.03 – 1.84 (m, 5H), 1.76 – 1.62 (m, 4H), 1.58 – 1.47 (m, 2H). ^{13}C NMR (150 MHz, D_2O) δ 174.7, 174.4, 102.6, 101.6, 94.8, 90.5, 79.1, 78.9, 74.7, 72.4, 71.4, 70.4, 70.2, 69.2, 67.8, 66.2, 61.8, 60.1, 59.9, 56.2, 53.7, 51.1, 51.1, 51.0, 34.7, 26.2, 26.2, 23.1, 22.8, 22.2, 21.9, 21.6. HRMS (ESI) for $C_{21}H_{35}N_7NaO_{11}$ ([M+Na$^+$]): calcd 584.2292, found 584.2280.

S38: ^{1}H NMR (400 MHz, D_2O) δ 5.13 (d, *J* = 2.3 Hz, 0.65H), 4.45 (d, *J* = 8.3 Hz, 1H), 4.32 (dd, *J* = 5.9, 2.1 Hz, 1H), 4.24 – 4.16 (m, 1H), 4.05 – 3.98 (m, 1H), 3.92 – 3.84 (m, 1H), 3.84 – 3.71 (m, 5H), 3.68 – 3.64 (m, 1H), 3.50 – 3.43 (m, 1H), 3.31 (t, *J* = 6.6 Hz, 2H), 3.27 (t, *J* = 6.5 Hz, 2H), 1.97 (s, 3H), 1.83 – 1.50 (m, 9H). ^{13}C NMR (150 MHz, D_2O) δ 174.7, 174.5, 113.3, 102.2, 94.8, 90.5, 79.2, 78.8, 78.6, 74.8, 73.8, 73.7, 73.0, 72.4, 70.2, 69.2, 60.8, 60.1, 59.9, 56.3, 53.7, 51.0, 50.8, 34.2, 33.0, 23.1, 23.1, 22.2, 21.9. HRMS (ESI) for $C_{21}H_{35}N_7NaO_{11}$ ([M+Na$^+$]): calcd 584.2292, found 584.2269.

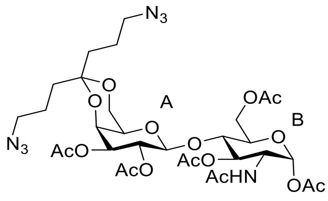

S37-Ac

The total acetylation was done as previously reported[20]. ^{1}H NMR (400 MHz, DMSO-d_6) δ 7.96 (d, *J* = 9.2 Hz, 1H, AcN*H*), 5.86 (d, *J* = 3.5 Hz, 1H, H-1^{B}), 5.12 (dd, *J* = 11.2, 9.1 Hz, 1H, H-3^{B}), 4.99 (dd, *J* = 10.4, 3.2 Hz, 1H, H-3^{A}), 4.94 (dd, *J* = 10.3, 7.5 Hz, 1H, H-2^{A}), 4.62 (d, *J* = 7.6 Hz, 1H, H-1^{A}), 4.34 – 4.27 (m, 1H, H-6a^{B}), 4.25 (d, *J* = 3.4 Hz, 1H, H-4^{A}), 4.22 – 4.15 (m, 1H, H-2^{B}), 4.13 – 4.04 (m, 2H, H-6b^{B}), 3.96 – 3.89 (m, 1H, H-5^{B}), 3.81 – 3.71 (m, 2H, H-4^{B}), 3.62 (s, 1H), 3.37 (t, *J* = 6.2 Hz, 2H), 2.17 (s, 3H), 2.08 (s, 3H), 2.06 – 1.95 (m, 10H), 1.80 (s, 3H), 1.76 – 1.43 (m, 6H), 1.42 – 1.30 (m, 1H). ^{13}C NMR (150 MHz, DMSO-d_6) δ 170.7, 170.4, 170.2, 169.9, 169.7, 169.6, 100.7, 100.6, 90.2, 76.9, 71.9, 70.9, 70.0, 69.2, 66.2, 65.5, 62.2, 61.7, 51.6, 50.8, 50.4, 35.6, 26.6, 23.5, 22.7, 22.2, 21.4, 21.1, 21.0, 20.9, 20.8.

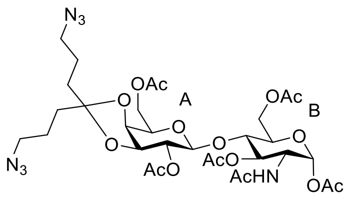

S38

^{1}H NMR (400 MHz, DMSO-d6) δ 7.97 (d, *J* = 9.2 Hz, 1H, AcN*H*), 5.86 (d, *J* = 3.5 Hz, 1H, H-1^B), 5.07 (dd, *J* = 11.1, 9.0 Hz, 1H, H-3^B), 4.66 (dd, *J* = 8.2, 6.7 Hz, 1H, H-2^A), 4.49 (d, *J* = 8.1 Hz, 1H, H-1^A), 4.32 – 4.25 (m, 2H, H-3^A and H-6a^B), 4.25 – 4.12 (m, 5H, H-2^B), 4.05 (dd, *J* = 12.2, 5.2 Hz, 1H, H-6b^B), 3.95 – 3.88 (m, 1H, H-5^B), 3.80 (t, *J* = 9.6 Hz, 1H, H-4^B), 2.16 (s, 3H), 2.06 (d, *J* = 7.7 Hz, 9H), 1.96 (s, 3H), 1.80 (s, 3H), 1.78 – 1.57 (m, 6H), 1.55 – 1.47 (m, 2H). ^{13}C NMR (150 MHz, DMSO-d6) δ 170.7, 170.6, 170.4, 169.9, 169.7, 169.6, 112.4, 100.4, 90.2, 77.2, 76.3, 73.8, 73.6, 70.9, 70.5, 70.2, 63.0, 62.3, 51.2, 51.0, 50.5, 34.5, 33.8, 23.7, 23.4, 22.7, 21.3, 21.1, 21.0, 20.9.

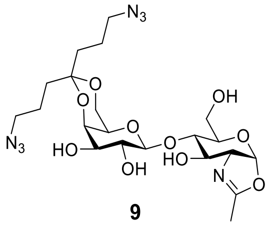

9

Oxazoline **9** (5.0 mg, yield 86.9%) was synthesized according to the "*General method for oxazoline synthesis*". ^{1}H NMR (400 MHz, D_2O) δ 6.02 (d, *J* = 7.3 Hz, 1H), 4.44 – 4.37 (m, 2H), 4.21 (d, *J* = 3.6 Hz, 1H), 4.19 – 4.10 (m, 2H), 3.89 – 3.80 (m, 1H), 3.77 (dd, *J* = 12.4, 2.5 Hz, 1H), 3.68 – 3.57 (m, 3H), 3.57 – 3.49 (m, 2H), 3.43 – 3.36 (m, 1H), 3.36 – 3.27 (m, 4H), 2.04 – 1.92 (m, 4H), 1.91 – 1.81 (m, 1H), 1.75 – 1.61 (m, 4H), 1.58 – 1.46 (m, 2H). ^{13}C NMR (150 MHz, D_2O) δ 168.3, 104.1, 101.6, 99.9, 78.3, 71.5, 70.9, 70.3, 68.9, 67.8, 66.2, 65.3, 62.0, 61.7, 51.2, 51.0, 34.8, 25.8, 22.8, 21.8, 13.0. HRMS (ESI) for $C_{21}H_{33}N_7NaO_{10}$ ([M+Na$^+$]): calcd 566.2187, found 566.2182; and $C_{21}H_{33}KN_7O_{10}$ ([M+K$^+$]): calcd 582.1926, found 582.1922.

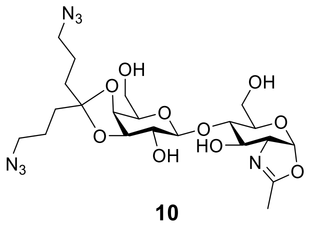

10

Oxazoline **9** (5.4 mg, yield 73.7%) was synthesized according to the "*General method for oxazoline synthesis*". ^{1}H NMR (400 MHz, D_2O) δ 6.02 (d, *J* = 7.3 Hz, 1H), 4.39 (d, *J* = 8.3 Hz, 1H), 4.32 (dd, *J* = 3.1, 1.7 Hz, 1H), 4.30 (dd, *J* = 5.7, 2.1 Hz, 1H), 4.19 – 4.10 (m, 2H), 4.02 – 3.96 (m, 1H), 3.83 – 3.76 (m, 2H), 3.73 (dd, *J* = 12.4, 2.5 Hz, 1H), 3.66 (d, *J* = 9.0 Hz, 1H), 3.59 (dd, *J* = 12.3, 6.2 Hz, 1H), 3.43 (t, *J* = 7.8 Hz, 1H), 3.37 (ddd, *J* = 8.9, 6.2, 2.5 Hz, 1H), 3.31 (t, *J* = 6.6 Hz, 2H), 3.26 (t, *J* = 6.5 Hz, 2H), 1.99 (d, *J* = 1.8 Hz, 3H), 1.84 – 1.73 (m, 2H), 1.73 – 1.61 (m, 4H), 1.60 – 1.51 (m, 2H). ^{13}C NMR (150 MHz, D_2O) δ 168.4, 113.3, 103.7, 99.8, 78.5, 78.2, 73.8, 73.7, 72.9, 70.9, 69.1, 65.2, 61.6, 60.8, 51.0, 50.9, 38.7, 34.3, 33.1, 23.1, 12.9. HRMS (ESI) for $C_{21}H_{33}N_7NaO_{10}$ ([M+Na$^+$]): calcd 566.2187, found 566.2180; and $C_{21}H_{33}KN_7O_{10}$ ([M+K$^+$]): calcd 582.1926, found 582.1920.

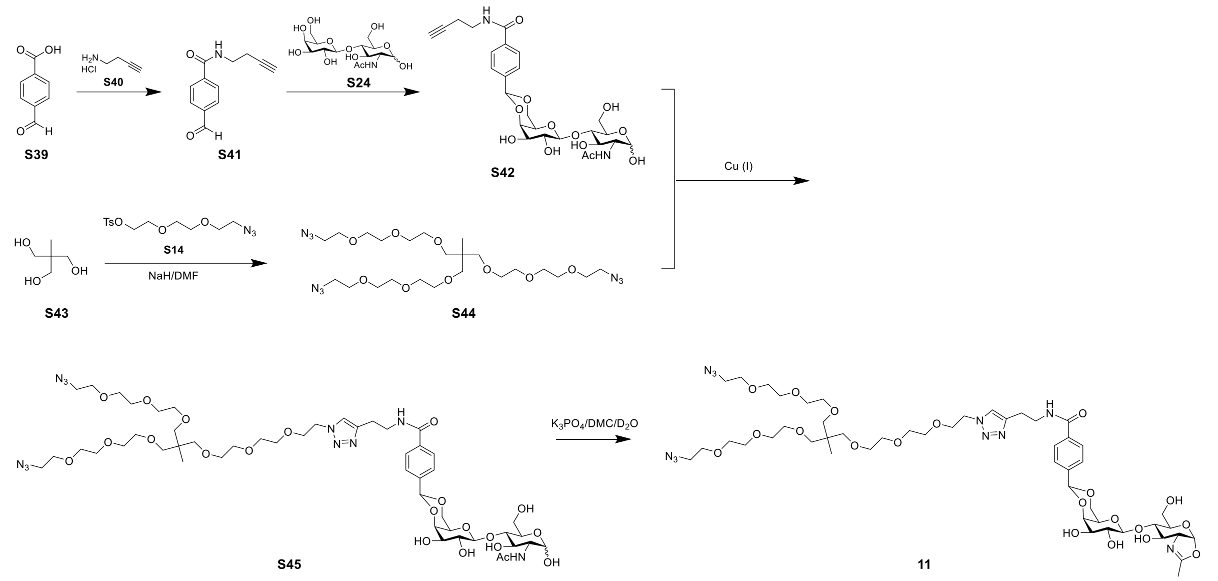

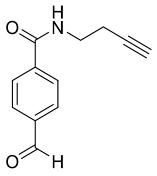

S41

S41 (0.37 g, yield 69.2%) was synthesized analogously to **S29**. ^{1}H NMR (400 MHz, $CDCl_3$) δ 10.07 (s, 1H), 7.94 (s, 4H), 6.79 (s, 1H), 3.64 (q, *J* = 6.3 Hz, 2H), 2.55 (td, *J* = 6.5, 2.7 Hz, 2H), 2.07 (d, *J* = 5.3 Hz, 1H). ^{13}C NMR (100 MHz, $CDCl_3$) δ 191.6, 166.6, 139.5, 138.3, 129.9, 127.7, 81.3, 77.4, 77.1, 76.8, 70.4, 38.6, 19.4.

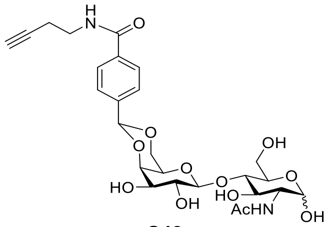

S42

S30 (33.1 mg, yield 45.0%) was synthesized according to the "*General method for acetal/ketal LacNAc synthesis*". ^{1}H NMR (400 MHz, D_2O) δ 7.74 (d, *J* = 8.1 Hz, 2H), 7.59 (d, *J* = 8.1 Hz, 2H), 5.75 (s, 1H), 5.14 (d, *J* = 3.0 Hz, 0.72H), 4.51 (d, *J* = 7.9 Hz, 1H), 4.33 (d, *J* = 3.6 Hz, 1H), 4.25 – 4.15 (m, 2H), 3.95 – 3.88 (m, 1H), 3.88 – 3.74 (m, 5H), 3.69 – 3.63 (m, 2H), 3.49 (t, *J* = 6.6 Hz, 2H), 2.48 (td, *J* = 6.6, 2.7 Hz, 2H), 2.29 (t, *J* = 2.6 Hz, 1H), 1.96 (s, 3H). ^{13}C NMR (100 MHz, D_2O) δ 174.4, 170.7, 168.1, 140.2, 134.8, 127.3, 126.5, 102.9, 100.3, 94.9, 90.5, 82.4, 79.1, 78.9, 75.7, 74.8, 72.4, 71.2, 70.4, 70.3, 69.1, 69.0, 66.6, 59.8, 56.1, 53.7, 38.5, 22.1, 21.8, 18.4. HRMS (ESI) for $C_{26}H_{34}N_2NaO_{12}$ ([M+Na$^+$]): calcd 589.2009, found 589.1997.

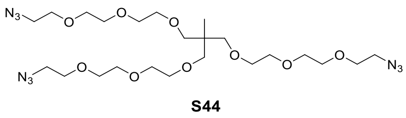

S44

S44 (61.5 mg, yield 57.8%) was synthesized analogously to **S15**. ^{1}H NMR (400 MHz, $CDCl_3$) δ 3.70 –

3.64 (m,18H), 3.64 – 3.59 (m, 6H), 3.58 – 3.53 (m, 6H), 3.38 (t, J = 5.1 Hz, 6H), 3.31 (s, 6H), 0.93 (s, 3H). ^{13}C NMR (100 MHz, $CDCl_3$) δ 77.4, 77.0, 76.7, 73.9, 71.1, 70.8, 70.7, 70.5, 70.1, 50.7, 41.0, 17.3.

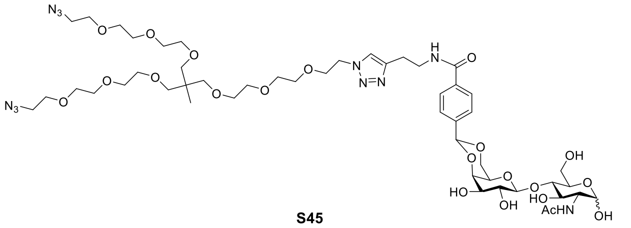

S45

S45 (12.3 mg, yield 48.9%) was synthesized according to the "*General method for click chemistry*". ^{1}H NMR (400 MHz, D_2O) δ 7.82 (s, 1H), 7.67 (d, J = 8.1 Hz, 2H), 7.58 (d, J = 8.1 Hz, 2H), 5.72 (s, 1H), 5.14 (d, J = 2.8 Hz, 0.64H), 4.50 (t, J = 5.4 Hz, 3H), 4.31 (d, J = 3.6 Hz, 1H), 4.26 – 4.13 (m, 2H), 3.98 – 3.72 (m, 9H), 3.71 – 3.51 (m, 28H), 3.50 – 3.36 (m, 12H), 3.34 – 3.23 (m, 6H), 2.99 (t, J = 6.6 Hz, 2H), 1.96 (s, 3H), 0.82 (s, 3H). ^{13}C NMR (125 MHz, D_2O) δ 174.8, 174.6, 170.3, 145.3, 140.6, 134.8, 127.4, 126.8, 124.2, 103.1, 100.4, 95.0, 90.6, 79.4, 79.1, 75.9, 75.0, 73.6, 73.6, 72.5, 71.4, 70.7, 70.7, 70.6, 70.4, 69.9, 69.8, 69.8, 69.7, 69.7, 69.6, 69.4, 69.3, 69.1, 69.0, 66.8, 60.1, 60.0, 56.3, 53.9, 50.3, 50.2, 40.6, 39.5, 24.9, 22.3, 22.0, 16.9.

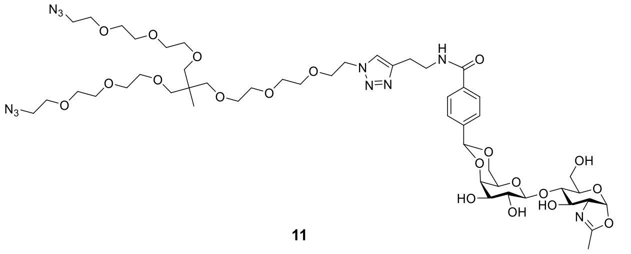

11

Oxazoline **11** (9.7 mg, yield 81.6%) was synthesized according to the "*General method for oxazoline synthesis*". ^{1}H NMR (400 MHz, D_2O) δ 7.82 (s, 1H), 7.67 (d, J = 8.4 Hz, 2H), 7.59 (d, J = 8.4 Hz, 2H), 6.03 (d, J = 7.3 Hz, 1H), 5.71 (s, 1H), 4.53 – 4.43 (m, 4H), 4.30 (d, J = 3.7 Hz, 1H), 4.27 – 4.16 (m, 2H), 4.16 – 4.10 (m, 1H), 3.84 (t, J = 4.9 Hz, 2H), 3.81 – 3.36 (m, 41H), 3.34 – 3.23 (m, 6H), 2.99 (t, J = 6.6 Hz, 2H), 2.00 (d, J = 1.7 Hz, 3H), 0.82 (s, 3H). ^{13}C NMR (125 MHz, D_2O) δ 170.3, 168.4, 160.4, 145.3, 140.6, 134.8, 127.4, 126.9, 124.2, 104.8, 100.5, 100.0, 78.9, 76.0, 73.6, 71.4, 71.1, 70.7, 70.7, 70.5, 69.9, 69.8, 69.8, 69.7, 69.7, 69.6, 69.4, 69.2, 69.0, 68.8, 66.8, 65.2, 61.9, 50.3, 40.6, 39.5, 24.9, 16.9, 13.1. HRMS (ESI) for $C_{49}H_{77}N_{11}NaO_{20}$ ([M+Na^+]): calcd 1162.5244, found 1162.5216.

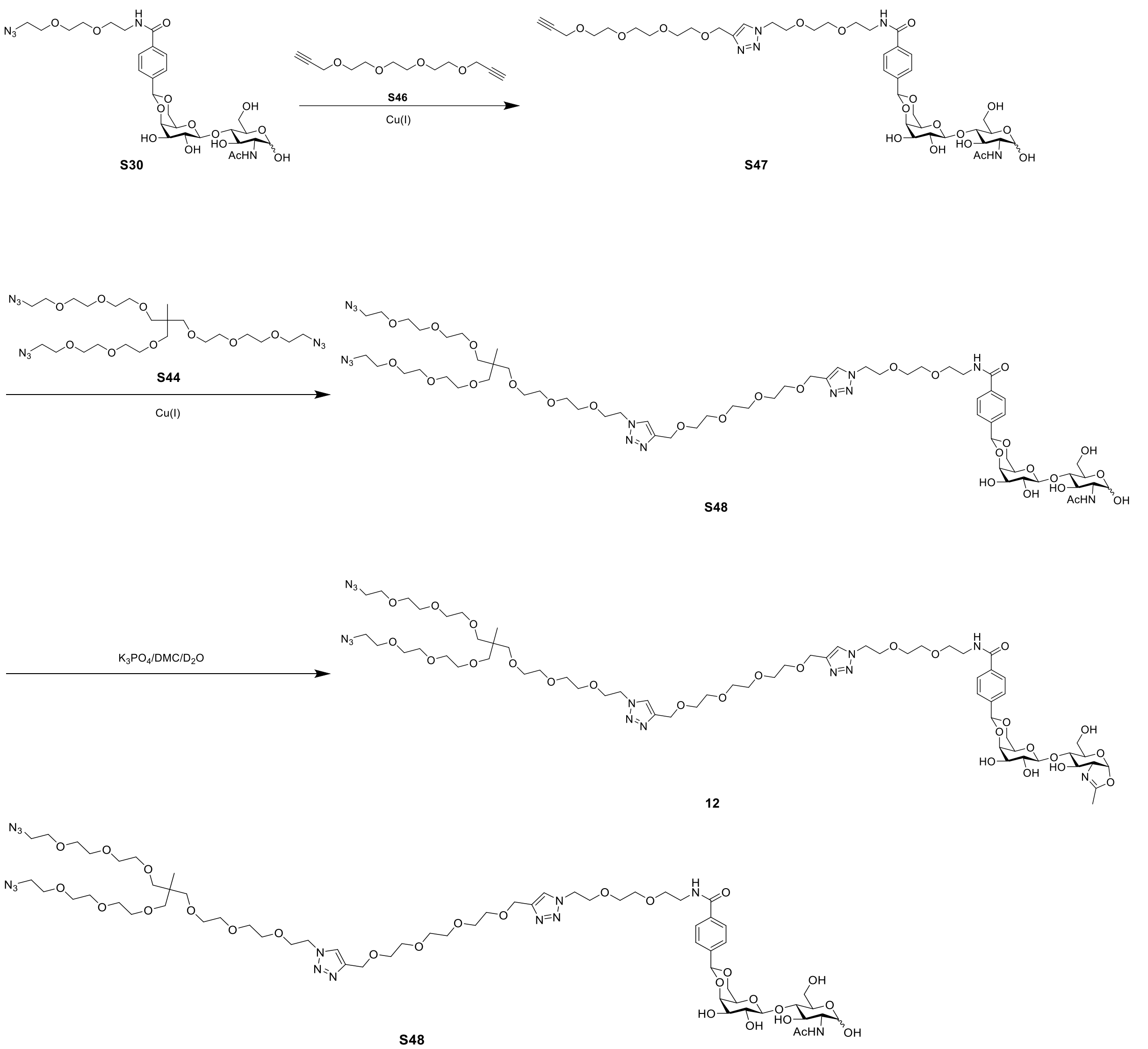

To a solution of **S30** (16 mg, 0.024 mmol), **S46** (27 mg, 0.12 mmol), ligand tris[(1-benzyl-1H-1,2,3-triazol-4-yl)methyl]amine (6.4 mg, 0.012 mmol) and copper(**II**) sulfate (1.9 mg, 0.012 mmol) in ACN/water (1/1, 1.6 mL) was added sodium ascorbate (9.5 mg, 0.048 mmol). The reaction was stirred at room temperature for 2 h. The reaction mixture was concentrated and diluted with water (1 mL). The aqueous solution was washed with ethyl ether (X 5) to remove excess **S46**. The **S47** containing aqueous solution was used for next step directly.

To a solution of **S44** (35.5 mg, 0.06 mmol), ligand tris[(1-benzyl-1H-1,2,3-triazol-4-yl)methyl]amine (15.9 mg, 0.03 mmol) and copper(**II**) sulfate (4.8 mg, 0.03 mmol) in DMF/water (10/1, 2.2 mL) was added sodium ascorbate (1.0 e.q.) under protection of nitrogen. Then, **S47** (crude from above in ~1 mL water) was added in portions (0.2 e.q./10 min). The reaction was stirred at room temperature for an additional 1 h. The reaction mixture was purified by preparative HPLC C18 reverse phase column eluting with acetonitrile/water (0.1% formic acid). **S48** (14.4 mg, yield 40.3% over two steps) was afforded. ^{1}H NMR (400 MHz, D_2O) δ 7.98 (s, 1H), 7.92 (s, 1H), 7.70 (d, *J* = 8.5 Hz, 2H), 7.56 (d, *J* = 8.5 Hz, 2H), 5.71 (s, 1H), 5.14 (d, *J* = 2.8 Hz, 0.64 H), 4.67 – 4.63 (m, 0.36 H), 4.58 (s, 2H), 4.56 – 4.50 (m, 5H), 4.48 (dd, *J* = 5.6, 4.4 Hz, 2H), 4.32 (d, *J* = 3.6 Hz, 1H), 4.24 – 4.14 (m, 2H), 3.95 – 3.82 (m, 8H), 3.82 – 3.78 (m, 1H), 3.78 – 3.73 (m, 2H), 3.69 – 3.46 (m, 58H), 3.40 (dd, *J* = 5.6, 4.2 Hz, 4H), 3.34 – 3.28 (m, 6H), 1.95 (d, *J* = 1.4 Hz, 3H), 0.83 (s, 3H). ^{13}C NMR (101 MHz, D_2O) δ 174.6, 174.4, 170.2, 143.8, 143.8, 140.5,

134.5, 127.2, 126.6, 125.4, 125.3, 103.0, 100.2, 94.9, 90.5, 79.3, 79.0, 75.7, 74.8, 73.5, 73.4, 72.4, 71.2, 70.6, 70.5, 70.3, 69.7, 69.7, 69.7, 69.6, 69.6, 69.5, 69.5, 69.4, 69.2, 69.1, 68.9, 68.7, 68.7, 66.6, 63.1, 63.0, 59.8, 56.2, 53.7, 50.2, 50.0, 49.9, 40.4, 39.5, 22.2, 21.9, 16.8. HRMS (ESI) for $C_{63}H_{104}N_{14}Na_2O_{27}$ ([M+2Na^{2+}]): calcd 767.3490, found 767.3495.

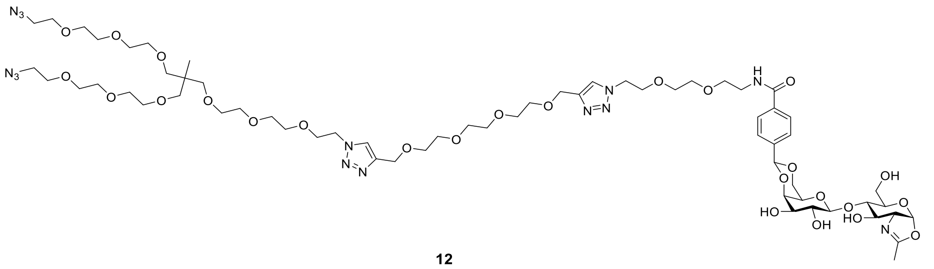

12

Oxazoline **12** (9.0 mg, yield 63.7%) was synthesized according to the "*General method for oxazoline synthesis*". ^{1}H NMR (400 MHz, D_2O) δ 7.98 (s, 1H), 7.92 (s, 1H), 7.70 (d, *J* = 8.1 Hz, 2H), 7.57 (d, *J* = 8.1 Hz, 2H), 6.02 (d, *J* = 7.3 Hz, 1H), 5.70 (s, 1H), 4.58 (s, 2H), 4.56 – 4.46 (m, 7H), 4.46 – 4.42 (m, 1H), 4.30 (d, *J* = 3.7 Hz, 1H), 4.26 – 4.16 (m, 2H), 4.15 – 4.09 (m, 1H), 3.87 (dt, *J* = 7.3, 5.0 Hz, 4H), 3.81 – 3.45 (m, 58H), 3.44 – 3.36 (m, 5H), 3.35 – 3.25 (m, 6H), 1.98 (d, *J* = 1.8 Hz, 3H), 0.83 (s, 3H). ^{13}C NMR (125 MHz, D_2O) δ 171.2, 170.4, 144.0, 144.0, 140.6, 134.7, 127.4, 126.8, 125.6, 125.5, 104.8, 100.5, 100.0, 78.9, 76.0, 73.6, 73.6, 71.4, 71.1, 70.7, 70.7, 70.6, 69.9, 69.8, 69.8, 69.8, 69.7, 69.7, 69.7, 69.7, 69.7, 69.7, 69.6, 69.4, 69.3, 69.1, 69.1, 68.9, 68.9, 68.8, 66.8, 65.3, 65.2, 63.2, 63.2, 61.9, 50.3, 50.2, 50.1, 40.6, 39.7, 17.0, 13.1. HRMS (ESI) for $C_{63}H_{102}N_{14}Na_2O_{26}$ ([M+2Na^{2+}]): calcd 758.3437, found 758.3430.

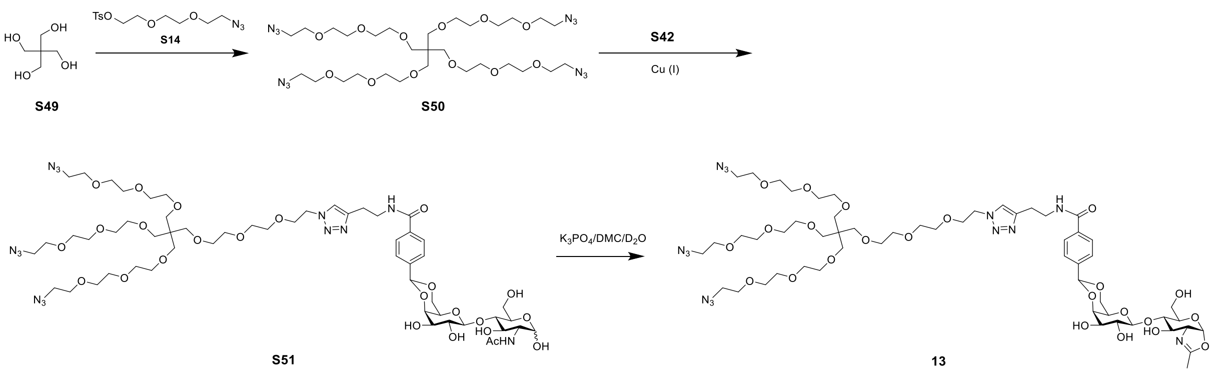

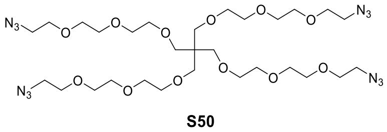

S50

S50 (199 mg, yield 38.9%) was synthesized analogously to **S15**. ^{1}H NMR (400 MHz, $CDCl_3$) δ 3.70 – 3.62 (m, 18H), 3.60 (td, *J* = 4.4, 1.0 Hz, 6H), 3.57 – 3.52 (m, 6H), 3.42 (s, 6H), 3.38 (t, *J* = 5.1 Hz, 6H). ^{13}C NMR (100 MHz, $CDCl_3$) δ 77.4, 77.1, 76.8, 71.1, 70.8, 70.7, 70.5, 70.1, 50.7.

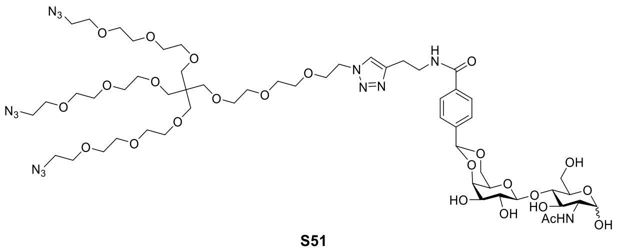

S51

S51 (11.2 mg, yield 35.7%) was synthesized according to the "*General method for click chemistry*". 1H NMR (400 MHz, D2O) δ 7.83 (s, 1H), 7.70 – 7.65 (m, 2H), 7.60 – 7.55 (m, 2H), 5.72 (s, 1H), 5.14 (d, J = 2.8 Hz, 0.67H), 4.64 (d, J = 6.7 Hz, 1H), 4.54 – 4.47 (m, 3H), 4.31 (d, J = 3.6 Hz, 1H), 4.24 – 4.14 (m, 2H), 3.95 – 3.70 (m, 9H), 3.68 – 3.51 (m, 36H), 3.51 – 3.31 (m, 23H), 2.99 (t, J = 6.7 Hz, 2H), 1.99 – 1.94 (m, 3H). ^{13}C NMR (125 MHz, D_2O) δ 174.6, 171.2, 170.2, 145.3, 140.6, 134.8, 127.4, 126.8, 124.3, 103.1, 100.4, 95.0, 90.6, 79.4, 79.1, 75.9, 75.0, 72.5, 71.4, 70.8, 70.6, 70.4, 69.9, 69.8, 69.8, 69.8, 69.7, 69.4, 69.3, 69.1, 69.0, 66.8, 60.1, 60.0, 56.3, 53.9, 50.3, 50.2, 45.2, 39.6, 24.9, 22.3, 22.0.

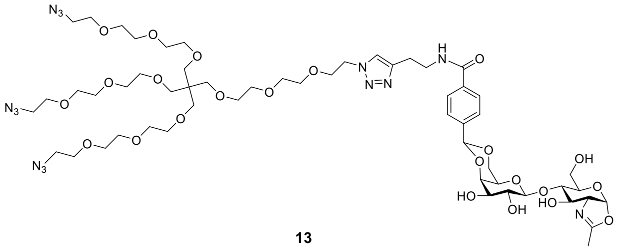

13

Oxazoline **13** (5.7 mg, yield 63.9%) was synthesized according to the "*General method for oxazoline synthesis*". ^{1}H NMR (400 MHz, D_2O) δ 7.83 (s, 1H), 7.67 (d, *J* = 8.4 Hz, 2H), 7.59 (d, *J* = 8.4 Hz, 2H), 6.03 (d, *J* = 7.3 Hz, 1H), 5.71 (s, 1H), 4.54 – 4.42 (m, 4H), 4.30 (d, *J* = 3.7 Hz, 1H), 4.27 – 4.16 (m, 2H), 4.16 – 4.10 (m, 1H), 3.84 (t, *J* = 4.9 Hz, 2H), 3.79 – 3.70 (m, 3H), 3.66 – 3.51 (m, 35H), 3.51 – 3.32 (m, 23H), 2.99 (t, *J* = 6.7 Hz, 2H), 2.00 (d, *J* = 1.8 Hz, 3H). ^{13}C NMR (125 MHz, D_2O) δ 170.3, 168.4, 160.7, 145.3, 140.6, 134.8, 127.4, 126.9, 124.3, 104.8, 100.5, 100.0, 78.9, 76.0, 71.4, 71.1, 70.8, 70.7, 70.5, 69.9, 69.8, 69.8, 69.8, 69.7, 69.4, 69.3, 69.0, 68.8, 66.8, 65.2, 61.9, 50.3, 45.2, 39.6, 24.9, 13.1. HRMS (ESI) for $C_{55}H_{88}N_{14}NaO_{23}$ ([M+Na$^+$]): calcd 1335.6044, found 1335.6029; and $C_{55}H_{88}KN_{14}O_{23}$ ([M+K$^+$]): calcd 1351.5784, found 1351.5772.

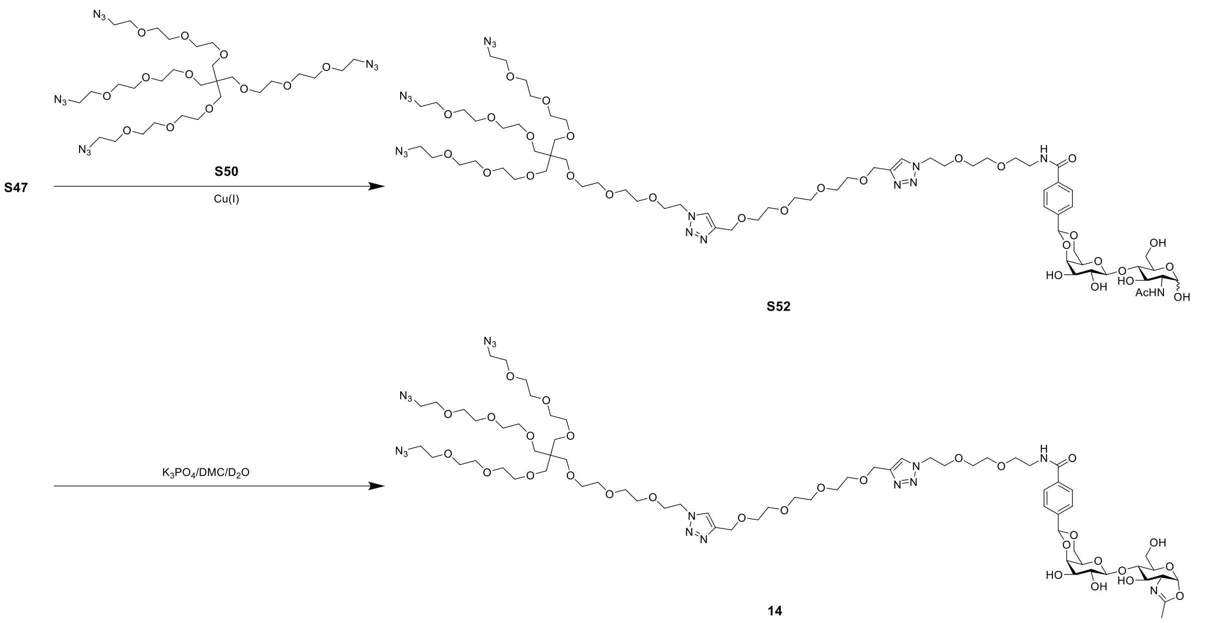

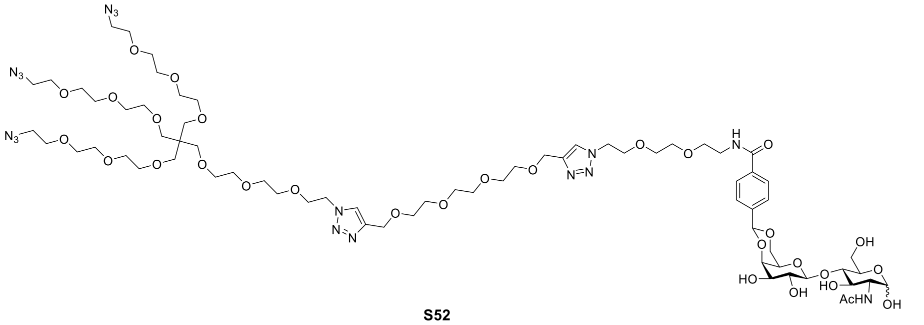

S52

S52 (199 mg, yield 38.9%) was synthesized analogously to **S48**. ^{1}H NMR (400 MHz, D_2O) δ 7.99 (d, *J* = 24.3 Hz, 2H), 7.70 (d, *J* = 7.8 Hz, 2H), 7.56 (d, *J* = 7.8 Hz, 2H), 5.69 (s, 1H), 5.14 (s, 0.69H), 4.63 – 4.38 (m, 10H), 4.31 (s, 1H), 4.18 (s, 2H), 4.04 – 3.14 (m, 108H), 1.96 (s, 3H). ^{13}C NMR (101 MHz, D_2O) δ 174.5, 174.3, 169.9, 169.9, 140.5, 140.5, 134.4, 127.2, 126.6, 103.0, 100.1, 94.9, 90.5, 79.4, 79.1, 75.7, 74.8, 72.4, 71.3, 70.6, 70.6, 70.5, 70.3, 69.8, 69.7, 69.7, 69.7, 69.6, 69.6, 69.6, 69.5, 69.4, 69.4, 69.3, 69.2, 69.1, 69.0, 68.9, 68.8, 68.7, 68.7, 68.0, 66.6, 63.1, 60.0, 59.9, 56.2, 53.7, 50.2, 50.1, 50.0, 45.0, 44.7, 39.5, 22.2, 21.9.

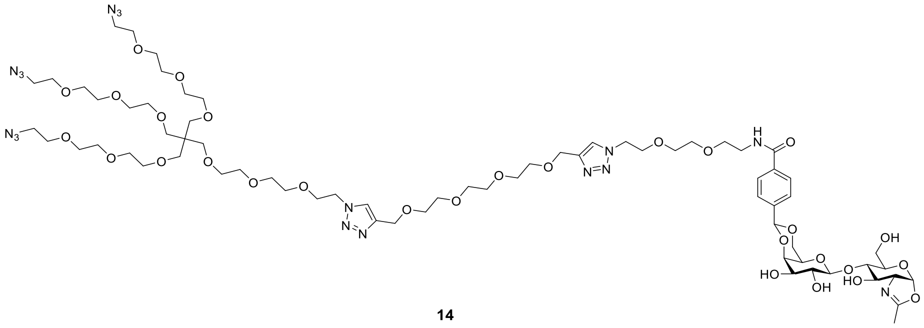

14

Oxazoline **14** (5.5 mg, yield 51.9%) was synthesized according to the "*General method for oxazoline synthesis*". ^{1}H NMR (400 MHz, D_2O) δ 7.98 (s, 1H), 7.92 (s, 1H), 7.71 (d, *J* = 8.1 Hz, 2H), 7.58 (d, *J* = 8.1 Hz, 2H), 6.02 (d, *J* = 7.2 Hz, 1H), 5.71 (s, 1H), 4.58 (s, 2H), 4.56 – 4.42 (m, 8H), 4.30 (d, *J* = 3.7 Hz, 1H), 4.19 (d, *J* = 16.7 Hz, 2H), 4.13 (s, 1H), 3.87 (dt, *J* = 9.9, 5.2 Hz, 4H), 3.81 – 3.71 (m, 4H), 3.70 – 3.46 (m, 77H), 3.46 – 3.33 (m, 19H), 1.99 (d, *J* = 1.7 Hz, 3H). ^{13}C NMR (126 MHz, D_2O) δ 171.2, 170.4,

144.0, 144.0, 140.7, 134.7, 127.4, 126.9, 125.6, 125.5, 104.8, 100.5, 100.0, 78.9, 76.0, 71.4, 71.1, 70.8, 70.7, 70.6, 69.9, 69.9, 69.8, 69.8, 69.7, 69.7, 69.7, 69.6, 69.5, 69.4, 69.4, 69.4, 69.3, 69.1, 69.1, 69.0, 68.9, 68.9, 68.8, 68.1, 66.8, 65.2, 63.2, 63.2, 61.9, 50.3, 50.2, 50.1, 45.2, 44.8, 39.7, 13.1. HRMS (ESI) for $C_{69}H_{115}N_{17}O_{29}$ ([M+2H^{2+}]): calcd 822.9018, found 822.9029.

Synthesis of ARV771-DBCO

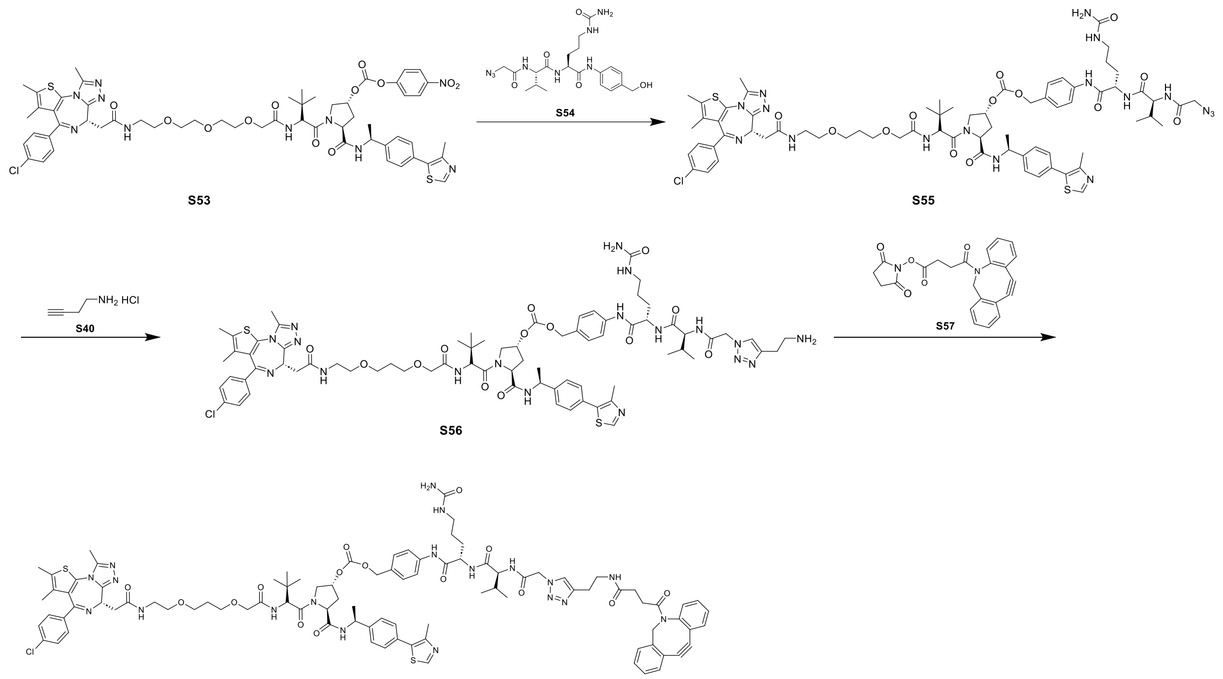

S53 was synthesized according to the reported work[21]. To a solution of **S53** (25 mg, 0.021 mmol), **S54** (10.8 mg, 0.023 mmol), and DIEA (7.3 μL, 0.042 mmol) in DMF (1 mL) was added 4-(dimethylamino)pyridine (1.3 mg, 0.011 mmol). The reaction was stirred at room temperature for 5 h. The reaction mixture was purified by preparative HPLC C18 reverse phase column eluting with acetonitrile/water (0.1% TFA) to afford **S55** (15.4 mg, yield 49.3%). ^{1}H NMR (400 MHz, DMSO-d_6) δ 9.99 (s, 1H), 8.91 (s, 1H), 8.40 (d, *J* = 7.7 Hz, 1H), 8.27 – 8.16 (m, 3H), 8.06 (d, *J* = 8.6 Hz, 1H), 7.55 (d, *J* = 8.3 Hz, 2H), 7.41 (d, *J* = 8.4 Hz, 2H), 7.39 – 7.32 (m, 5H), 7.32 – 7.20 (m, 5H), 5.93 (t, *J* = 5.8 Hz, 1H), 5.36 (s, 2H), 5.08 – 4.95 (m, 2H), 4.83 (p, *J* = 7.1 Hz, 1H), 4.48 – 4.27 (m, 4H), 4.22 (dd, *J* = 8.7, 6.5 Hz, 1H), 3.95-3.80 (m, 4H), 3.76 – 3.61 (m, 1H), 3.52 – 3.32 (m, 6H), 3.25 – 3.09 (m, 2H), 3.03 – 2.82 (m, 2H), 2.52 (s, 3H), 2.38 (s, 3H), 2.33 (s, 3H), 2.31 – 2.21 (m, 1H), 1.99 – 1.85 (m, 2H), 1.72 (p, *J* = 6.5 Hz, 2H), 1.64 (q, *J* = 7.2 Hz, 1H), 1.55 (s, 4H), 1.40 (d, *J* = 7.0 Hz, 1H), 1.37 – 1.25 (m, 4H), 1.19 – 0.96 (m, 3H), 0.92 – 0.83 (m, 9H), 0.81 (d, *J* = 6.7 Hz, 3H), 0.76 (d, *J* = 6.8 Hz, 3H). ^{13}C NMR (100 MHz, DMSO-d_6) δ 171.2, 171.1, 170.1, 170.0, 169.6, 169.2, 168.0, 163.5, 159.4, 156.8, 155.6, 154.2, 152.7, 152.0, 150.3, 148.2, 145.0, 144.8, 139.7, 137.2, 135.7, 132.7, 131.6, 131.2, 130.6, 130.3, 130.2, 130.0, 129.7, 129.3, 128.9, 126.8, 125.6, 123.2, 119.5, 77.3, 69.9, 69.4, 68.5, 67.5, 58.5, 58.0, 56.4, 54.3, 54.0, 53.7, 51.0, 48.4, 46.2, 42.4, 42.2, 39.1, 38.0, 35.8, 35.1, 31.2, 29.9, 29.6, 27.4, 26.6, 23.0, 19.6, 18.5, 16.5, 14.5, 13.1, 11.8.

To a solution of **S55** (8.1 mg, 0.0055 mmol), **S40** (0.69 mg, 0.0066 mmol), and copper(II) sulfate (0.88 mg, 0.0055 mmol) in DMF/water (10/1, 1.1 mL) was added sodium ascorbate (1.1 mg, 0.0055 mmol). The reaction was stirred at room temperature for 2 h. The reaction mixture was purified by preparative

HPLC C18 reverse phase column eluting with acetonitrile/water (0.1% TFA) to afford **S56** (5.9 mg). **S56** (5.9 mg, 0.0038 mmol) and triethylamine (0.79 μL, 0.0057 mmol) in DMF (0.5 mL) was added **S57** (1.7 mg, 0.0042 mmol). The reaction was stirred at room temperature for 1 h. The reaction mixture was purified by preparative HPLC C18 reverse phase column eluting with acetonitrile/NH_4HCO_3(a.q., 10 mM) to afford **S56** (3.5 mg, 34.8% over two steps). ^1H NMR (400 MHz, DMSO-d_6) δ 10.01 (s, 1H), 8.91 (s, 1H), 8.40 (d, *J* = 7.6 Hz, 1H), 8.34 (d, *J* = 8.8 Hz, 1H), 8.28 (d, *J* = 7.4 Hz, 1H), 8.20 (t, *J* = 5.7 Hz, 1H), 7.75 (t, *J* = 5.7 Hz, 1H), 7.70 (d, *J* = 2.9 Hz, 1H), 7.64 – 7.59 (m, 1H), 7.54 (t, *J* = 8.1 Hz, 3H), 7.45 – 7.33 (m, 10H), 7.32 – 7.20 (m, 8H), 6.01 – 5.87 (m, 1H), 5.35 (s, 2H), 5.15 – 4.92 (m, 6H), 4.88 – 4.76 (m, 1H), 4.51 – 4.27 (m, 4H), 4.22 (t, *J* = 7.6 Hz, 1H), 3.95 – 3.79 (m, 3H), 3.76 – 3.67 (m, 1H), 3.54 (d, *J* = 14.0 Hz, 1H), 3.44 (dt, *J* = 17.5, 6.4 Hz, 4H), 3.35 (t, *J* = 5.7 Hz, 2H), 3.18 (dd, *J* = 10.7, 5.6 Hz, 2H), 3.15 – 3.06 (m, 2H), 2.98 – 2.83 (m, 2H), 2.62 – 2.48 (m, 7H), 2.38 (s, 3H), 2.33 (s, 3H), 2.30 – 2.20 (m, 1H), 2.15 (dt, *J* = 15.2, 7.6 Hz, 1H), 2.00 – 1.84 (m, 3H), 1.77 – 1.67 (m, 3H), 1.65 – 1.58 (m, 1H), 1.58 – 1.47 (m, 4H), 1.43 – 1.21 (m, 6H), 0.88 (s, 9H), 0.79 (dd, *J* = 12.9, 6.7 Hz, 6H). ^{13}C NMR (126 MHz, DMSO-d_6) δ 171.7, 171.6, 171.4, 171.3, 170.3, 170.2, 169.8, 169.4, 169.0, 166.2, 163.6, 159.6, 155.8, 154.4, 152.3, 152.1, 150.5, 149.1, 148.4, 145.2, 144.6, 139.8, 137.4, 135.9, 133.1, 132.9, 131.7, 131.3, 130.8, 130.5, 130.3, 130.2, 129.9, 129.6, 129.5, 129.5, 129.1, 128.8, 128.6, 128.3, 127.4, 126.9, 125.8, 124.5, 123.2, 122.1, 119.6, 114.9, 108.8, 77.5, 70.0, 69.6, 68.6, 67.7, 58.7, 58.1, 56.6, 55.5, 54.5, 53.9, 52.0, 48.5, 39.3, 39.0, 38.1, 36.5, 36.0, 35.3, 31.6, 31.0, 30.3, 30.1, 29.8, 27.5, 26.8, 26.0, 23.1, 19.8, 18.6, 16.6, 14.7, 13.3, 11.9. HRMS (ESI) for $C_{93}H_{109}ClN_{19}O_{15}S_2$ ([M+H$^+$]): calcd 1830.7480, found 1830.7446. HPLC purity: 97.2% (254 nm).

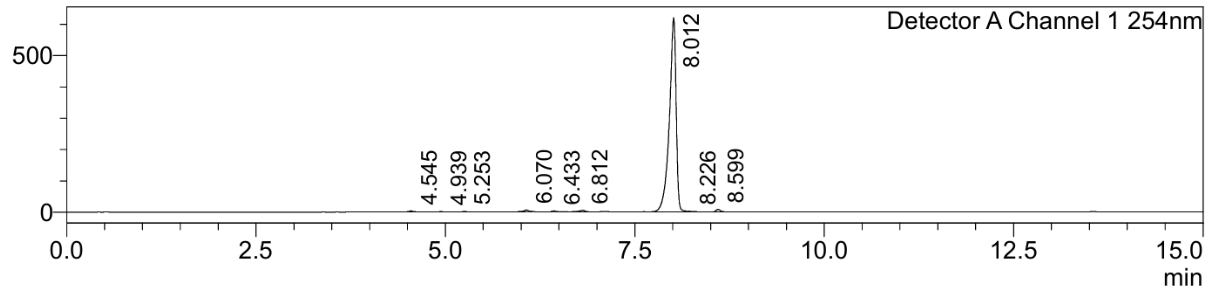

Detector A Channel 1 254nm

Peak#	Ret. Time	Height	Width at 5% Height	Area	Area%
1	4.545	3357	0.200	14342	0.361
2	4.939	990	0.194	3789	0.095
3	5.253	1525	0.159	4989	0.126
4	6.070	5274	0.170	23450	0.590
5	6.433	2117	--	3229	0.081
6	6.812	4703	0.203	25120	0.632
7	8.012	617672	0.233	3862161	97.178
8	8.226	1515	--	6536	0.164
9	8.599	7857	0.133	30707	0.773
Total		645009		3974323	100.000

NMR

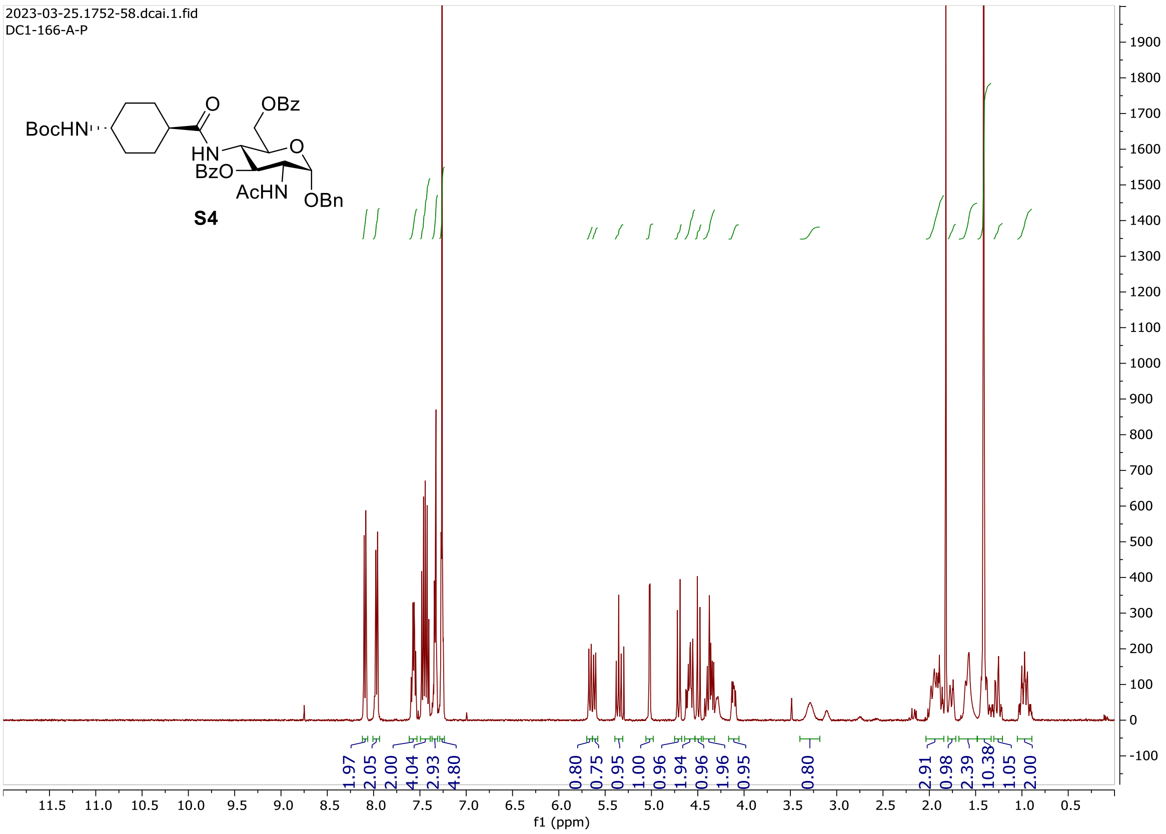

2023-03-25.1752-58.dcai.1.fid
DC1-166-A-P
BocHN
O
OBz
HN
O
BzO
AcHN
OBn
S4
1.97
2.05
2.00
4.04
2.93
4.80
0.80
0.75
0.95
1.00
0.96
1.94
0.96
1.96
0.95
0.80
2.91
0.98
2.39
10.38
1.05
2.00
f1 (ppm)

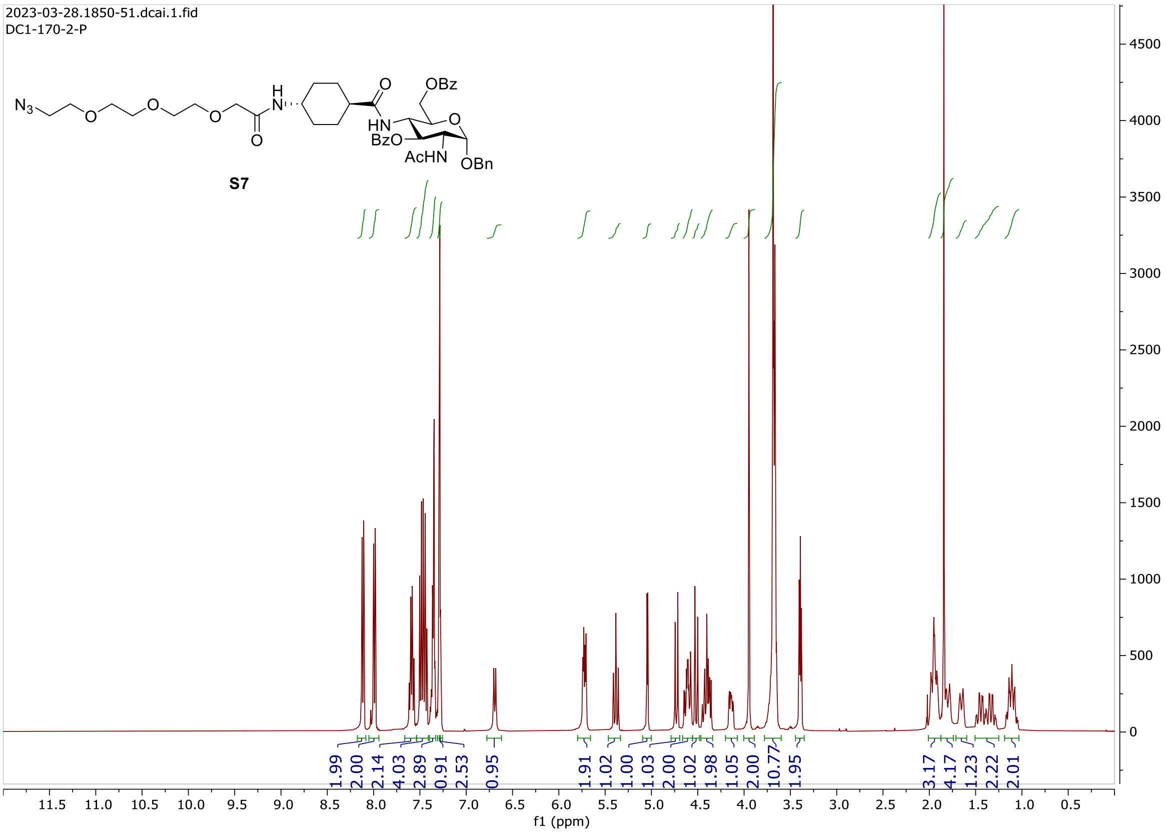
2023-03-28.1850-51.dcai.1.fid
DC1-170-2-P
S7
1.99
2.00
2.14
4.03
2.89
0.91
2.53
0.95
1.91
1.02
1.00
1.03
2.00
1.02
1.98
1.05
2.00
10.77
1.95
3.17
4.17
1.23
2.22
2.01
f1 (ppm)

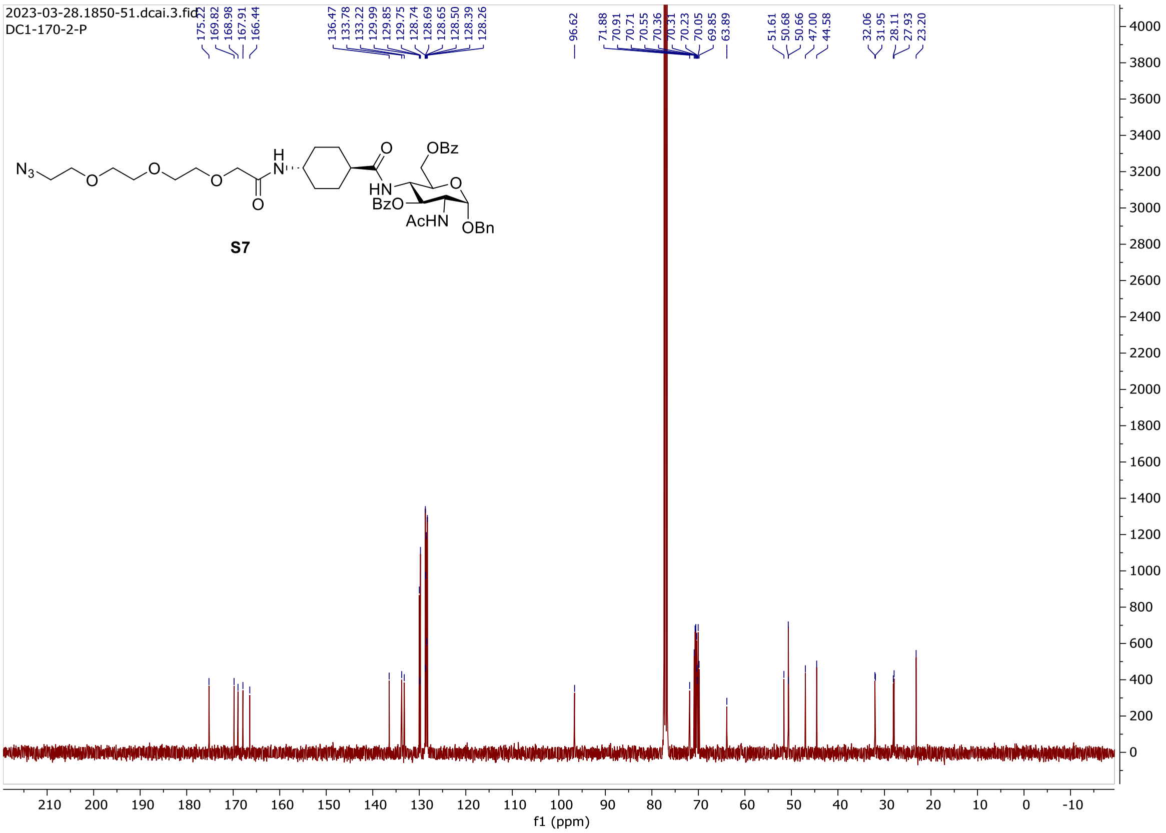
2023-03-28.1850-51.dcai.3.fid
DC1-170-2-P
175.22
169.82
168.98
167.91
166.44
136.47
133.78
133.22
129.99
129.85
129.75
128.74
128.69
128.65
128.50
128.39
128.26
96.62
71.88
70.91
70.71
70.55
70.36
70.31
70.23
70.05
69.85
63.89
51.61
50.68
50.66
47.00
44.58
32.06
31.95
28.11
27.93
23.20
S7
f1 (ppm)

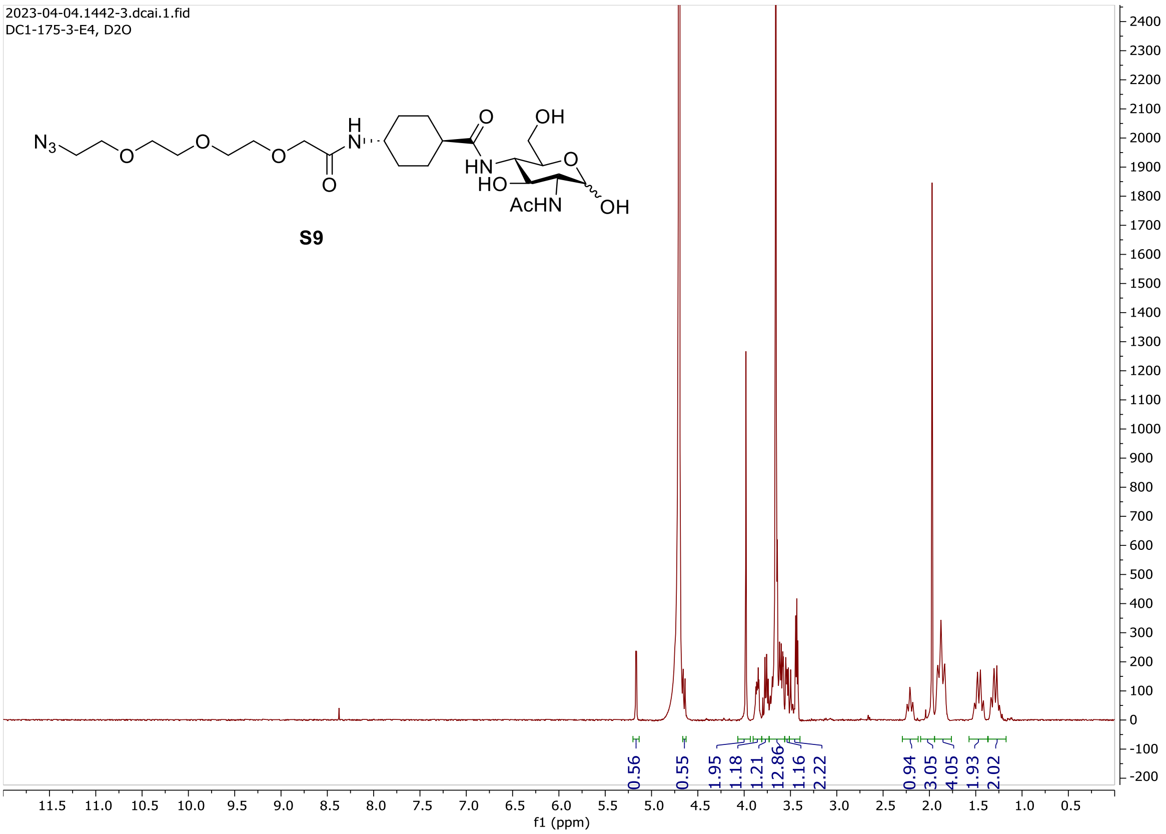

2023-04-04.1442-3.dcai.1.fid
DC1-175-3-E4, D2O
S9
0.56
0.55
1.95
1.18
1.21
12.86
1.16
2.22
0.94
3.05
4.05
1.93
2.02
f1 (ppm)

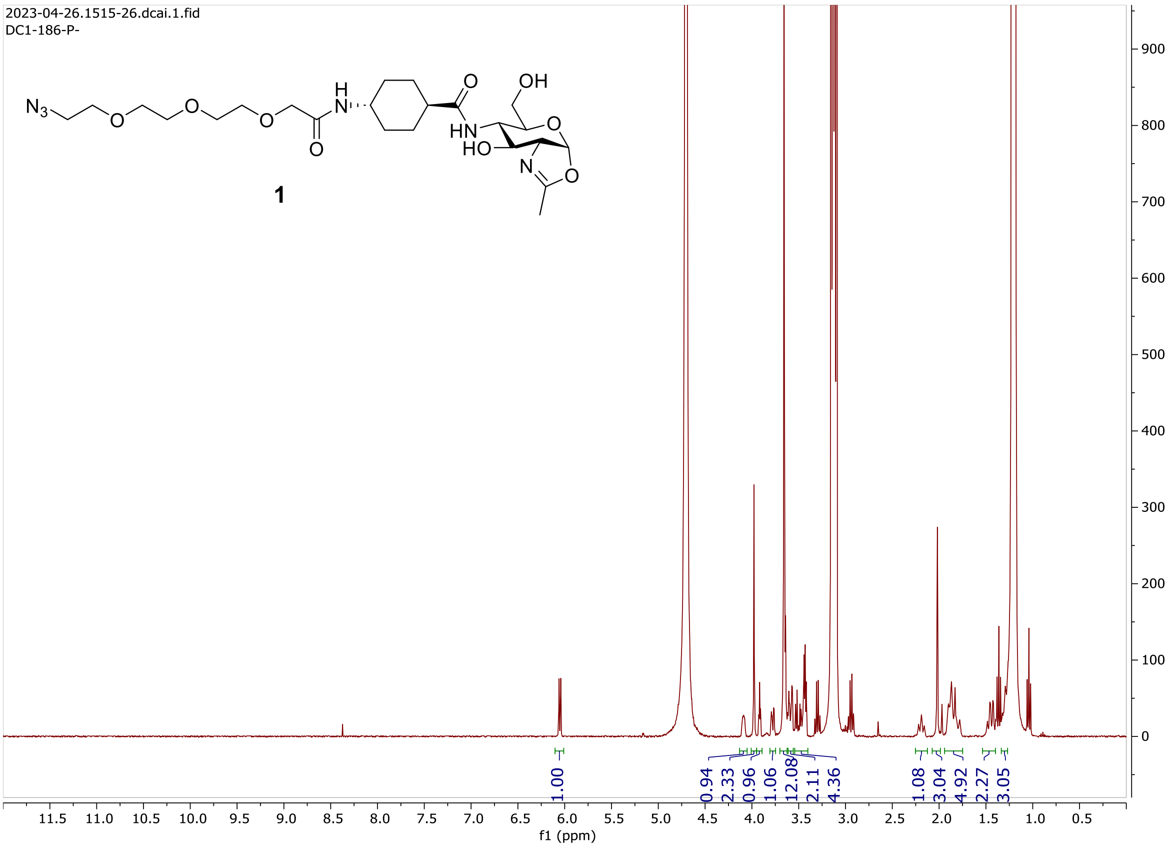

2023-04-26.1515-26.dcai.1.fid
DC1-186-P-
1
1.00
0.94
2.33
0.96
1.06
12.08
2.11
4.36
1.08
3.04
4.92
2.27
3.05
f1 (ppm)

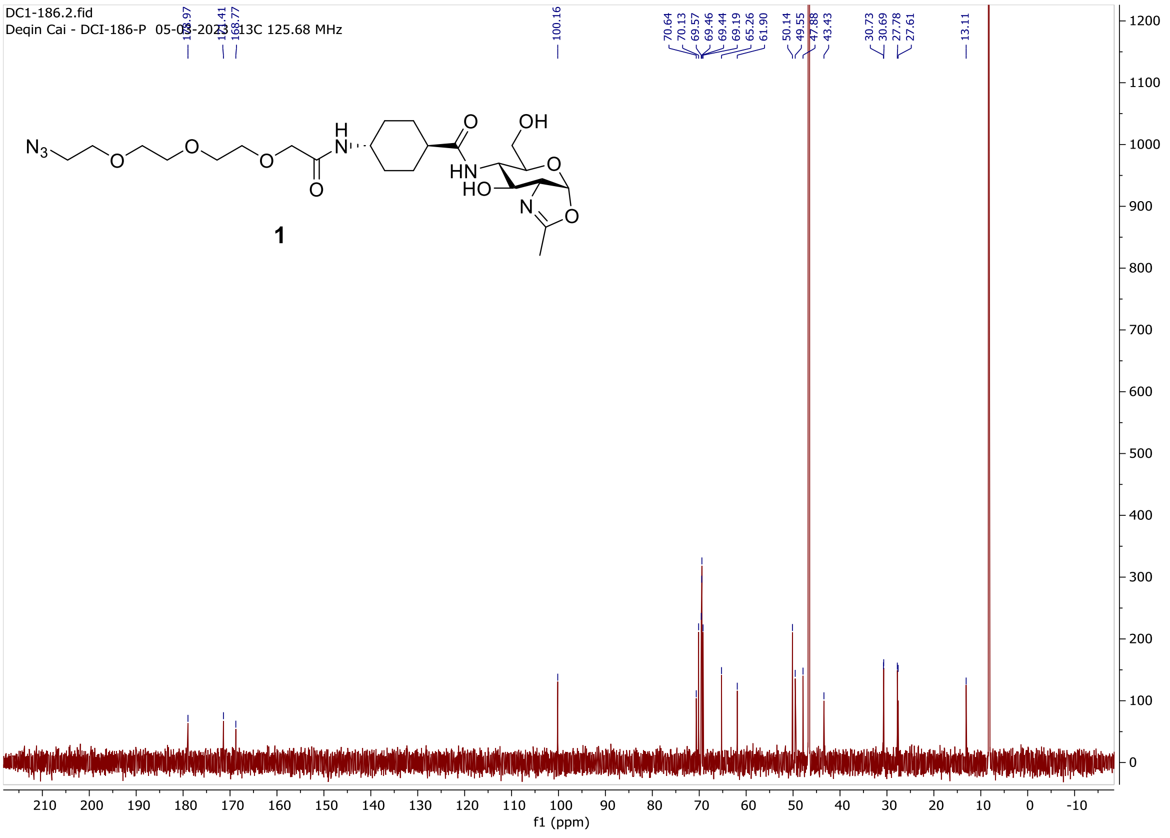

DC1-186.2.fid
Deqin Cai - DCI-186-P 05-03-2023 13C 125.68 MHz
100.16
70.64
70.13
69.57
69.46
69.44
69.19
65.26
61.90
50.14
49.55
47.88
43.43
30.73
30.69
27.78
27.61
13.11
1
f1 (ppm)

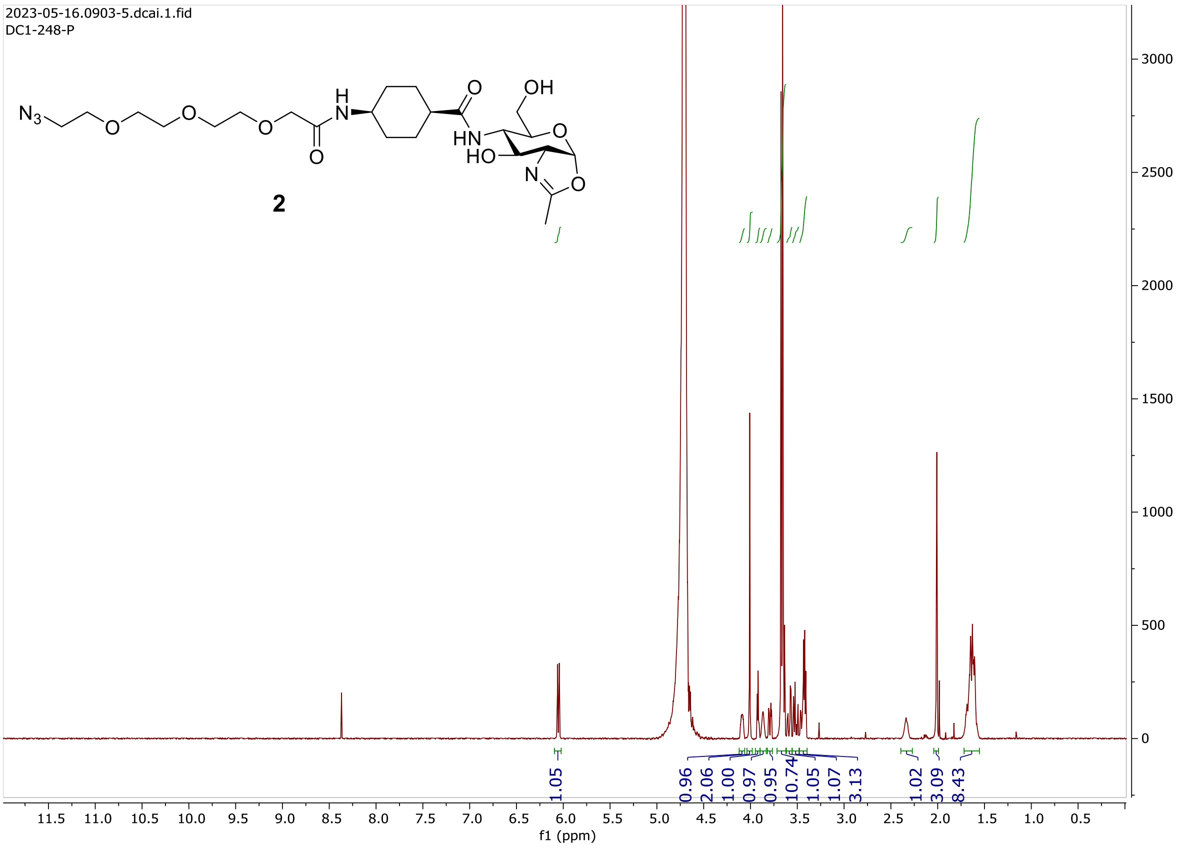
2023-05-16.0903-5.dcai.1.fid
DC1-248-P
2
f1 (ppm)

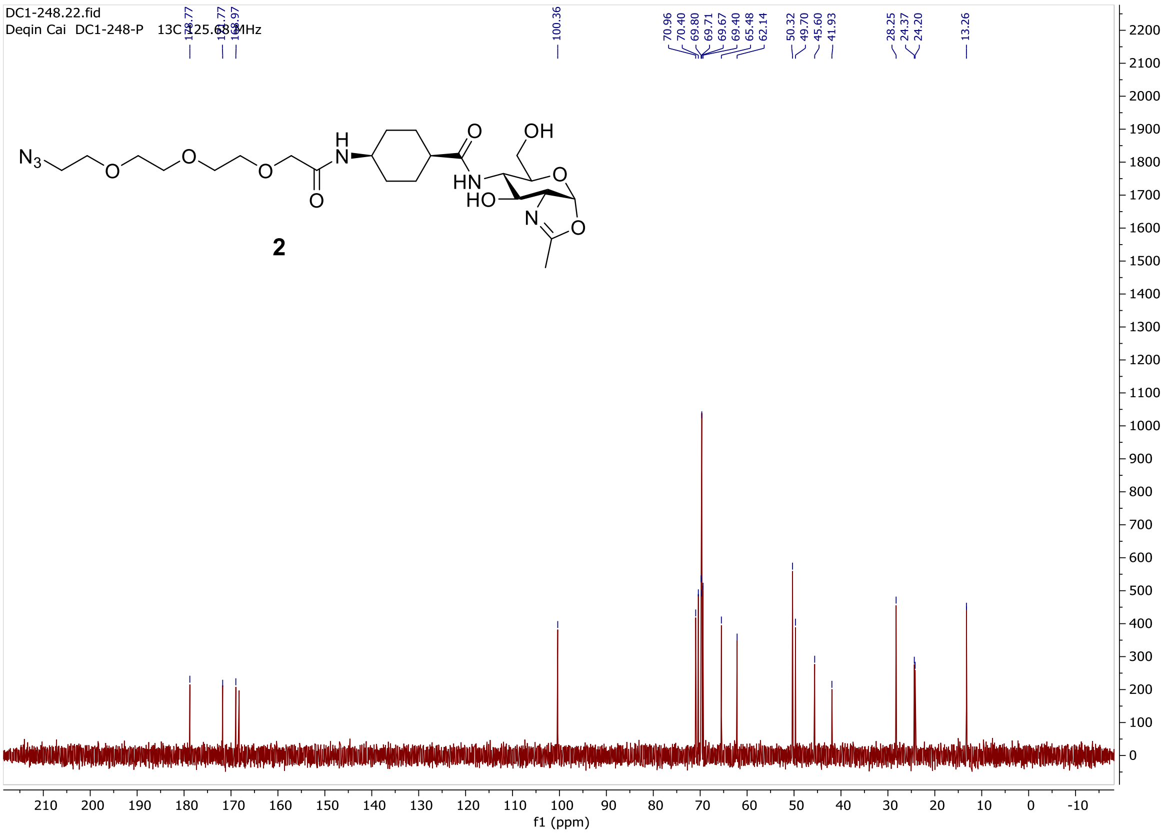
DC1-248.22.fid
Deqin Cai DC1-248-P 13C 125.68 MHz
100.36
70.96
70.40
69.80
69.71
69.67
69.40
65.48
62.14
50.32
49.70
45.60
41.93
28.25
24.37
24.20
13.26
2
f1 (ppm)

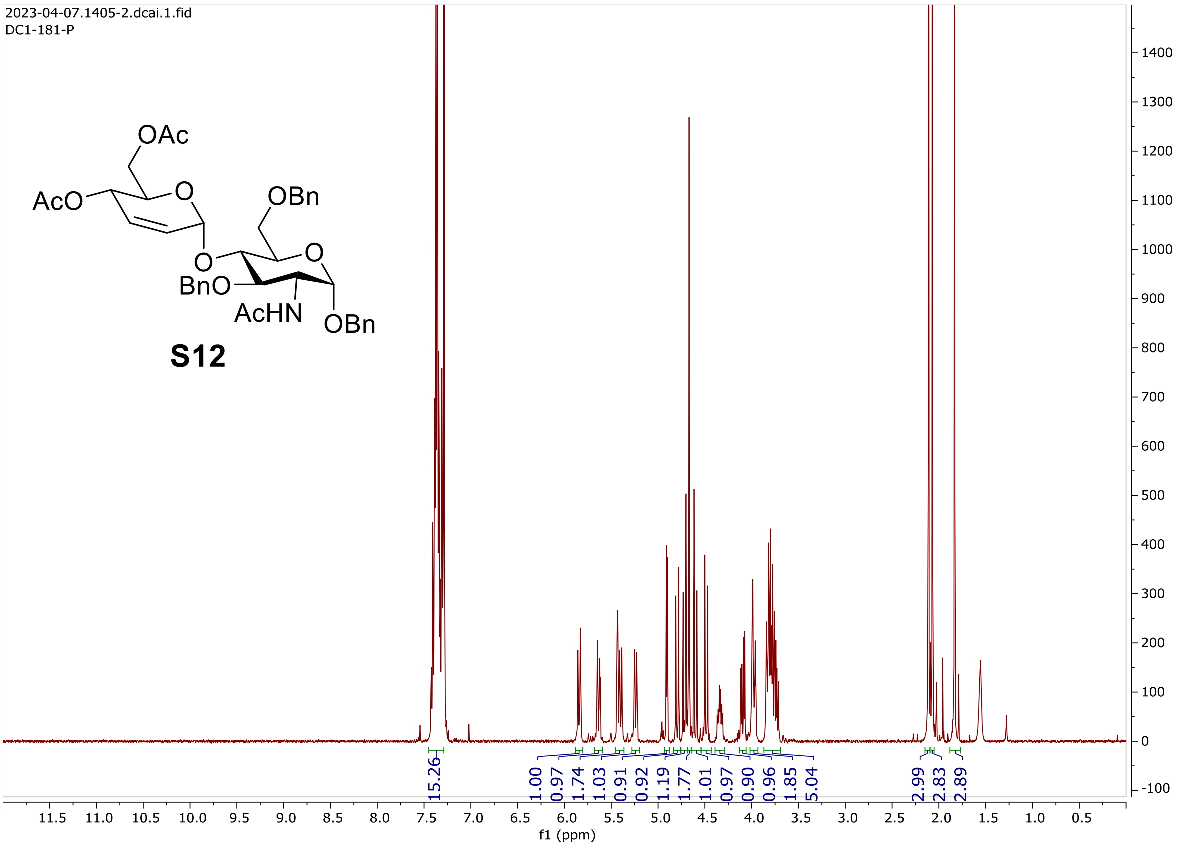
2023-04-07.1405-2.dcai.1.fid
DC1-181-P
OAc
AcO
O
OBn
O
O
BnO
AcHN
OBn
S12
15.26
1.00
0.97
1.74
1.03
0.91
0.92
1.19
1.77
1.01
0.97
0.90
0.96
1.85
5.04
2.99
2.83
2.89
f1 (ppm)

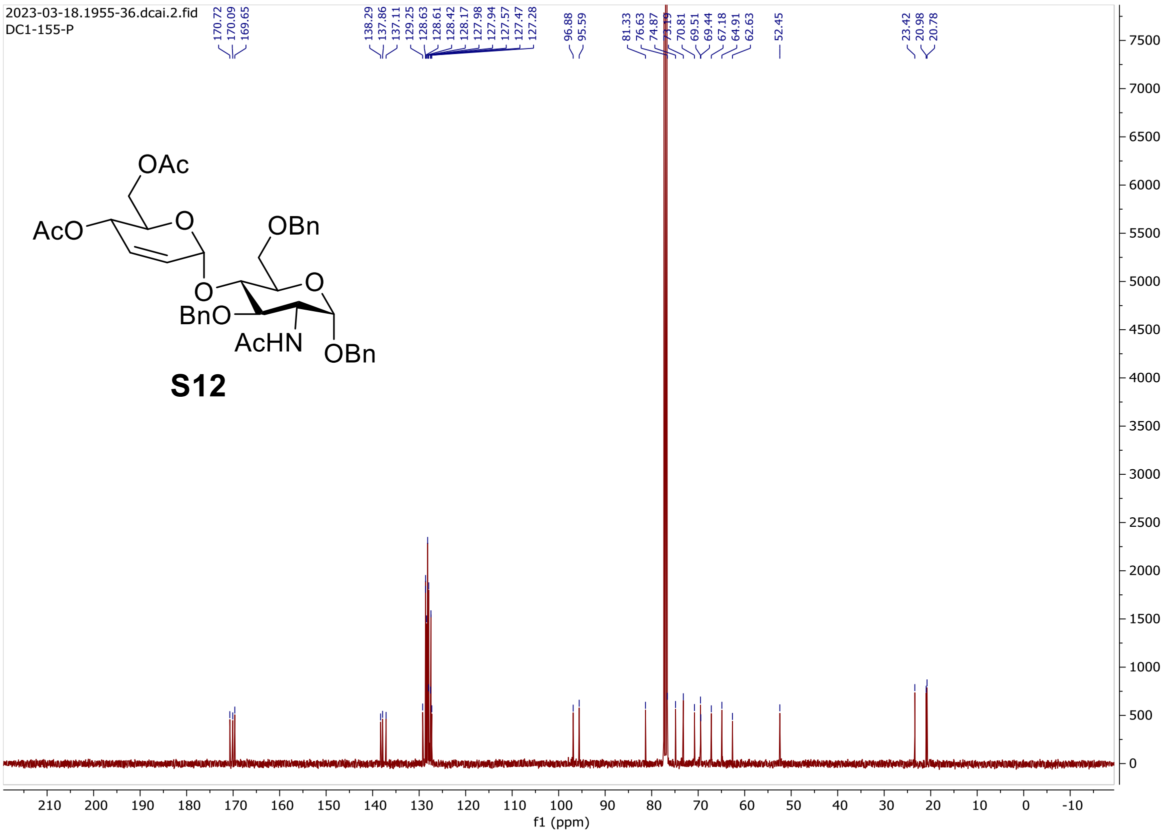
2023-03-18.1955-36.dcai.2.fid
DC1-155-P
170.72
170.09
169.65
138.29
137.86
137.11
129.25
128.63
128.61
128.42
128.17
127.98
127.94
127.57
127.47
127.28
96.88
95.59
81.33
76.63
74.87
73.19
70.81
69.51
69.44
67.18
64.91
62.63
52.45
23.42
20.98
20.78
OAc
AcO
O
OBn
O
O
BnO
AcHN
OBn
S12
f1 (ppm)

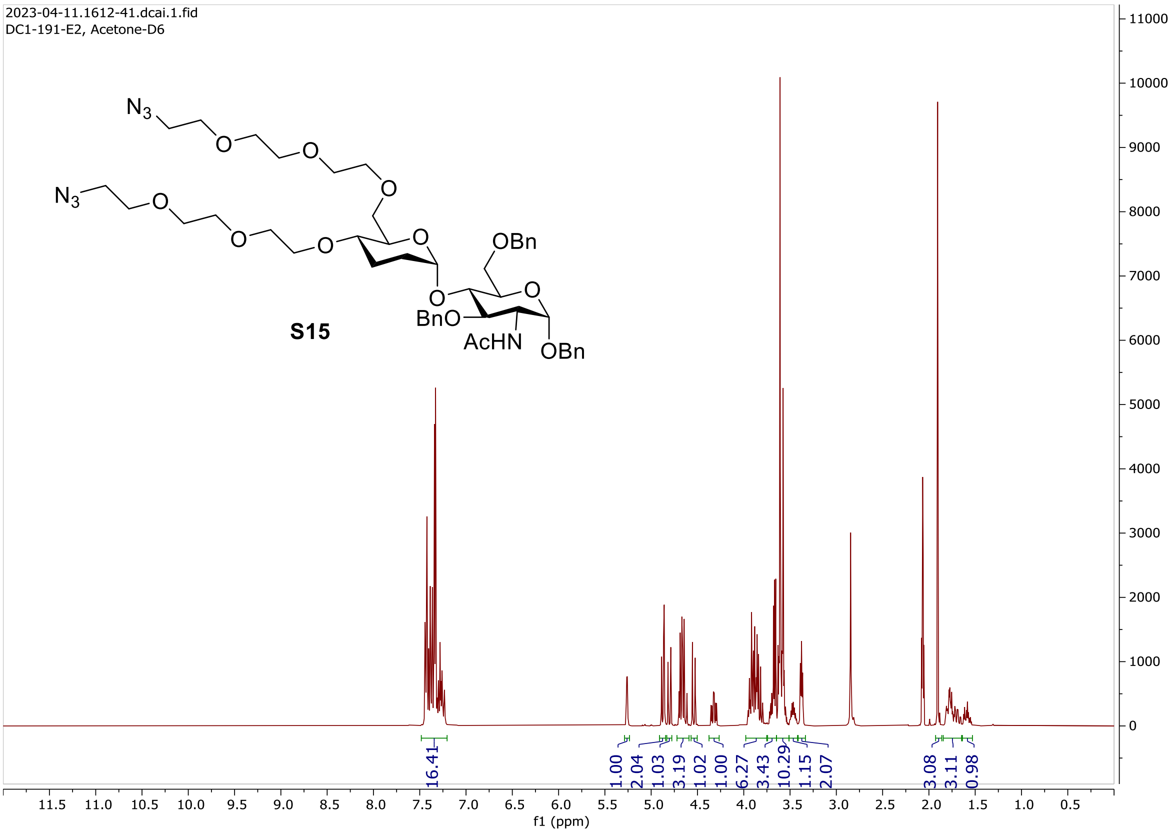

2023-04-11.1612-41.dcai.1.fid
DC1-191-E2, Acetone-D6
S15
16.41
1.00
2.04
1.03
3.19
1.02
1.00
6.27
3.43
10.29
1.15
2.07
3.08
3.11
0.98
f1 (ppm)

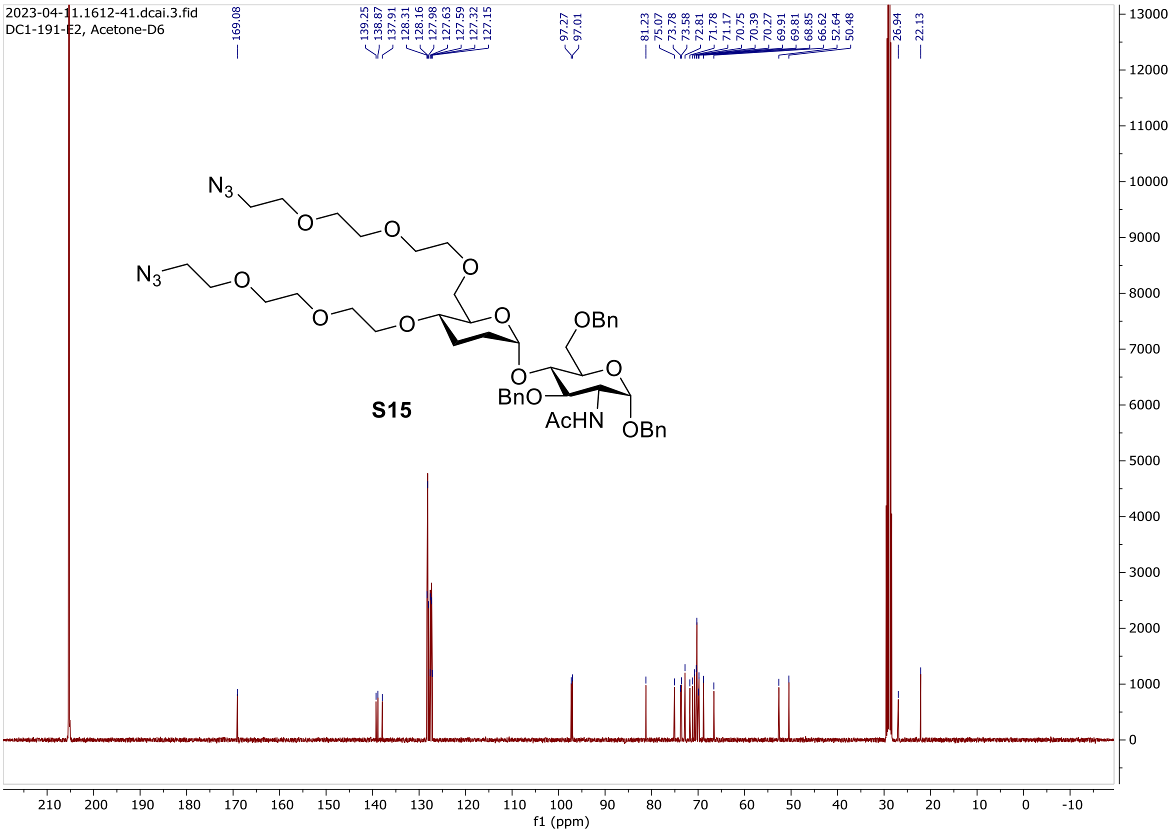

2023-04-11.1612-41.dcai.3.fid
DC1-191-E2, Acetone-D6
169.08
139.25
138.87
137.91
128.31
128.16
127.98
127.63
127.59
127.32
127.15
97.27
97.01
81.23
75.07
73.78
73.58
72.81
71.78
71.17
70.75
70.39
70.27
69.91
69.81
68.85
66.62
52.64
50.48
26.94
22.13
S15
f1 (ppm)

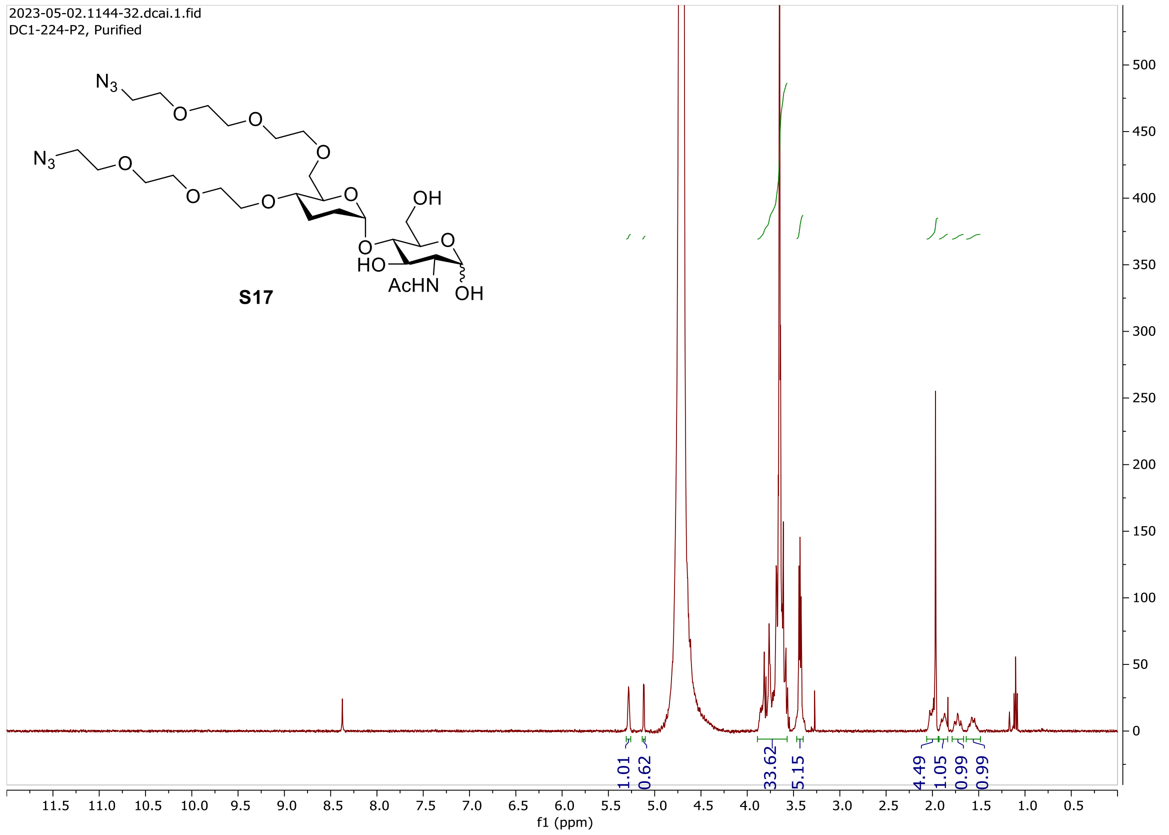

2023-05-02.1144-32.dcai.1.fid
DC1-224-P2, Purified
S17
1.01
0.62
33.62
5.15
4.49
1.05
0.99
0.99
f1 (ppm)

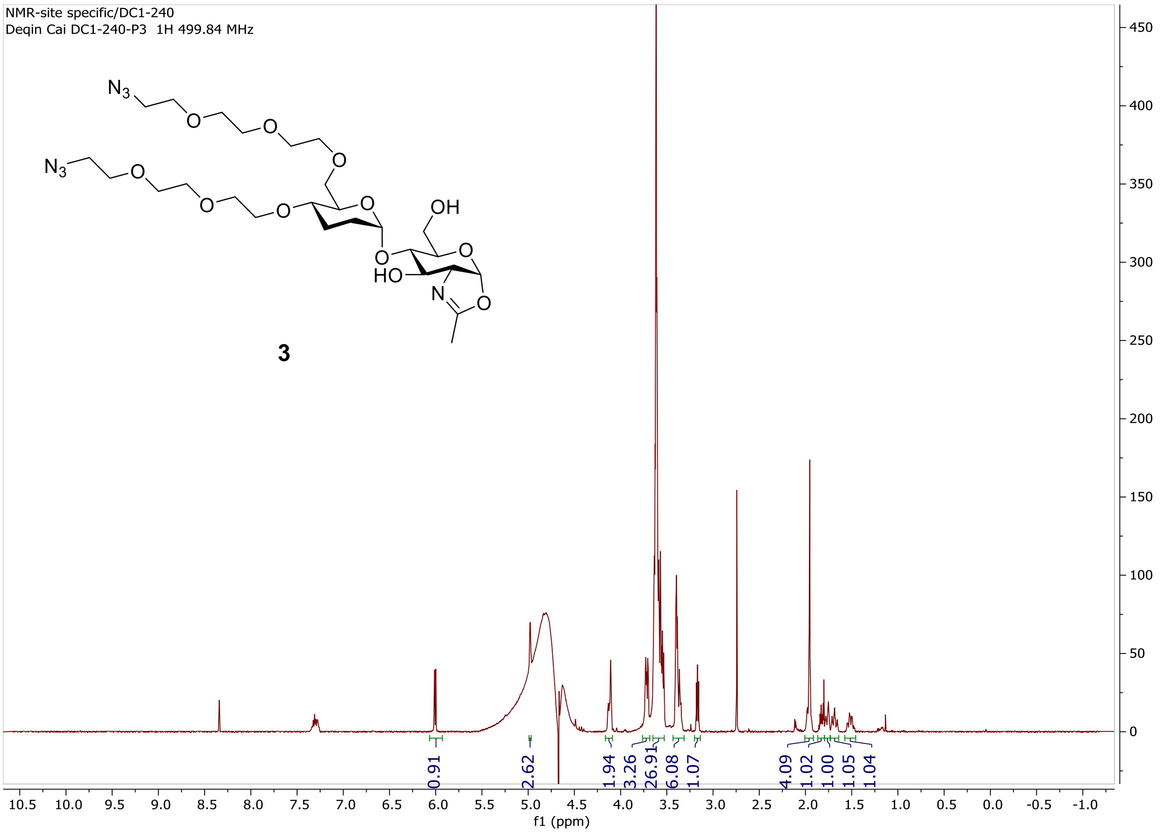
NMR-site specific/DC1-240
Deqin Cai DC1-240-P3 1H 499.84 MHz
N3
O
O
O
N3
O
O
O
O
OH
O
O
HO
N
O
3
0.91
2.62
1.94
3.26
26.91
6.08
1.07
4.09
1.02
1.00
1.05
1.04
f1 (ppm)
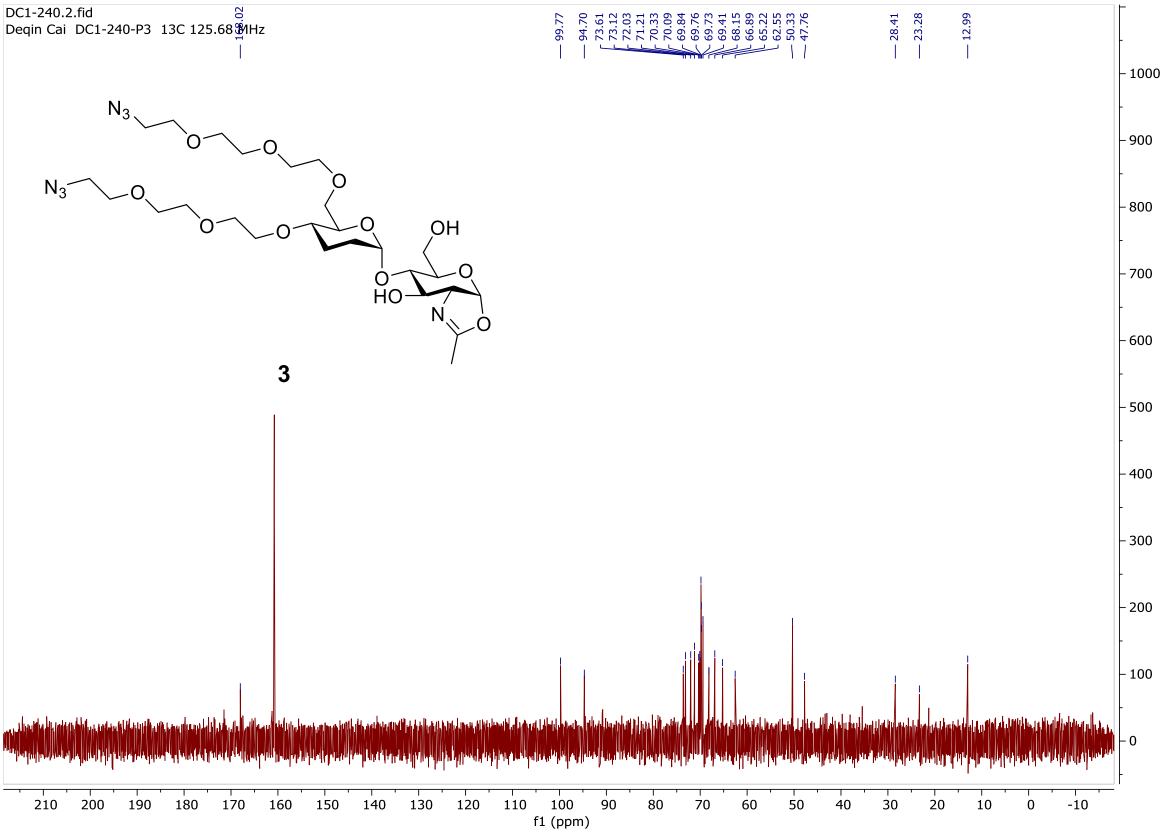
DC1-240.2.fid
Deqin Cai DC1-240-P3 13C 125.68 MHz
99.77
94.70
73.61
73.12
72.03
71.21
70.33
70.09
69.84
69.76
69.73
69.41
68.15
66.89
65.22
62.55
50.33
47.76
28.41
23.28
12.99
3
f1 (ppm)

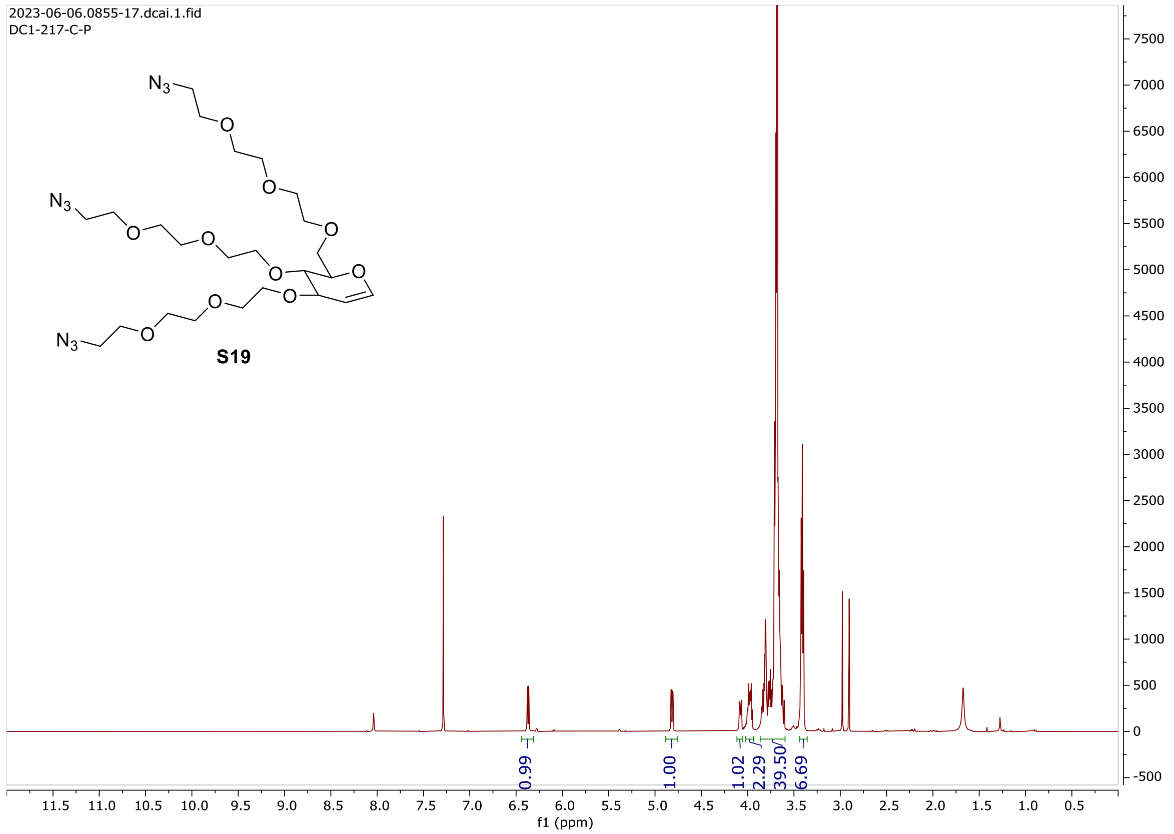

2023-06-06.0855-17.dcai.1.fid
DC1-217-C-P
S19
0.99
1.00
1.02
2.29
39.50
6.69
f1 (ppm)

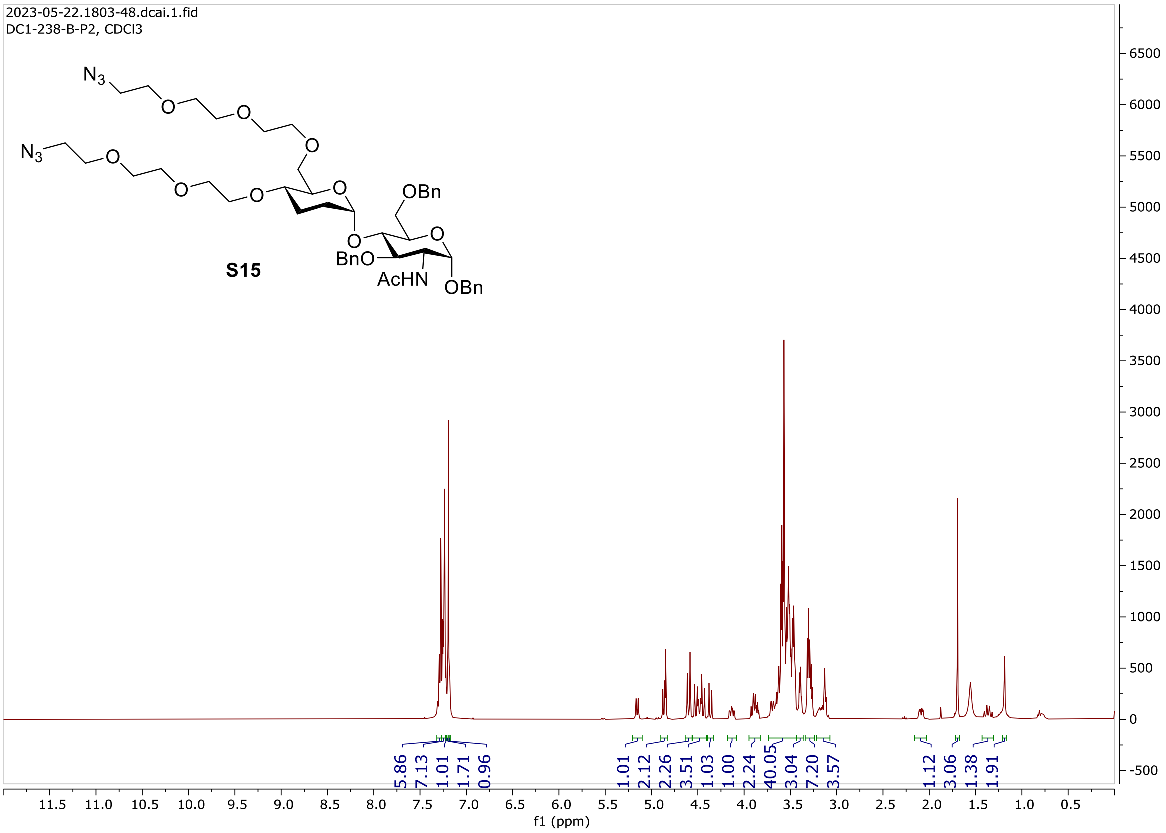
2023-05-22.1803-48.dcai.1.fid
DC1-238-B-P2, CDCl3
N3
N3
OBn
BnO
AcHN
OBn
S15
5.86
7.13
1.01
1.71
0.96
1.01
2.12
2.26
3.51
1.03
1.00
2.24
40.05
3.04
7.20
3.57
1.12
3.06
1.38
1.91
f1 (ppm)

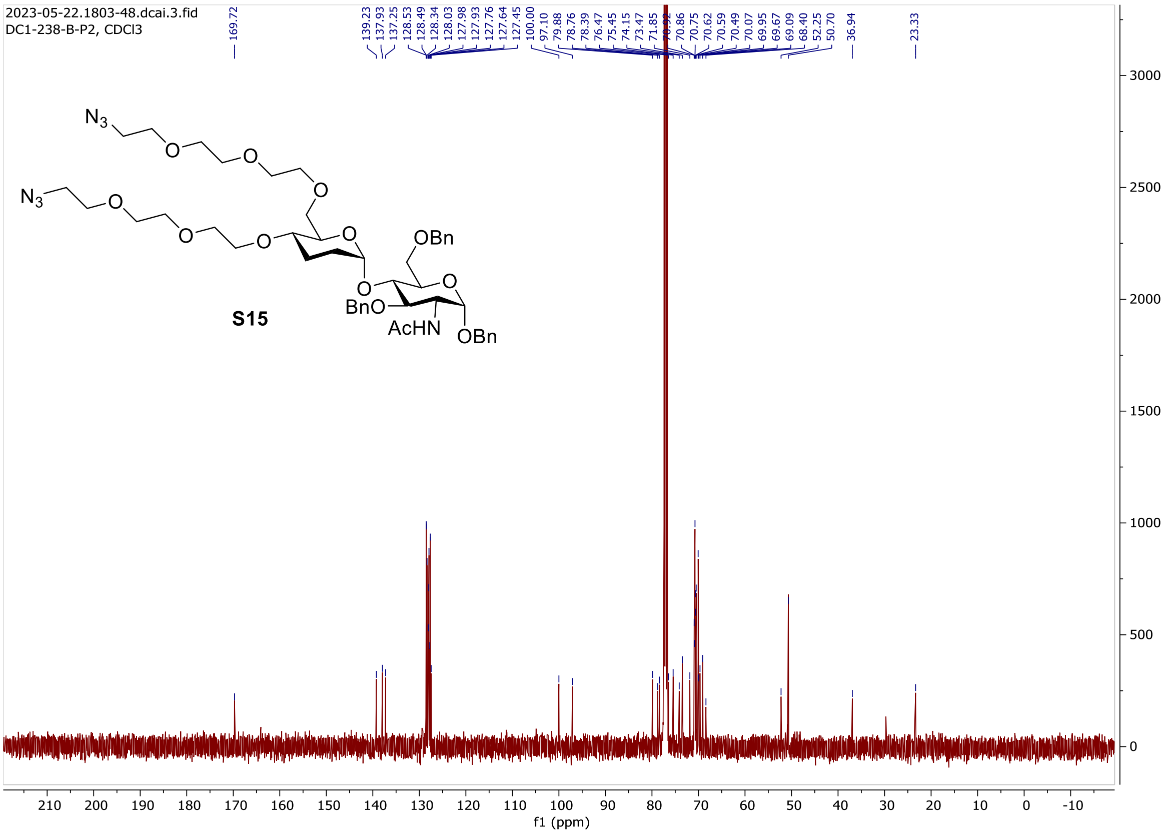
2023-05-22.1803-48.dcai.3.fid
DC1-238-B-P2, CDCl3
169.72
139.23
137.93
137.25
128.53
128.49
128.34
128.03
127.98
127.93
127.76
127.64
127.45
100.00
97.10
79.88
78.76
78.39
76.47
75.45
74.15
73.47
71.85
70.86
70.75
70.62
70.59
70.49
70.07
69.95
69.67
69.09
68.40
52.25
50.70
36.94
23.33
N3
N3
OBn
BnO
AcHN
OBn
S15
f1 (ppm)

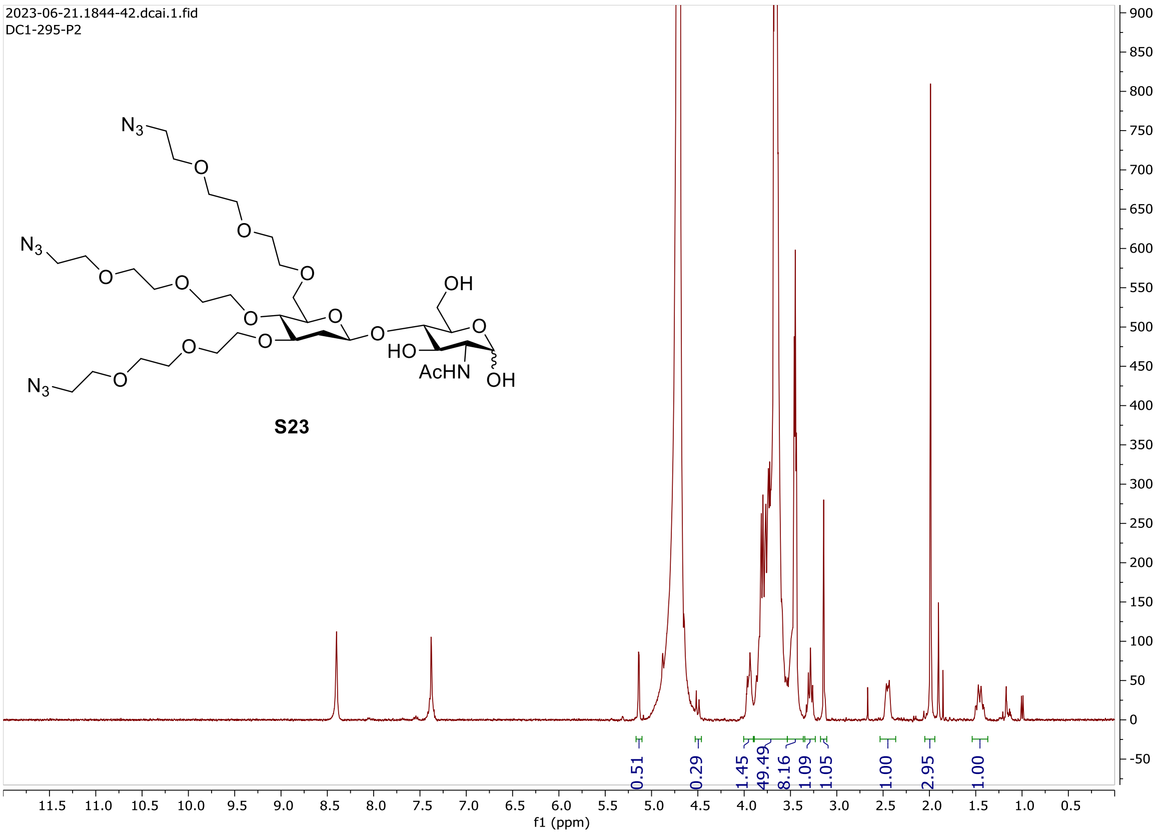
2023-06-21.1844-42.dcai.1.fid
DC1-295-P2
S23
f1 (ppm)

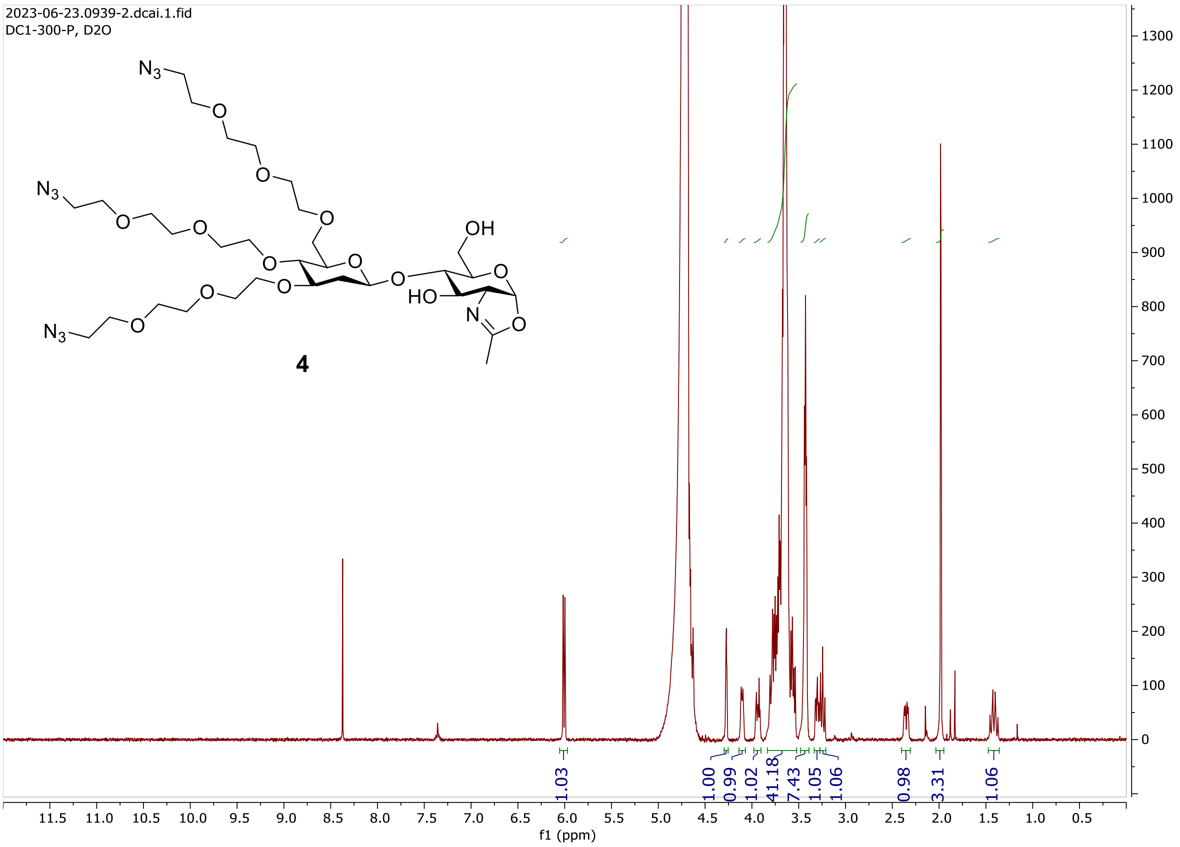
2023-06-23.0939-2.dcai.1.fid
DC1-300-P, D2O
4
1.03
1.00
0.99
1.02
41.18
7.43
1.05
1.06
0.98
3.31
1.06
f1 (ppm)

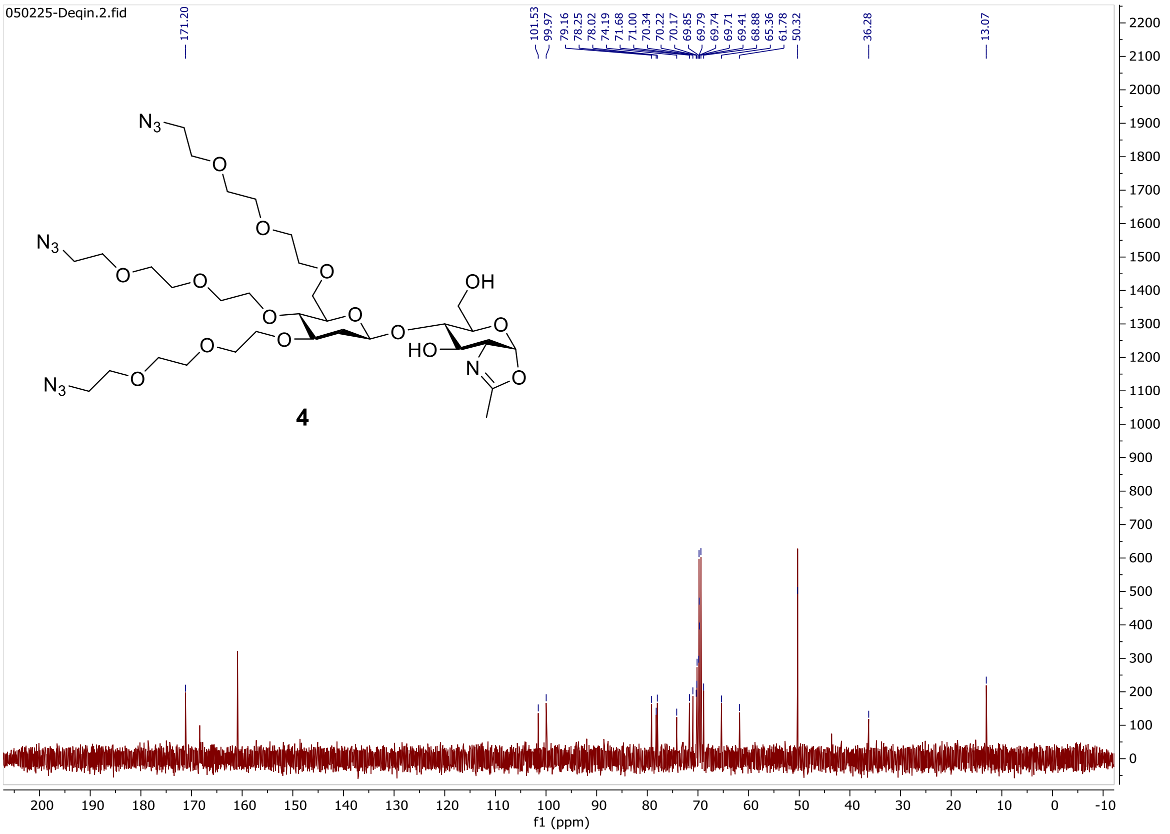
050225-Deqin.2.fid
171.20
101.53
99.97
79.16
78.25
78.02
74.19
71.68
71.00
70.34
70.22
70.17
69.85
69.79
69.74
69.71
69.41
68.88
65.36
61.78
50.32
36.28
13.07
4
f1 (ppm)

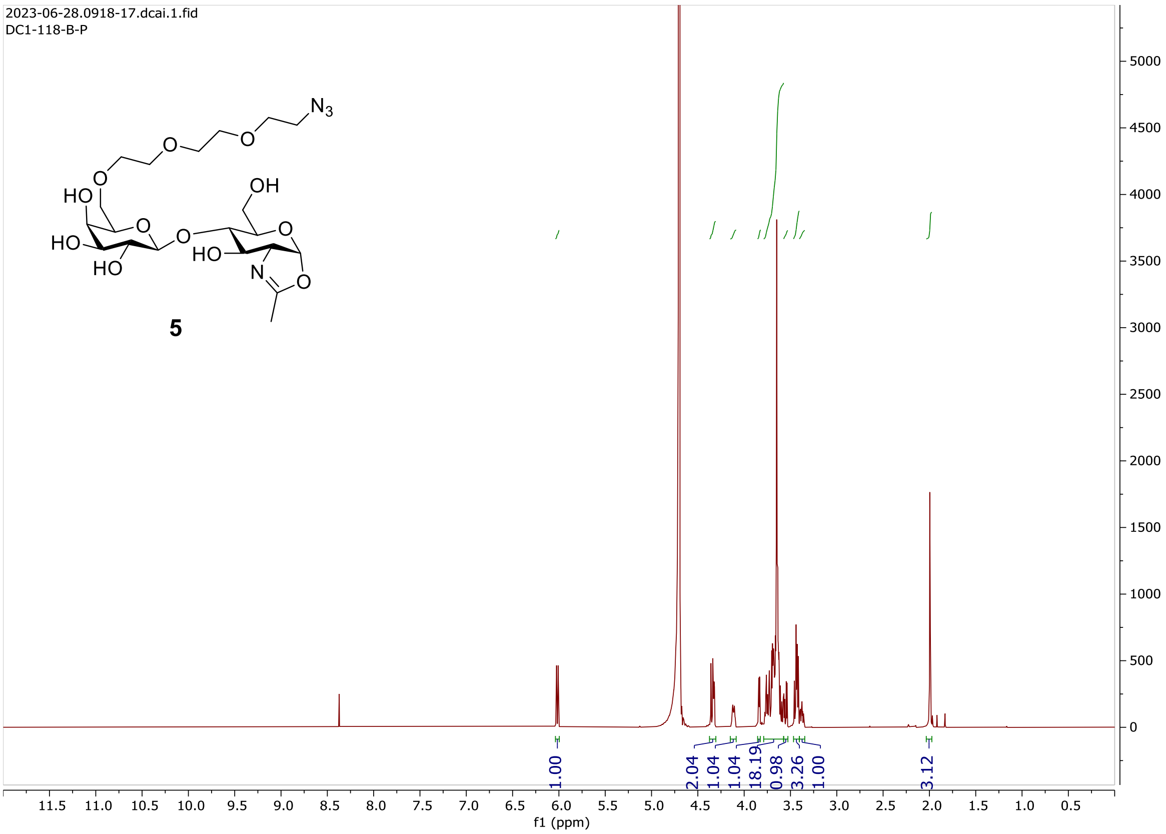
2023-06-28.0918-17.dcai.1.fid
DC1-118-B-P
5
1.00
2.04
1.04
1.04
18.19
0.98
3.26
1.00
3.12
f1 (ppm)

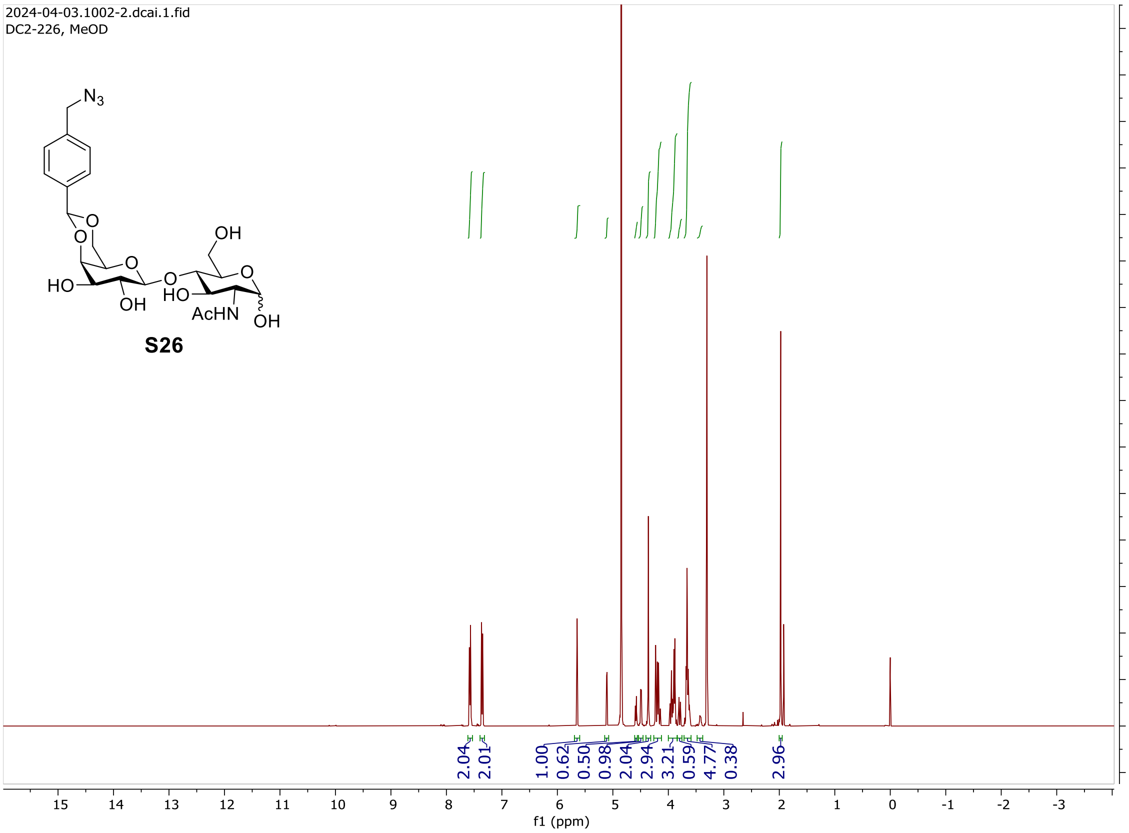
2024-04-03.1002-2.dcai.1.fid
DC2-226, MeOD
N3
OH
O
O
O
O
O
HO
OH
HO
AcHN
OH
S26
2.04
2.01
1.00
0.62
0.50
0.98
2.04
2.94
3.21
0.59
4.77
0.38
2.96
f1 (ppm)

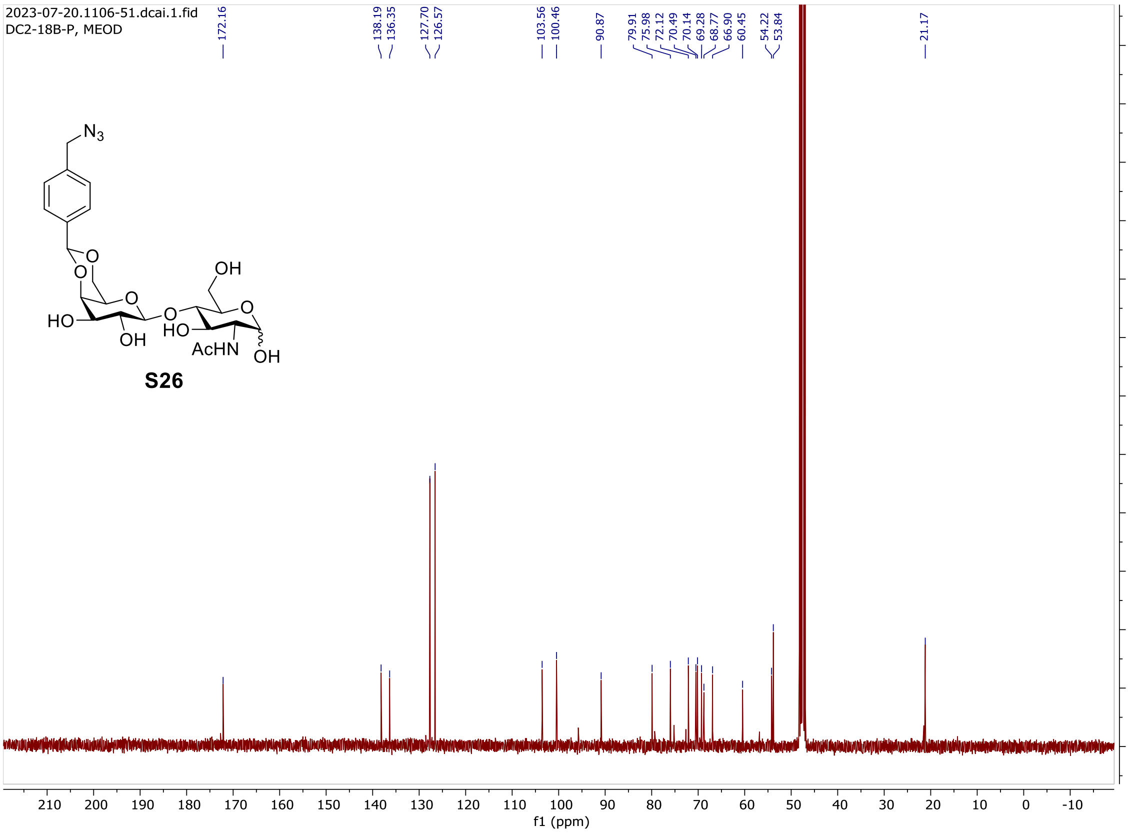
2023-07-20.1106-51.dcai.1.fid
DC2-18B-P, MEOD
172.16
138.19
136.35
127.70
126.57
103.56
100.46
90.87
79.91
75.98
72.12
70.49
70.14
69.28
68.77
66.90
60.45
54.22
53.84
21.17
N3
OH
O
O
O
O
O
HO
OH
HO
AcHN
OH
S26
f1 (ppm)

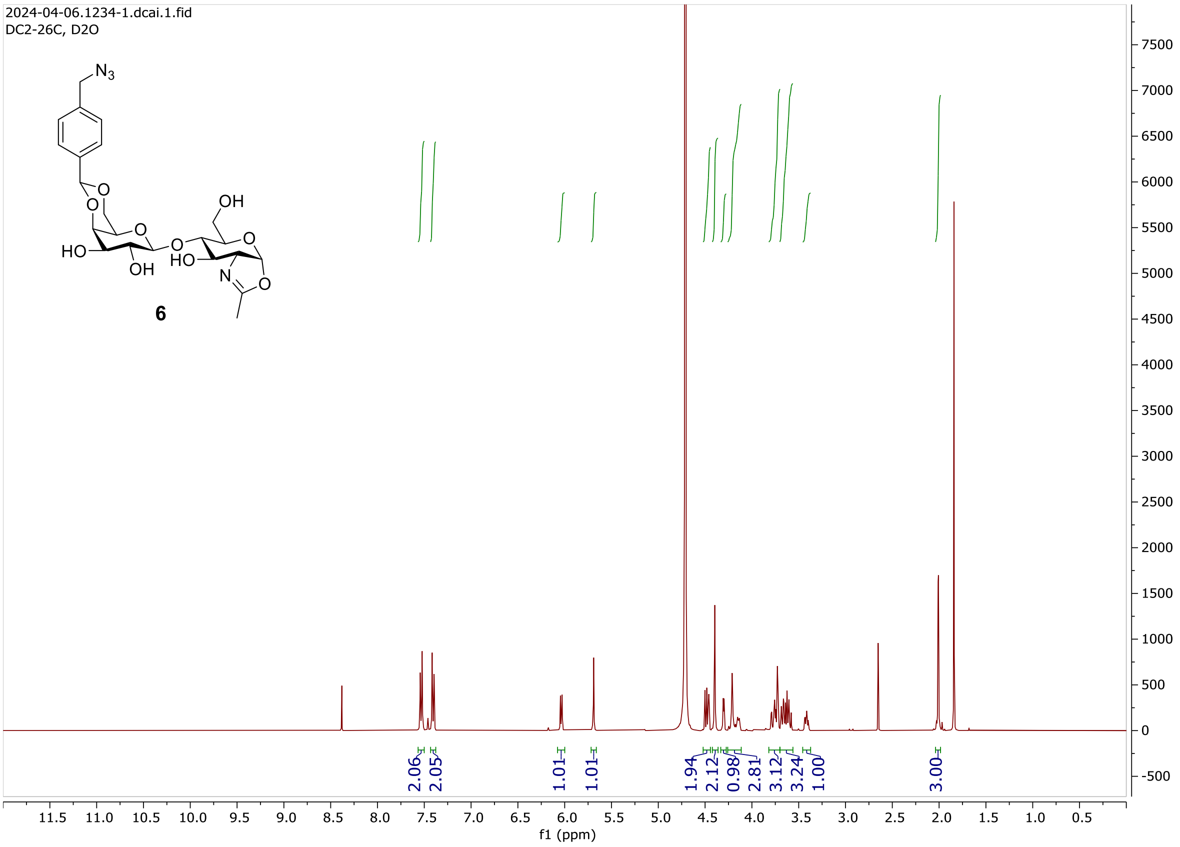
2024-04-06.1234-1.dcai.1.fid
DC2-26C, D2O
6
2.06
2.05
1.01
1.01
1.94
2.12
0.98
2.81
3.12
3.24
1.00
3.00
f1 (ppm)

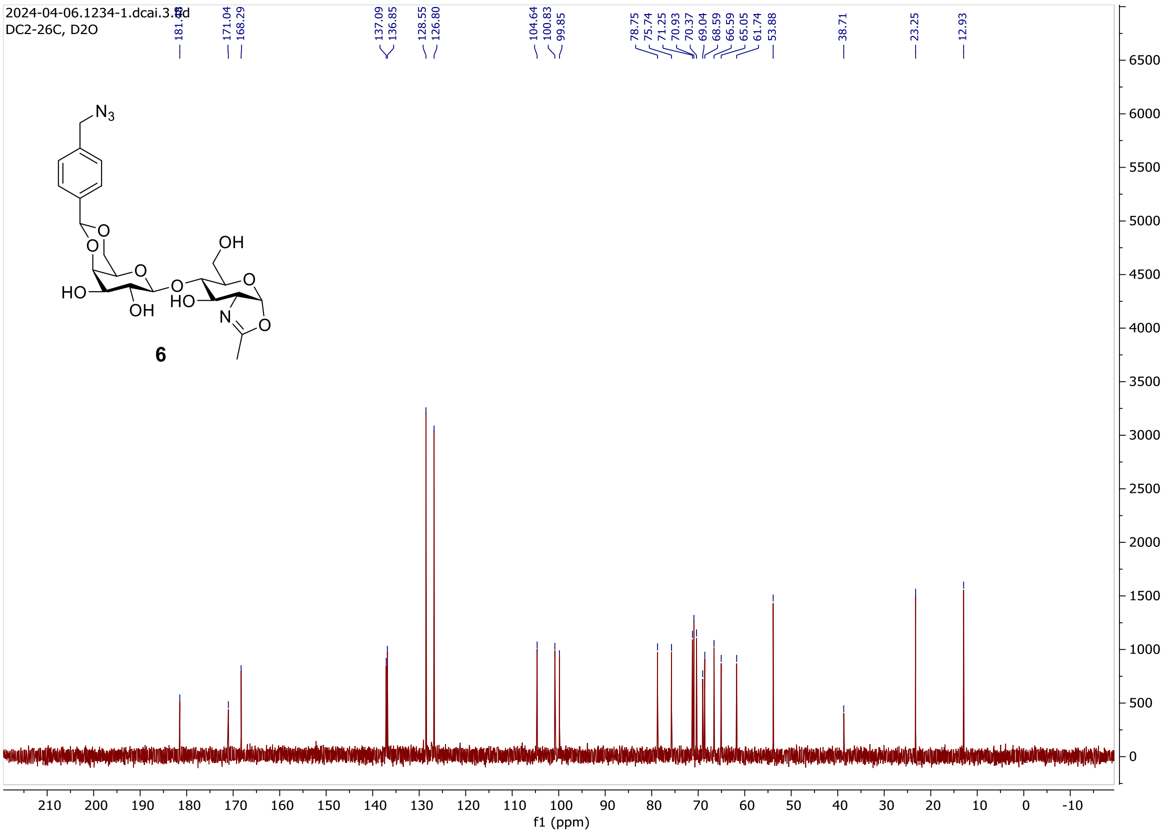
2024-04-06.1234-1.dcai.3.fid
DC2-26C, D2O
181.
171.04
168.29
137.09
136.85
128.55
126.80
104.64
100.83
99.85
78.75
75.74
71.25
70.93
70.37
69.04
68.59
66.59
65.05
61.74
53.88
38.71
23.25
12.93
6
f1 (ppm)

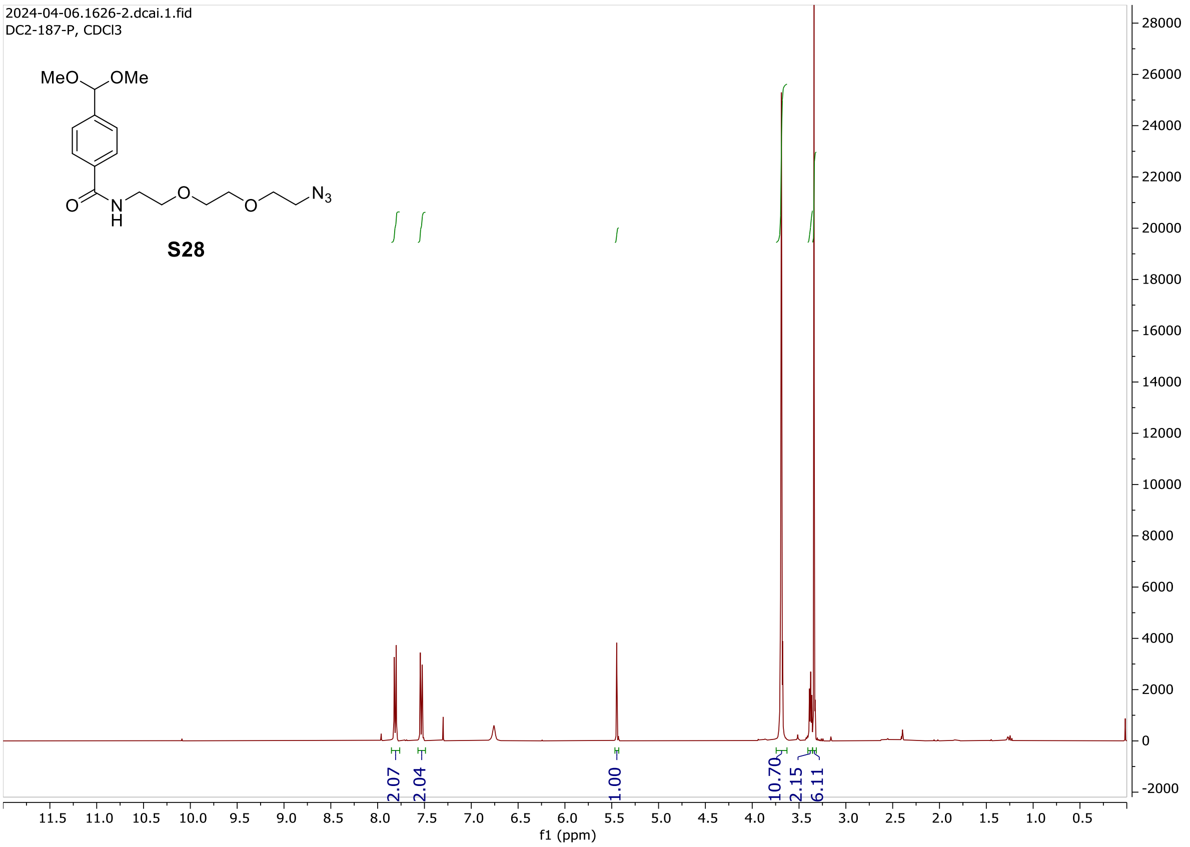
2024-04-06.1626-2.dcai.1.fid
DC2-187-P, CDCl3
MeO
OMe
O
N
H
O
O
N3
S28
2.07
2.04
1.00
10.70
2.15
6.11
f1 (ppm)

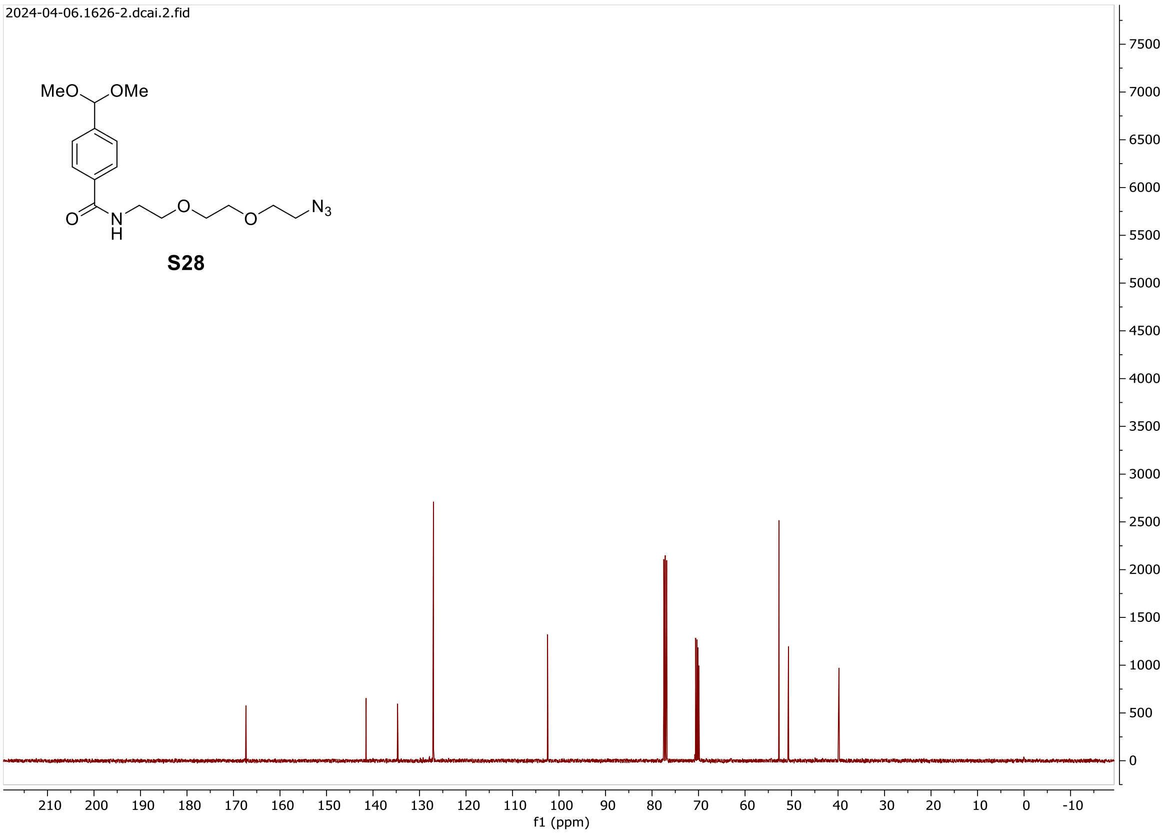
2024-04-06.1626-2.dcai.2.fid
MeO
OMe
O
N
H
O
O
N3
S28
f1 (ppm)

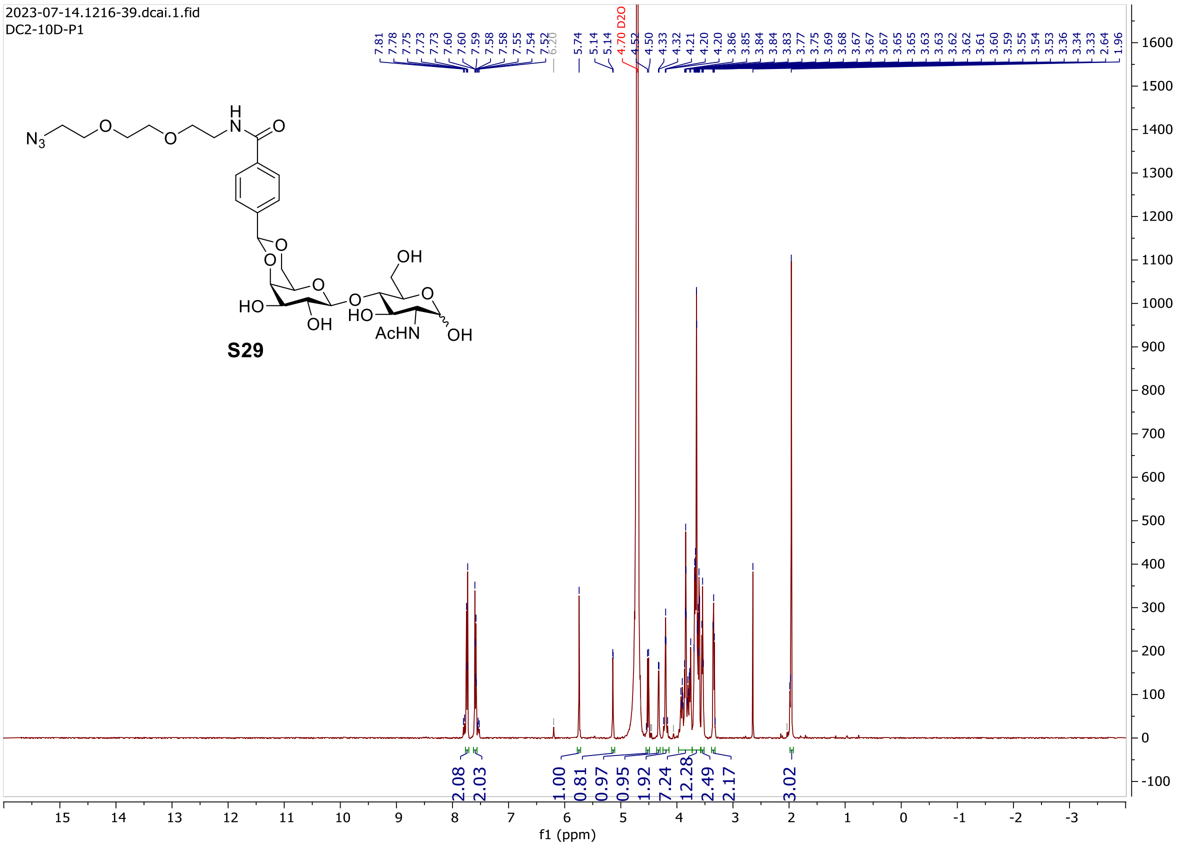
2023-07-14.1216-39.dcai.1.fid
DC2-10D-P1
S29
f1 (ppm)
4.70 D2O

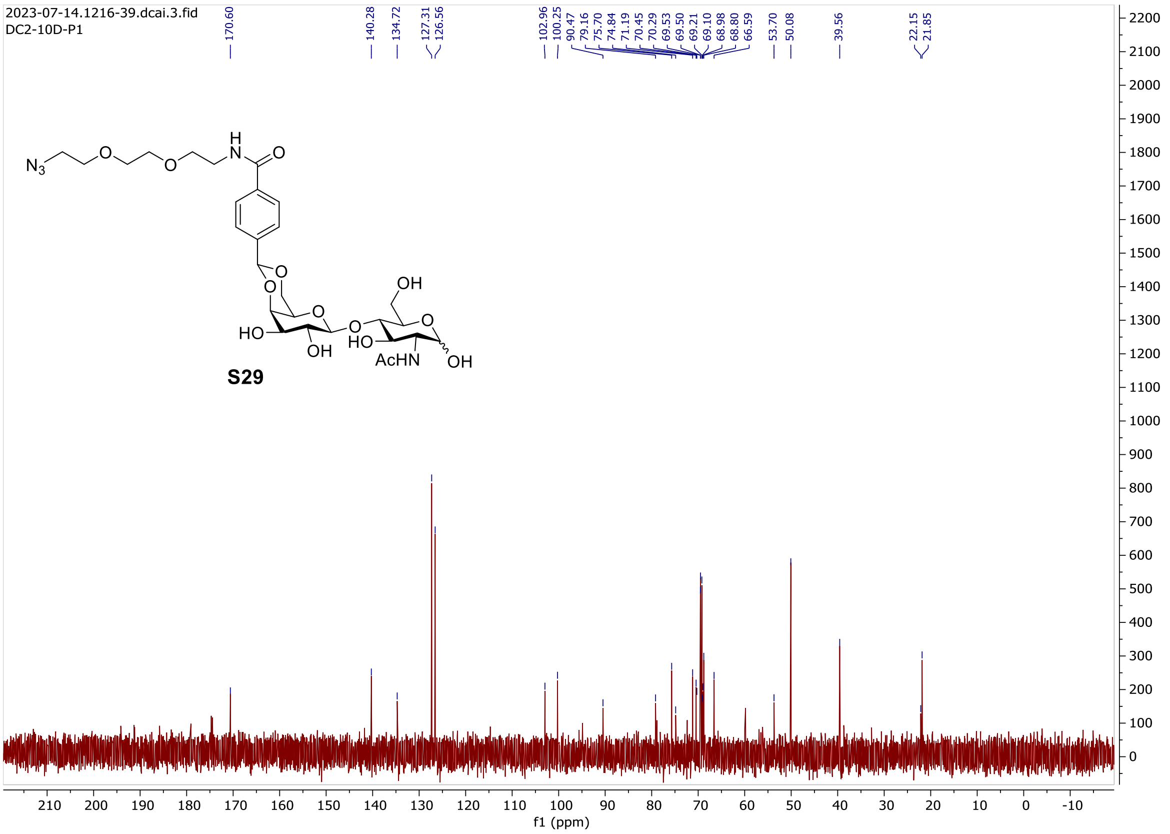
2023-07-14.1216-39.dcai.3.fid
DC2-10D-P1
S29
f1 (ppm)

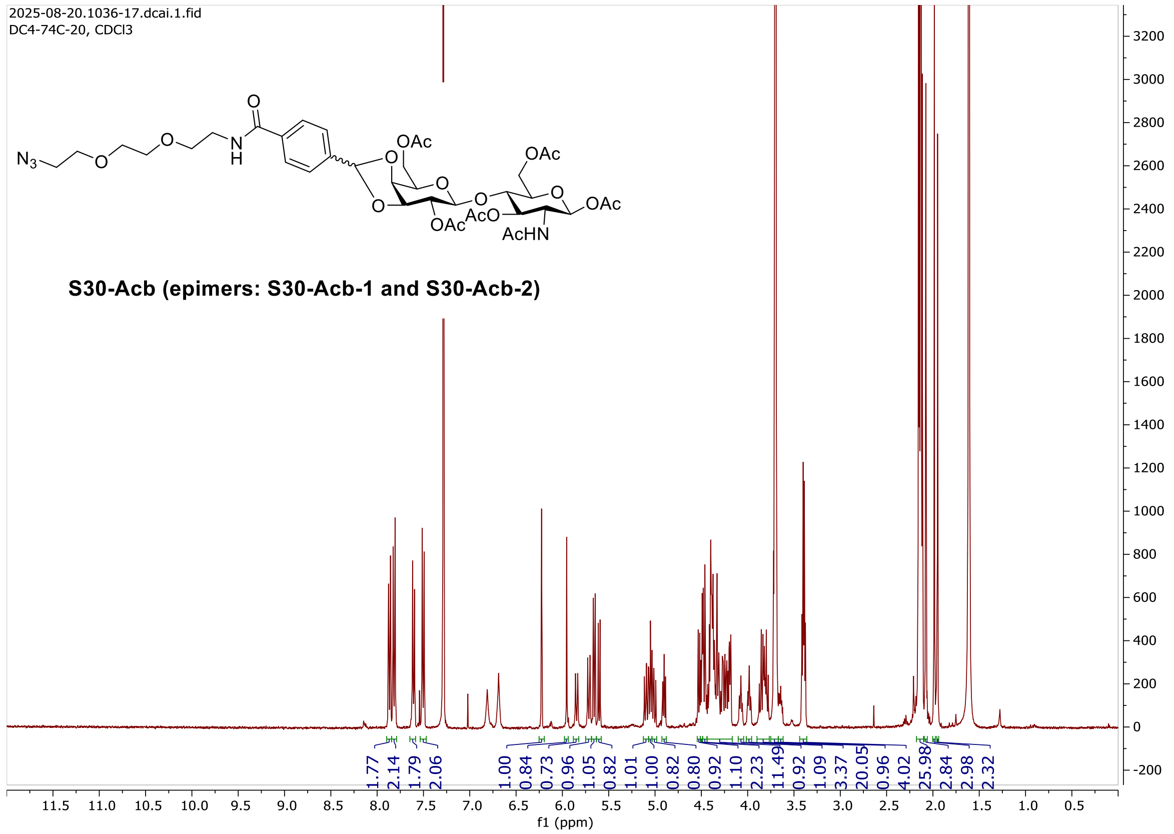
2025-08-20.1036-17.dcai.1.fid
DC4-74C-20, CDCl3
N3
O
O
N
H
O
OAc
O
O
O
OAc
O
AcO
OAc
O
OAc
AcHN
S30-Acb (epimers: S30-Acb-1 and S30-Acb-2)
1.77
2.14
1.79
2.06
1.00
0.84
0.73
0.96
1.05
0.82
1.01
1.00
0.82
0.80
0.92
1.10
2.23
11.49
0.92
1.09
3.37
20.05
0.96
4.02
25.98
2.84
2.98
2.32
f1 (ppm)

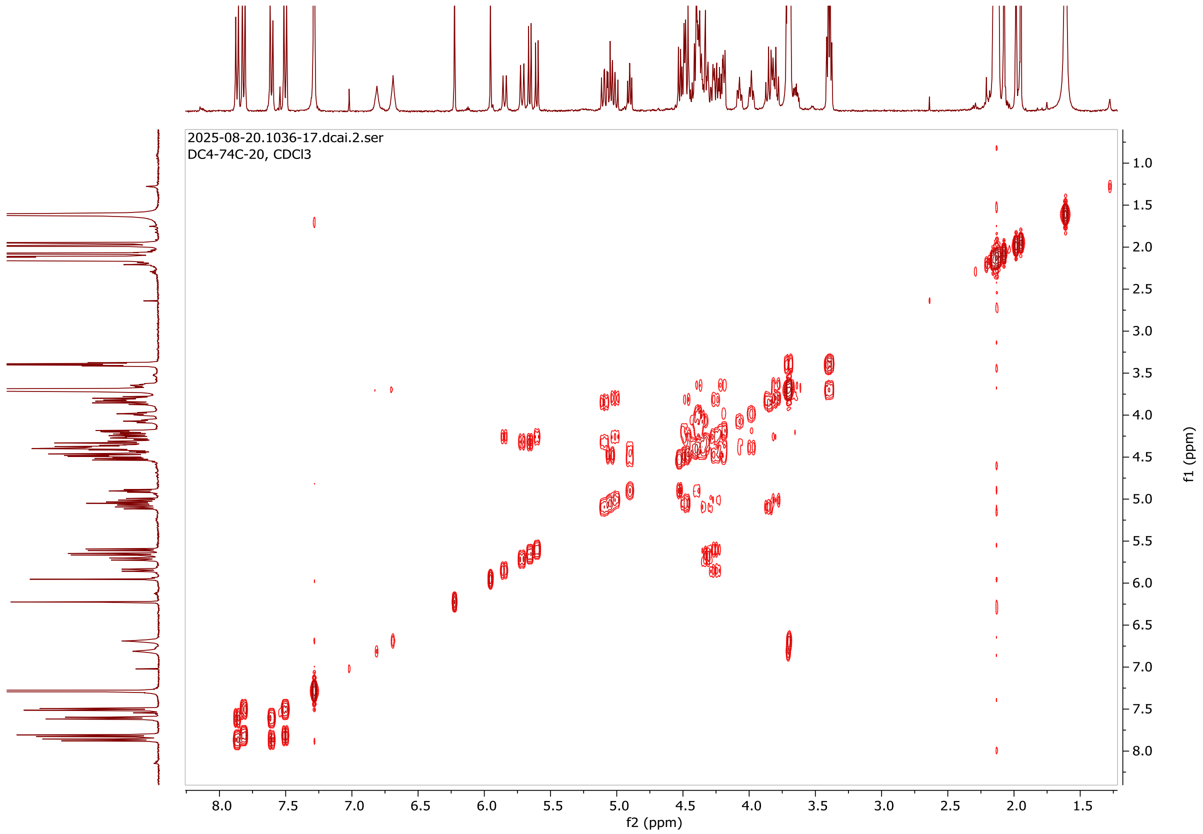
2025-08-20.1036-17.dcai.2.ser
DC4-74C-20, CDCl3
f2 (ppm)
f1 (ppm)

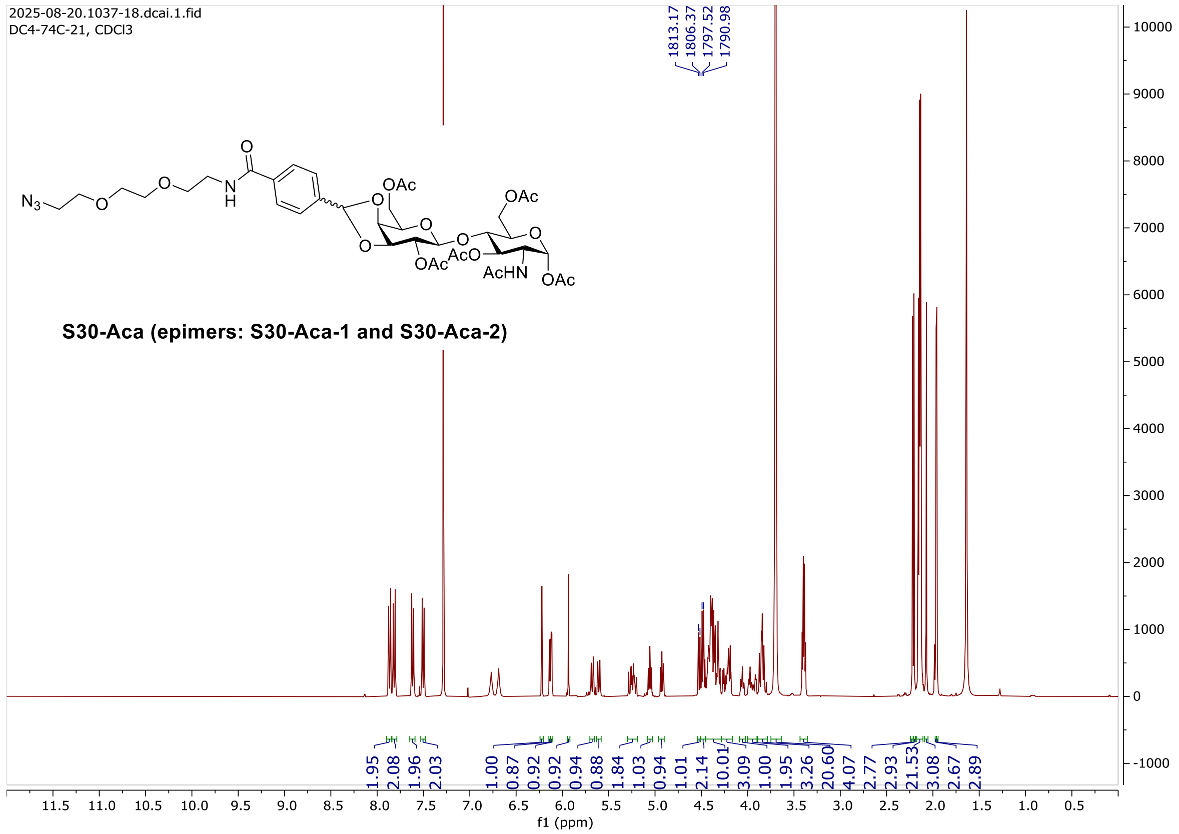
2025-08-20.1037-18.dcai.1.fid
DC4-74C-21, CDCl3
S30-Aca (epimers: S30-Aca-1 and S30-Aca-2)
1813.17
1806.37
1797.52
1790.98
f1 (ppm)

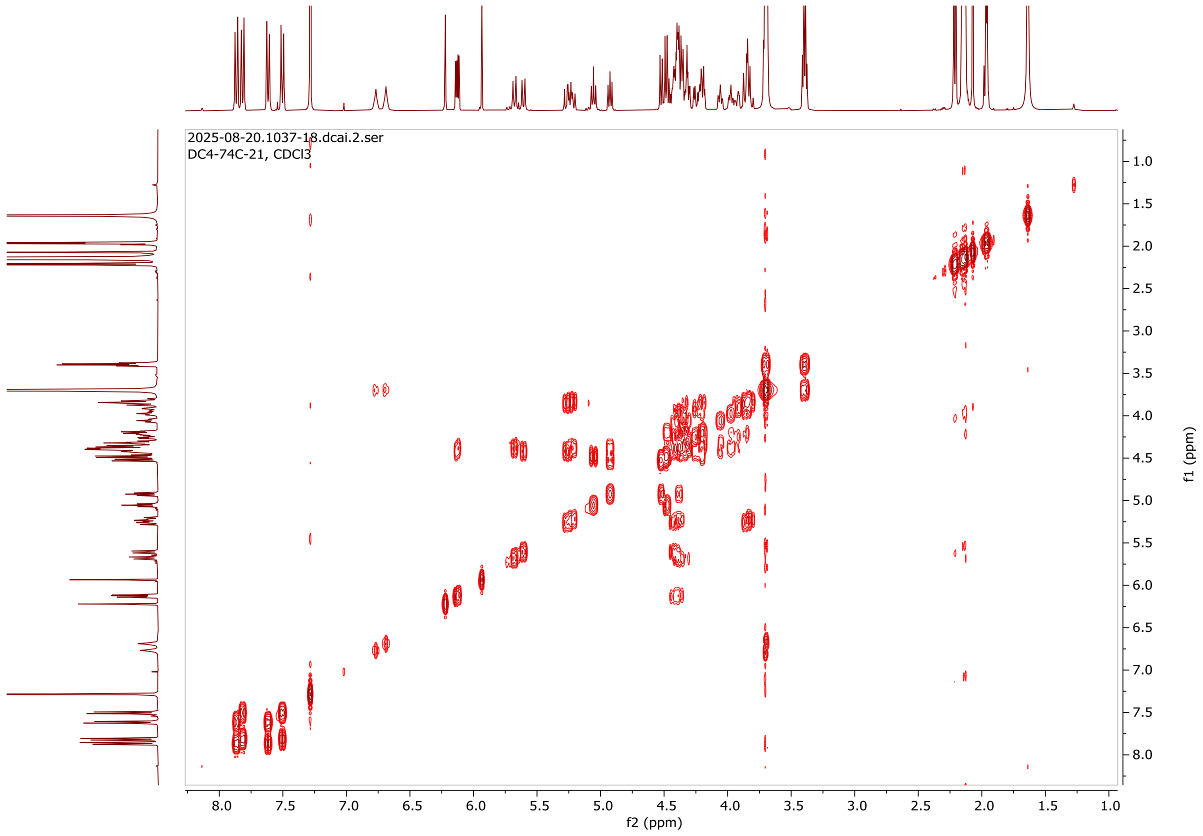
2025-08-20.1037-18.dcai.2.ser
DC4-74C-21, CDCl3
f2 (ppm)
f1 (ppm)

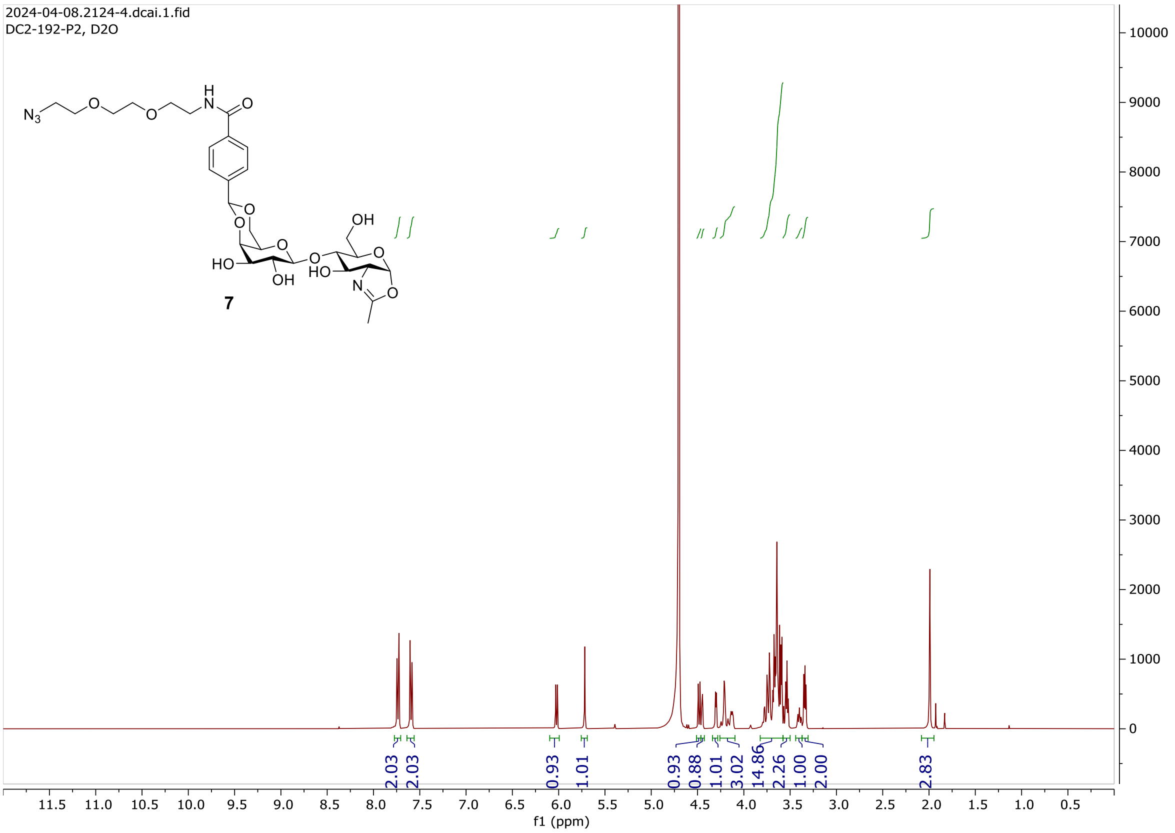
2024-04-08.2124-4.dcai.1.fid
DC2-192-P2, D2O
7
2.03
2.03
0.93
1.01
0.93
0.88
1.01
3.02
14.86
2.26
1.00
2.00
2.83
f1 (ppm)

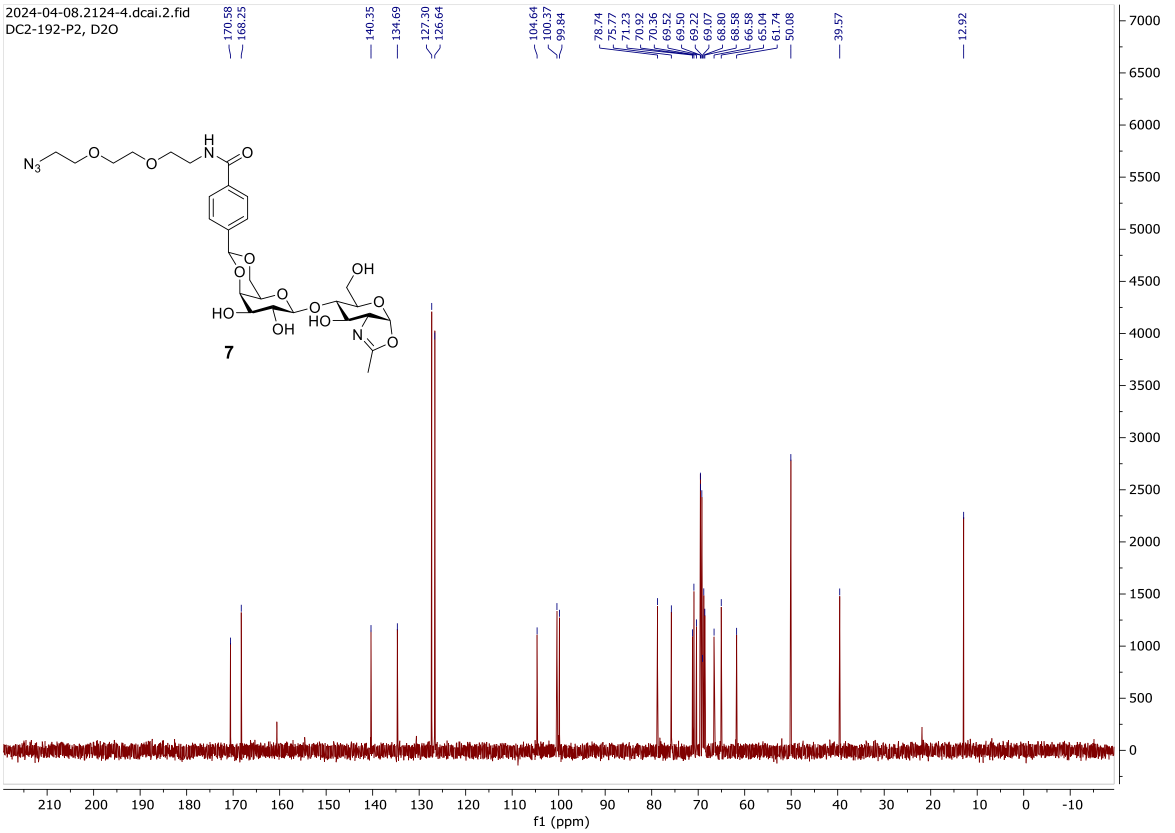
2024-04-08.2124-4.dcai.2.fid
DC2-192-P2, D2O
170.58
168.25
140.35
134.69
127.30
126.64
104.64
100.37
99.84
78.74
75.77
71.23
70.92
70.36
69.52
69.50
69.22
69.07
68.80
68.58
66.58
65.04
61.74
50.08
39.57
12.92
7
f1 (ppm)

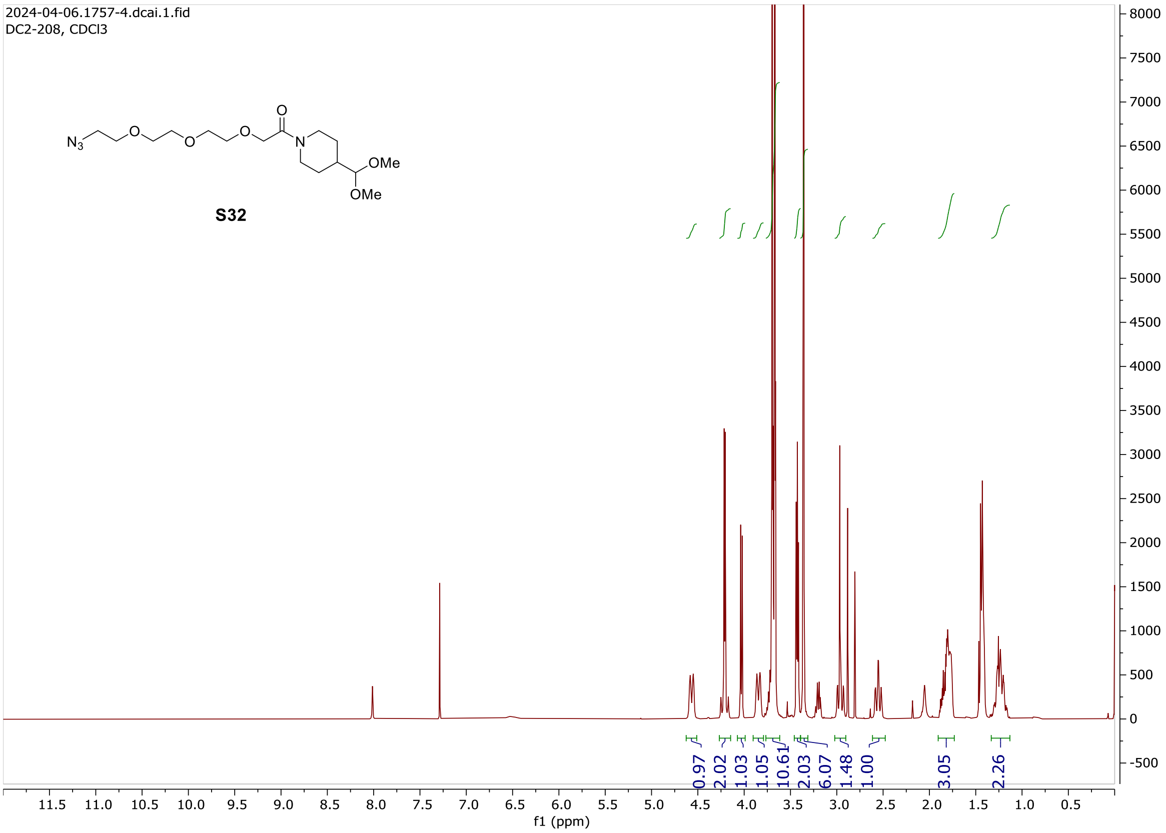
2024-04-06.1757-4.dcai.1.fid
DC2-208, CDCl3
S32
0.97
2.02
1.03
1.05
10.61
2.03
6.07
1.48
1.00
3.05
2.26
f1 (ppm)

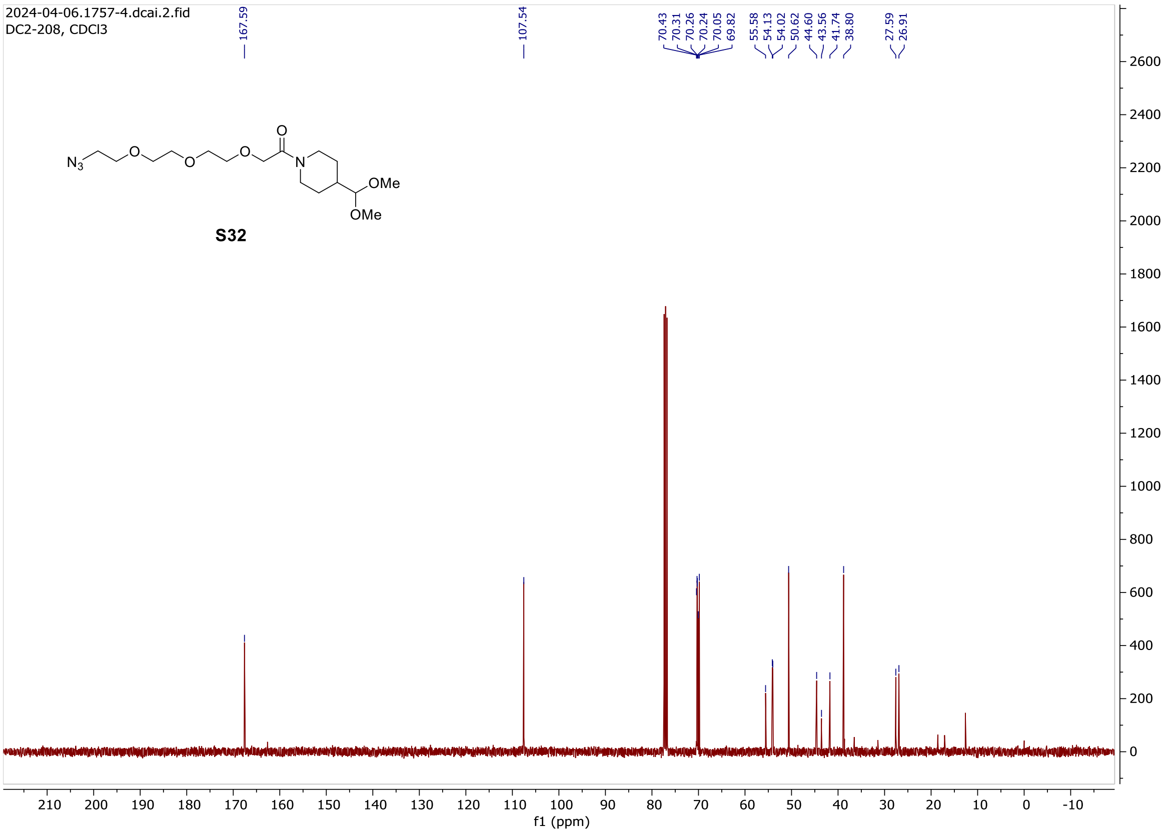
2024-04-06.1757-4.dcai.2.fid
DC2-208, CDCl3
167.59
107.54
70.43
70.31
70.26
70.24
70.05
69.82
55.58
54.13
54.02
50.62
44.60
43.56
41.74
38.80
27.59
26.91
S32
f1 (ppm)

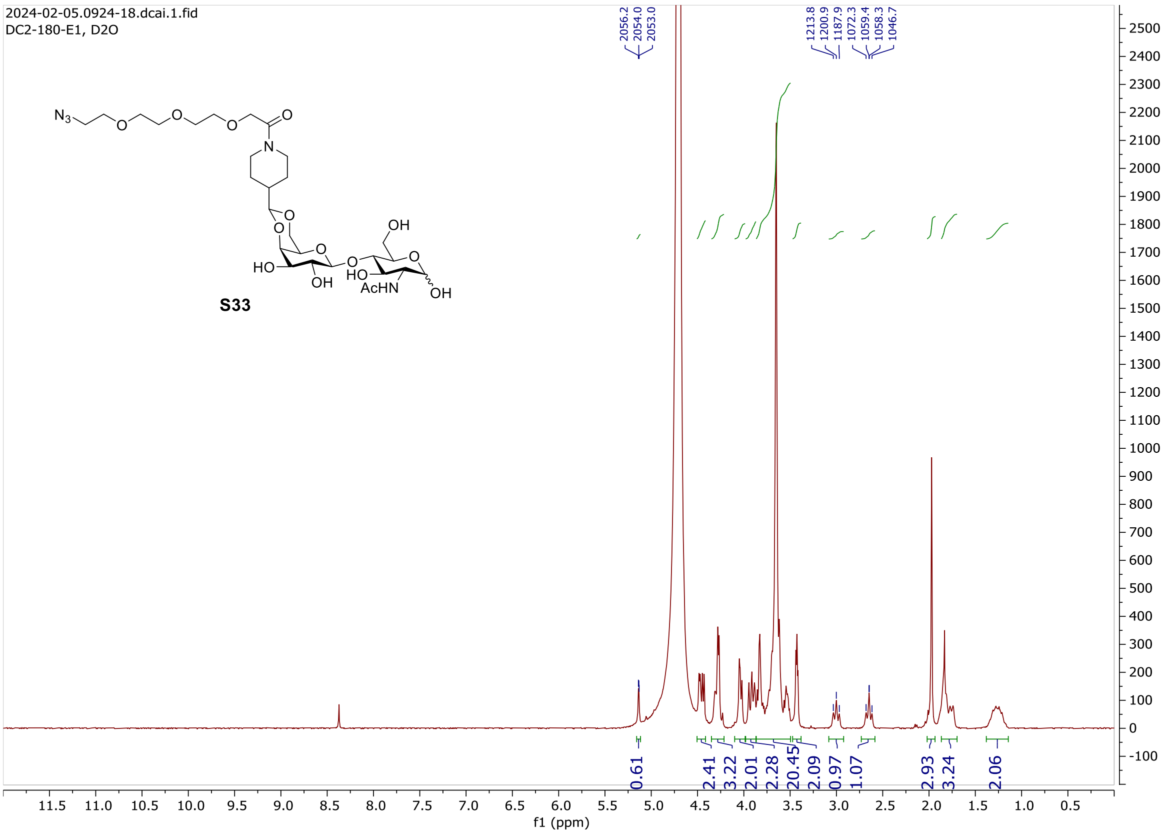
2024-02-05.0924-18.dcai.1.fid
DC2-180-E1, D2O
2056.2
2054.0
2053.0
1213.8
1200.9
1187.9
1072.3
1059.4
1058.3
1046.7
S33
0.61
2.41
3.22
2.01
2.28
20.45
2.09
0.97
1.07
2.93
3.24
2.06
f1 (ppm)

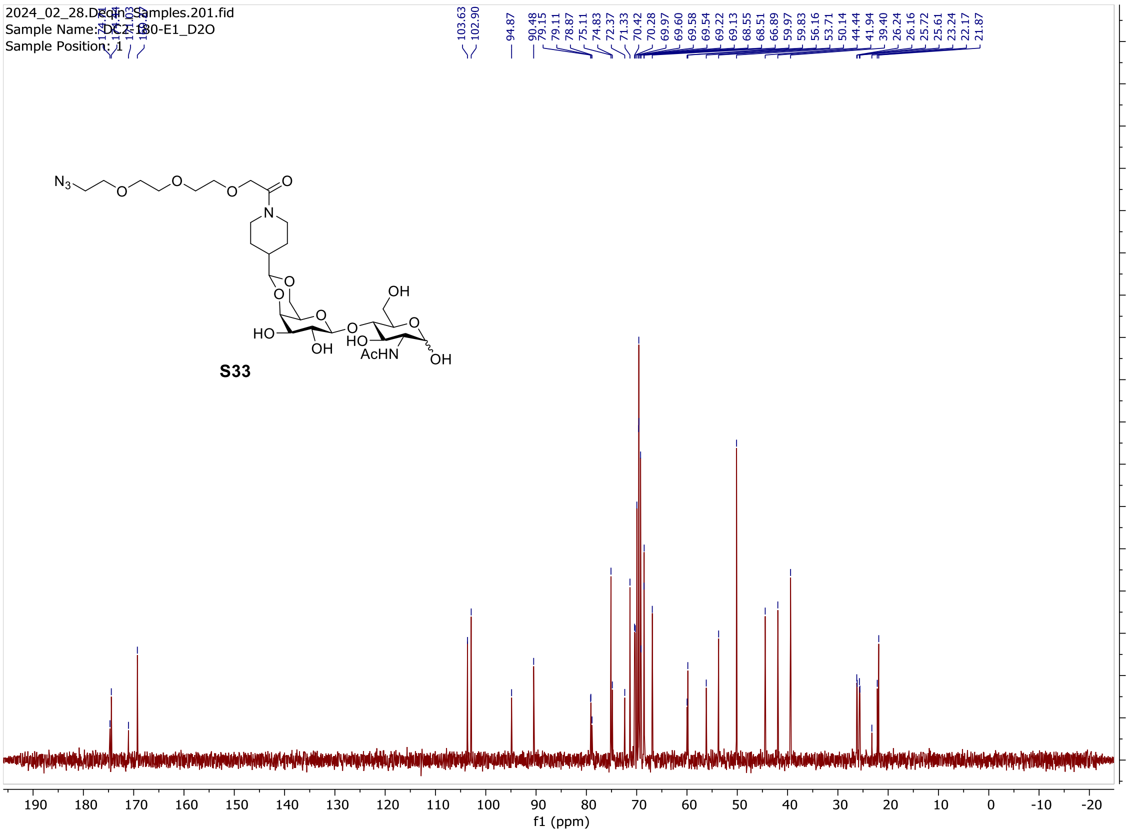
Sample Position: 1
103.63
102.90
94.87
90.48
79.15
79.11
78.87
75.11
74.83
72.37
71.33
70.42
70.28
69.97
69.60
69.58
69.54
69.22
69.13
68.55
68.51
66.89
59.97
59.83
56.16
53.71
50.14
44.44
41.94
39.40
26.24
26.16
25.72
25.61
23.24
22.17
21.87
S33
f1 (ppm)

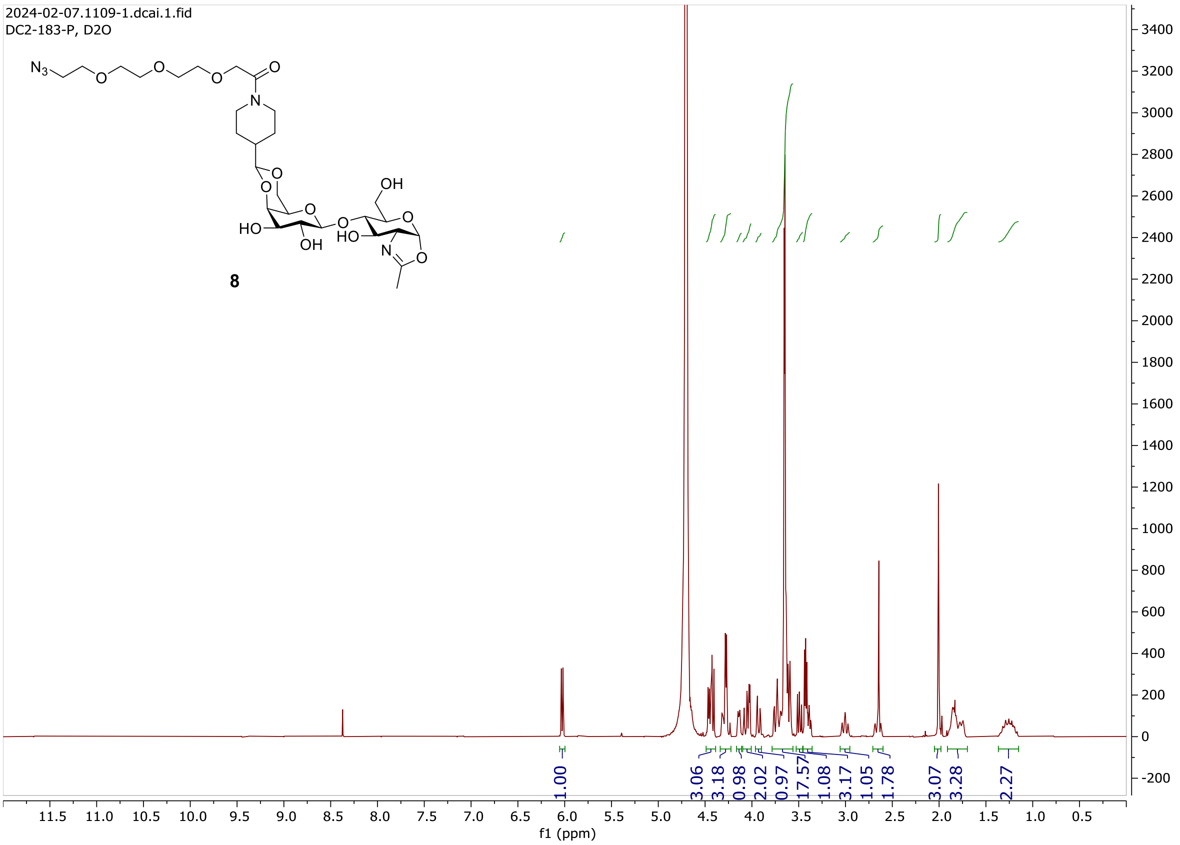
2024-02-07.1109-1.dcai.1.fid
DC2-183-P, D2O
8
1.00
3.06
3.18
0.98
2.02
0.97
17.57
1.08
3.17
1.05
1.78
3.07
3.28
2.27
f1 (ppm)

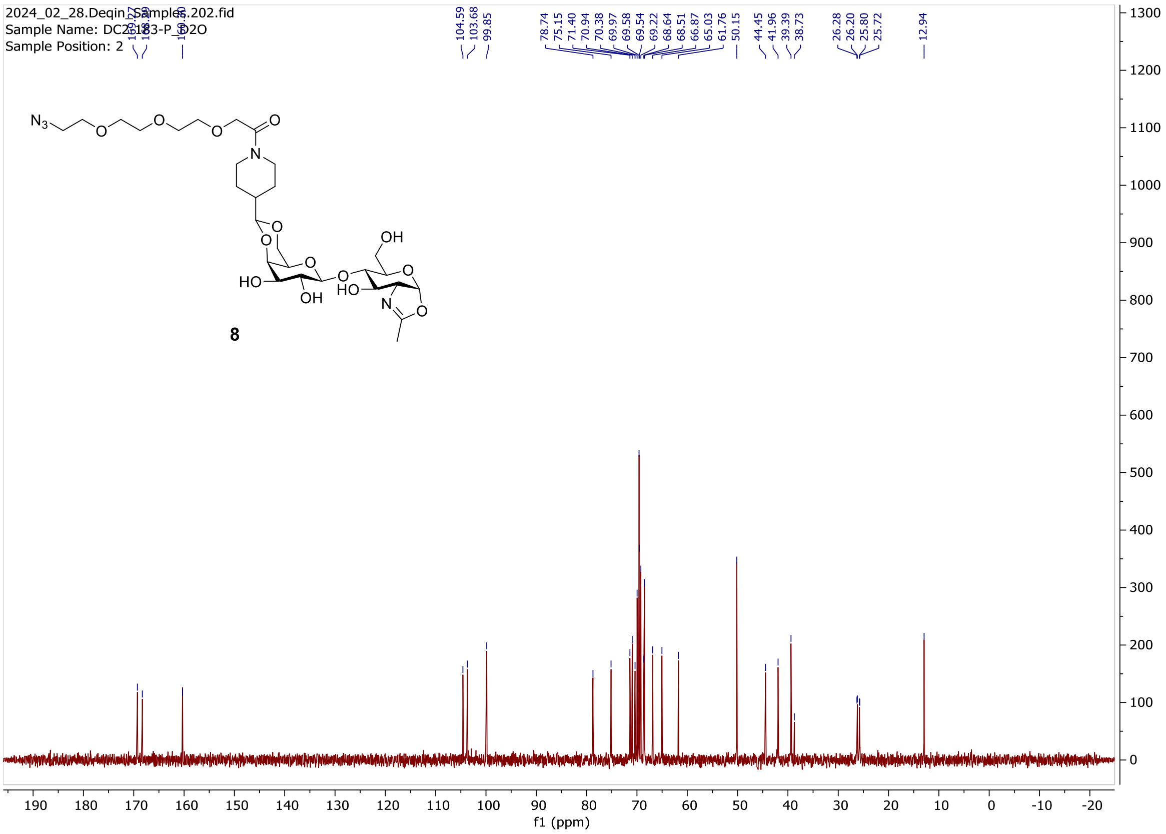
2024_02_28.Deqin_Samples.202.fid
Sample Name: DC2-183-P_D2O
Sample Position: 2
104.59
103.68
99.85
78.74
75.15
71.40
70.94
70.38
69.97
69.58
69.54
69.22
68.64
68.51
66.87
65.03
61.76
50.15
44.45
41.96
39.39
38.73
26.28
26.20
25.80
25.72
12.94
8
f1 (ppm)

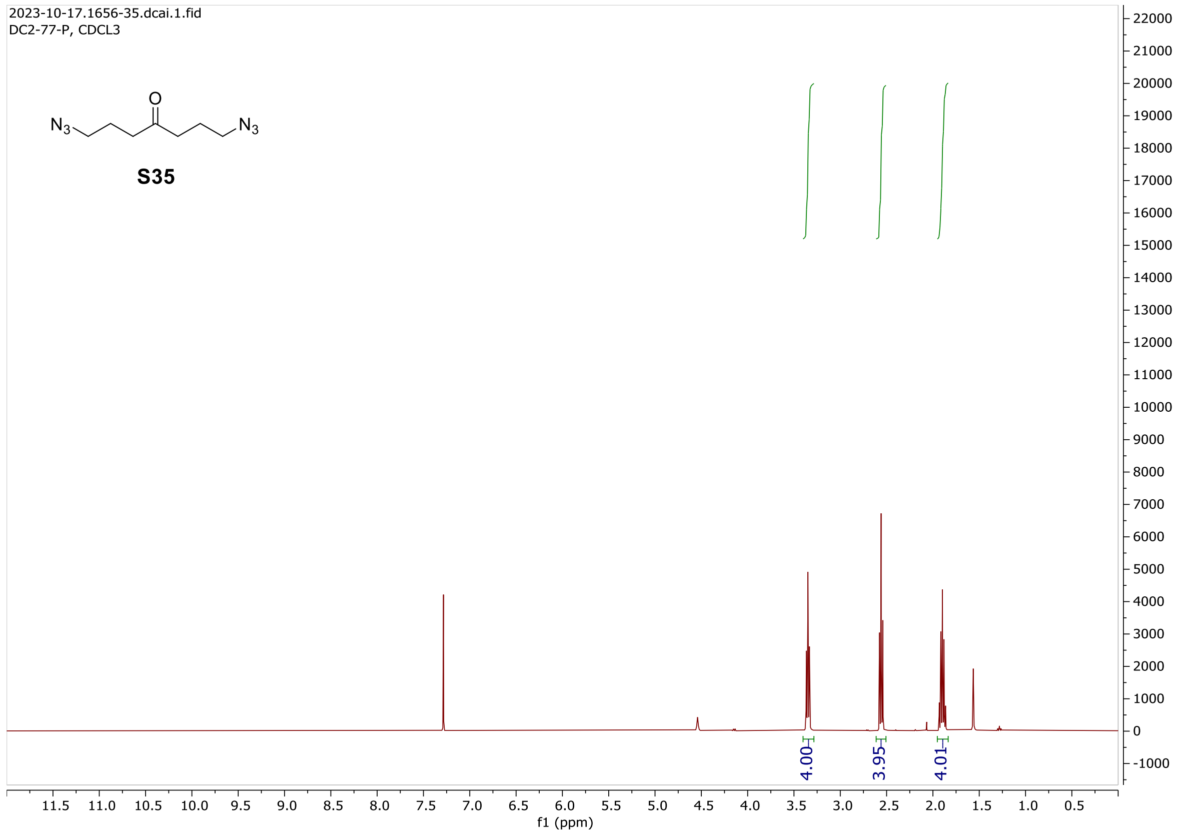
2023-10-17.1656-35.dcai.1.fid
DC2-77-P, CDCL3
S35
4.00
3.95
4.01
f1 (ppm)

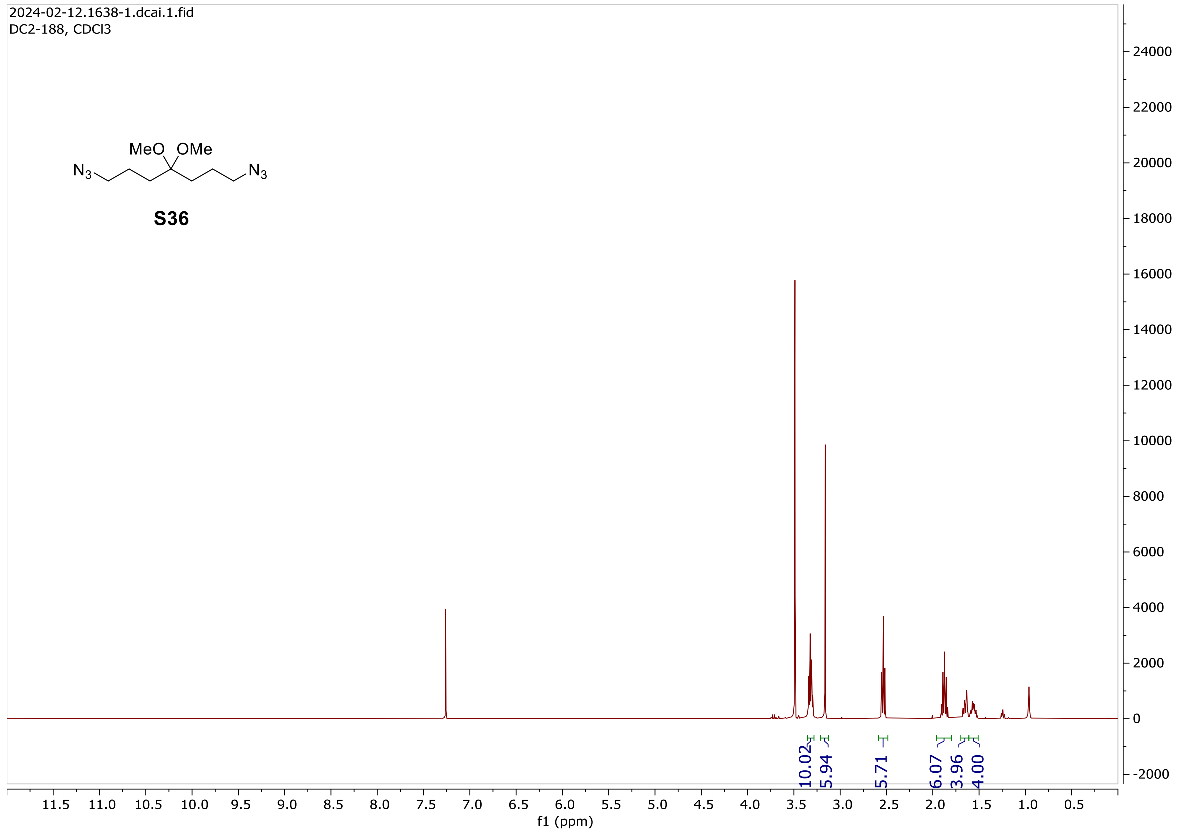
2024-02-12.1638-1.dcai.1.fid
DC2-188, CDCl3
MeO OMe
S36
10.02
5.94
5.71
6.07
3.96
4.00
f1 (ppm)

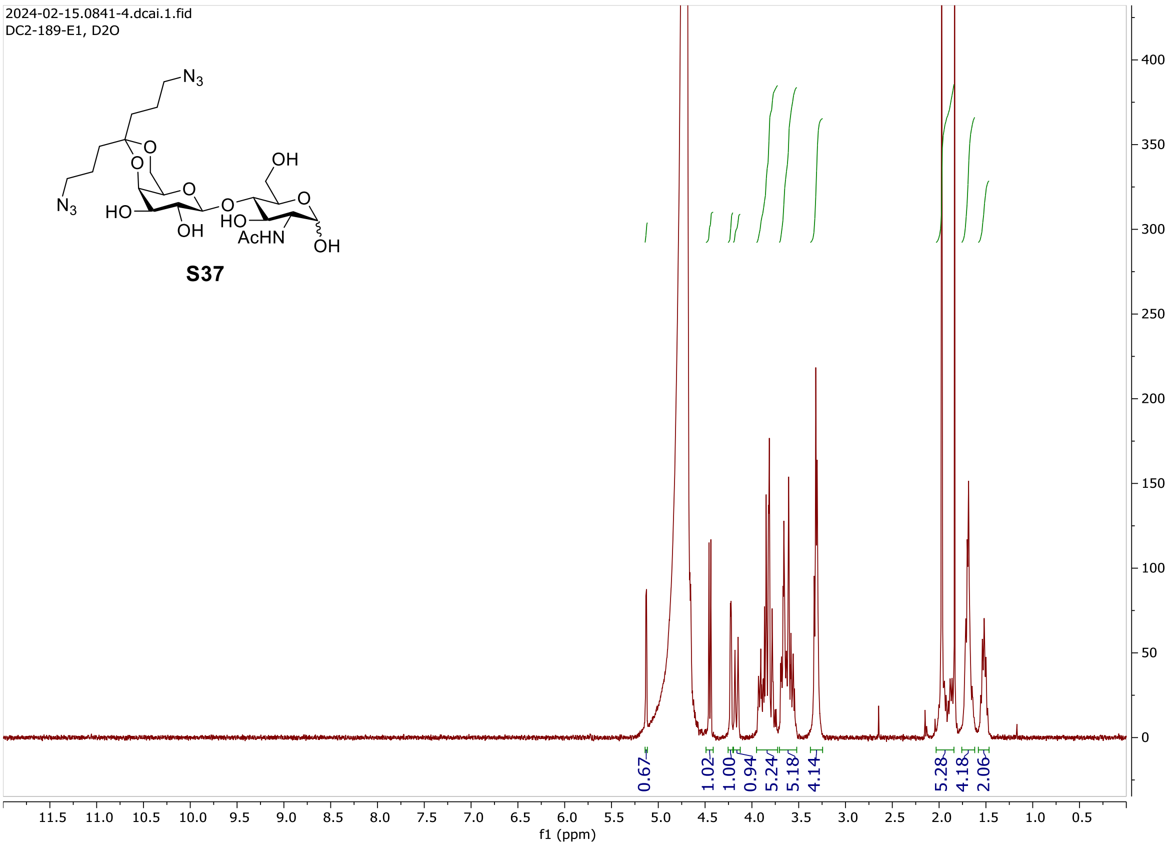
2024-02-15.0841-4.dcai.1.fid
DC2-189-E1, D2O
S37
0.67
1.02
1.00
0.94
5.24
5.18
4.14
5.28
4.18
2.06
f1 (ppm)

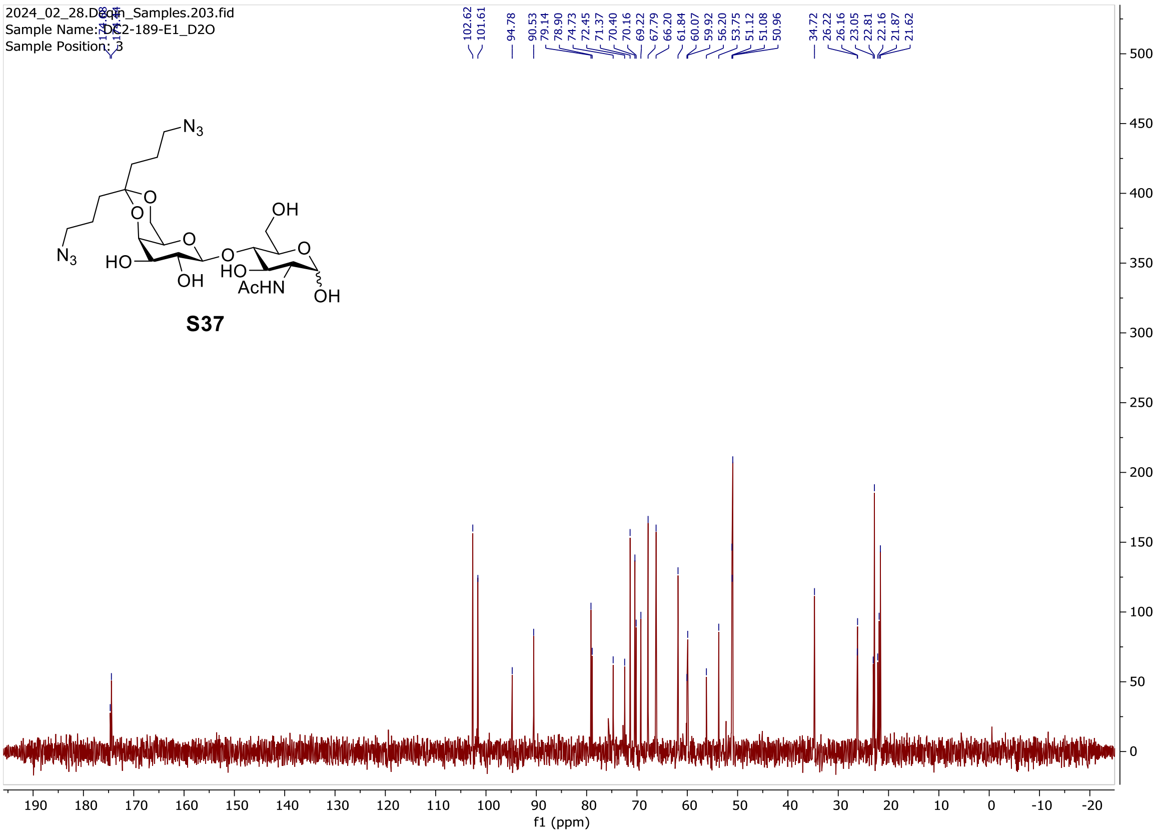
2024_02_28.Dean_Samples.203.fid
Sample Name: DC2-189-E1_D2O
Sample Position: 3
102.62
101.61
94.78
90.53
79.14
78.90
74.73
72.45
71.37
70.40
70.16
69.22
67.79
66.20
61.84
60.07
59.92
56.20
53.75
51.12
51.08
50.96
34.72
26.22
26.16
23.05
22.81
22.16
21.87
21.62
S37
f1 (ppm)

2024-02-19.1234-8.dcai.1.fid
DC2-189-E1-Ac-E5, DMSO-D6

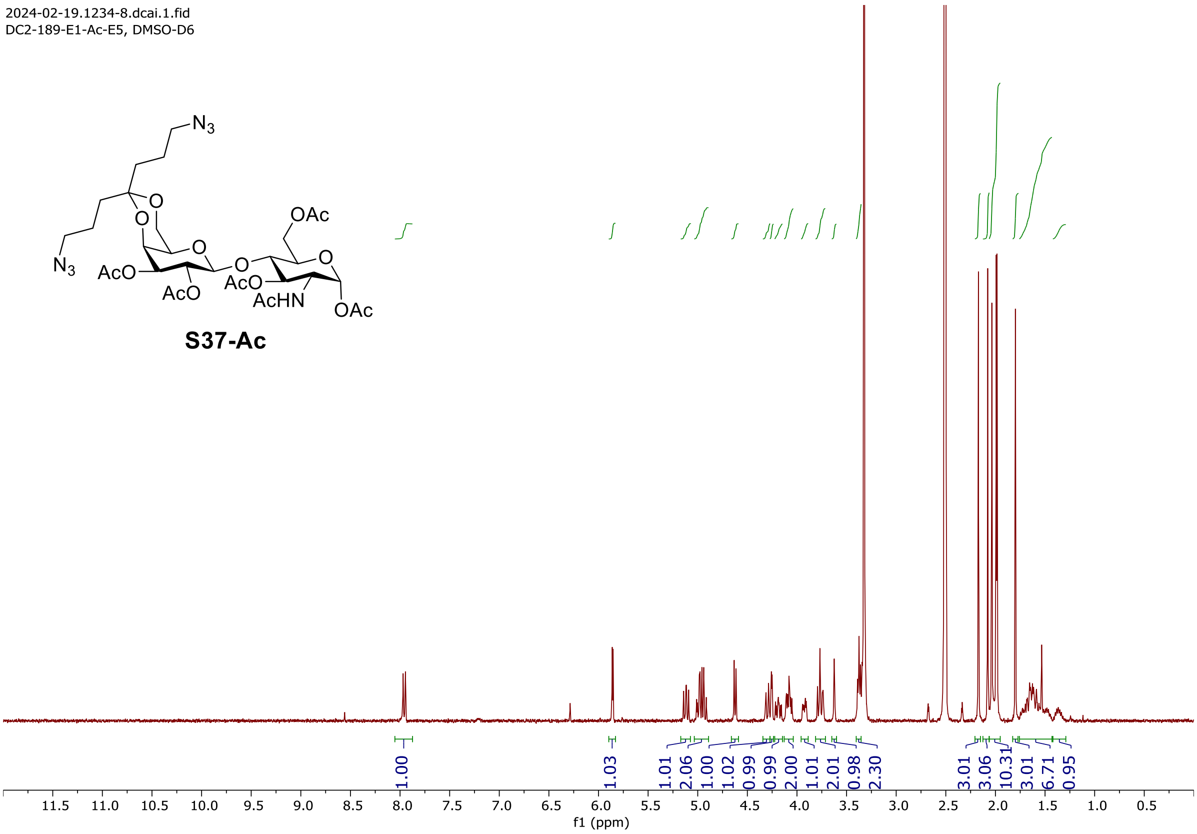

S37-Ac

2024_02_28.Deqin_Samples.208.fid
Sample Name: DC2-189-E1-Ac-E5_DMSO
Sample Position: 8

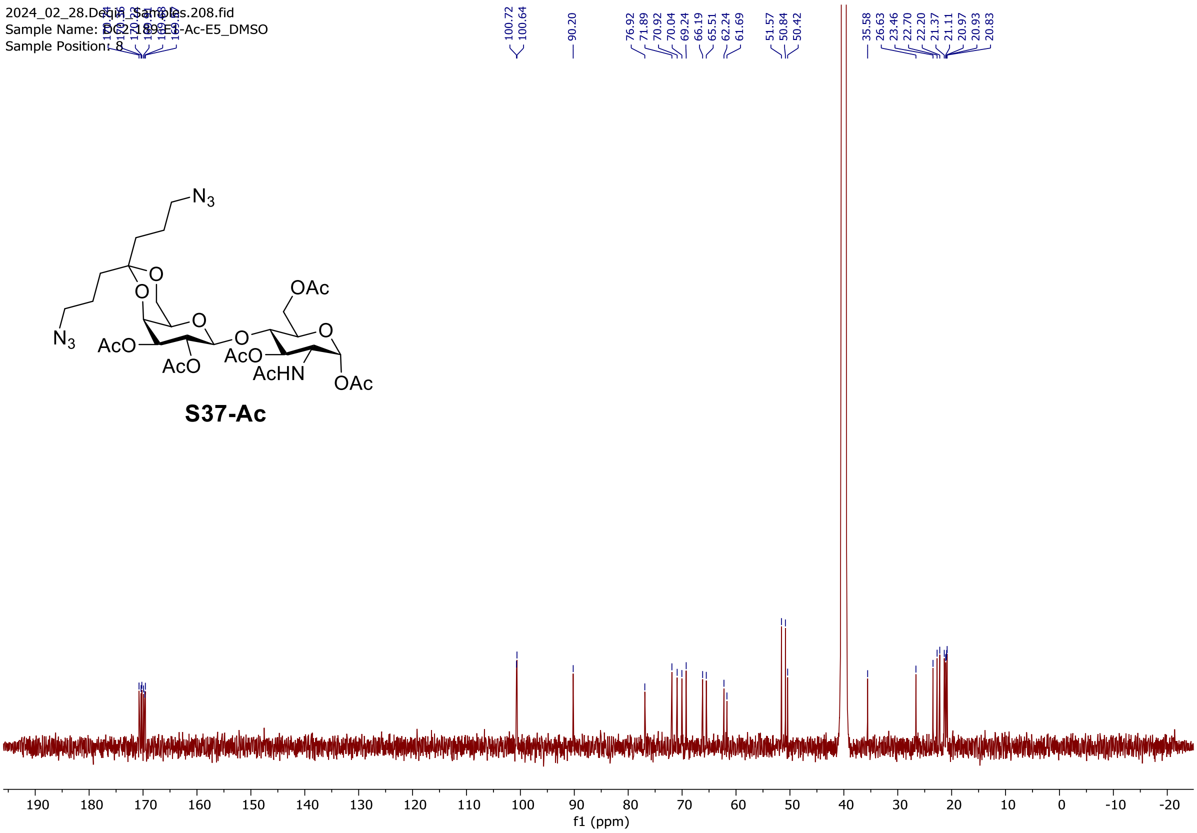

S37-Ac

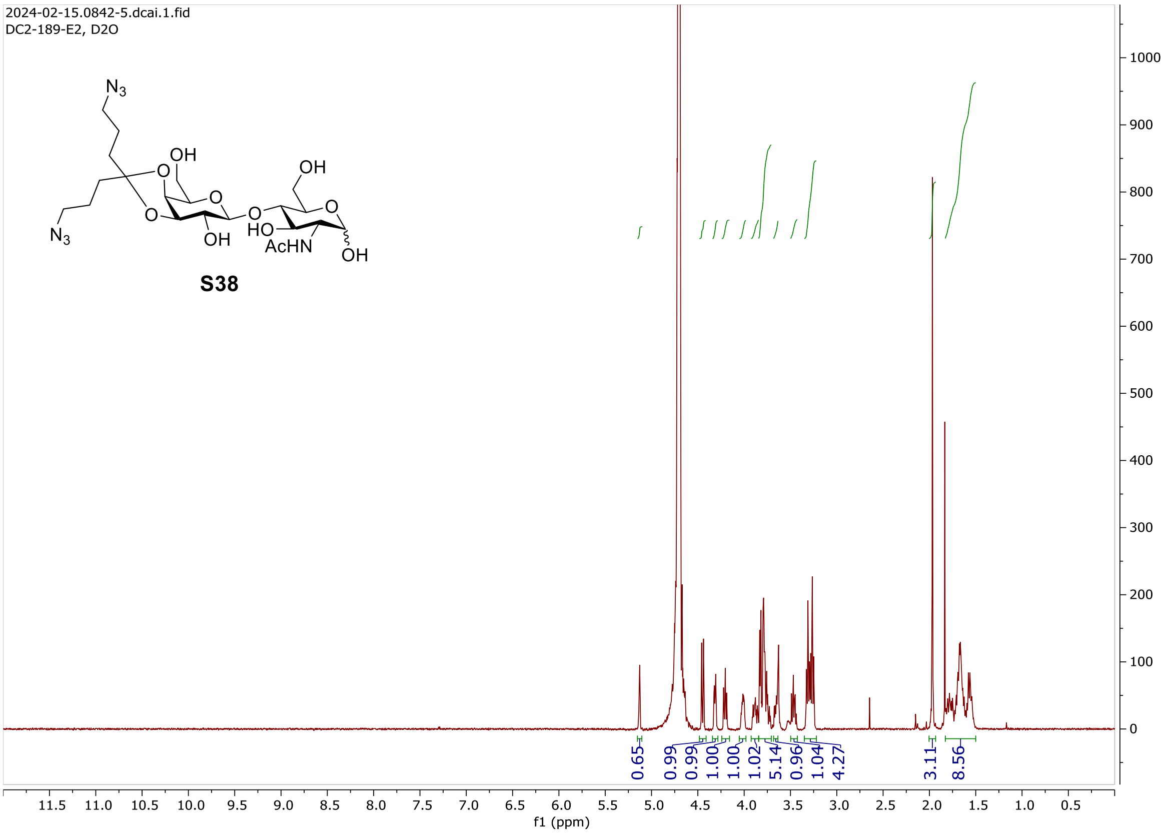
2024-02-15.0842-5.dcai.1.fid
DC2-189-E2, D2O
N3
OH
O
OH
O
O
O
N3
OH
HO
AcHN
OH
S38
0.65
0.99
0.99
1.00
1.00
1.02
5.14
0.96
1.04
4.27
3.11
8.56
f1 (ppm)

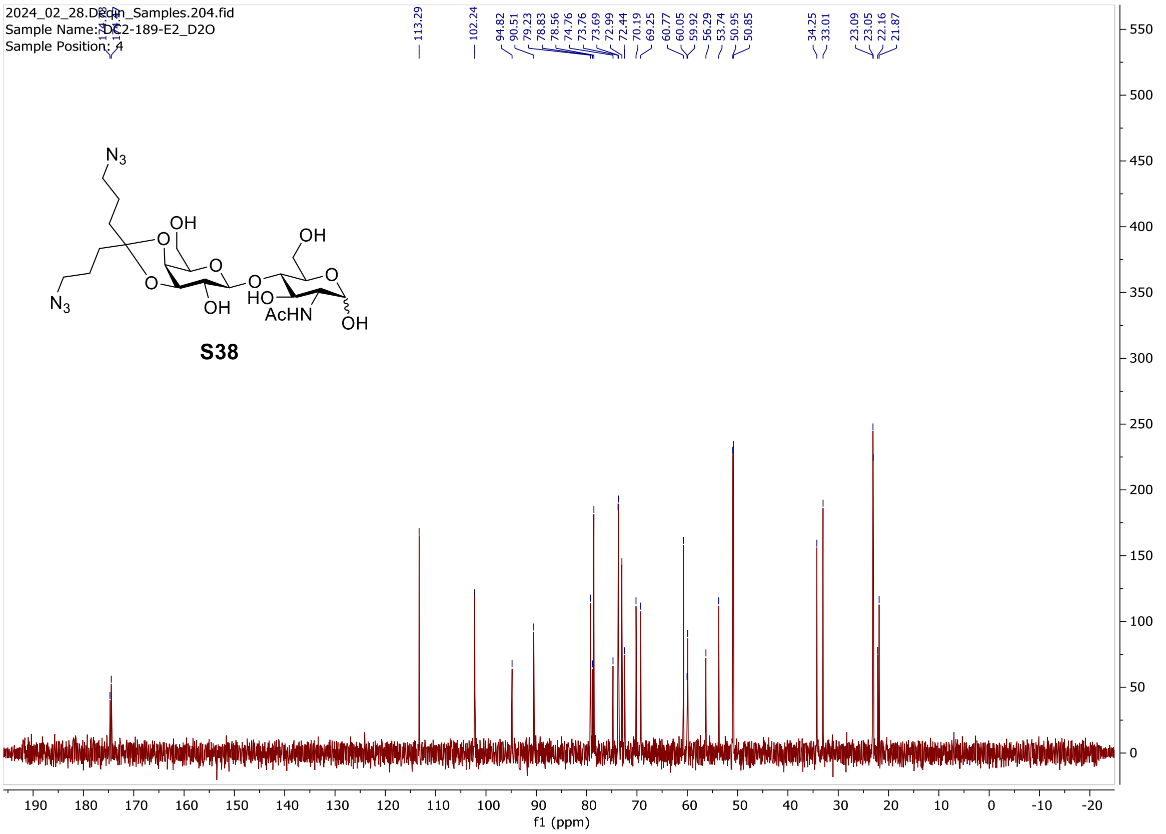
2024_02_28.D[illegible]_Samples.204.fid
Sample Name: DC2-189-E2_D2O
Sample Position: 4
113.29
102.24
94.82
90.51
79.23
78.83
78.56
74.76
73.76
73.69
72.99
72.44
70.19
69.25
60.77
60.05
59.92
56.29
53.74
50.95
50.85
34.25
33.01
23.09
23.05
22.16
21.87
S38
f1 (ppm)

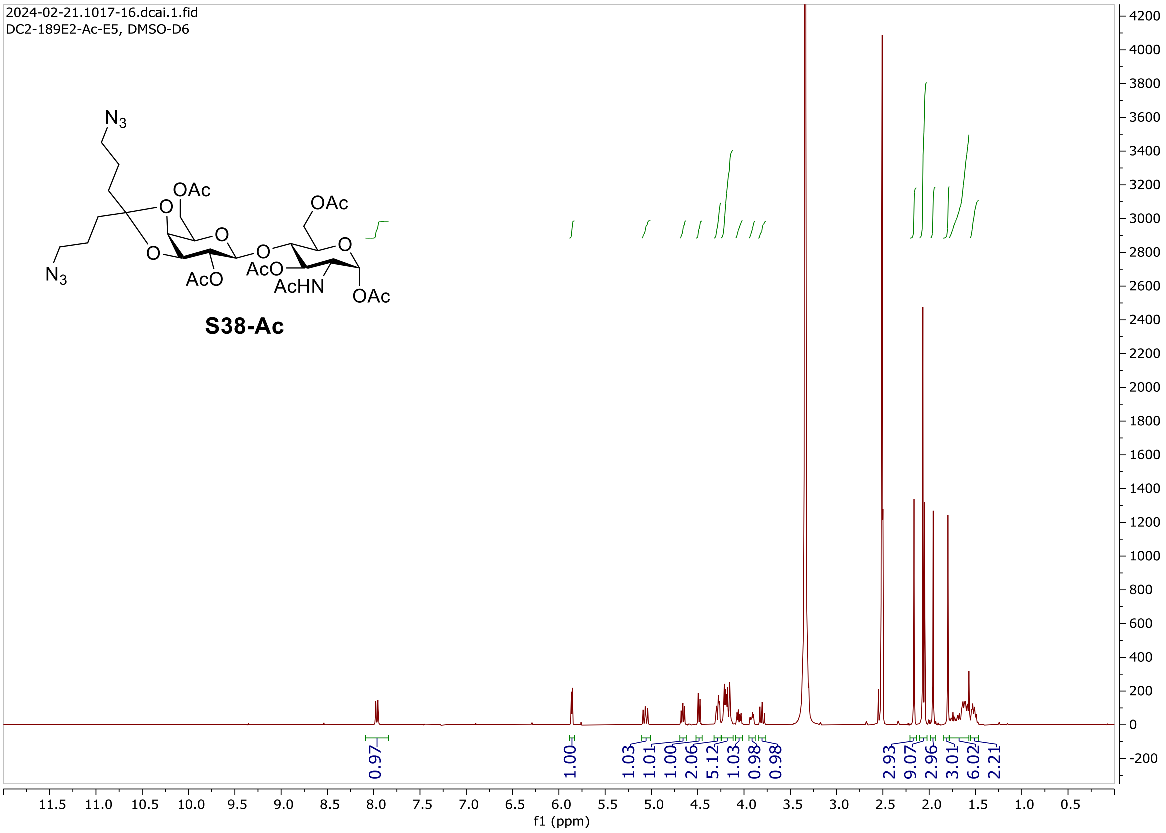
2024-02-21.1017-16.dcai.1.fid
DC2-189E2-Ac-E5, DMSO-D6
S38-Ac
0.97
1.00
1.03
1.01
1.00
2.06
5.12
1.03
0.98
0.98
2.93
9.07
2.96
3.01
6.02
2.21
f1 (ppm)

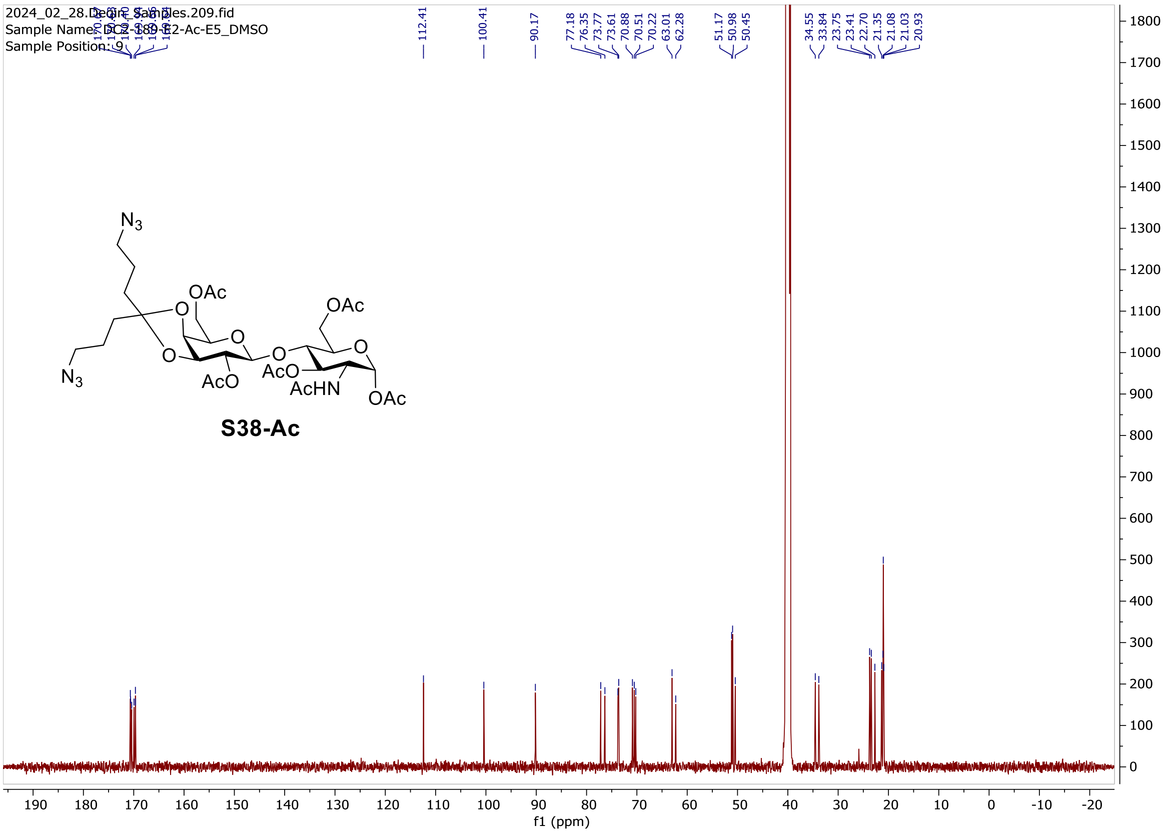
2024_02_28.Degre_Samples.209.fid
Sample Name: DC2-189-E2-Ac-E5_DMSO
Sample Position: 9
112.41
100.41
90.17
77.18
76.35
73.77
73.61
70.88
70.51
70.22
63.01
62.28
51.17
50.98
50.45
34.55
33.84
23.75
23.41
22.70
21.35
21.08
21.03
20.93
S38-Ac
f1 (ppm)

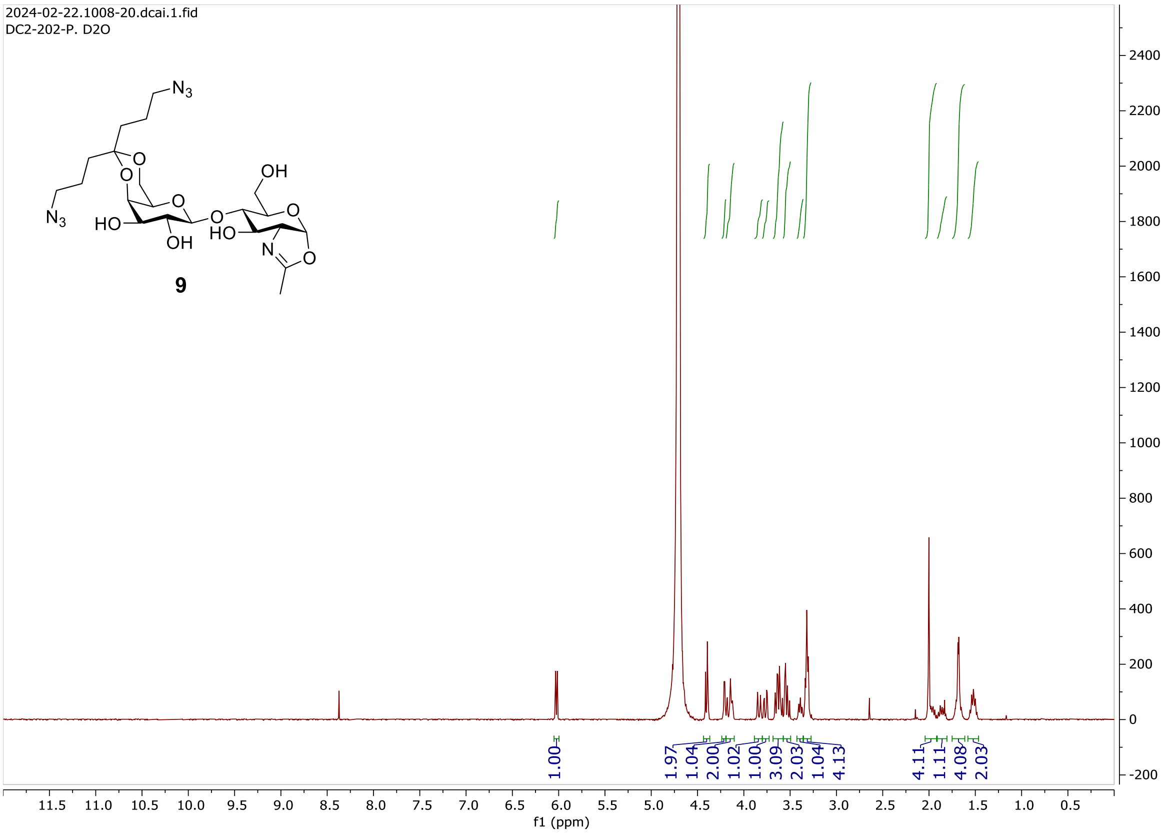
2024-02-22.1008-20.dcai.1.fid
DC2-202-P. D2O
9
f1 (ppm)

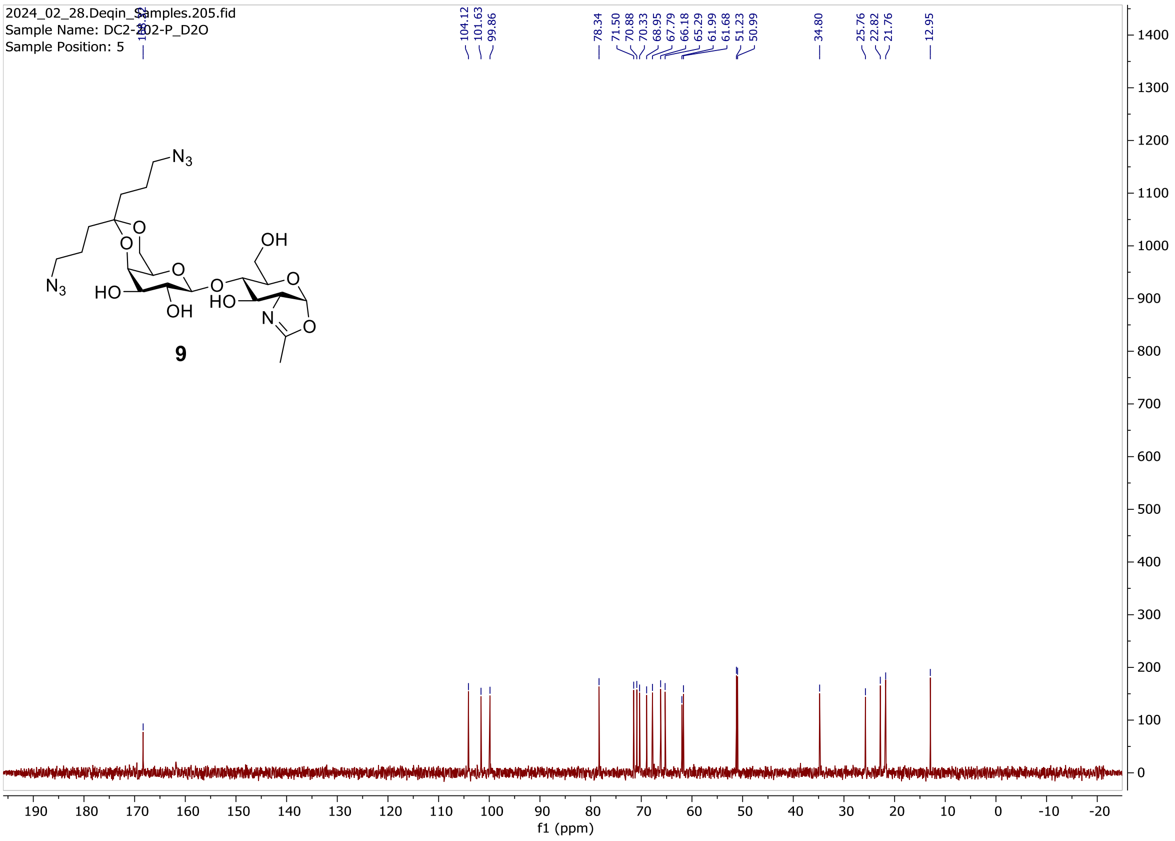
2024_02_28.Deqin_Samples.205.fid
Sample Name: DC2-202-P_D2O
Sample Position: 5
104.12
101.63
99.86
78.34
71.50
70.88
70.33
68.95
67.79
66.18
65.29
61.99
61.68
51.23
50.99
34.80
25.76
22.82
21.76
12.95
9
f1 (ppm)

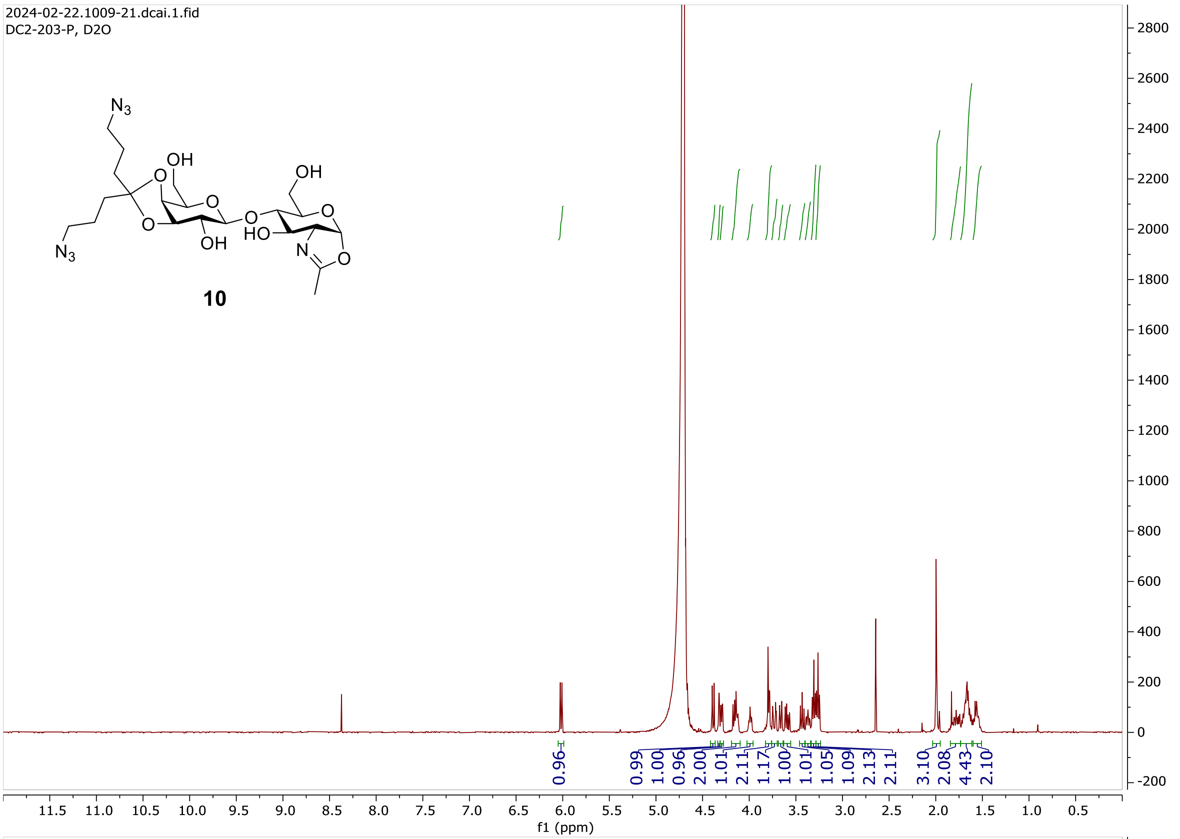
2024-02-22.1009-21.dcai.1.fid
DC2-203-P, D2O
N3
OH
O
O
OH
O
OH
HO
N
O
N3
10
0.96
0.99
1.00
0.96
2.00
1.01
2.11
1.17
1.00
1.01
1.05
1.09
2.13
2.11
3.10
2.08
4.43
2.10
f1 (ppm)

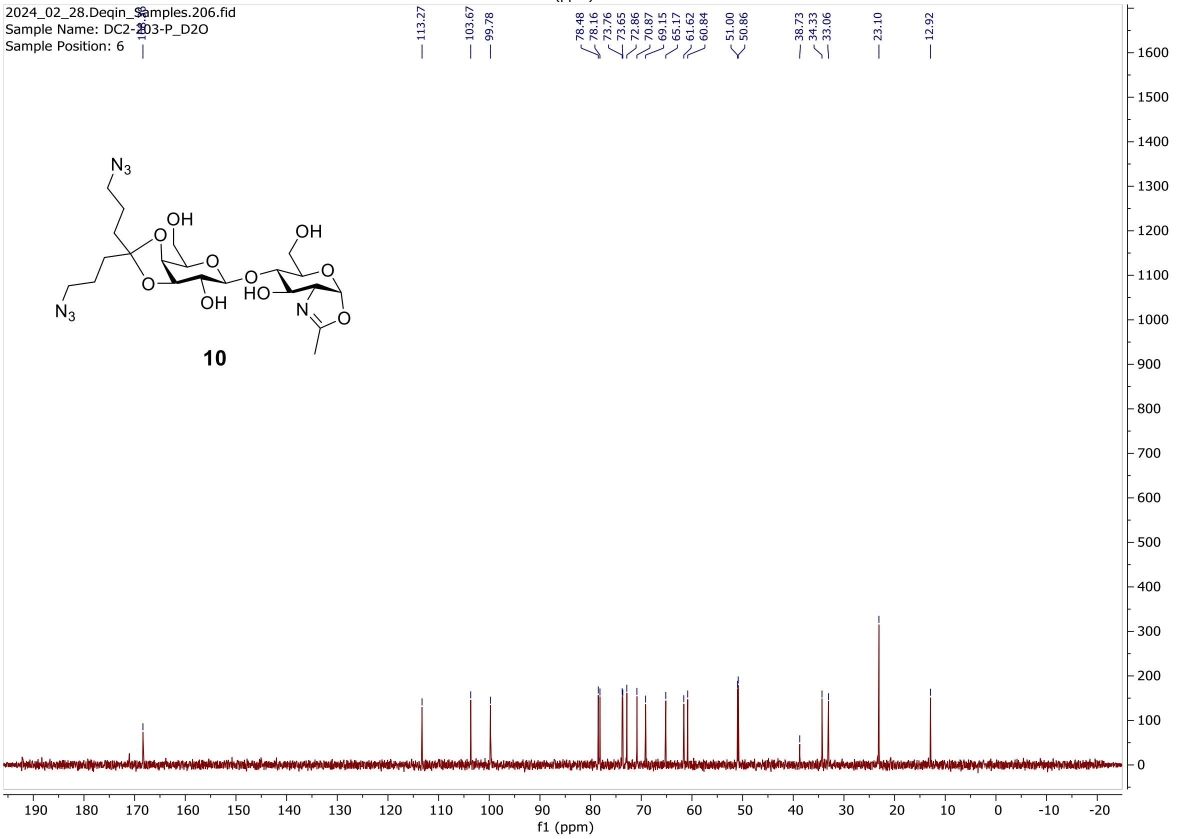
2024_02_28.Deqin_Samples.206.fid
Sample Name: DC2-203-P_D2O
Sample Position: 6
113.27
103.67
99.78
78.48
78.16
73.76
73.65
72.86
70.87
69.15
65.17
61.62
60.84
51.00
50.86
38.73
34.33
33.06
23.10
12.92
N3
OH
O
O
OH
O
OH
HO
N
O
N3
10
f1 (ppm)

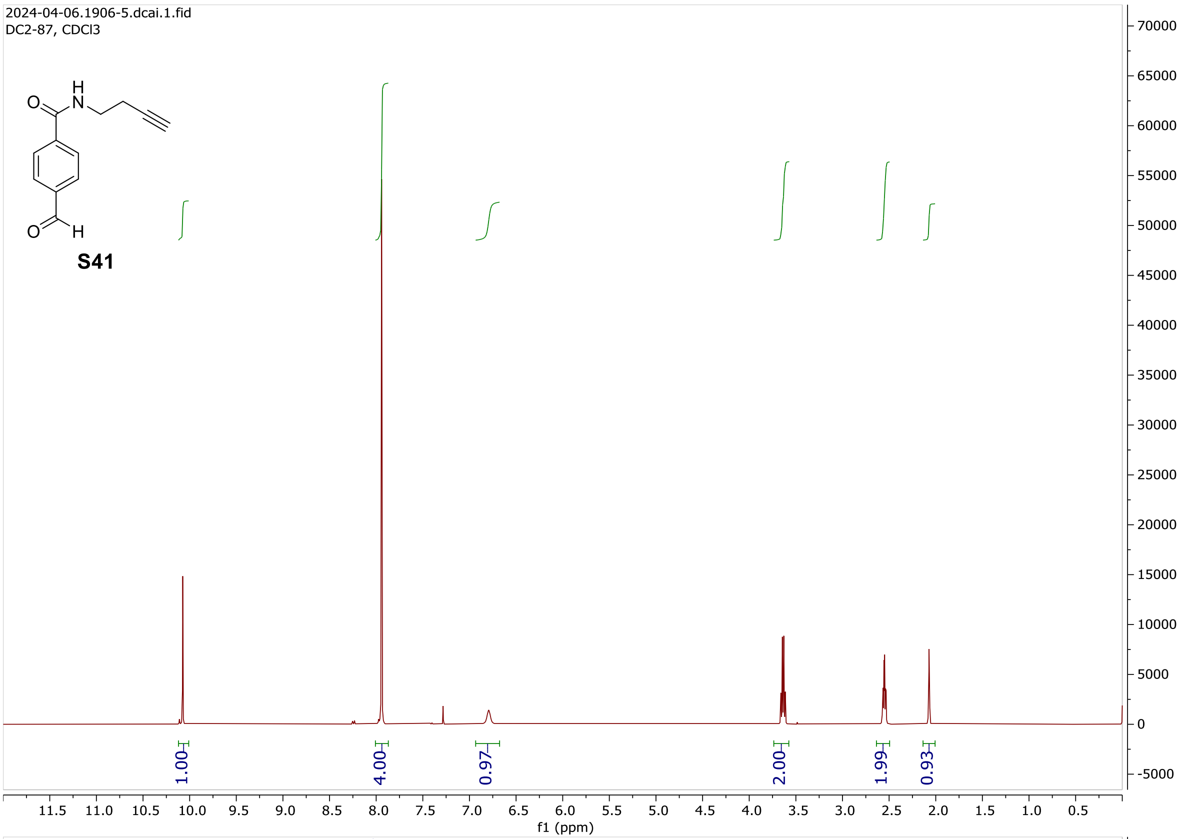
2024-04-06.1906-5.dcai.1.fid
DC2-87, CDCl3
S41
1.00
4.00
0.97
2.00
1.99
0.93
f1 (ppm)

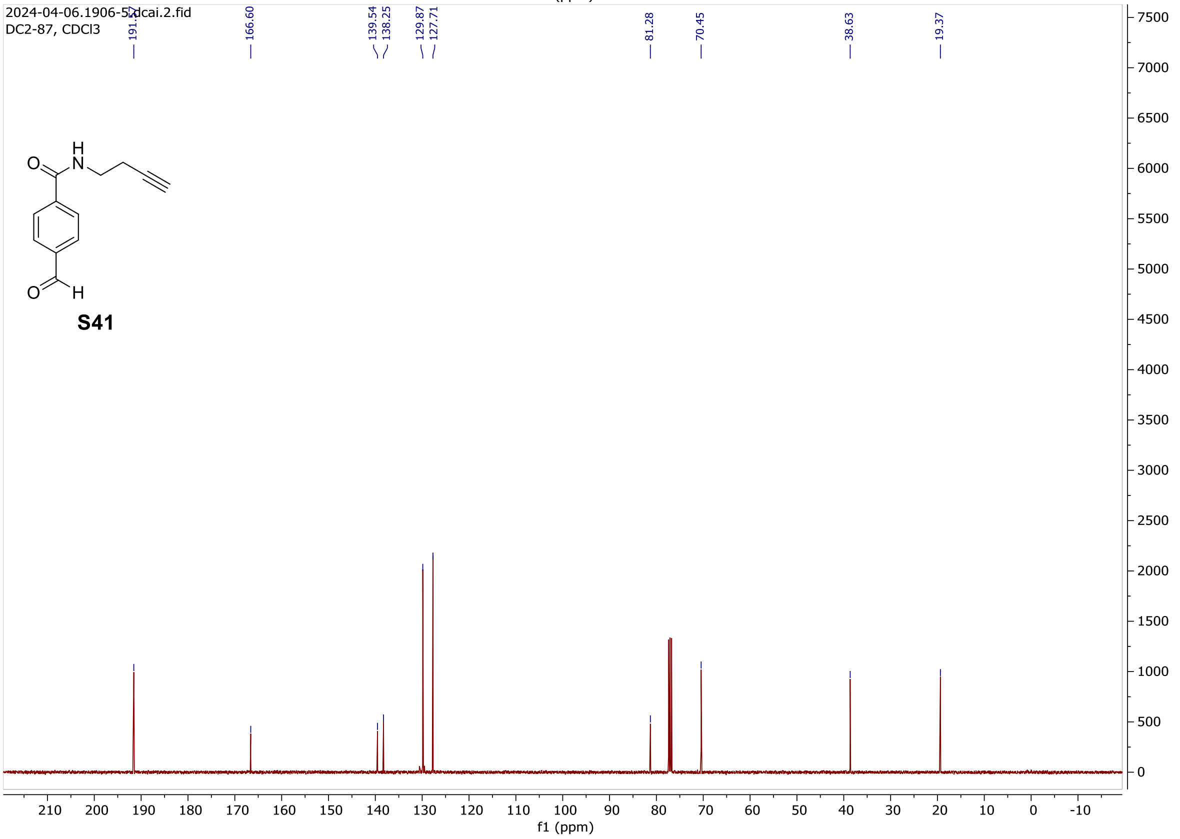
DC2-87, CDCl3
166.60
139.54
138.25
129.87
127.71
81.28
70.45
38.63
19.37
S41
f1 (ppm)

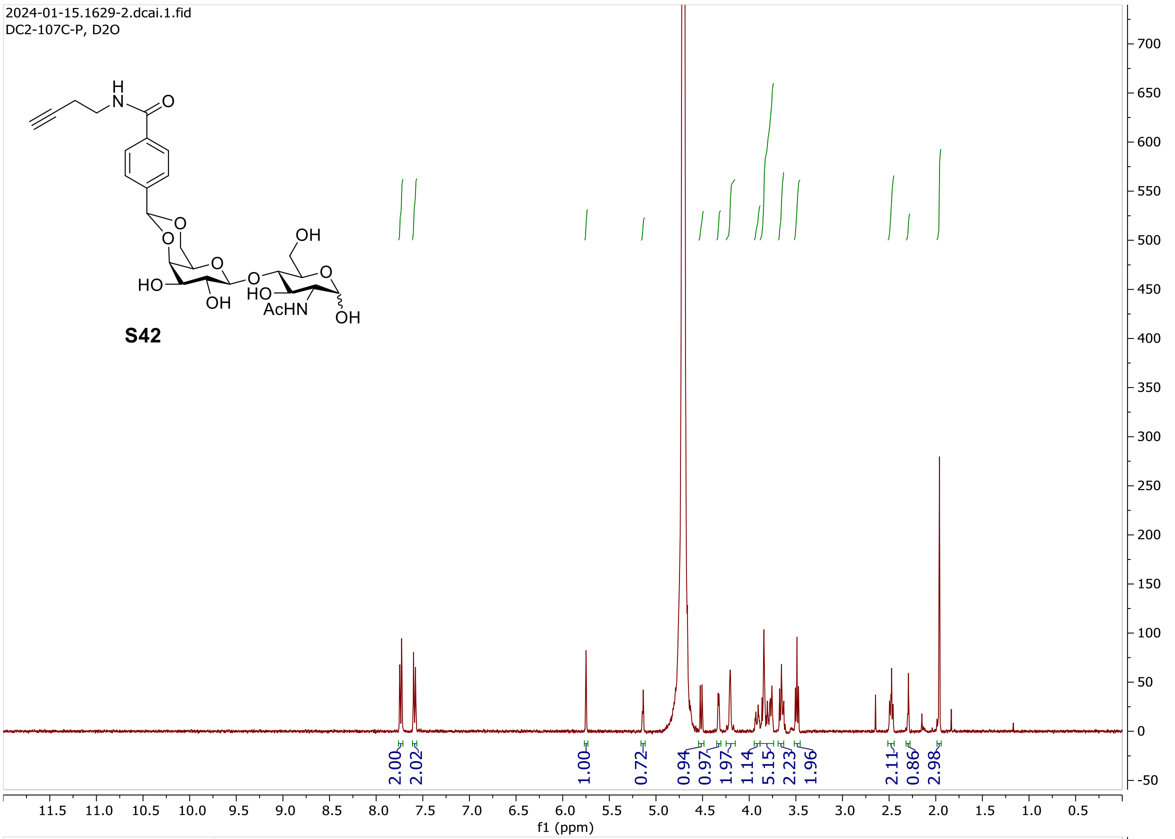
2024-01-15.1629-2.dcai.1.fid
DC2-107C-P, D2O
S42
OH
HO
OH
HO
AcHN
OH
2.00
2.02
1.00
0.72
0.94
0.97
1.97
1.14
5.15
2.23
1.96
2.11
0.86
2.98
f1 (ppm)

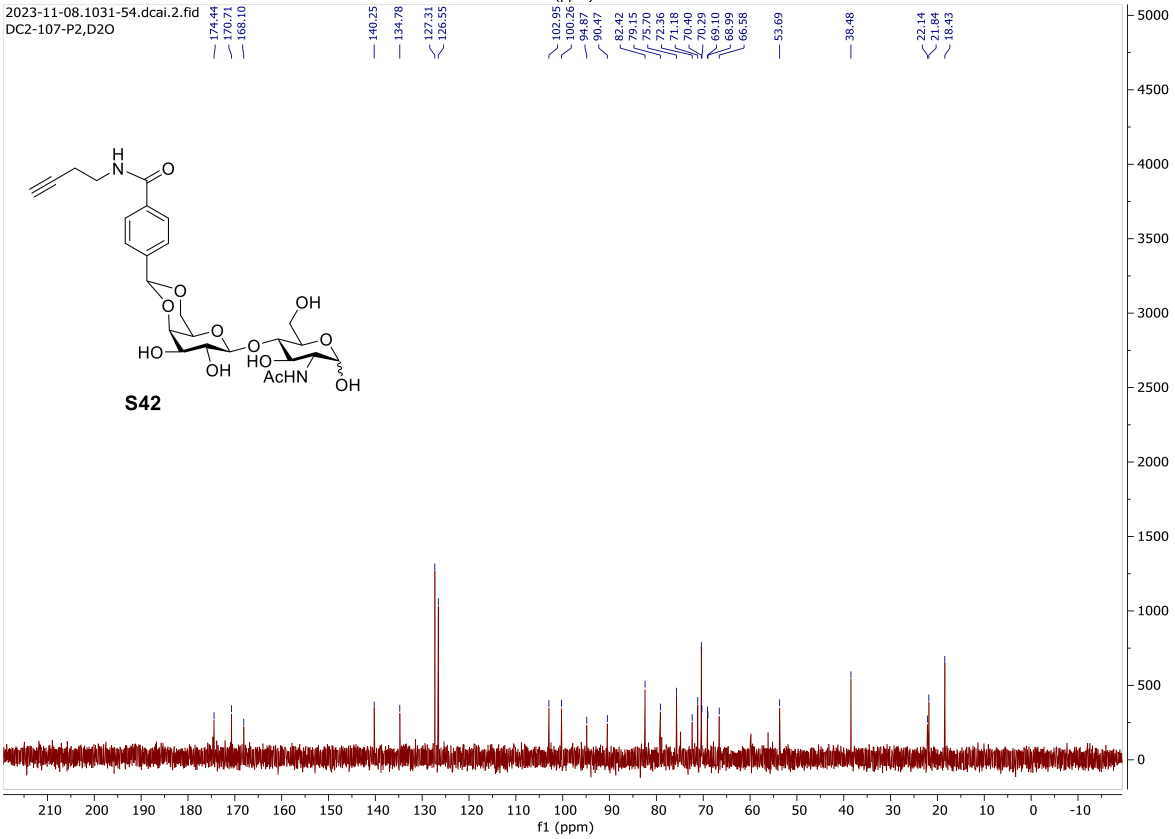
2023-11-08.1031-54.dcai.2.fid
DC2-107-P2,D2O
174.44
170.71
168.10
140.25
134.78
127.31
126.55
102.95
100.26
94.87
90.47
82.42
79.15
75.70
72.36
71.18
70.40
70.29
69.10
68.99
66.58
53.69
38.48
22.14
21.84
18.43
S42
OH
HO
OH
HO
AcHN
OH
f1 (ppm)

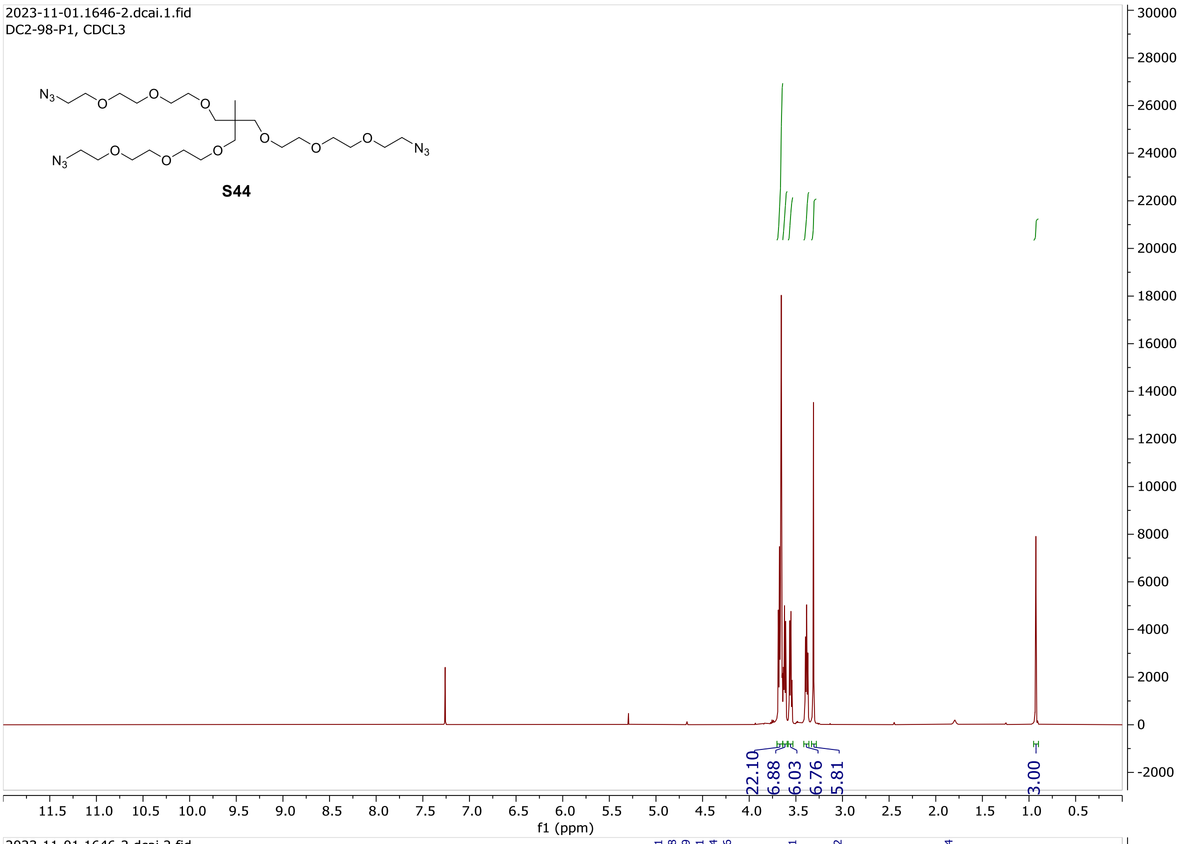
2023-11-01.1646-2.dcai.1.fid
DC2-98-P1, CDCL3
S44
22.10
6.88
6.03
6.76
5.81
3.00
f1 (ppm)

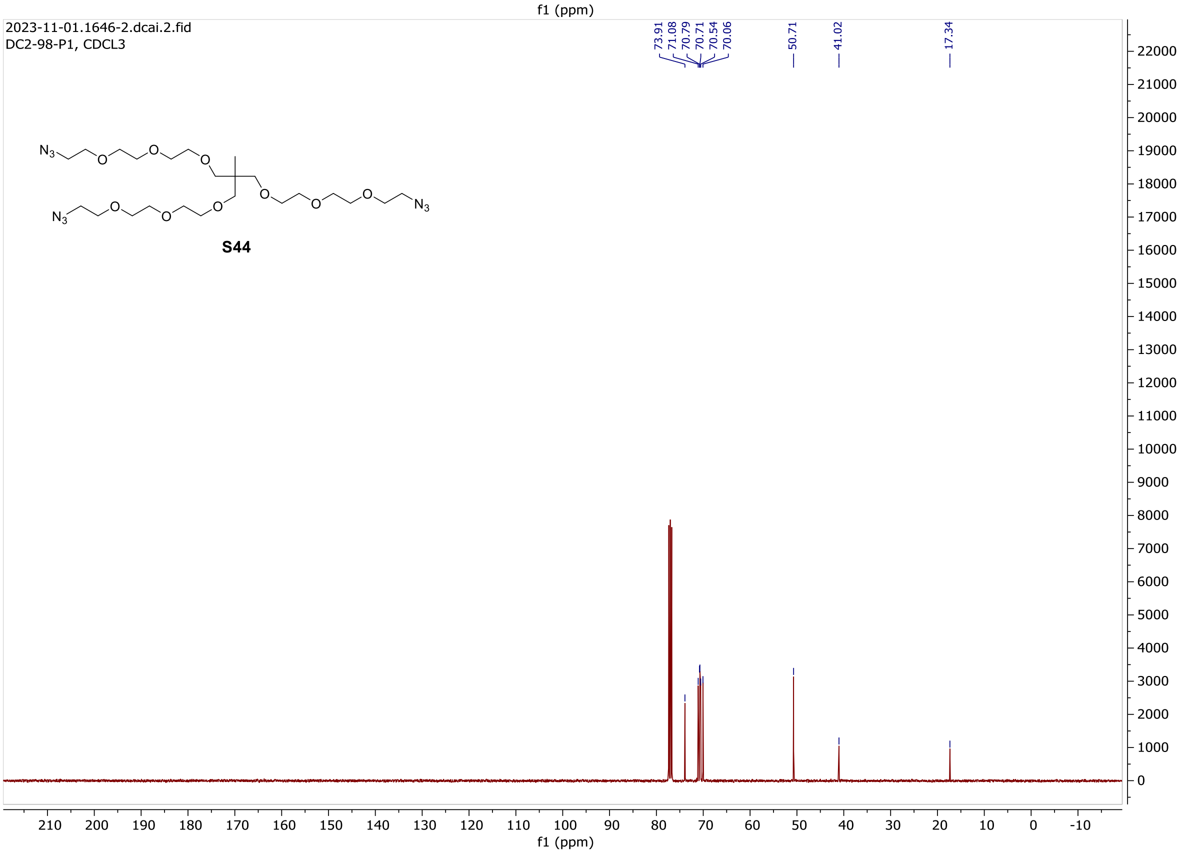
2023-11-01.1646-2.dcai.2.fid
DC2-98-P1, CDCL3
73.91
71.08
70.79
70.71
70.54
70.06
50.71
41.02
17.34
S44
f1 (ppm)

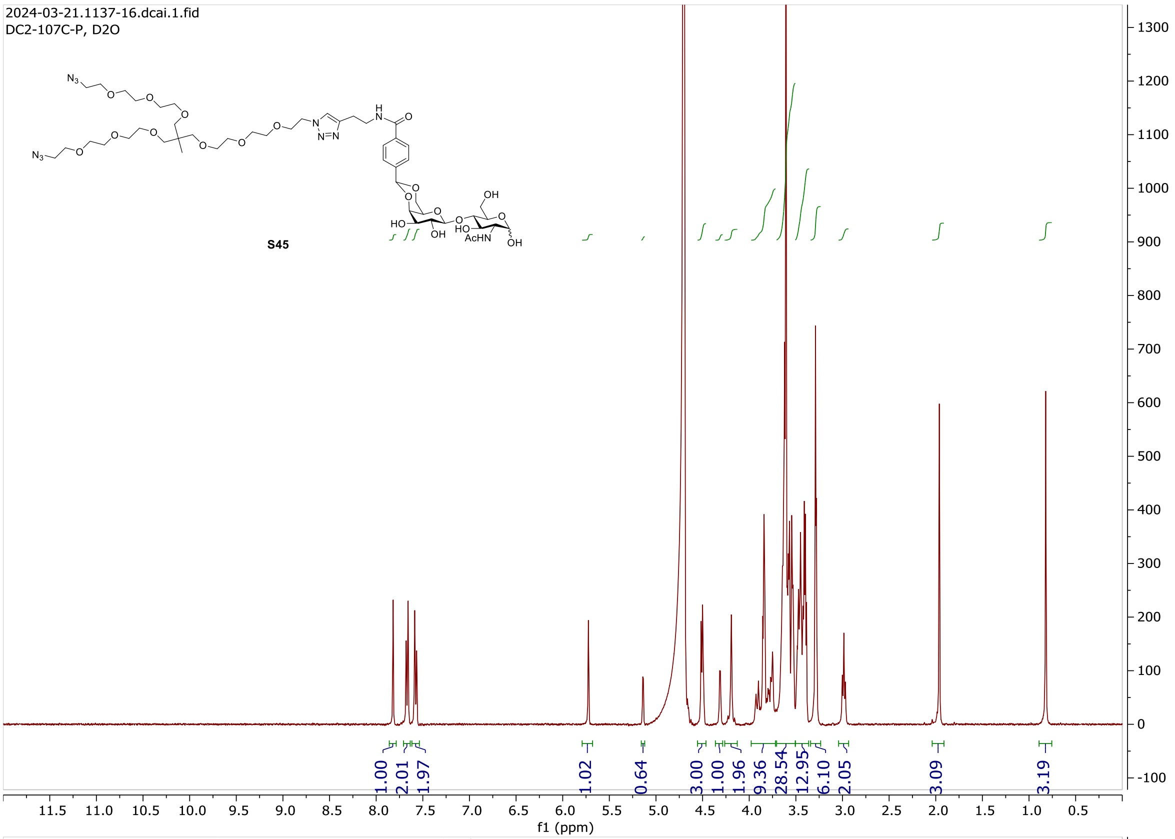
2024-03-21.1137-16.dcai.1.fid
DC2-107C-P, D2O
S45
1.00
2.01
1.97
1.02
0.64
3.00
1.00
1.96
9.36
28.54
12.95
6.10
2.05
3.09
3.19
f1 (ppm)

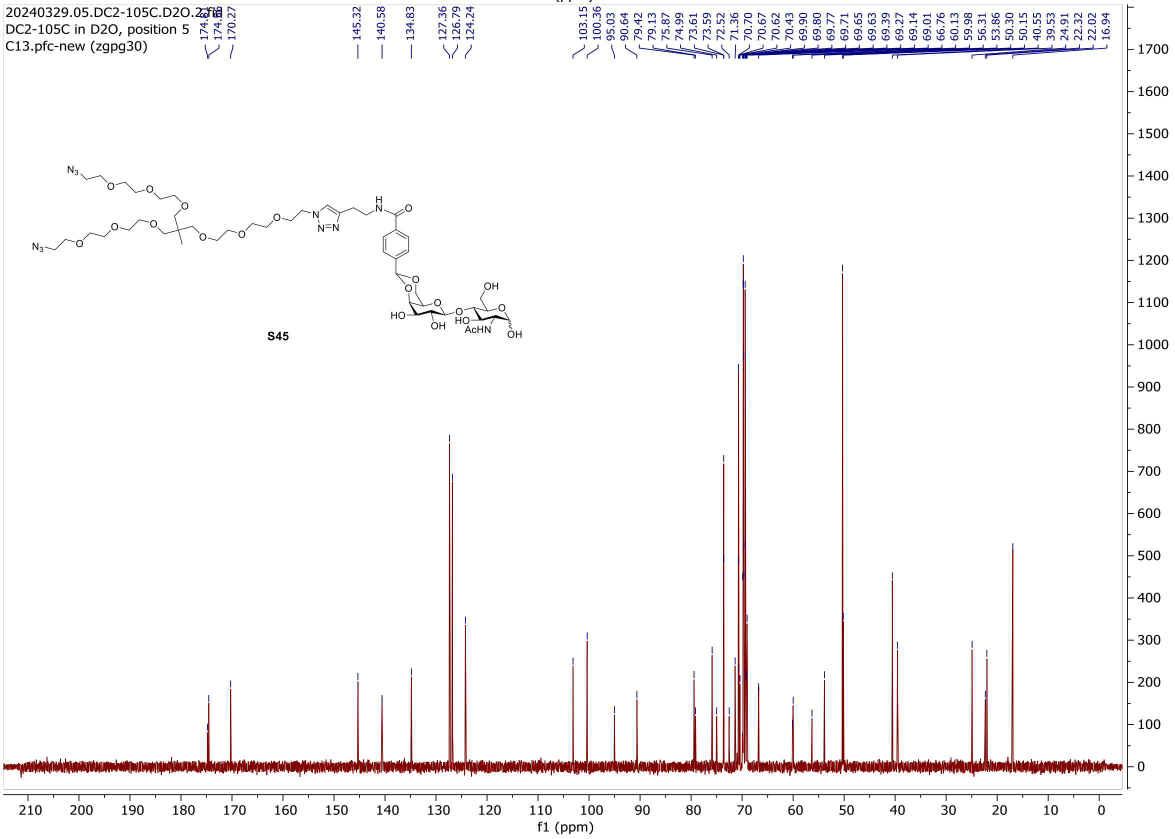
20240329.05.DC2-105C.D2O.2.fid
DC2-105C in D2O, position 5
C13.pfc-new (zgpg30)
170.27
145.32
140.58
134.83
127.36
126.79
124.24
103.15
100.36
95.03
90.64
79.42
79.13
75.87
74.99
73.61
73.59
72.52
71.36
70.70
70.67
70.62
70.43
69.90
69.80
69.77
69.71
69.65
69.63
69.39
69.27
69.14
69.01
66.76
60.13
59.98
56.31
53.86
50.30
50.15
40.55
39.53
24.91
22.32
22.02
16.94
S45
f1 (ppm)

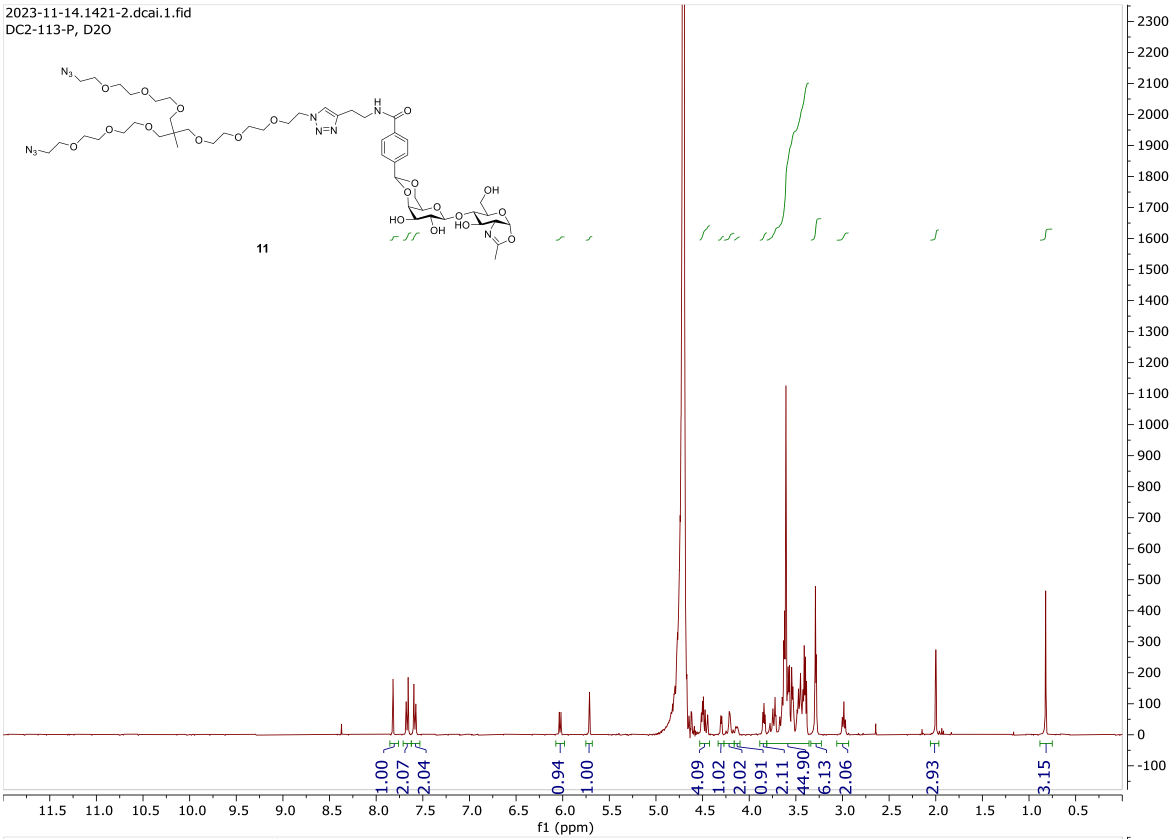
2023-11-14.1421-2.dcai.1.fid
DC2-113-P, D2O
11
1.00
2.07
2.04
0.94
1.00
4.09
1.02
2.02
0.91
2.11
44.90
6.13
2.06
2.93
3.15
f1 (ppm)

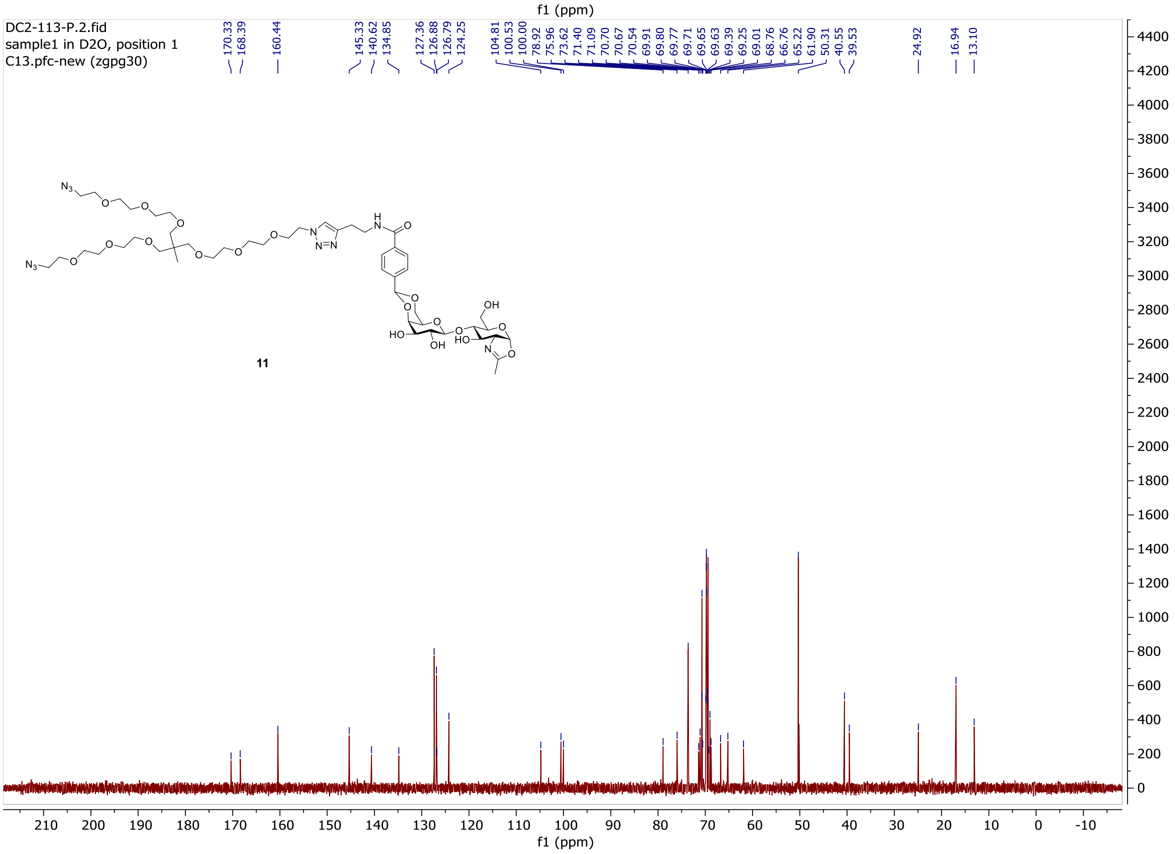
DC2-113-P.2.fid
sample1 in D2O, position 1
C13.pfc-new (zgpg30)
170.33
168.39
160.44
145.33
140.62
134.85
127.36
126.88
126.79
124.25
104.81
100.53
100.00
78.92
75.96
73.62
71.40
71.09
70.70
70.67
70.54
69.91
69.80
69.77
69.71
69.65
69.63
69.39
69.25
69.01
68.76
66.76
65.22
61.90
50.31
40.55
39.53
24.92
16.94
13.10
11
f1 (ppm)

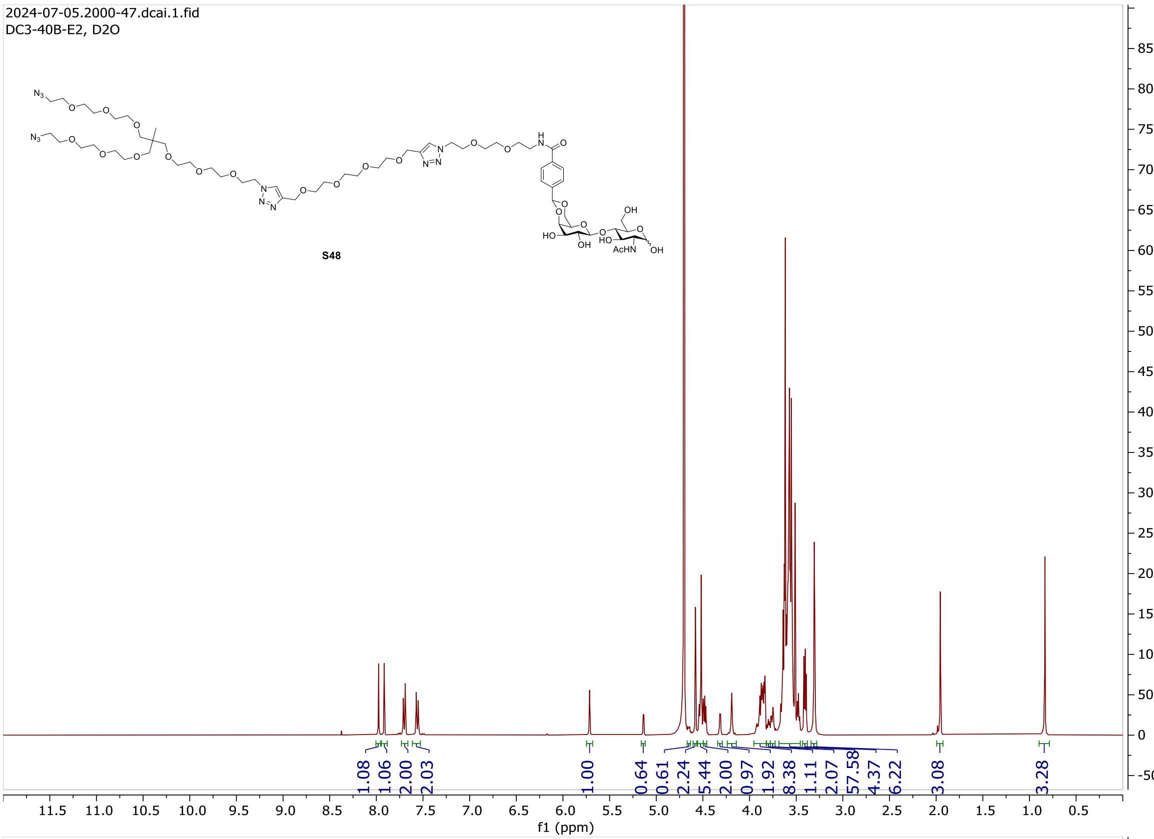
2024-07-05.2000-47.dcai.1.fid
DC3-40B-E2, D2O
S48
1.08
1.06
2.00
2.03
1.00
0.64
0.61
2.24
5.44
2.00
0.97
1.92
8.38
1.11
2.07
57.58
4.37
6.22
3.08
3.28
f1 (ppm)

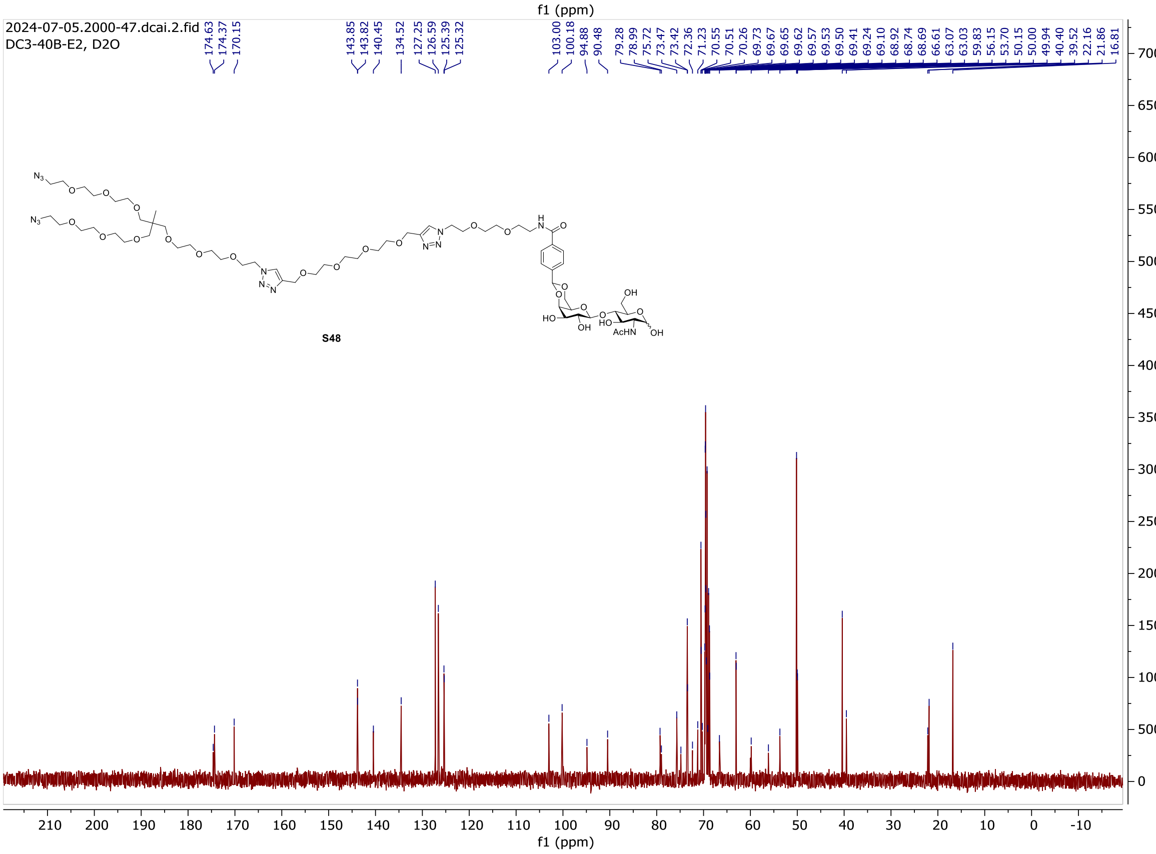
2024-07-05.2000-47.dcai.2.fid
DC3-40B-E2, D2O
174.63
174.37
170.15
143.85
143.82
140.45
134.52
127.25
126.59
125.39
125.32
103.00
100.18
94.88
90.48
79.28
78.99
75.72
73.47
73.42
72.36
71.23
70.55
70.51
70.26
69.73
69.67
69.65
69.62
69.57
69.53
69.50
69.41
69.24
69.10
68.92
68.74
68.69
66.61
63.07
63.03
59.83
56.15
53.70
50.15
50.00
49.94
40.40
39.52
22.16
21.86
16.81
S48
f1 (ppm)

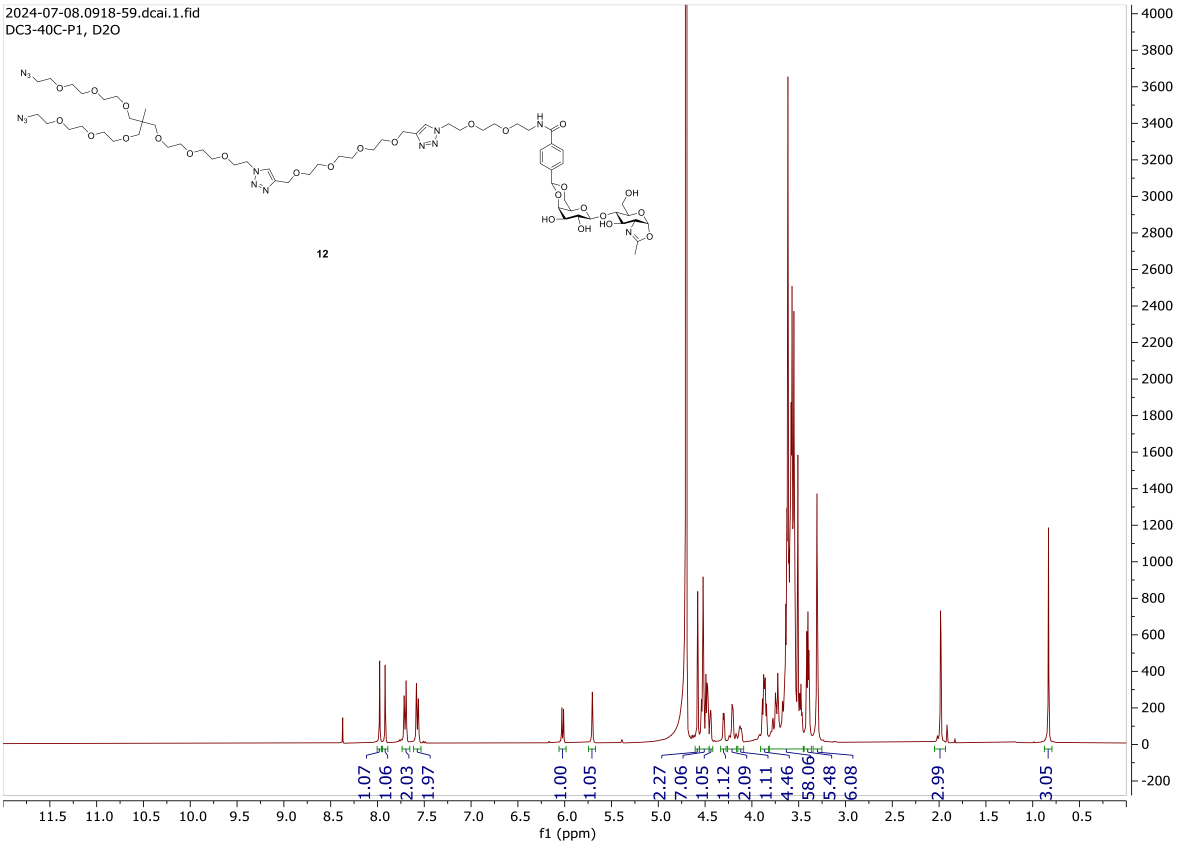
2024-07-08.0918-59.dcai.1.fid
DC3-40C-P1, D2O
12
f1 (ppm)

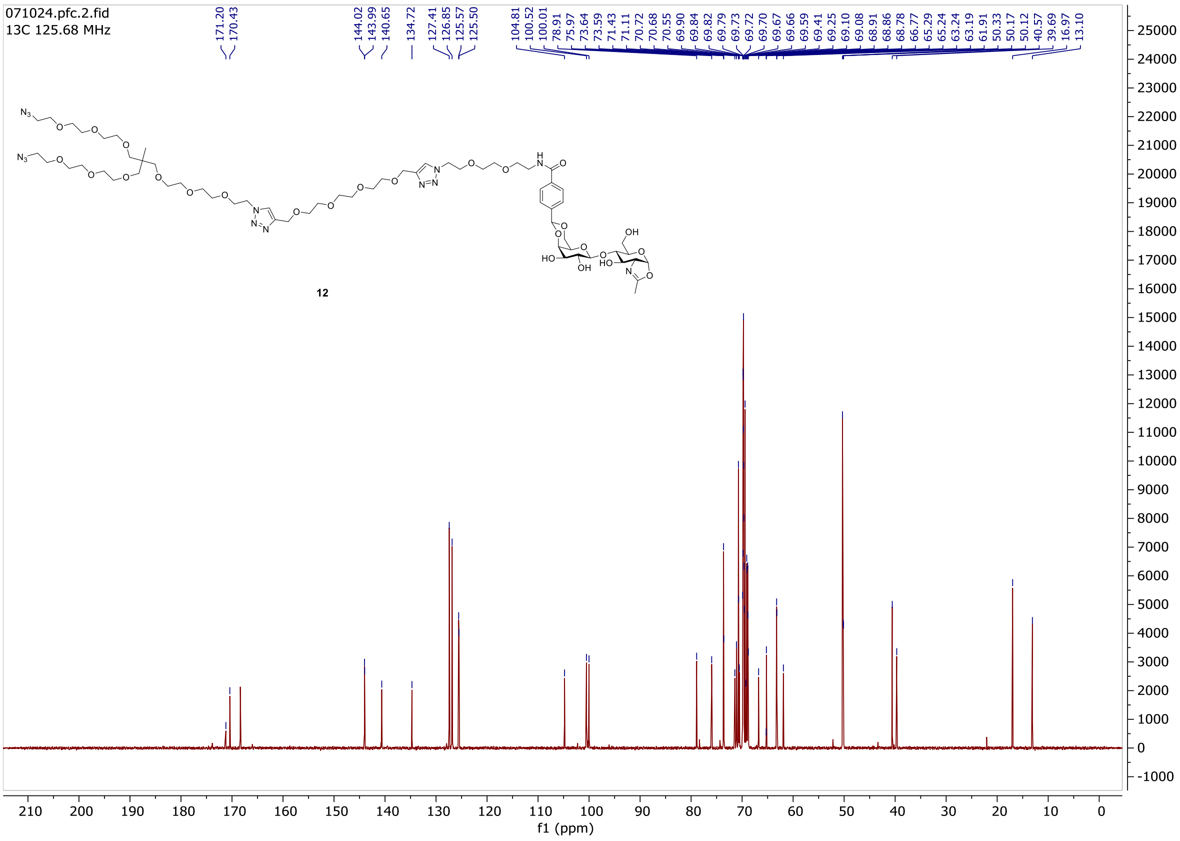
071024.pfc.2.fid
13C 125.68 MHz
12
f1 (ppm)

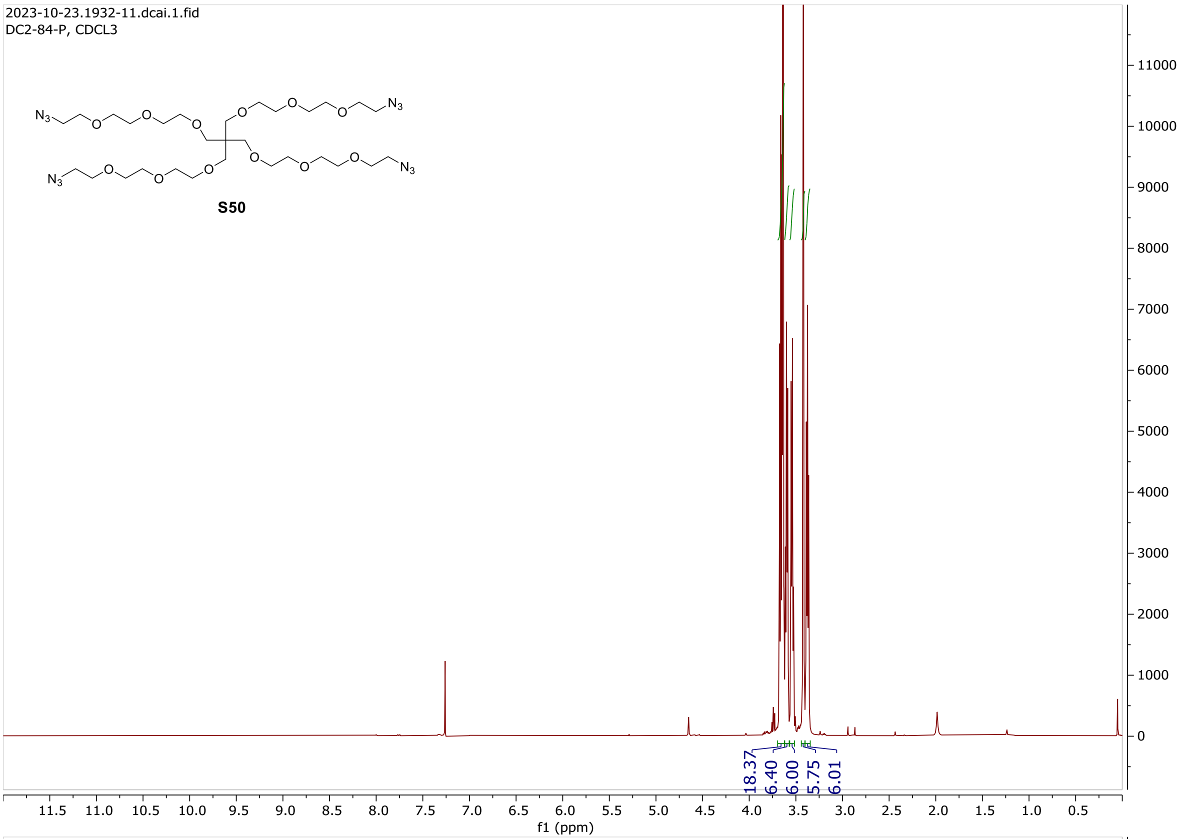
2023-10-23.1932-11.dcai.1.fid
DC2-84-P, CDCL3
N3
N3
N3
N3
S50
18.37
6.40
6.00
5.75
6.01
f1 (ppm)

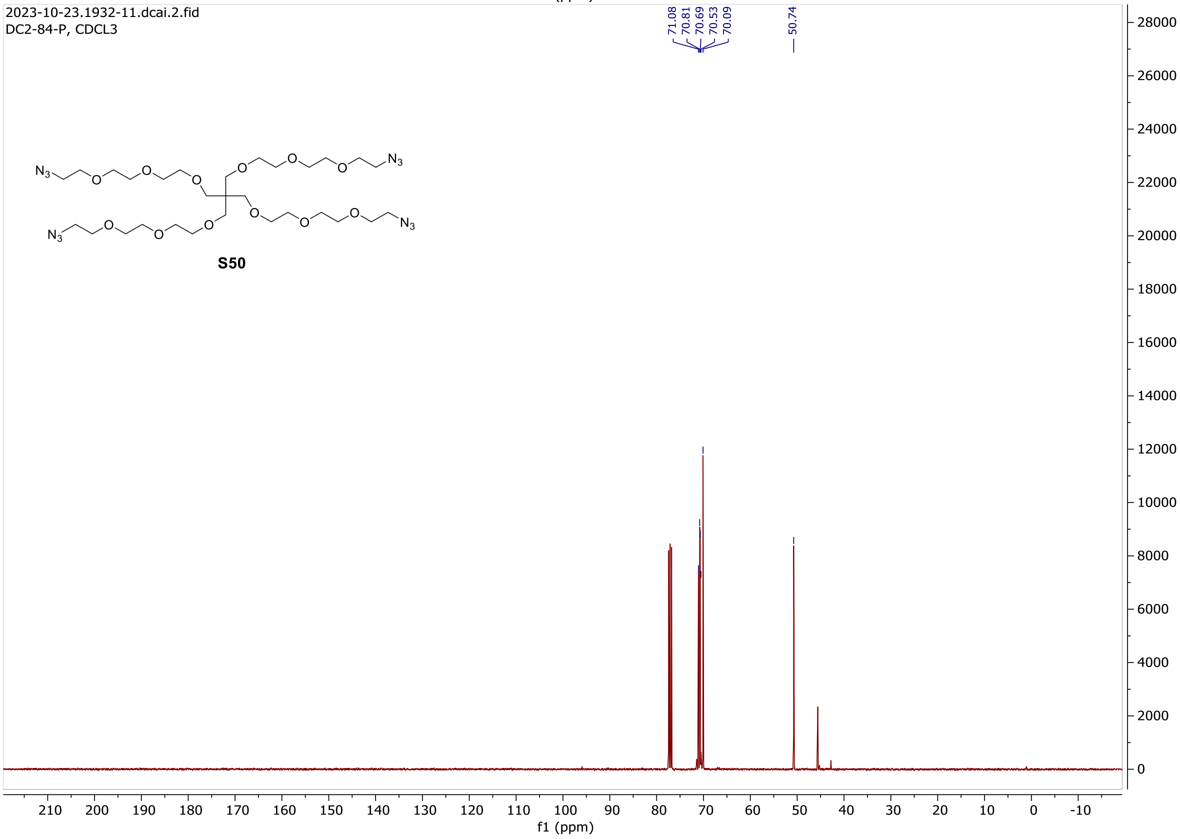
2023-10-23.1932-11.dcai.2.fid
DC2-84-P, CDCL3
71.08
70.81
70.69
70.53
70.09
50.74
S50
f1 (ppm)

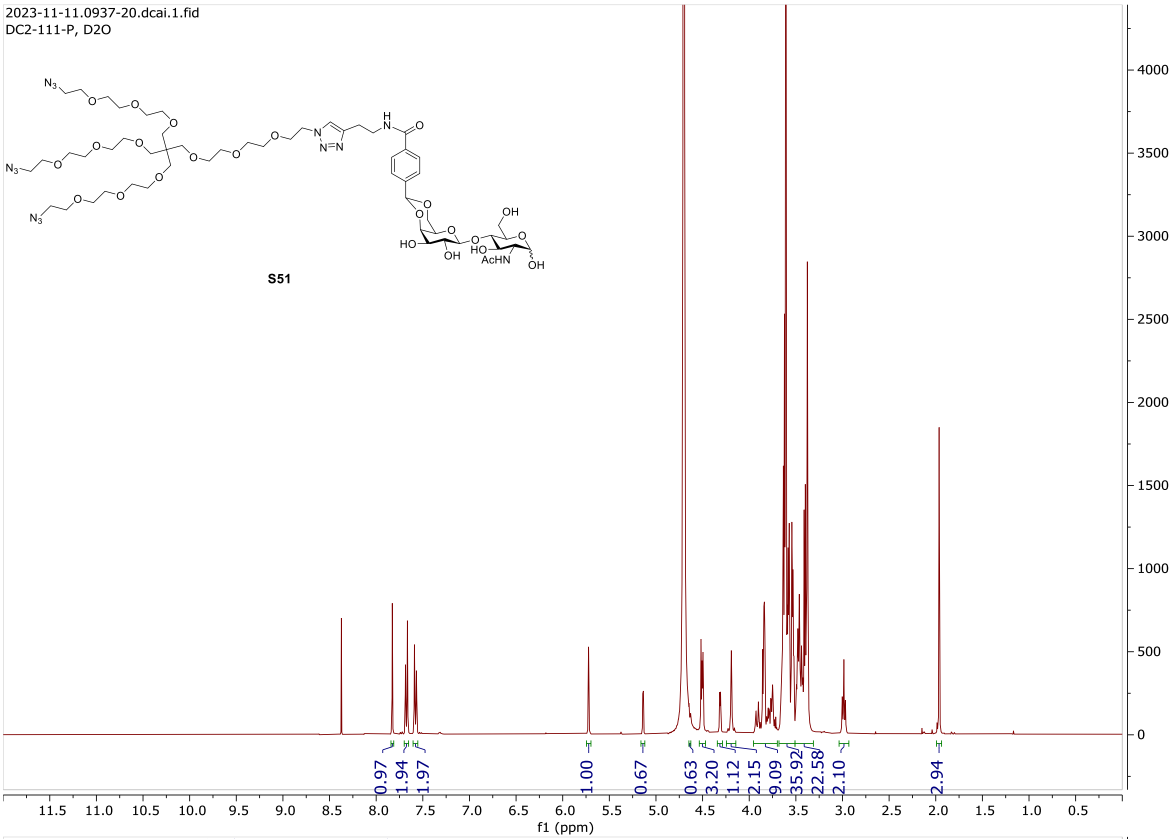
2023-11-11.0937-20.dcai.1.fid
DC2-111-P, D2O
S51
0.97
1.94
1.97
1.00
0.67
0.63
3.20
1.12
2.15
9.09
35.92
22.58
2.10
2.94
f1 (ppm)

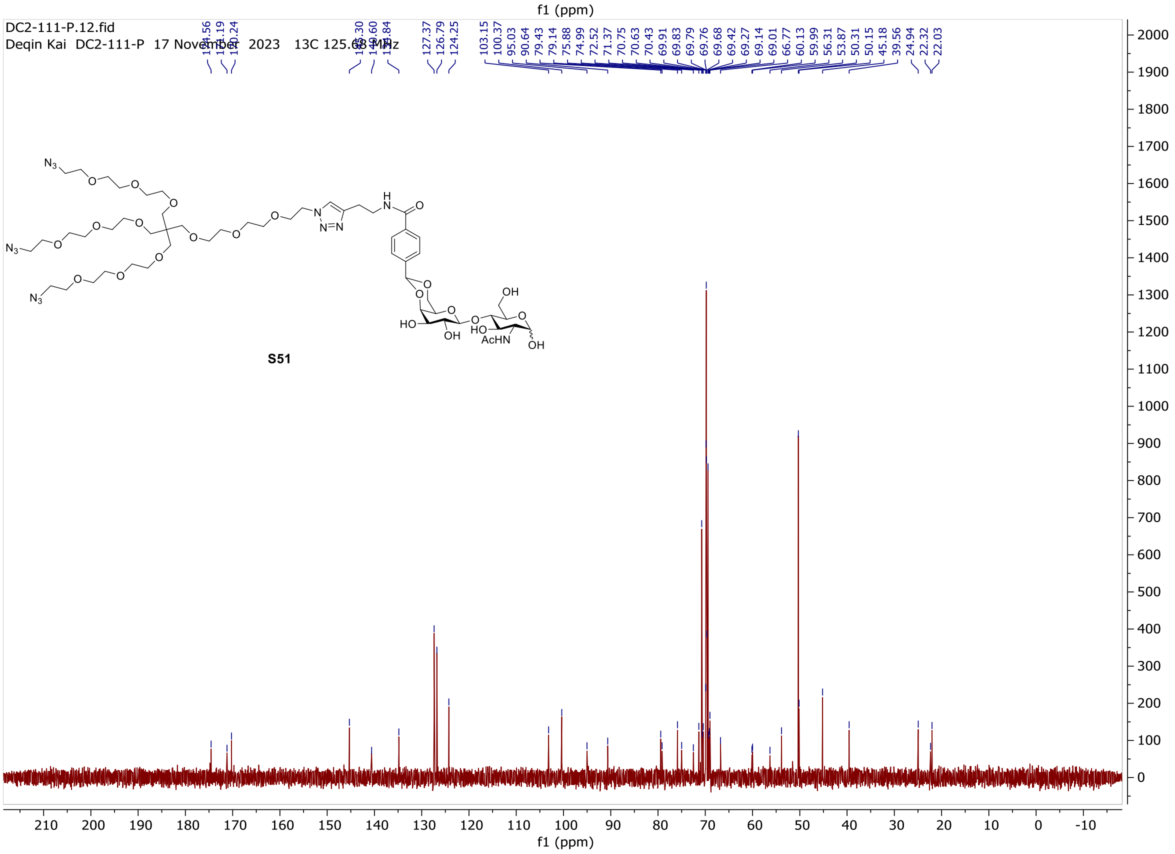
DC2-111-P.12.fid
Deqin Kai DC2-111-P 17 November 2023 13C 125.68 MHz
145.30
140.60
134.84
127.37
126.79
124.25
103.15
100.37
95.03
90.64
79.43
79.14
75.88
74.99
72.52
71.37
70.75
70.63
70.43
69.91
69.83
69.79
69.76
69.68
69.42
69.27
69.14
69.01
66.77
60.13
59.99
56.31
53.87
50.31
50.15
45.18
39.56
24.94
22.32
22.03
S51
f1 (ppm)

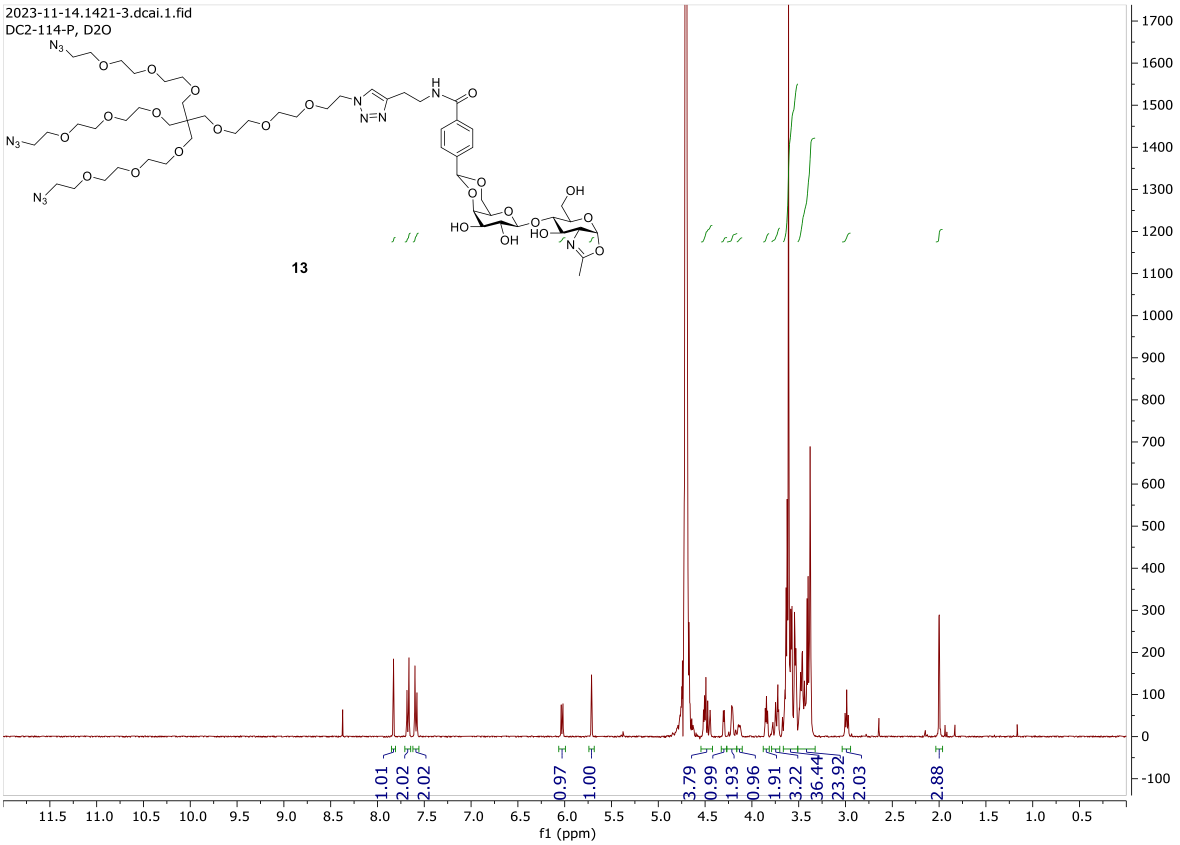
2023-11-14.1421-3.dcai.1.fid
DC2-114-P, D2O
13
1.01
2.02
2.02
0.97
1.00
3.79
0.99
1.93
0.96
1.91
3.22
36.44
23.92
2.03
2.88
f1 (ppm)

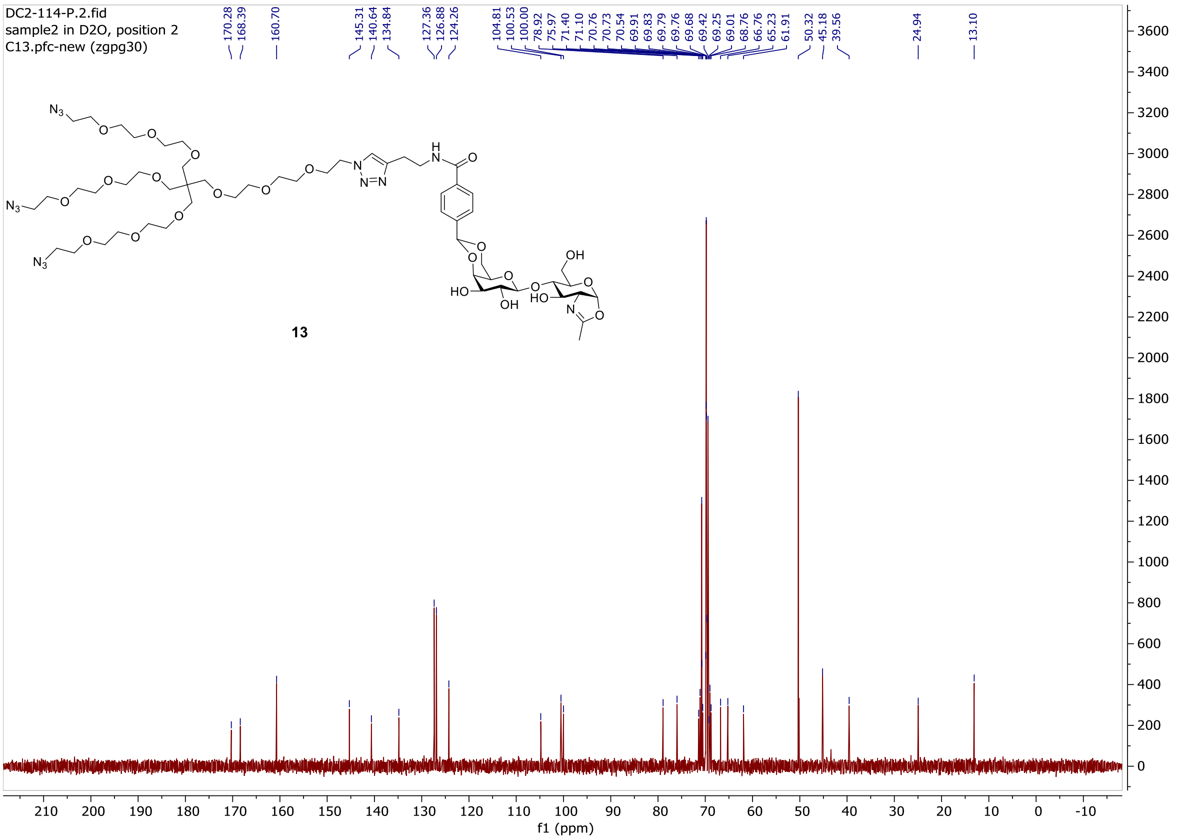
DC2-114-P.2.fid
sample2 in D2O, position 2
C13.pfc-new (zgpg30)
170.28
168.39
160.70
145.31
140.64
134.84
127.36
126.88
124.26
104.81
100.53
100.00
78.92
75.97
71.40
71.10
70.76
70.73
70.54
69.91
69.83
69.79
69.76
69.68
69.42
69.25
69.01
68.76
66.76
65.23
61.91
50.32
45.18
39.56
24.94
13.10
13
f1 (ppm)

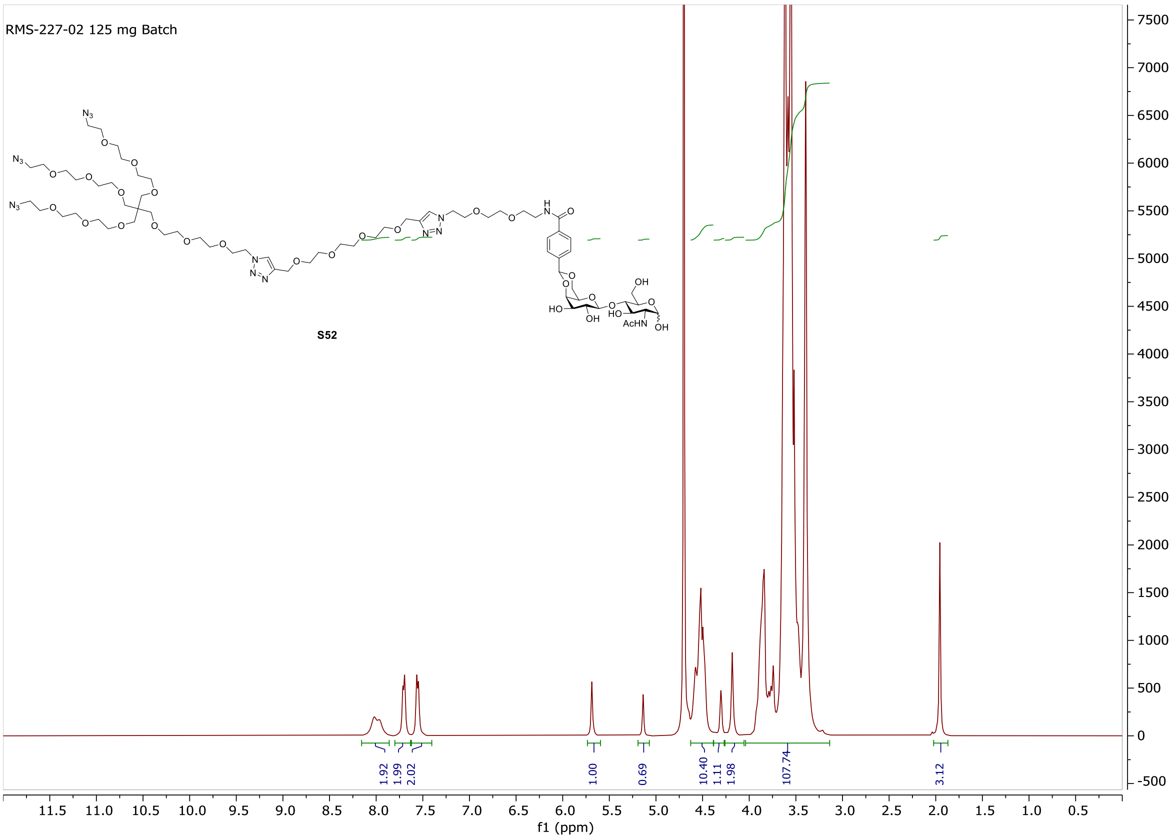
RMS-227-02 125 mg Batch
S52
1.92
1.99
2.02
1.00
0.69
10.40
1.11
1.98
107.74
3.12
f1 (ppm)

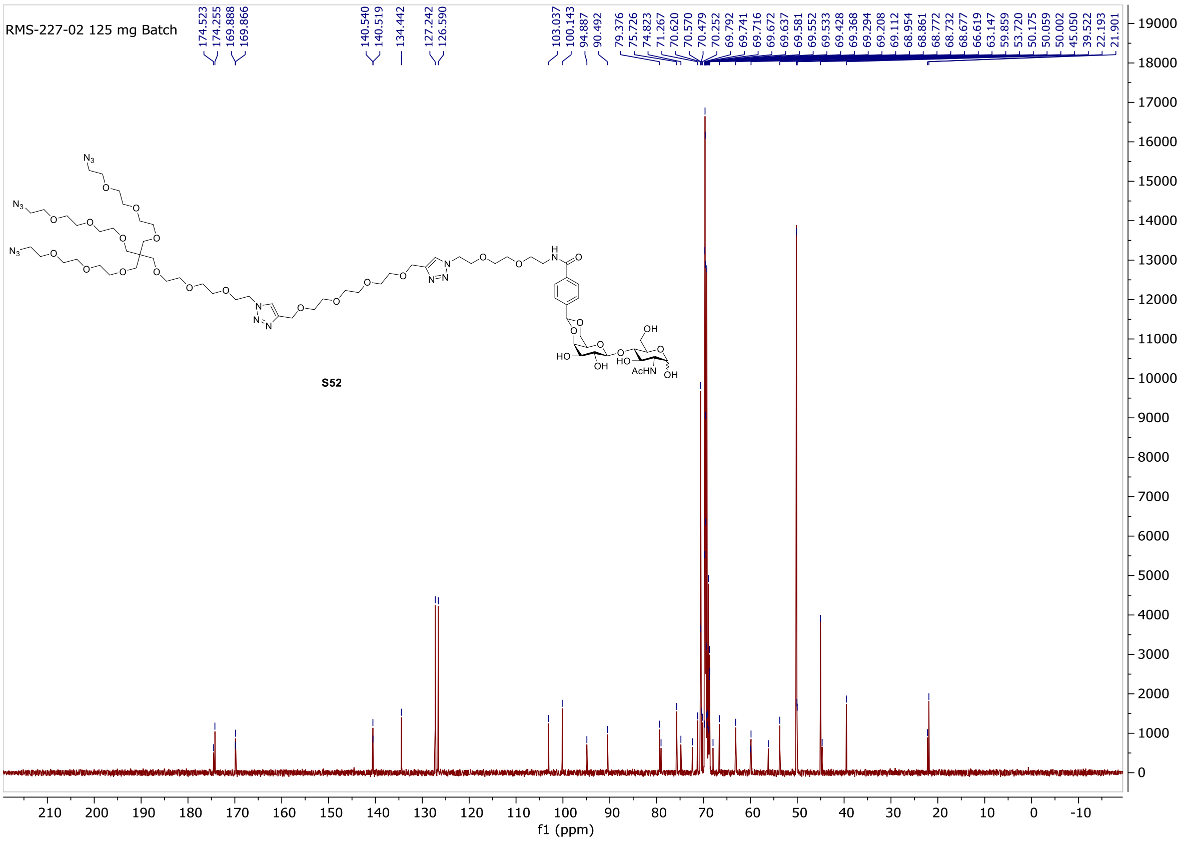
RMS-227-02 125 mg Batch
174.523
174.255
169.888
169.866
140.540
140.519
134.442
127.242
126.590
103.037
100.143
94.887
90.492
79.376
75.726
74.823
71.267
70.620
70.570
70.479
70.252
69.792
69.741
69.716
69.672
69.637
69.581
69.552
69.533
69.428
69.368
69.294
69.208
69.112
68.954
68.861
68.772
68.732
68.677
66.619
63.147
59.859
53.720
50.175
50.059
50.002
45.050
39.522
22.193
21.901
S52
f1 (ppm)

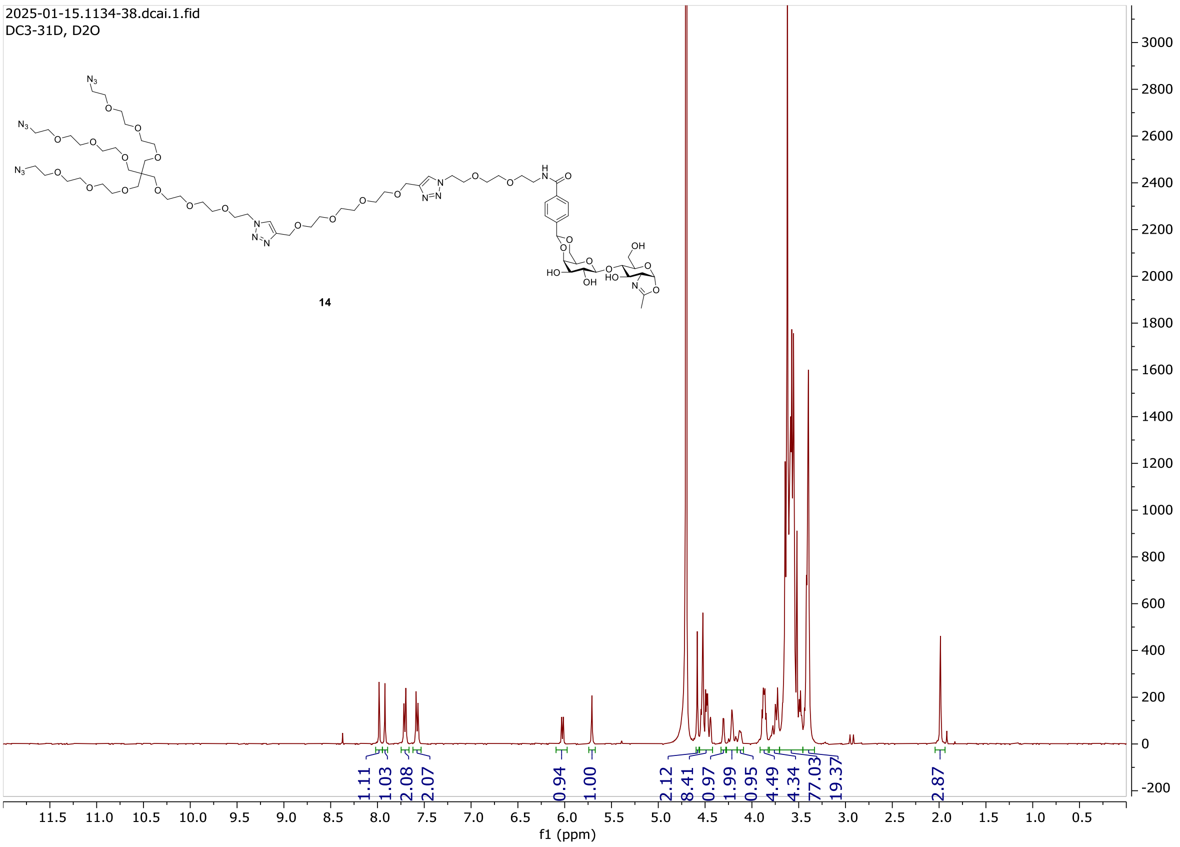
2025-01-15.1134-38.dcai.1.fid
DC3-31D, D2O
14
f1 (ppm)

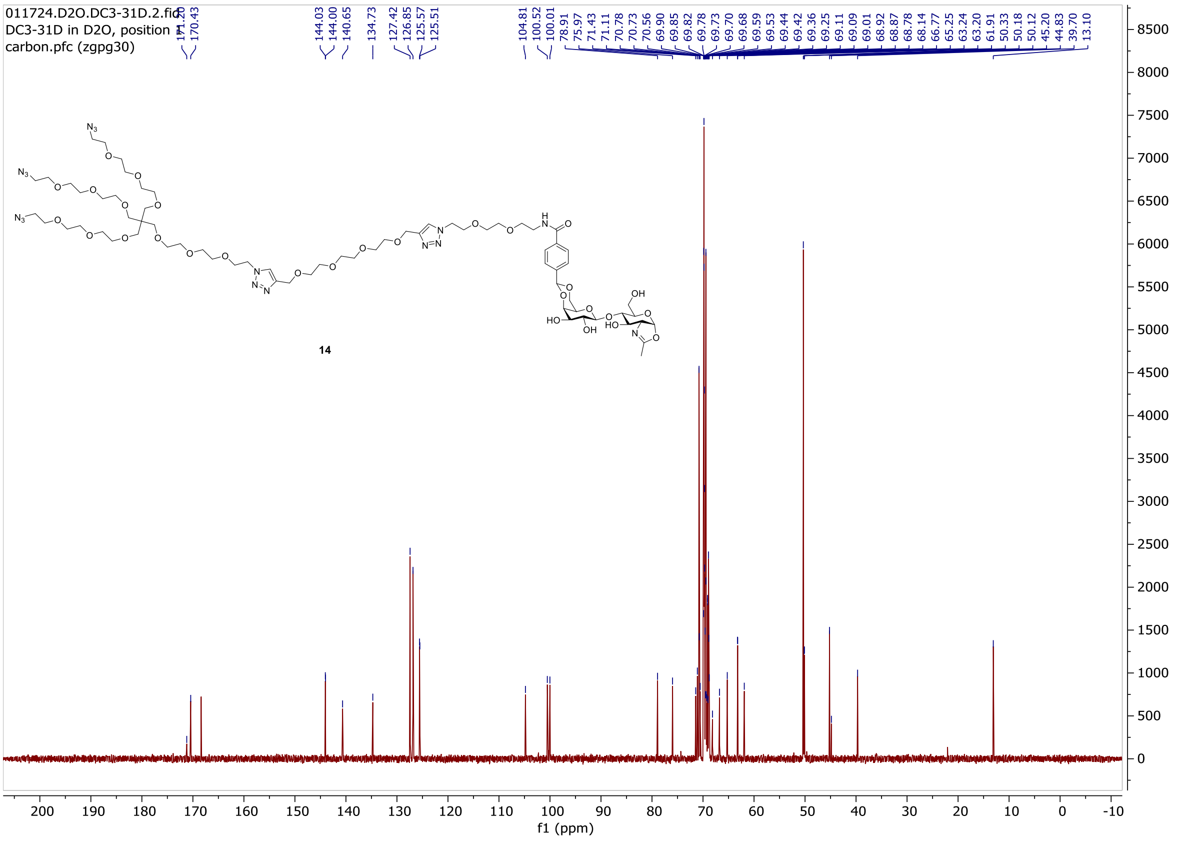
011724.D2O.DC3-31D.2.fid
DC3-31D in D2O, position 1
carbon.pfc (zgpg30)
14
f1 (ppm)

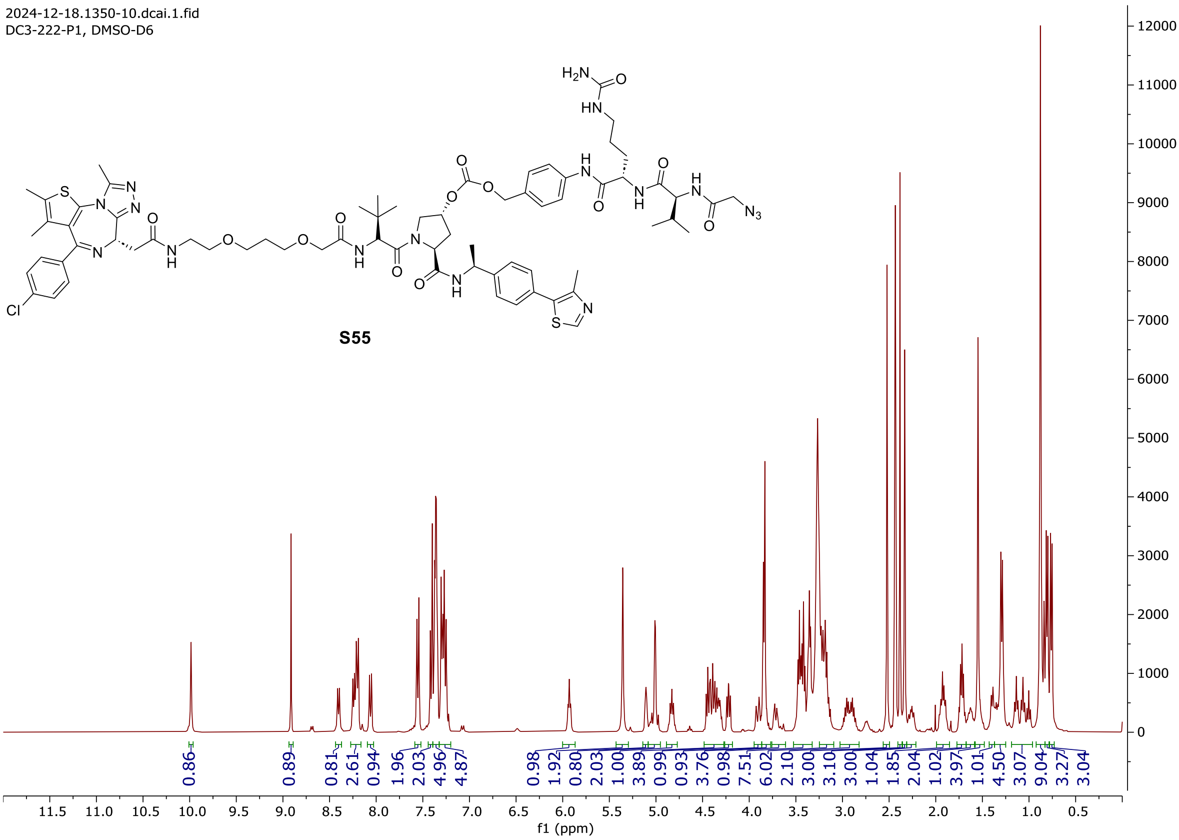
2024-12-18.1350-10.dcai.1.fid
DC3-222-P1, DMSO-D6
S55
0.86 0.89 0.81 2.61 0.94 1.96 2.03 4.96 4.87 0.98 1.92 0.80 2.03 1.00 3.89 0.99 0.93 3.76 0.98 7.51 6.02 2.10 3.00 3.10 3.00 1.04 1.85 2.04 1.02 3.97 1.01 4.50 3.07 9.04 3.27 3.04
f1 (ppm)

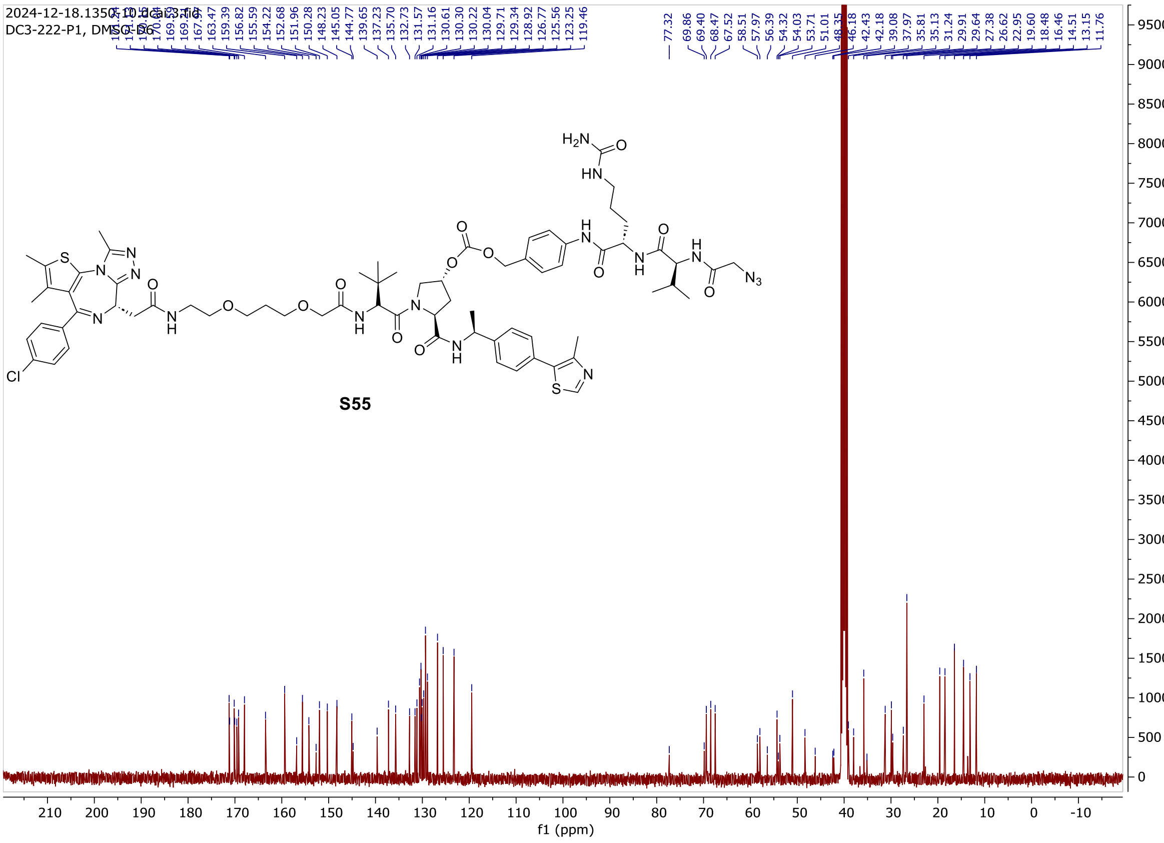
DC3-222-P1, DMSO-D6
163.47 159.39 156.82 155.59 154.22 152.68 151.96 150.28 148.23 145.05 144.77 139.65 137.23 135.70 132.73 131.57 131.16 130.61 130.30 130.22 130.04 129.71 129.34 128.92 126.77 125.56 123.25 119.46
77.32 69.86 69.40 68.47 67.52 58.51 57.97 56.39 54.32 54.03 53.71 51.01 46.18 42.43 42.18 39.08 37.97 35.81 35.13 31.24 29.91 29.64 27.38 26.62 22.95 19.60 18.48 16.46 14.51 13.15 11.76
S55
f1 (ppm)

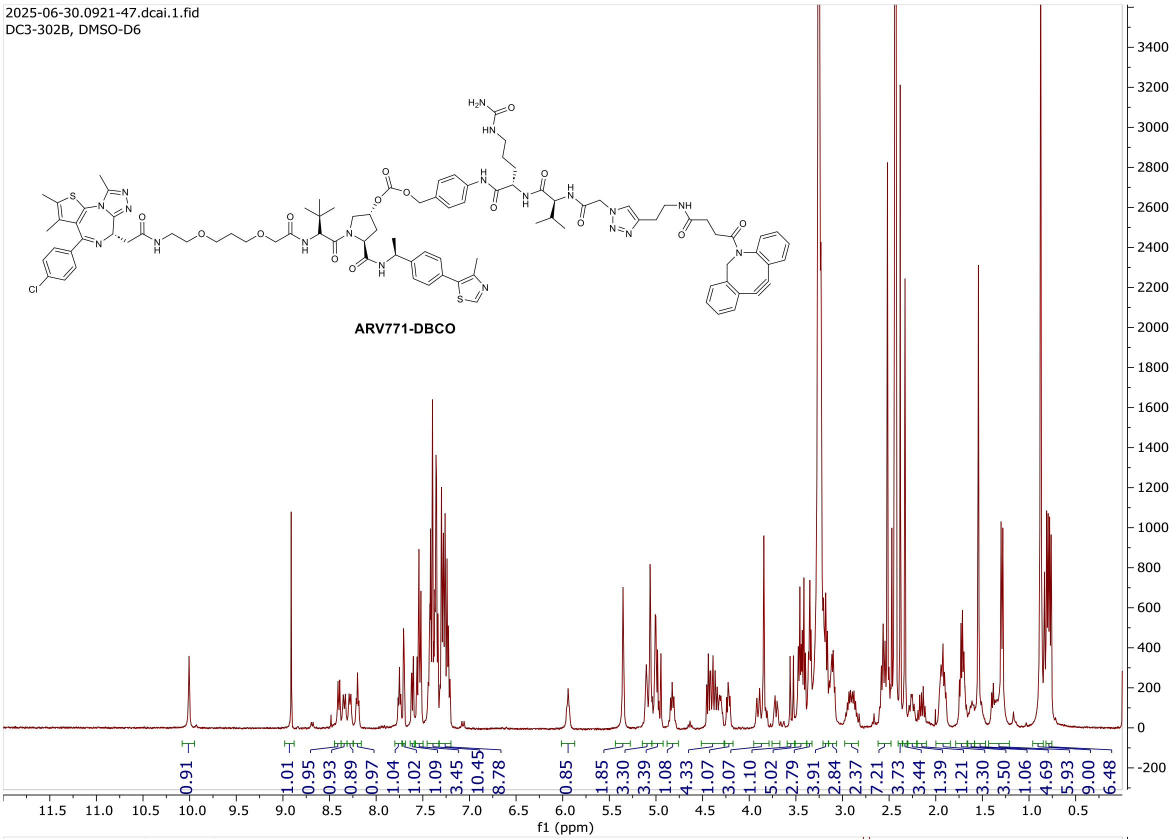
2025-06-30.0921-47.dcai.1.fid
DC3-302B, DMSO-D6
ARV771-DBCO
f1 (ppm)

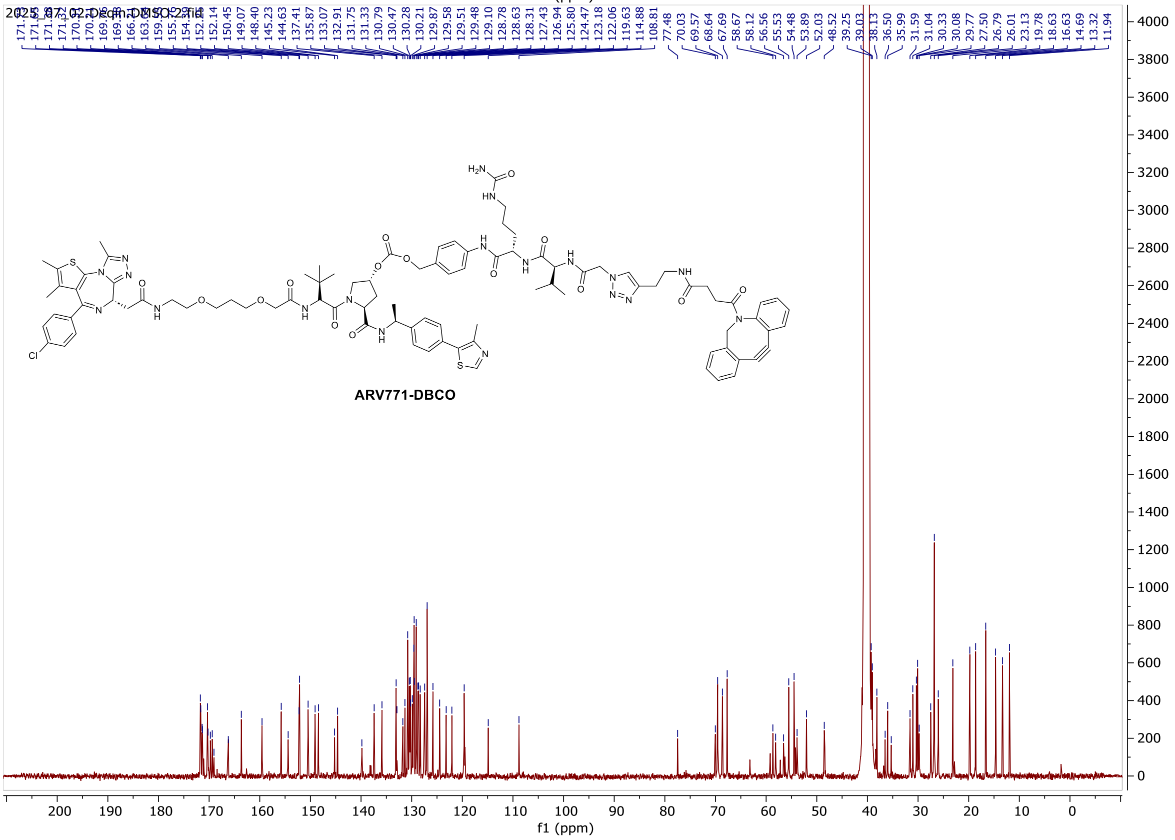
ARV771-DBCO
f1 (ppm)

HRMS

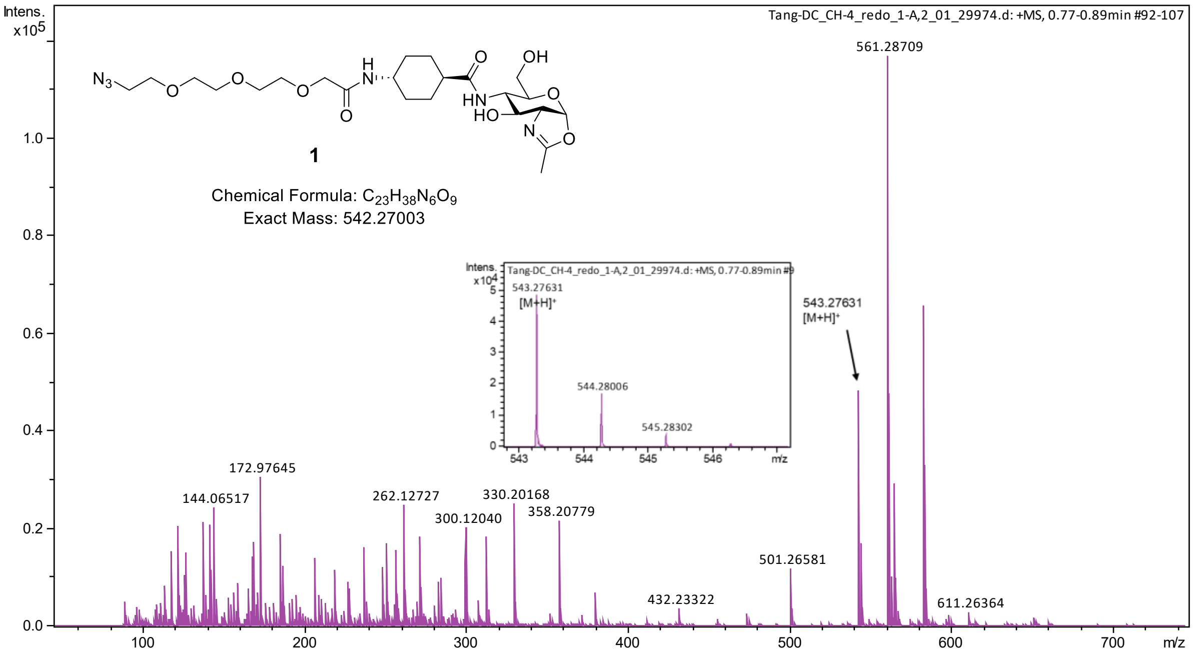

Tang-DC_CH-4_redo_1-A,2_01_29974.d: +MS, 0.77-0.89min #92-107
1
Chemical Formula: $C_{23}H_{38}N_6O_9$
Exact Mass: 542.27003
561.28709
543.27631
[M+H]+
544.28006
545.28302
172.97645
144.06517
262.12727
330.20168
300.12040
358.20779
501.26581
432.23322
611.26364
Intens. x10^5
m/z

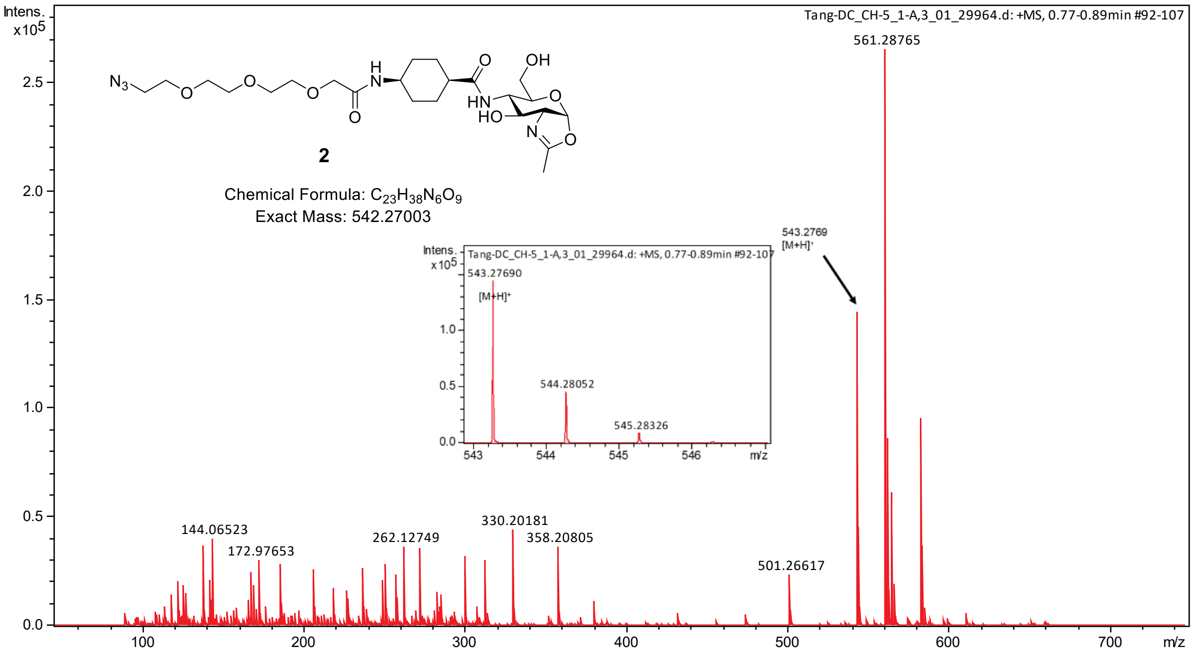

Tang-DC_CH-5_1-A,3_01_29964.d: +MS, 0.77-0.89min #92-107
2
Chemical Formula: $C_{23}H_{38}N_6O_9$
Exact Mass: 542.27003
561.28765
543.2769
[M+H]+
543.27690
544.28052
545.28326
144.06523
172.97653
262.12749
330.20181
358.20805
501.26617
Intens. x10^5
m/z

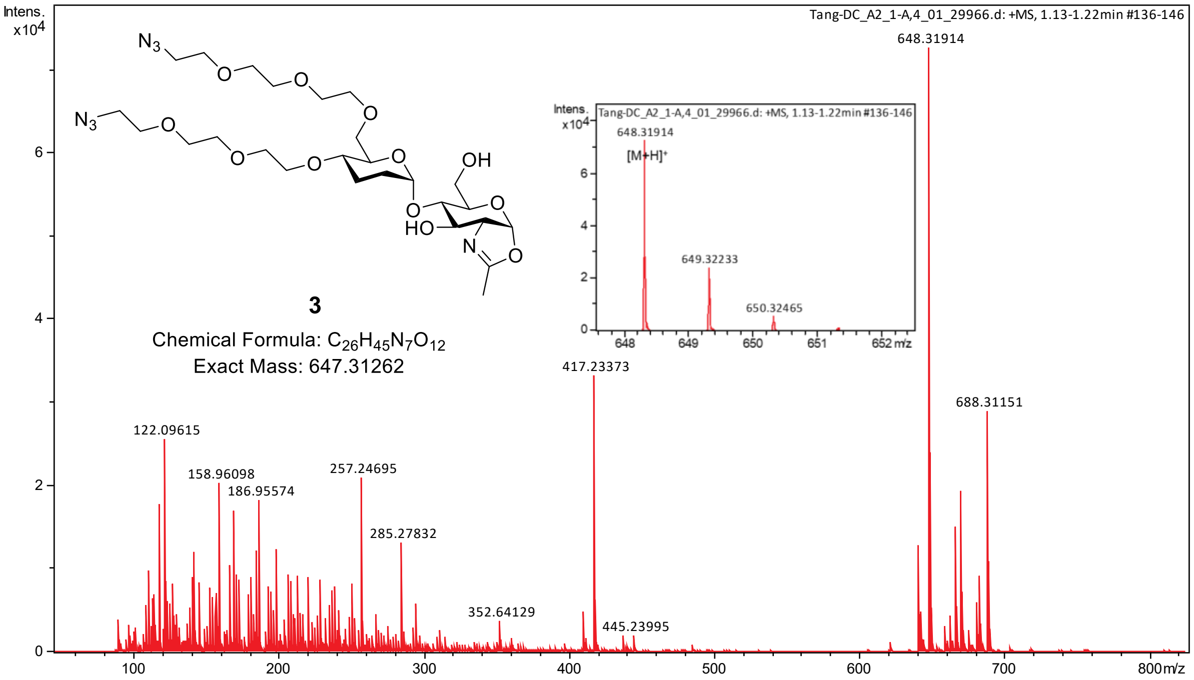
Intens.
x10^4
Tang-DC_A2_1-A,4_01_29966.d: +MS, 1.13-1.22min #136-146
648.31914
N3
OH
O
HO
N
3
Chemical Formula: C26H45N7O12
Exact Mass: 647.31262
648.31914
[M+H]+
649.32233
650.32465
417.23373
688.31151
122.09615
158.96098
186.95574
257.24695
285.27832
352.64129
445.23995
m/z

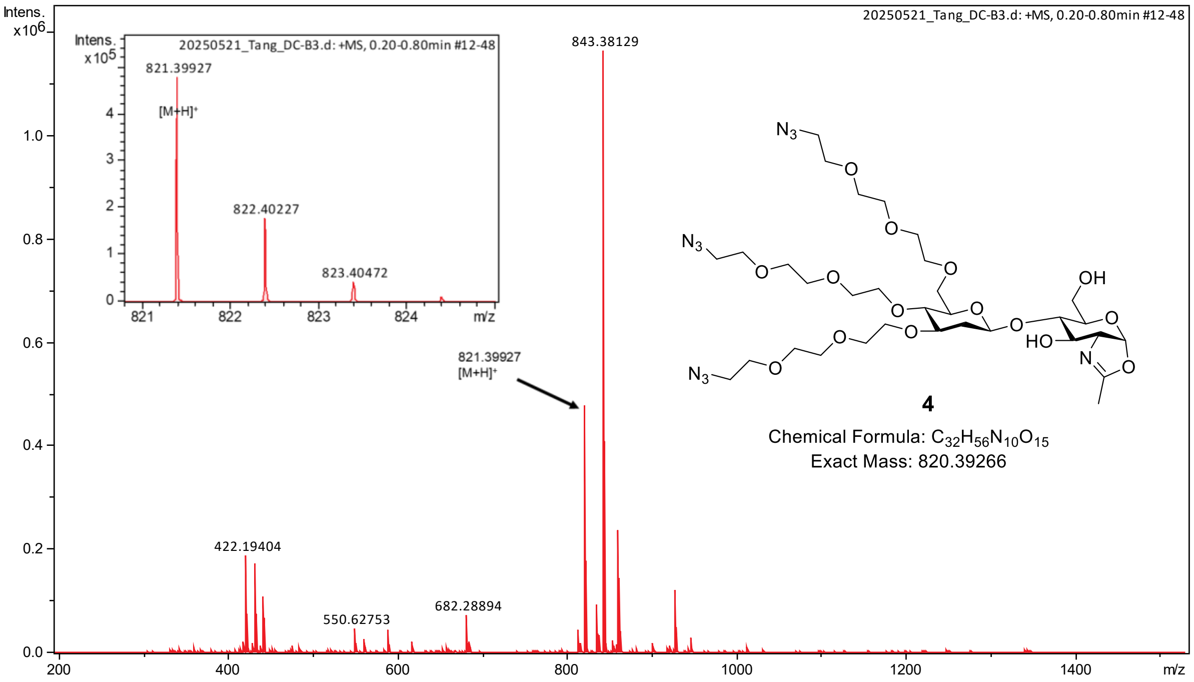
Intens.
x10^6
20250521_Tang_DC-B3.d: +MS, 0.20-0.80min #12-48
843.38129
821.39927
[M+H]+
822.40227
823.40472
821.39927
[M+H]+
4
Chemical Formula: C32H56N10O15
Exact Mass: 820.39266
422.19404
550.62753
682.28894
m/z

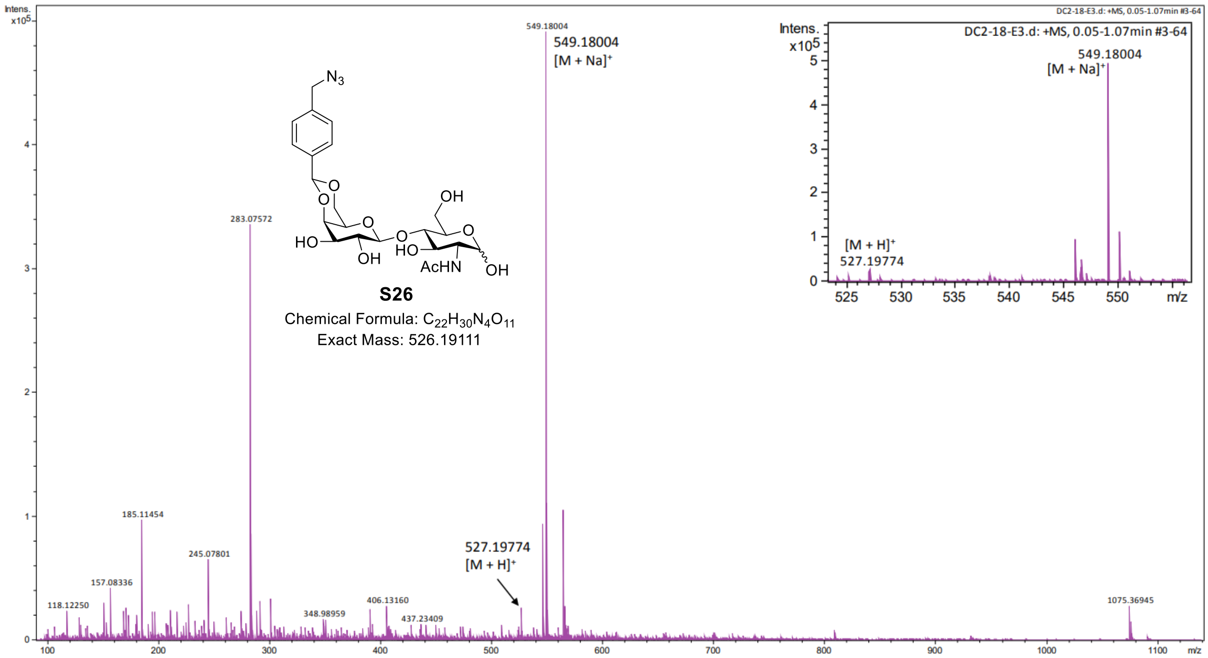

DC2-18-E3.d: +MS, 0.05-1.07min #3-64
549.18004
[M + Na]+
527.19774
[M + H]+
283.07572
185.11454
245.07801
157.08336
118.12250
348.98959
406.13160
437.23409
1075.36945
S26
Chemical Formula: C22H30N4O11
Exact Mass: 526.19111
Intens. x10^5
m/z

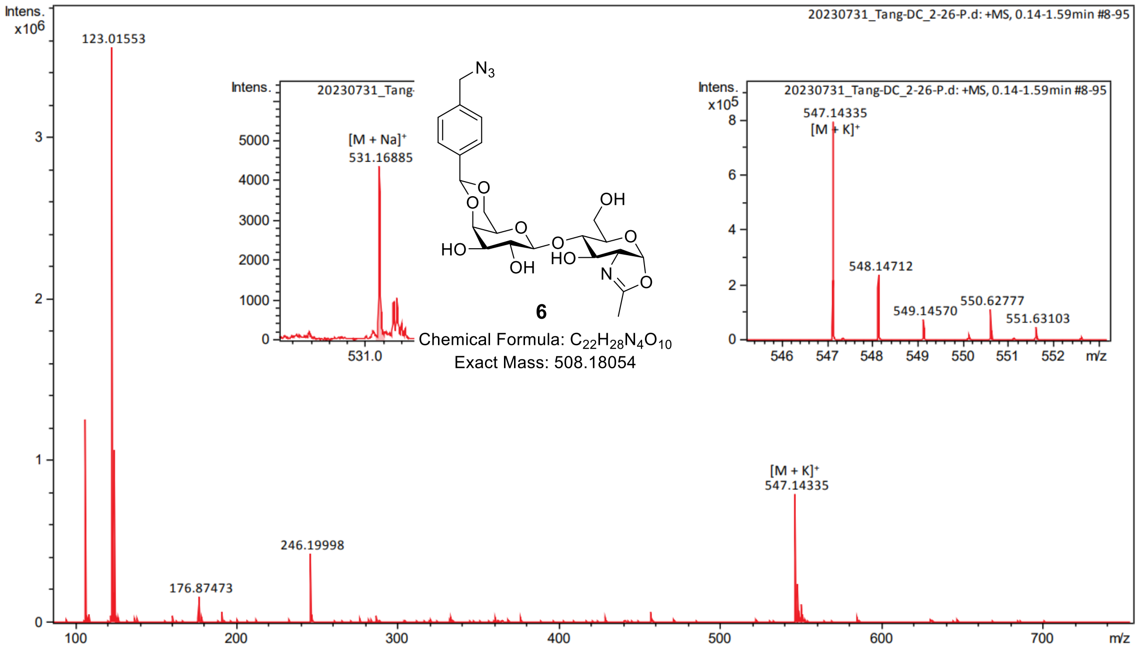

20230731_Tang-DC_2-26-P.d: +MS, 0.14-1.59min #8-95
123.01553
[M + Na]+
531.16885
547.14335
[M + K]+
548.14712
549.14570
550.62777
551.63103
176.87473
246.19998
6
Chemical Formula: C22H28N4O10
Exact Mass: 508.18054
Intens. x10^6
m/z

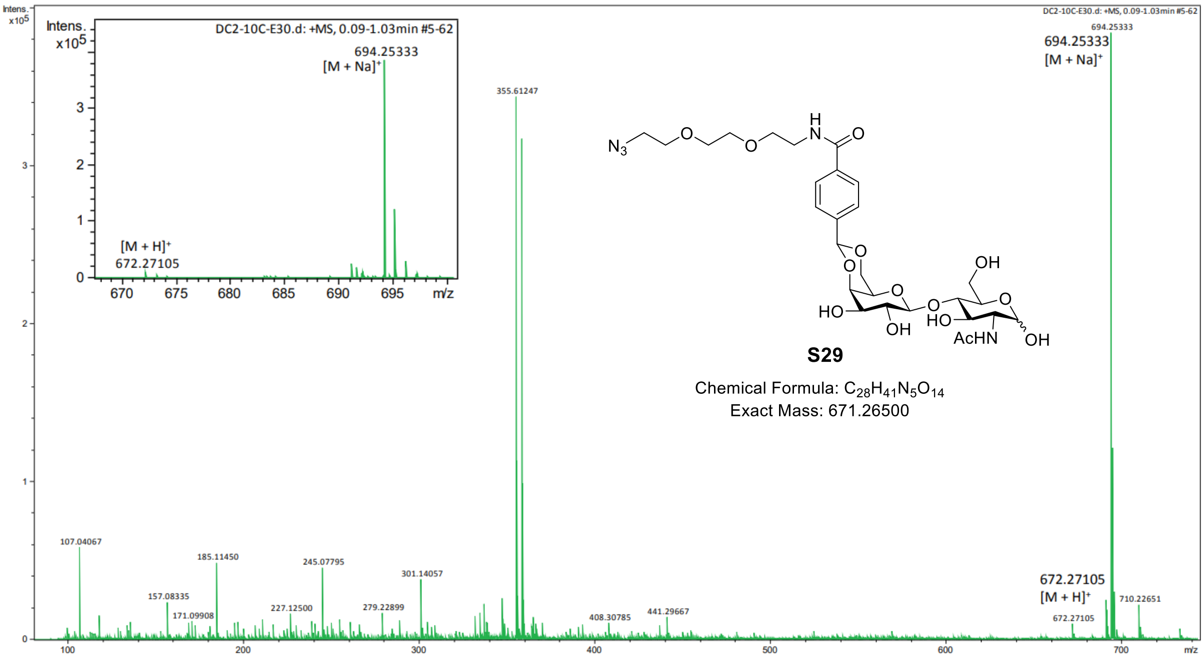
DC2-10C-E30.d: +MS, 0.09-1.03min #5-62
694.25333
[M + Na]+
672.27105
[M + H]+
355.61247
107.04067
185.11450
245.07795
301.14057
157.08335
171.09908
227.12500
279.22899
408.30785
441.29667
710.22651
S29
Chemical Formula: C28H41N5O14
Exact Mass: 671.26500

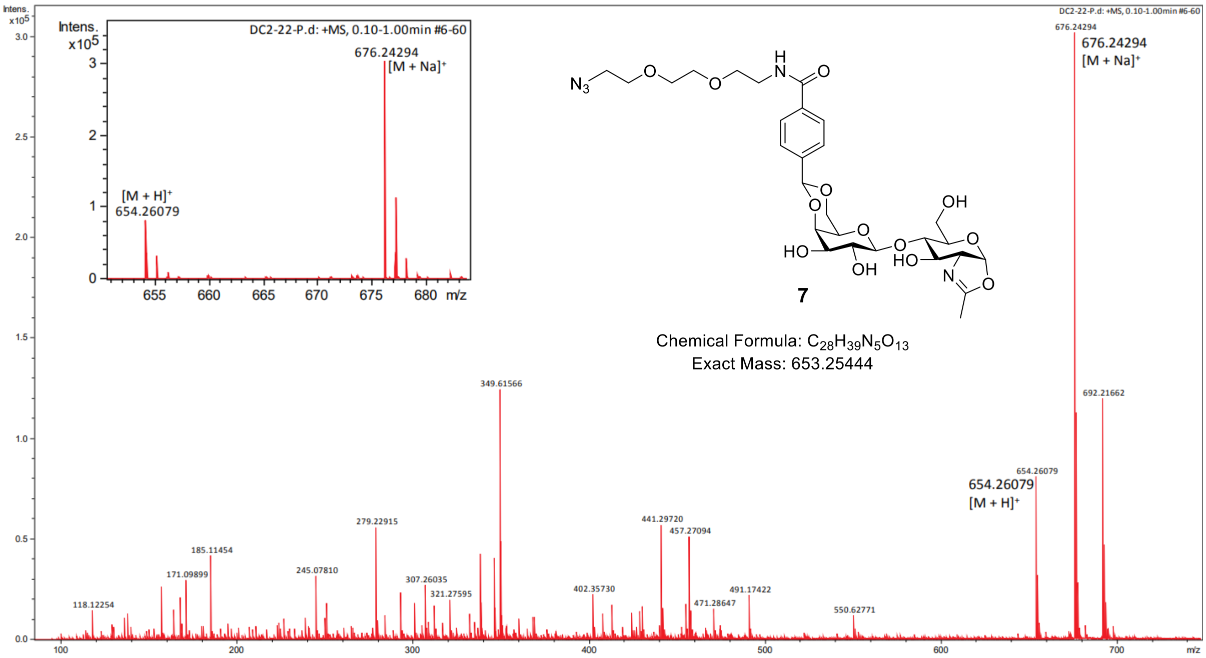
DC2-22-P.d: +MS, 0.10-1.00min #6-60
676.24294
[M + Na]+
654.26079
[M + H]+
692.21662
349.61566
279.22915
441.29720
457.27094
185.11454
171.09899
245.07810
307.26035
321.27595
402.35730
471.28647
491.17422
550.62771
118.12254
7
Chemical Formula: C28H39N5O13
Exact Mass: 653.25444

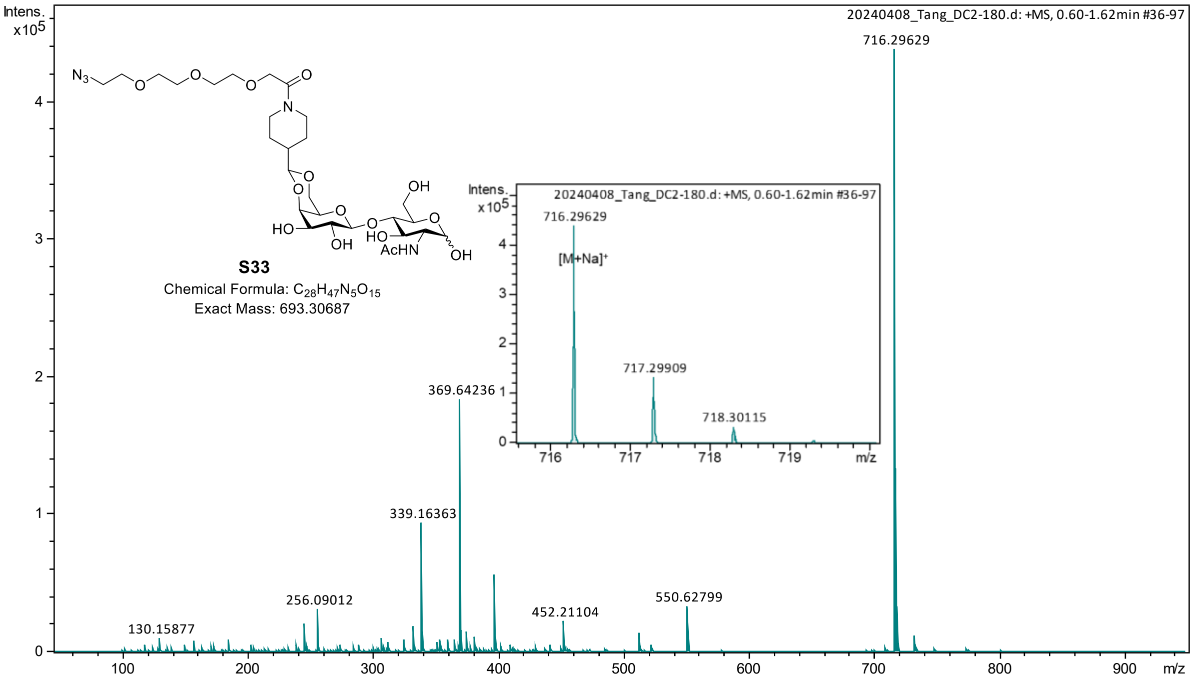
20240408_Tang_DC2-180.d: +MS, 0.60-1.62min #36-97
Intens. x10^5
716.29629
369.64236
339.16363
256.09012
130.15877
452.21104
550.62799
S33
Chemical Formula: C28H47N5O15
Exact Mass: 693.30687
[M+Na]+
717.29909
718.30115
m/z

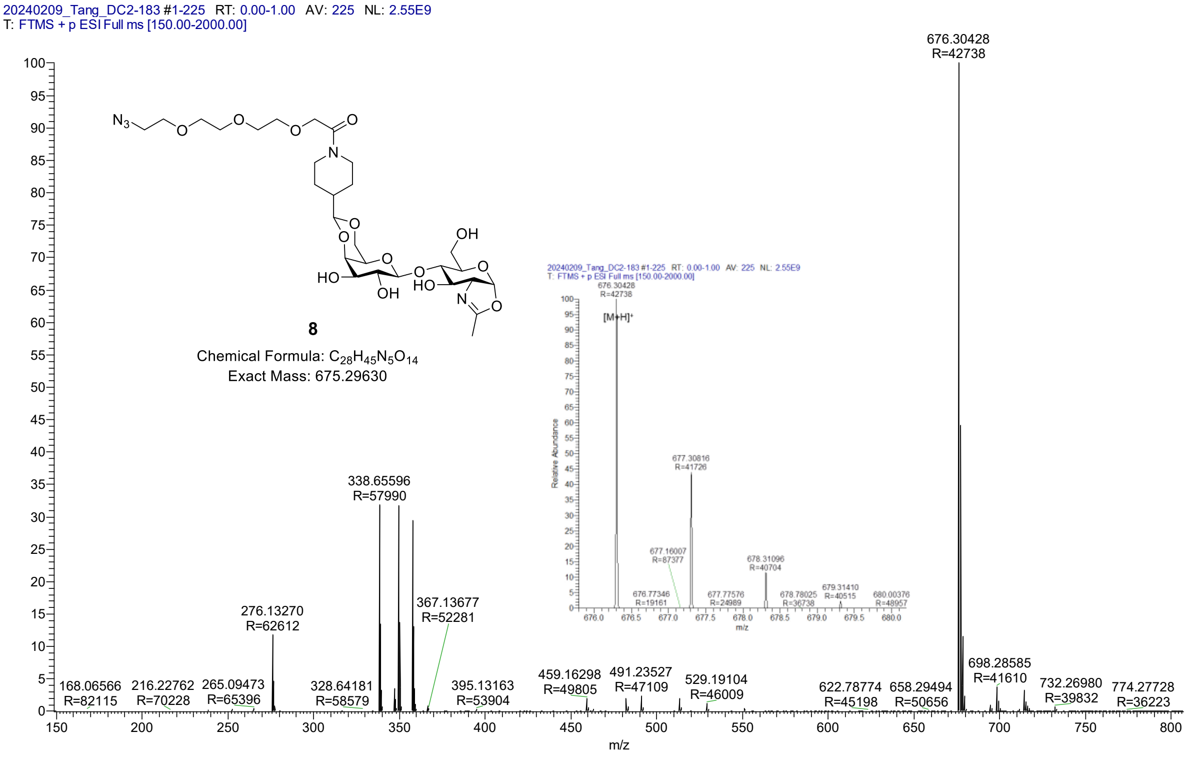
20240209_Tang_DC2-183 #1-225 RT: 0.00-1.00 AV: 225 NL: 2.55E9
T: FTMS + p ESI Full ms [150.00-2000.00]
676.30428
R=42738
8
Chemical Formula: C28H45N5O14
Exact Mass: 675.29630
[M+H]+
338.65596
R=57990
276.13270
R=62612
367.13677
R=52281
168.06566
R=82115
216.22762
R=70228
265.09473
R=65396
328.64181
R=58579
395.13163
R=53904
459.16298
R=49805
491.23527
R=47109
529.19104
R=46009
622.78774
R=45198
658.29494
R=50656
698.28585
R=41610
732.26980
R=39832
774.27728
R=36223
677.30816
R=41726
678.31096
R=40704
m/z

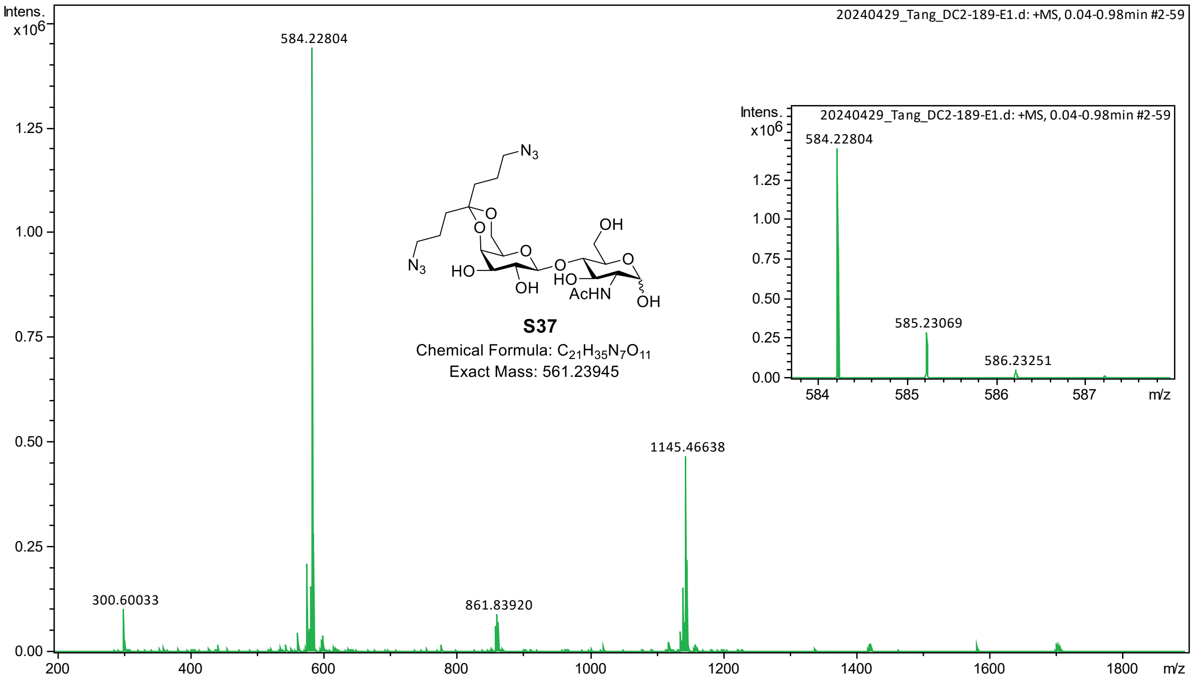

Intens.
x10^6
20240429_Tang_DC2-189-E1.d: +MS, 0.04-0.98min #2-59
584.22804
1145.46638
861.83920
300.60033
585.23069
586.23251
S37
Chemical Formula: C21H35N7O11
Exact Mass: 561.23945
m/z

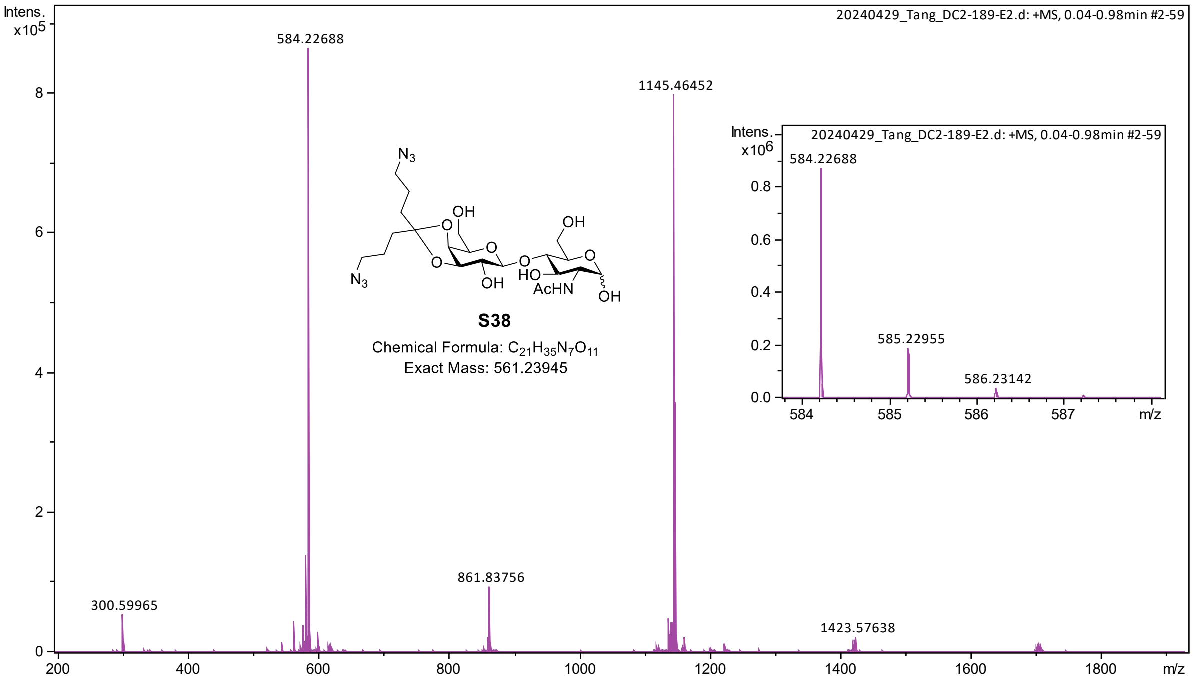

Intens.
x10^5
20240429_Tang_DC2-189-E2.d: +MS, 0.04-0.98min #2-59
584.22688
1145.46452
861.83756
300.59965
1423.57638
585.22955
586.23142
S38
Chemical Formula: C21H35N7O11
Exact Mass: 561.23945
m/z

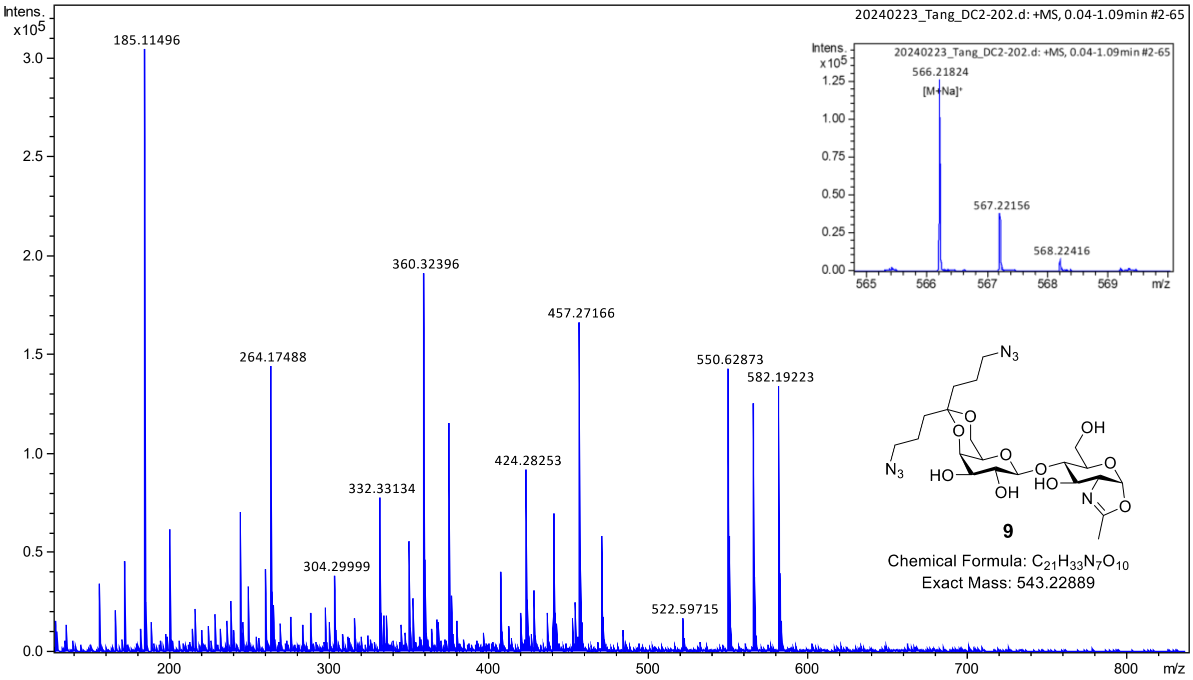

Intens.
x10^5
20240223_Tang_DC2-202.d: +MS, 0.04-1.09min #2-65
185.11496
264.17488
360.32396
332.33134
304.29999
424.28253
457.27166
522.59715
550.62873
582.19223
566.21824
[M+Na]+
567.22156
568.22416
m/z
N3
OH
HO
O
N
9
Chemical Formula: C21H33N7O10
Exact Mass: 543.22889

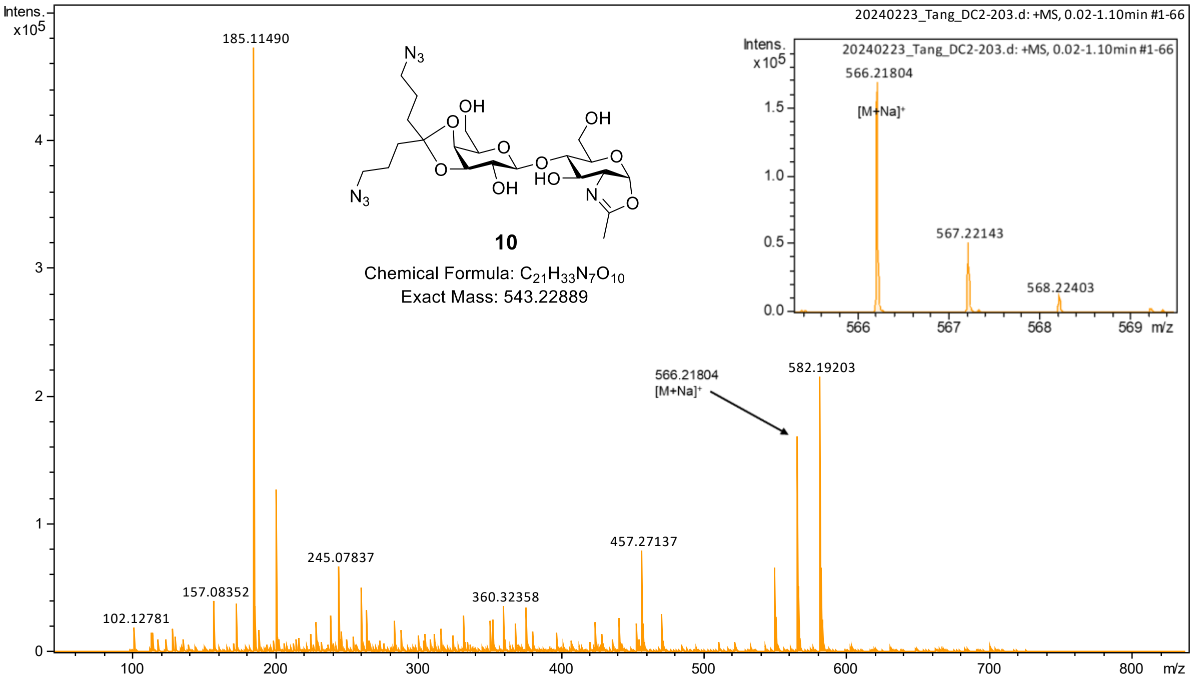

Intens.
x10^5
20240223_Tang_DC2-203.d: +MS, 0.02-1.10min #1-66
185.11490
102.12781
157.08352
245.07837
360.32358
457.27137
566.21804
[M+Na]+
582.19203
567.22143
568.22403
m/z
N3
OH
HO
O
N
10
Chemical Formula: C21H33N7O10
Exact Mass: 543.22889

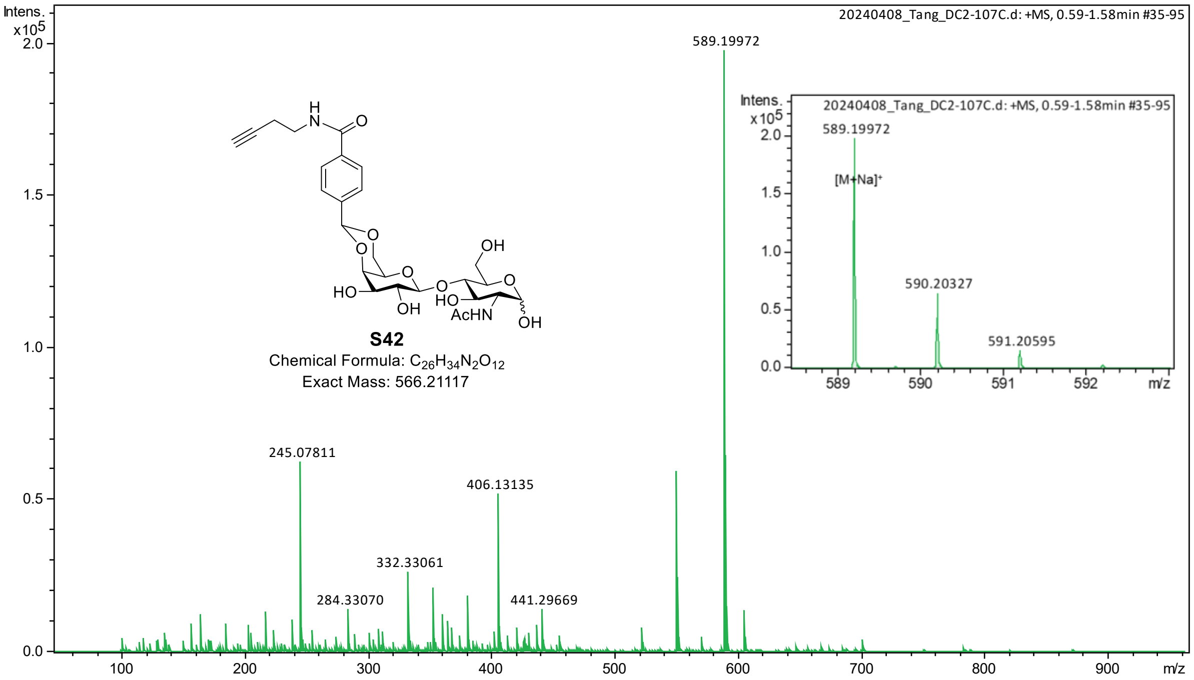
Intens. x10^5
20240408_Tang_DC2-107C.d: +MS, 0.59-1.58min #35-95
589.19972
245.07811
406.13135
332.33061
284.33070
441.29669
S42
Chemical Formula: C26H34N2O12
Exact Mass: 566.21117
[M+Na]+
590.20327
591.20595
m/z

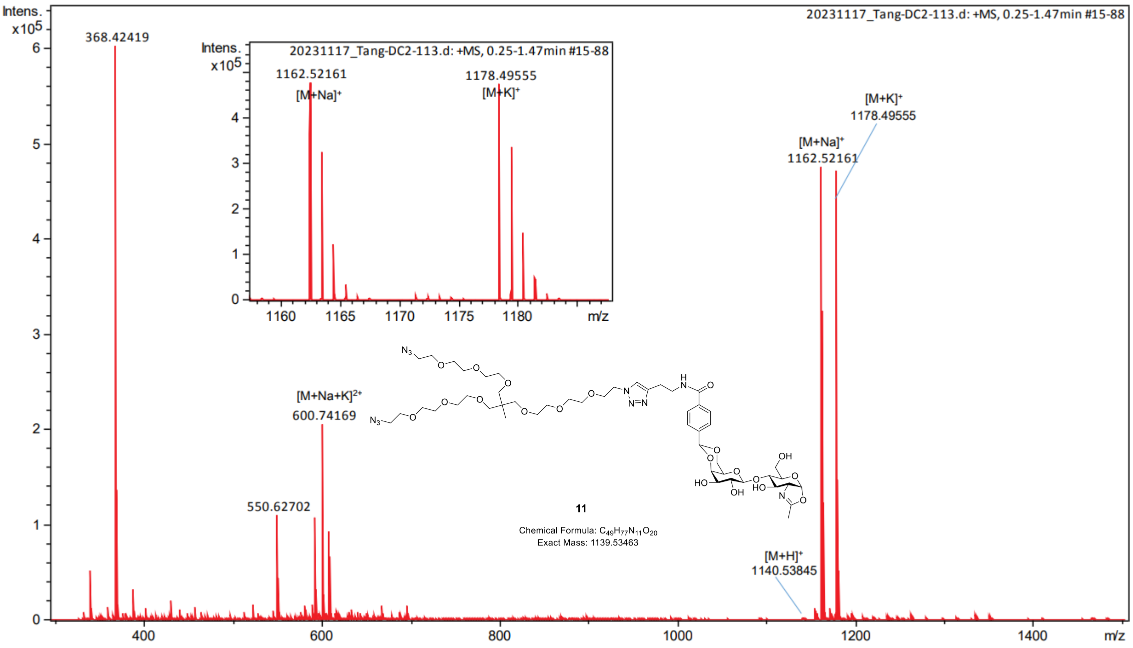
Intens. x10^5
20231117_Tang-DC2-113.d: +MS, 0.25-1.47min #15-88
368.42419
1162.52161
[M+Na]+
1178.49555
[M+K]+
[M+Na+K]2+
600.74169
550.62702
[M+H]+
1140.53845
11
Chemical Formula: C49H77N11O20
Exact Mass: 1139.53463
m/z

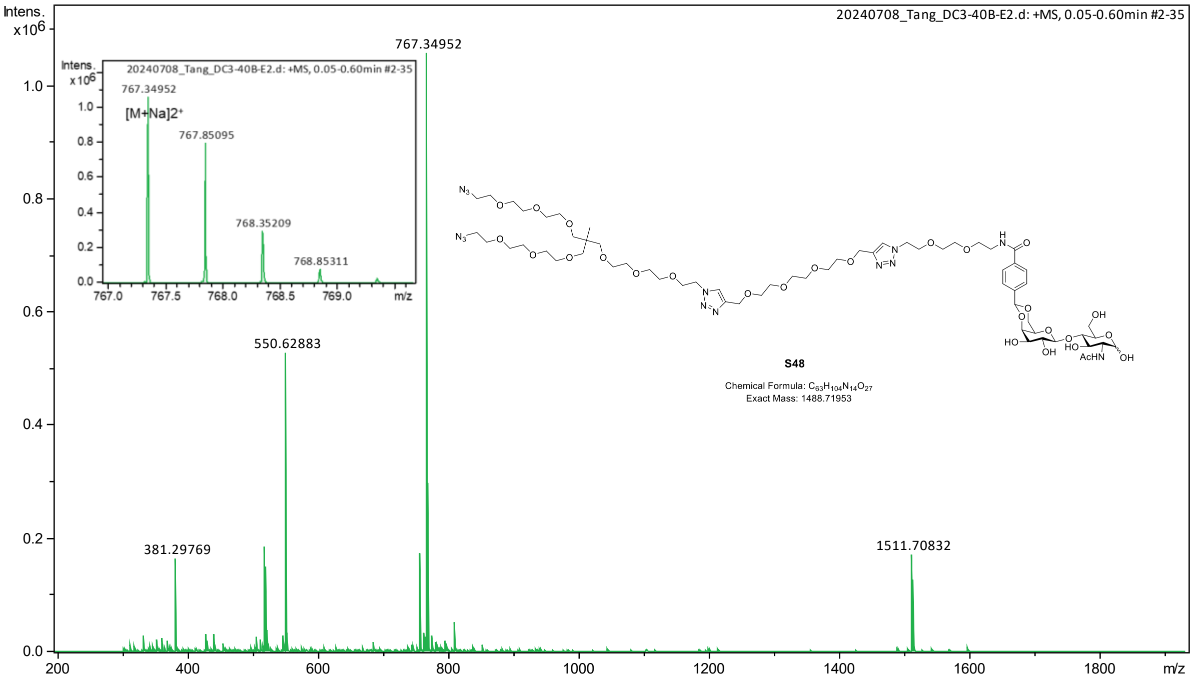

Intens.
x10^6
20240708_Tang_DC3-40B-E2.d: +MS, 0.05-0.60min #2-35
767.34952
[M+Na]2+
767.85095
768.35209
768.85311
550.62883
381.29769
1511.70832
m/z
S48
Chemical Formula: C63H104N14O27
Exact Mass: 1488.71953

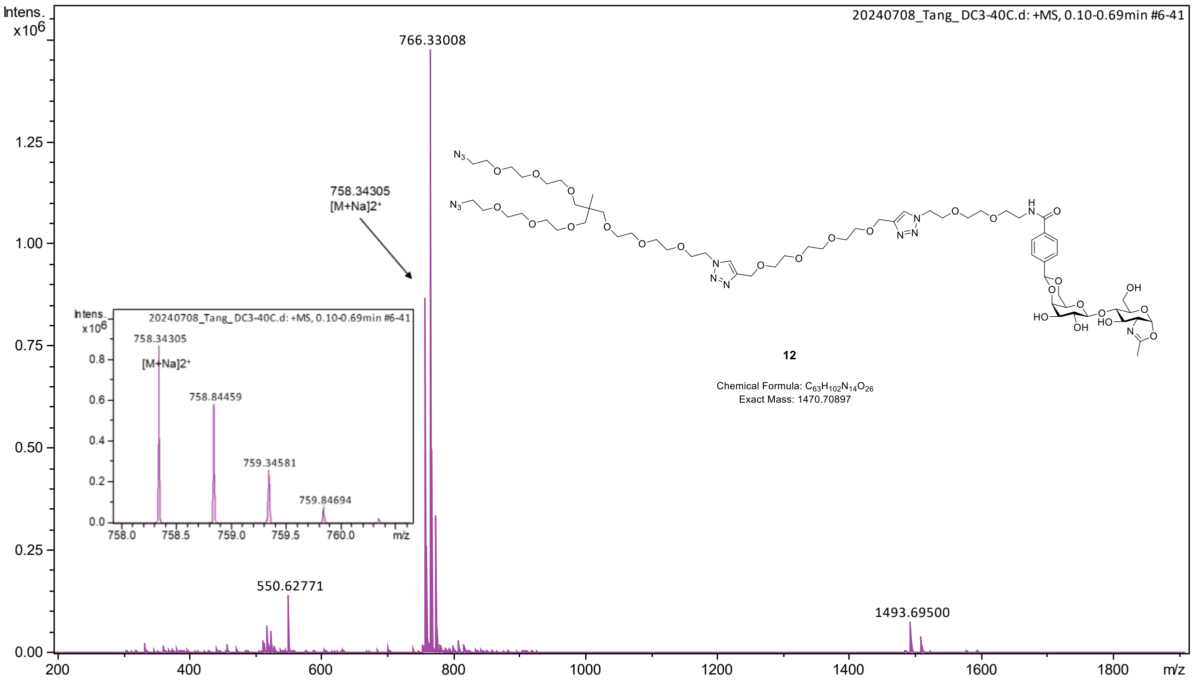

Intens.
x10^6
20240708_Tang_DC3-40C.d: +MS, 0.10-0.69min #6-41
766.33008
758.34305
[M+Na]2+
758.84459
759.34581
759.84694
550.62771
1493.69500
m/z
12
Chemical Formula: C63H102N14O26
Exact Mass: 1470.70897

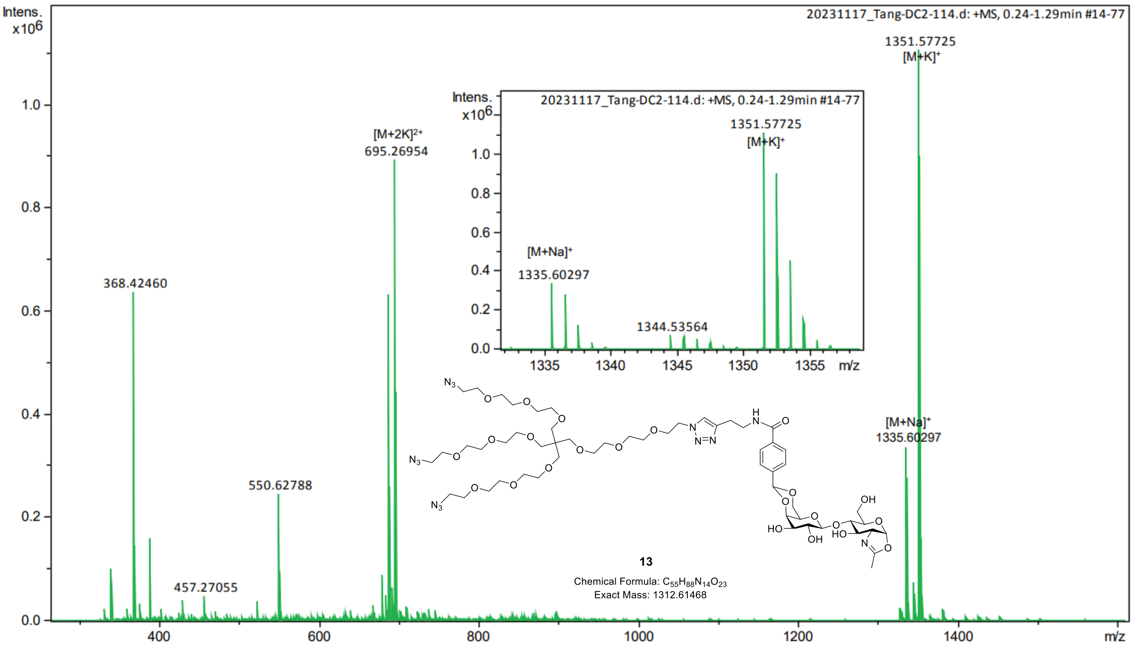

Intens. x10^6
20231117_Tang-DC2-114.d: +MS, 0.24-1.29min #14-77
1351.57725
[M+K]+
[M+2K]2+
695.26954
368.42460
550.62788
457.27055
[M+Na]+
1335.60297
1344.53564
13
Chemical Formula: C55H88N14O23
Exact Mass: 1312.61468
m/z

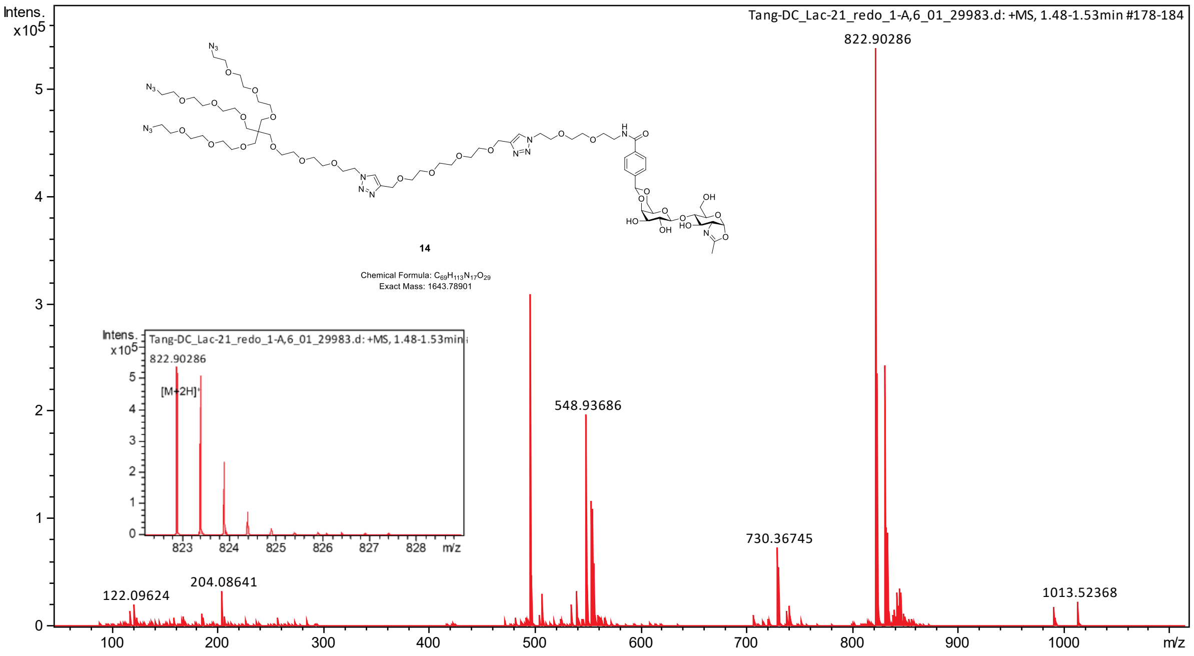

Intens. x10^5
Tang-DC_Lac-21_redo_1-A,6_01_29983.d: +MS, 1.48-1.53min #178-184
822.90286
[M+2H]2+
548.93686
730.36745
204.08641
122.09624
1013.52368
14
Chemical Formula: C69H113N17O29
Exact Mass: 1643.78901
m/z

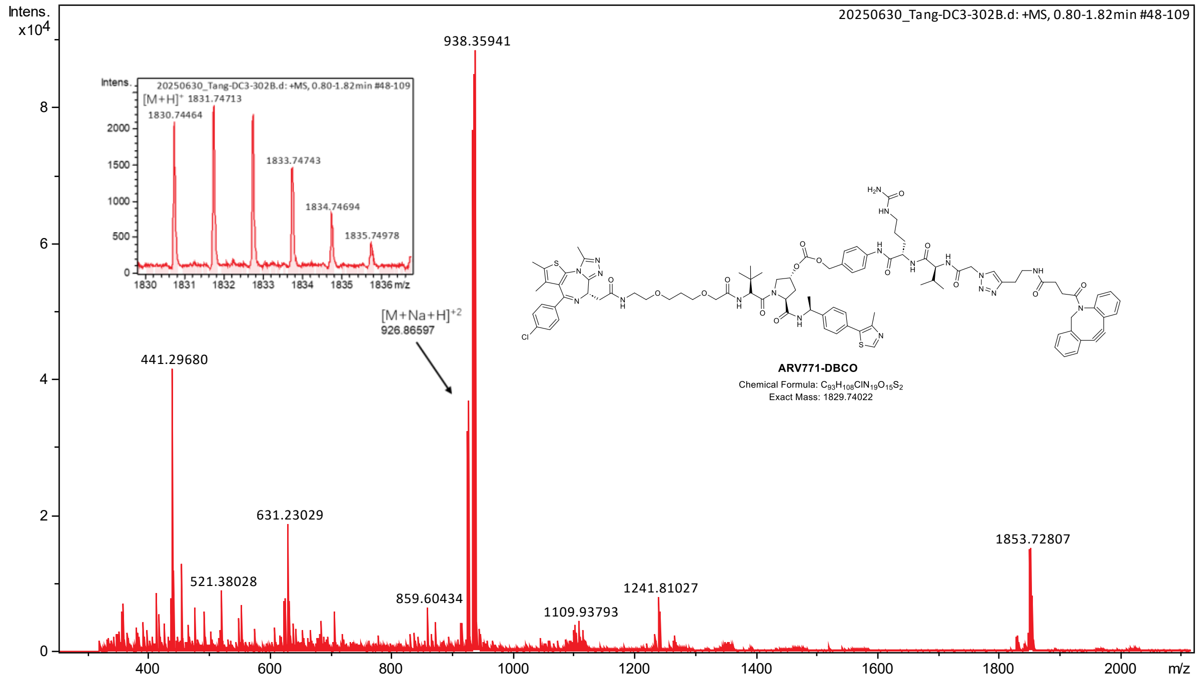

Intens.
x10^4
20250630_Tang-DC3-302B.d: +MS, 0.80-1.82min #48-109
938.35941
[M+H]+ 1831.74713
1830.74464
1833.74743
1834.74694
1835.74978
[M+Na+H]+2
926.86597
441.29680
631.23029
521.38028
859.60434
1109.93793
1241.81027
1853.72807
ARV771-DBCO
Chemical Formula: C93H108ClN19O15S2
Exact Mass: 1829.74022
m/z

Reference

[1] S. Passaro, G. Corso, J. Wohlwend, M. Reveiz, S. Thaler, V. R. Somnath, N. Getz, T. Portnoi, J. Roy, H. Stark, D. Kwabi-Addo, D. Beaini, T. Jaakkola, R. Barzilay, "Boltz-2: Towards Accurate and Efficient Binding Affinity Prediction" *bioRxiv* **2025**, 2025.2006.2014.659707.

[2] W. L. Jorgensen, J. Chandrasekhar, J. D. Madura, R. W. Impey, M. L. Klein, "Comparison of simple potential functions for simulating liquid water" *The Journal of Chemical Physics* **1983**, *79*, 926-935.

[3] G. Bussi, D. Donadio, M. Parrinello, "Canonical sampling through velocity rescaling" *The Journal of Chemical Physics* **2007**, *126*, 014101.

[4] M. Parrinello, A. Rahman, "Polymorphic transitions in single crystals: A new molecular dynamics method" *J. Appl. Phys.* **1981**, *52*, 7182-7190.

[5] U. Essmann, L. Perera, M. L. Berkowitz, T. Darden, H. Lee, L. G. Pedersen, "A smooth particle mesh Ewald method" *The Journal of Chemical Physics* **1995**, *103*, 8577-8593.

[6] T. Darden, D. York, L. Pedersen, "Particle mesh Ewald: An N·log(N) method for Ewald sums in large systems" *The Journal of Chemical Physics* **1993**, *98*, 10089-10092.

[7] B. Hess, H. Bekker, H. J. C. Berendsen, J. G. E. M. Fraaije, "LINCS: A linear constraint solver for molecular simulations" *J. Comput. Chem.* **1997**, *18*, 1463-1472.

[8] M. J. Abraham, T. Murtola, R. Schulz, S. Páll, J. C. Smith, B. Hess, E. Lindahl, "GROMACS: High performance molecular simulations through multi-level parallelism from laptops to supercomputers" *SoftwareX* **2015**, *1-2*, 19-25.

[9] J. Huang, S. Rauscher, G. Nawrocki, T. Ran, M. Feig, B. L. de Groot, H. Grubmüller, A. D. MacKerell, "CHARMM36m: an improved force field for folded and intrinsically disordered proteins" *Nat. Methods* **2017**, *14*, 71-73.

[10] K. Vanommeslaeghe, E. Hatcher, C. Acharya, S. Kundu, S. Zhong, J. Shim, E. Darian, O. Guvench, P. Lopes, I. Vorobyov, A. D. Mackerell Jr, "CHARMM general force field: A force field for drug-like molecules compatible with the CHARMM all-atom additive biological force fields" *J. Comput. Chem.* **2010**, *31*, 671-690.

[11] M. S. Valdés-Tresanco, M. E. Valdés-Tresanco, P. A. Valiente, E. Moreno, "gmx_MMPBSA: A New Tool to Perform End-State Free Energy Calculations with GROMACS" *J. Chem. Theory Comput.* **2021**, *17*, 6281-6291.

[12] J. Wang, Q. Cai, Y. Xiang, R. Luo, "Reducing Grid Dependence in Finite-Difference Poisson–Boltzmann Calculations" *J. Chem. Theory Comput.* **2012**, *8*, 2741-2751.

[13] C. Tan, Y.-H. Tan, R. Luo, "Implicit Nonpolar Solvent Models" *The Journal of Physical Chemistry B* **2007**, *111*, 12263-12274.

[14] R. Nunes, D. Vila-Viçosa, P. J. Costa, "Tackling Halogenated Species with PBSA: Effect of Emulating the σ-Hole" *J. Chem. Theory Comput.* **2019**, *15*, 4241-4251.

[15] X. Zhang, D. E. Green, V. L. Schultz, L. Lin, X. Han, R. Wang, A. Yaksic, S. Y. Kim, P. L. DeAngelis, R. J. Linhardt, "Synthesis of 4-Azido-N-acetylhexosamine Uridine Diphosphate Donors: Clickable Glycosaminoglycans" *J. Org. Chem.* **2017**, *82*, 9910-9915.

[16] X. Zhang, C. Ou, H. Liu, L. X. Wang, "Synthesis and Evaluation of Three Azide-Modified Disaccharide Oxazolines as Enzyme Substrates for Single-Step Fc Glycan-Mediated Antibody-Drug Conjugation" *Bioconjug. Chem.* **2022**, *33*, 1179-1191.

[17] A. Sau, R. Williams, C. Palo-Nieto, A. Franconetti, S. Medina, M. C. Galan, "Palladium-Catalyzed Direct Stereoselective Synthesis of Deoxyglycosides from Glycals" *Angew. Chem. Int. Ed.* **2017**, *56*, 3640-3644.

[18] E. G. Jackson, G. Cutolo, B. Yang, N. Yarravarapu, M. W. N. Burns, G. Bineva-Todd, C. Roustan, J. B. Thoden, H. M. Lin-Jones, T. H. van Kuppevelt, H. M. Holden, B. Schumann, J. J. Kohler, C. M. Woo, M. R. Pratt, "4-Deoxy-4-fluoro-GalNAz (4FGalNAz) Is a Metabolic Chemical Reporter of O-GlcNAc Modifications, Highlighting the Notable Substrate Flexibility of O-GlcNAc Transferase" *ACS Chem. Biol.* **2022**, *17*, 159-170.

[19] J. P. Issa, C. S. Bennett, "A reagent-controlled SN2-glycosylation for the direct synthesis of beta-linked 2-deoxy-sugars" *J. Am. Chem. Soc.* **2014**, *136*, 5740-5744.

[20] D. Cai, F. He, S. Wu, Z. Wang, Y. Bian, C. Wen, K. Ding, "Functional structural domain synthesis of anti-pancreatic carcinoma pectin-like polysaccharide RN1" *Carbohydr. Polym.* **2024**, *327*, 121668.

[21] S. He, F. Gao, J. Ma, H. Ma, G. Dong, C. Sheng, "Aptamer-PROTAC Conjugates (APCs) for Tumor-Specific Targeting in Breast Cancer" *Angew. Chem. Int. Ed.* **2021**, *60*, 23299-23305.
